# SCMBench: benchmarking domain-specific and foundation models for single-cell multi-omics data integration

**DOI:** 10.1038/s41467-026-72570-x

**Published:** 2026-05-02

**Authors:** Yixuan Wang, Yimin Fan, Xuesong Wang, Tingyang Yu, Yongshuo Zong, Xinyuan Liu, Gaoyang Zhong, Meitong Liu, Qing Li, Kin Hei Lee, Khachatur Dallakyan, Zhichao Hu, Yaqian Qi, Junjie Huang, Gengjie Jia, Jiao Yuan, Ting-Fung Chan, Xin Gao, Irwin King, Yu Li

**Affiliations:** 1 Department of Computer Science and Engineering, CUHK, Hong Kong SAR, China; 2 Mohamed bin Zayed University of Artificial Intelligence, Abu Dhabi, United Arab Emirates; 3 Department of Information Engineering, CUHK, Hong Kong SAR, China; 4 School of Informatics, University of Edinburgh, Edinburgh, UK; 5 Department of Forensic Medicine, Tongji Medical College, Huazhong University of Science and Technology, Wuhan, China; 6 Department of Computer Science, HKU, Hong Kong SAR, China; 7 Department of Information Technology & Operations Management, Nanyang Business School, NTU, Singapore, Singapore; 8 Quantitative Methods and Modeling, Baruch College, CUNY, New York, NY USA; 9 JC School of Public Health and Primary Care, Faculty of Medicine, CUHK, Hong Kong SAR, China; 10 Genome Analysis Laboratory of the Ministry of Agriculture and Rural Affairs, Agricultural Genomics Institute at Shenzhen, Chinese Academy of Agricultural Sciences, Shenzhen, China; 11 GMU-GIBH Joint School of Life Sciences, The Guangdong-Hong Kong-Macau Joint Laboratory for Cell Fate Regulation and Diseases, Guangzhou National Laboratory, Guangzhou Medical University, Guangzhou, China; 12 School of Life Sciences, CUHK, Hong Kong SAR, China; 13 State Key Laboratory of Agrobiotechnology, The Chinese University of Hong Kong, Hong Kong SAR, China; 14 Computer Science Program, Computer, Electrical and Mathematical Sciences and Engineering Division, King Abdullah University of Science and Technology (KAUST), Thuwal, Kingdom of Saudi Arabia; 15 Center of Excellence for Smart Health (KCSH), King Abdullah University of Science and Technology (KAUST), Thuwal, Kingdom of Saudi Arabia; 16 Center of Excellence on Generative AI, King Abdullah University of Science and Technology (KAUST), Thuwal, Kingdom of Saudi Arabia; 17 The CUHK Shenzhen Research Institute, Hi-Tech Park, Nanshan, Shenzhen, China

**Keywords:** Gene expression analysis, Data integration, Bioinformatics, Gene expression profiling

## Abstract

Recent advancements in single-cell sequencing technologies have led to the generation of vast amounts of multi-omics data, spurring the development of numerous integration tools. While multi-omics integration has significantly advanced cell research, there is still a lack of comprehensive evaluations and guidelines for these tools. This study benchmarks Domain-specific Models (DMs) and Foundation Models (FMs) for multi-omics data integration, assessing 23 methods with optimized hyperparameters on integration accuracy, biomarker detection, trajectory inference, and quantitative batch effect correction. We address current gaps in assessing the efficacy and limitations of FMs compared to DMs in the multi-omics integration task. Importantly, our comprehensive analysis goes beyond basic integration accuracy, focusing on the preservation of cellular characteristics, transcriptomic biomarkers, epigenomic regulatory elements, and development trajectories. This holistic approach enables researchers to extract meaningful insights from integration results, facilitating a deeper understanding of individual cells. Generally, we find FMs fall short of state-of-the-art DMs in this field. To bridge this performance gap, we propose a lightweight adaptation strategy that enhances their effectiveness in this task. Our findings serve as a guide for researchers in selecting suitable integration methods for specific single-cell analysis objectives and provide insights for future model design.

## Abstract (web summary)

Integrating single-cell multi-omics data remains challenging. Here, the authors benchmark 23 integration methods including foundation models across diverse datasets, revealing task-specific trade-offs and proposing an adaptation strategy that enhances foundation model performance.

## Introduction

The proliferation of multi-omics single-cell sequencing technologies, such as the transcriptome (scRNA-seq)^1,2^, chromatin accessibility (scATAC-seq)^3,4^, and DNA methylation (snmC-seq, sci-MET)^5,6^, has revolutionized biological research at the single-cell level. Integrating these diverse data sources provides comprehensive insights across different modalities, facilitating the identification of clinically relevant biomarkers, inference of cellular trajectories, and the discovery of regulatory interactions within various cell types^7–10^. Beyond the primary focus on using cell type labels to quantify integration accuracy, it is crucial to preserve informative biological features such as cellular characteristics and biological processes. Additionally, significant batch effects in single-cell sequencing datasets, along with the independent and unpaired nature of different omics measurements, pose substantial challenges for multi-omics data integration^11,12^.

To tackle these challenges, specialized tools have been developed to integrate multi-omics data and align diverse omics information into a joint latent space. These tools, designed by experts based on extensive knowledge of single-cell multi-omics integration, are referred to as DMs. DMs can be broadly classified into two categories based on their underlying algorithms: statistical-based methods, which rely on analytical mathematical operations, and deep learning-based methods, which harness the computational power of deep neural networks. Statistical integration methods encompass factor analysis^13,14^, matrix factorization^15–17^, manifold alignment^18–20^, weighted nearest neighbor algorithms^21^, and dictionary learning algorithms^22^. These methods are widely employed to uncover patterns and relationships within the data. In contrast, deep learning-based approaches are designed to capture intricate patterns and dependencies in the data, offering a more comprehensive understanding of cell-specific regulatory networks and enabling the identification of clinically relevant biomarkers^11,23–27^. Examples include graph-guided neural networks^24,27^ and deep generative networks^26,27^. Recently, FMs have made significant strides in single-cell research. Models such as Geneformer^28^ focus on gene and cell state classification, while others like scGPT^29^ and scFoundation^30^ are designed to handle multiple tasks in single-cell research. Unlike these models, which are limited to human cells, UCE^31^ introduces a comprehensive biological latent space capable of encompassing all cellular entities, regardless of tissue origin or species. Pre-training on massive and diverse datasets, FMs provide powerful biological representations that can serve as a foundation for various downstream applications, though task-specific adaptation is typically required to fully leverage their capabilities for specialized tasks.

As the field of single-cell multi-omics integration expands, comprehensive evaluations are critical to guide method selection in practical experiments. However, given the rapid advancements in deep learning-based approaches in this field and the increasing importance of extensive downstream analyses, a more comprehensive benchmark involving FMs is needed^32–35^. To fill this gap, we propose SCMBench, a holistic benchmarking study of multi-omics integration methods, incorporating 23 methods and six real datasets across various downstream tasks. This comprehensive framework allows us to thoroughly evaluate the efficacy and limitations of FMs compared to specialized deep learning approaches in multi-omics integration, providing key insights into their relative strengths and potential applications.

We assess cell type label inference accuracy from the integrated representations of these methods, considering both paired and unpaired scenarios. To determine their ability to extract informative and complex biological features like biomarkers and biological processes from multiple modalities^36,37^, and tackle the nested batch effects in these data^7^, we conduct comprehensive evaluations on three essential downstream tasks: biomarker detection, trajectory inference, and batch effect correction. For biomarker detection, we implement a multi-level assessment framework that evaluates both transcriptomic and epigenomic features. At the transcriptomic level, we assess each method’s ability to identify consistent cell-type-specific marker genes and further validate these findings against established reference markers from the CellMarker 2.0 database for the PBMC dataset. At the epigenomic level, we quantify each method’s performance in detecting differentially accessible chromatin regions and their associated regulatory motifs, providing a comprehensive view of how integration methods preserve biological signatures across modalities. In trajectory inference, we evaluate the methods’ capability to reconstruct developmental or differentiation trajectories from the integrated representations. Then, we examine the effectiveness of these methods in batch effect correction, aiming to mitigate the confounding effects introduced by technical variations across datasets. We include simulated batch effects for a more thorough and quantitative evaluation.

Through extensive experiments, we find that FMs generally underperform compared to DMs for integrating single-cell multi-omics data. To systematically assess the potential of FMs in multi-omics integration, we evaluate multiple adaptation strategies, including using scVI as an adaptation module for FM embeddings, alongside fine-tuning and parameter-efficient tuning approaches. We also provide significance reports and a scalability analysis, offering a comprehensive evaluation of the current state of single-cell multi-omics data integration. These findings assist researchers and practitioners in selecting the most appropriate integration methods based on factors such as data characteristics, paired or unpaired scenarios, and scalability across different species. We believe that SCMBench will also serve as a valuable guide for future model design in this field, particularly for the development and optimization of FMs.

## Results

### SCMBench provides a comprehensive benchmarking pipeline

**Fig. 1:**
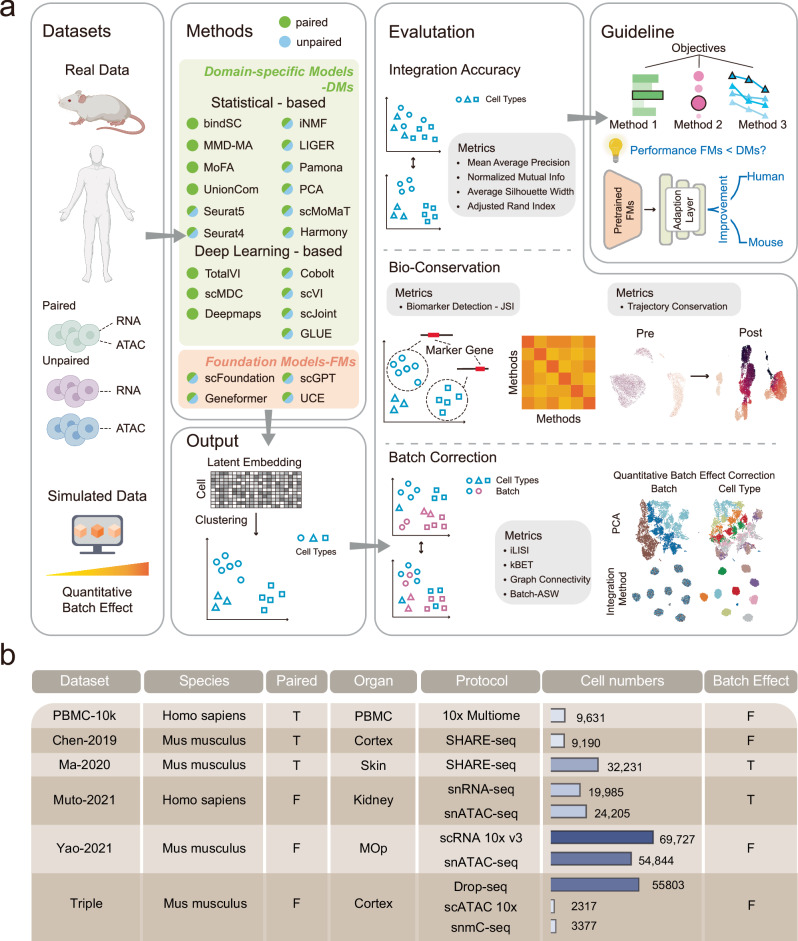
SCMBench workflow and summary characteristics of the datasets. **a** Overview of the SCMBench pipeline. Paired and unpaired real multi-omics datasets profiling cells from four organs and two species are used for benchmarking, assisted with simulated data in varying degrees of batch effect. 19 DMs as well as four FMs are examined; the colors of the bullet points indicate the methods' applicability to paired and unpaired data. From another perspective, 12 of them are statistical-based methods and 11 of them are deep learning-based methods. Latent embeddings and cell-type inferences are acquired for performance evaluation, focusing on integration accuracy, bio-conservation, and batch correction. We then provide guidelines for the software selection under various conditions and strategies for boosting the FMs' performance on this task. **b** Detailed characteristics of the real dataset considered in this work. The cell numbers of the two modalities are equal for the paired datasets. We annotate one of them for simplicity. `T' and `F' stand for `True' and `False' respectively. Created in BioRender. Group, A. (2026) https://BioRender.com/4n82nqs.

In this study, we benchmark in total 23 integration methods, covering a variety of underlying algorithms. Statistical-based methods based on analytical mathematical operations include PCA^38^, MOFA^14^, bindSC^39^, LIGER^40^, online-iNMF^16^, scMoMaT^17^, MMD-MA^18^, Pamona^19^, UnionCom^20^, Harmony^41^, Seurat4^21^, and Seurat5^22^. Methods utilizing the computational power of deep learning include scMDC^23^, scJoint^24^, GLUE^11^, Cobolt^25^, scVI^42^, TotalVI^27^, DeepMAPS^43^, and scGPT^29^. Notably, scGPT is the only FM that achieves the paired multi-omics data integration task. We additionally modify UCE^31^, Geneformer^28^, and scFoundation^30^ to extend the evaluation to other FMs. To comprehensively evaluate FMs for multi-omics integration, we evaluate multiple adaptation strategies, including adapter-based integration that combines FM embeddings with scVI’s framework, fine-tuning, and parameter-efficient tuning approaches. We implement these adaptations as standalone methods and benchmark them alongside other integration methods throughout our pipeline. The classification and detailed information of the models can be found in Fig. 1a and Supplementary Table 2.

Due to the token constraints of pre-trained FMs, which are primarily designed to process gene expression data, we cannot directly input scATAC-seq peak features. Instead, we transform chromatin accessibility data into a gene-centric format using a biologically informed approach implemented in the MAESTRO package^44^. This approach weights peak signals based on their distance to transcription start sites, reflecting the established relationship between chromatin accessibility and gene regulation. To ensure the validity of this transformation, we perform supplementary analyses on the *PBMC-10k* dataset (Supplementary Fig. 1), confirming that the MAESTRO-derived activity matrix preserves key biological structure and gene-level correspondence with RNA. We additionally compare MAESTRO with alternative gene-activity derivation methods, including Seurat4^21^ and the cross-modal translation model BABEL^45^. The BABEL-translated activity matrix also demonstrates strong consistency with gene expression and provides comparable FM-compatible representations. Further comparisons of FM performance across datasets (Supplementary Figs. 16–17) show that MAESTRO and BABEL each have dataset-dependent advantages and neither uniformly dominates. Together, these results indicate that transforming ATAC-seq data into activity matrices offers a reasonable and biologically grounded strategy for adapting chromatin accessibility profiles to FM processing, while multiple alternative implementations remain feasible and validated.

Although paired datasets have become more available with the advancements in multi-omics technologies, most data remain unpaired, with the two omics representing distinct cell groups acquired by single-cell sequencing technologies. Unpaired datasets, despite their higher quality and larger throughput, create greater challenges for most integration methods to align different cell samples from distinct omics feature spaces. To cover a wide range of application scenarios and thoroughly evaluate different methods, we carefully select three paired datasets and two unpaired datasets that are both representative and popular. Our selection criteria take into account various factors, including species diversity, organ/tissue diversity, sample size, batch effects, and sequencing techniques (Fig. 1b). The first paired dataset, *PBMC-10k*^46^, provides a relatively simple case for predicting cell types as PBMC types are well-separated, allowing for an evaluation and comparison of the near-optimal performance of all methods. The second and third datasets, *Chen-2019*^47^ and *Ma-2020*^48^, profile brain cells of *Mus musculus*, composed of less distinguishable cell types, increasing the difficulty level and allowing us to assess the consistency of method performance. The two unpaired datasets, *Muto-2021*^8^ profiling human kidney cells and *Yao-2021*^49^ profiling MOp cells of *Mus musculus*, are utilized for evaluating methods designed for unpaired omics integration only. Notably, datasets *Ma-2020* and *Muto-2021* include multiple batches, making them suitable for batch correction experiments. Furthermore, we have expanded our evaluation to include a triple-omics integration benchmark using single-cell RNA-seq, scATAC-seq, and DNA methylation data from mouse cortex tissue, providing insights into DMs’ performance in more complex multi-modal integration scenarios^5,50^. More data preprocessing details can be found in the Methods and Supplementary Data 1. To further analyze results under a series of continuously varying conditions, we generate simulated paired data with progressively increasing batch effect sizes to explore the upper limits of method performance.

The overall benchmark pipeline is demonstrated in Fig. 1a. Using both real and simulated multi-omics datasets, we evaluate the performance of 19 DMs and four FMs on integration accuracy, biological information conservation, and batch effect correction. For biological information conservation, we conduct two detailed downstream analyses: biomarker detection and trajectory inference. A total of 13 metrics are considered in this study.

### Benchmarking data integration on both paired and unpaired datasets

**Fig. 2:**
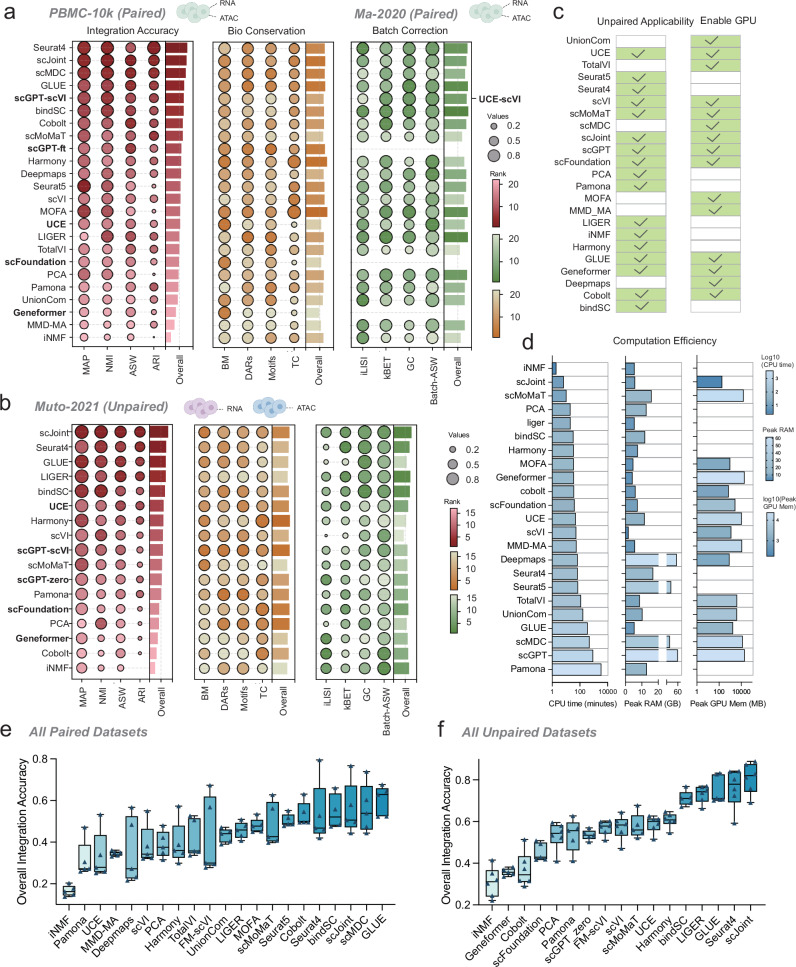
Benchmarking results of the integration accuracy. All metrics are the higher the better. **a**, **b** Benchmarking results on the paired and unpaired datasets. Metrics are categorized into three groups: integration accuracy, bio-conservation, and batch correction. For dataset-specific results, FM-scVI denotes the specific foundation model combined with scVI: scGPT on *PBMC-10k* and the adapted UCE on *Ma-2020*. scGPT-ft refers to the fine-tuned version of scGPT, while scGPT-zero is the zero-shot version. **c** Applicability of integration methods to unpaired data and availability of GPU acceleration for each method. **d** Computational efficiency evaluated on the full *PBMC-10k* dataset, showing processing time and memory requirements across methods. **e**, **f** Overall integration accuracy scores across all paired and unpaired datasets, respectively. For overall metrics, FM-scVI represents the best-performing foundation model combined with scVI, which varies across datasets. Datasets with batch information are split into smaller ones to minimize the impact of batch effects on the integration metrics. Methods are ranked according to the mean performance. Box plots (**e**, **f**): centre line, median; box bounds, 25th--75th percentiles; whiskers, minimum and maximum values. Triangles represent individual dataset scores (*n* = 5 for paired datasets; *n* = 6 for unpaired datasets). Source data are provided as a Source Data file. Created in BioRender. Group, A. (2026) https://BioRender.com/en3gmtr.

We first evaluate integration accuracy across 19 DMs and four FMs using paired datasets to establish their baseline performance with four metrics. Figure 2a illustrates the detailed scores and rankings for the *PBMC-10k* dataset. The overall score represents the mean of these four integration accuracy metrics. scJoint consistently ranks among the top two methods across all integration accuracy metrics for *PBMC-10k*. While Seurat5 achieves the highest MAP score, surpassing Seurat4, it is outperformed by Seurat4 in NMI, ASW, and ARI metrics. The top three methods for *PBMC-10k* are Seurat4, scJoint, and scMDC, with overall integration accuracy scores of 0.7952, 0.7671, and 0.7391, respectively. We evaluate four representative FMs (scGPT, UCE, Geneformer, and scFoundation) on the multi-omics data integration task. Among these, only scGPT originally provides both fine-tuning and zero-shot learning implementations for multi-omics integration. The other three methods require a zero-shot learning approach to obtain embeddings separately for different omics. To thoroughly investigate how fine-tuning influences integration performance, we benchmark different depths of transformer layers and compare loading pretrained parameters versus re-training from scratch. We also compare performance using scGPT’s whole-human model and blood-specific model. Interestingly, our analysis reveals that pretrained weights do not provide substantial benefits compared to training from scratch, suggesting that for this specific multi-omics integration task, the benefits may primarily come from the model’s capacity to learn task-specific representations during fine-tuning rather than leveraging pretrained knowledge. The scGPT blood model generally outperforms the human model under the same settings, suggesting that models fine-tuned with datasets similar in cellular context to their training data typically exhibit competitive performance. Among FMs, scGPT and UCE demonstrate superior performance, with scGPT-scVI achieving results comparable to GLUE. Figure 2e presents the overall paired performance across datasets. GLUE demonstrates a consistent overall score of 0.599 with relatively low variance, indicating reliable performance across diverse conditions. Critical difference diagrams in Supplementary Fig. 14g reveal overlapping statistical groups. While GLUE ranks first, it does not significantly outperform scJoint and other top methods. Conversely, scJoint, despite ranking second, shows no significant advantage over the remaining methods. This suggests that GLUE occupies a unique position by grouping with top performers while maintaining a consistent first-place ranking, whereas scJoint bridges the top and middle-tier methods. Based on this statistical grouping pattern and GLUE’s stable top-tier performance across diverse datasets, we conclude that GLUE emerges as the most consistently reliable method for paired multi-omics integration.

Figure 2c displays the 17 methods that can be applied to unpaired integration scenarios. For these applicable methods, we calculate four integration scores and visualize the detailed performances for *Muto-2021* in Fig. 2b. To eliminate batch effect influence on integration accuracy, we present only the integration accuracy of *Muto-2021-Batch1*, with additional results shown in Supplementary Fig. 2. Notably, scJoint outperforms all other methods across all metrics on this dataset, leading by more than 10% compared to the next best method, Seurat4. Furthermore, scJoint consistently achieves the highest overall integration accuracy across all unpaired datasets, suggesting it is the most effective solution for such scenarios. However, the critical difference diagrams in Supplementary Fig. 14h indicate that scJoint’s superiority is not statistically significant. Regarding FMs’ zero-shot performance, UCE distinguishes itself as the only model capable of handling the *Yao-2021* mouse dataset and demonstrates superior performance across all unpaired datasets. This advantage stems from UCE’s training on a diverse corpus of cell atlas data from various species in a fully self-supervised manner without data annotations. Consequently, it exhibits less dependence on paired information between samples during multi-omics integration tasks. As a result, compared to other FMs, UCE provides more accurate cell embeddings, contributing to its superior performance.

To further assess the versatility and robustness of existing multi-omics integration methods, we extend our benchmarking to the *triple-omics* dataset comprising scRNA-seq, scATAC-seq, and DNA methylation data derived from mouse cortex. Seven methods capable of handling these three modalities are evaluated. FMs are excluded due to the distributional mismatch between continuous methylation *β*-values and the sparse count data these models are trained on. Integration performance is assessed using established batch correction metrics, where omic modality is treated as a surrogate for batch labels (Supplementary Fig. 3a). In parallel, we evaluate cell type resolution by measuring the accuracy of clustering after integration (Supplementary Fig. 3b). Among the tested methods, GLUE consistently achieves the highest scores in both modality alignment and cell type clustering, demonstrating its strong capacity to integrate heterogeneous omic layers and preserve biologically meaningful structures.

Computational efficiency represents a critical factor in method selection, particularly for large-scale multi-omics analyses that can strain computational resources. To provide comprehensive guidance for practical applications, we conduct detailed measurements of wall clock time, CPU time, RAM, and GPU VRAM consumption across all methods in a standardized testing environment (detailed hardware specifications are provided in Supplementary Data 5). As shown in Fig. 2d, methods vary dramatically in their computational demands. We sort the results by CPU processing time, revealing that computational demands generally scale with model complexity. scJoint also performs efficiently with moderate processing times and notably low peak RAM and GPU memory usage, making it suitable for resource-constrained environments. In contrast, fine-tuning scGPT requires substantially longer processing times and higher memory consumption, presenting potential barriers for researchers with limited computational resources. Our adapter-based integration approach (e.g., scGPT-scVI) maintains computational efficiency similar to standard scVI, offering a significant advantage over direct FM fine-tuning. This strategy leverages pre-trained biological knowledge while avoiding the computational burden of large FMs, demonstrating a cost-effective approach to harnessing FMs’ capabilities.

In summary, GLUE and scJoint emerge as the most recommended methods for multi-omics integration tasks. GLUE excels in paired datasets, while scJoint leads in unpaired scenarios with relatively low time complexity compared to GLUE. FMs in zero-shot mode exhibit inferior performance compared to specialized deep learning methods, but proper fine-tuning and adapter-based integration strategies demonstrate promising improvements in integration accuracy while maintaining reasonable computational efficiency. However, challenges in unpaired scenarios and cross-species applications persist. Overcoming these obstacles may unlock opportunities and enhance the effectiveness of FMs in broader biological contexts.

### Preservation of cellular identity supported by biomarker discovery

Identifying biomarkers is essential for characterizing and understanding distinct cell populations^36,51^. These biomarkers, typically genes with specific or predominant expression in particular cell types, serve as critical indicators for cell identification and function^52^. To assess the efficacy of various integration methods in biomarker detection, we apply each method to generate latent spaces, excluding cells with missing values. We evaluate biomarker discovery performance using three complementary metrics. For the BioMarker detection (BM) score, we use a K-Nearest Neighbors classifier on the integrated latent representations to categorize cell types, then identify candidate biomarkers with Scanpy’s rank_genes_groups function^53^ and quantify consistency across methods using the Jaccard similarity index (JSI). To provide a more comprehensive multi-omics perspective, at the scATAC-seq level, we identify DARs and perform motif enrichment analysis using GimmeMotifs^54^ to detect enriched transcription factor binding sites, calculating consistency scores for both metrics using the JSI approach. Together, these three metrics provide a comprehensive evaluation framework that spans both transcriptomic (gene expression biomarkers) and epigenomic (chromatin accessibility and regulatory motifs) layers of cellular identity, enabling assessment of integration methods across complementary biological dimensions.

**Fig. 3:**
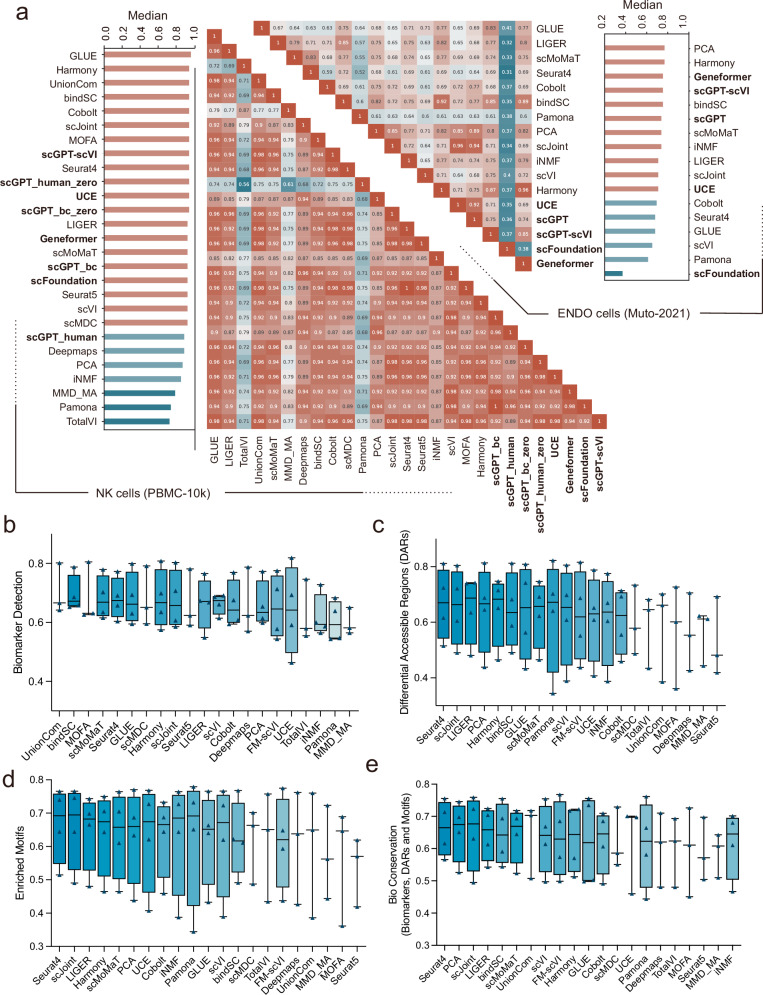
Comparison of the cellular identity preservation performance of DMs and FMs through biomarker detection. **a** Comparative analysis of biomarker detection performance across various computational methods. Heatmaps display the JSI for biomarker detection between methods, with darker red shades representing higher overlap. NK cells in *PBMC-10k* and ENDO cells in *Muto-2021* are taken as examples. Methods are ranked according to their BM score. **b–d** Method rankings based on average performance across datasets for the detection of (**b**) cell-type-specific biomarkers, (**c**) differentially accessible regions (DARs), and (**d**) enriched transcription factor motifs. These rankings highlight the relative effectiveness of each method in preserving biologically meaningful features. **e** Box plot comparing overall biological conservation scores across methods. The conservation score is computed as the average detection consistency of cell-type-specific biomarkers, DARs, and enriched transcription factor motifs. Box plots (**b**–**e**): centre line, median; box bounds, 25th--75th percentiles; whiskers, minimum and maximum values. Triangles represent individual dataset scores (*n* = 4 datasets per method). Narrower boxes and shorter whiskers indicate greater robustness and consistency across datasets. Source data are provided as a Source Data file.

We first examine biomarker detection accuracy using our BM score approach. To compare the consistency of biomarker identification across integration methods, we present heatmaps in Fig. 3a illustrating the JSI between biomarker sets identified by each method pair. We focused our analysis on NK cells from the *PBMC-10k* dataset and ENDO cells from the *Muto-2021* dataset as representative examples. For NK cells in the paired setting, we observe that most methods achieve relatively high median JSI values, indicating substantial agreement in biomarker identification across different integration approaches. This suggests that NK cell-specific gene expression signatures are robustly preserved by multiple integration methods, reflecting the distinct transcriptional identity of this cell type. In contrast, under unpaired conditions, none of the methods achieve a biomarker conservation capability above 0.8, highlighting the increased challenge of consistent biomarker identification when cells are not directly matched across modalities. Among the methods evaluated, GLUE demonstrates particularly strong performance for NK cells, while PCA shows superior results for ENDO cells. Notably, Harmony exhibits top median JSI for both NK and ENDO cells, suggesting it maintains more consistent biomarker identification capabilities across these two distinct settings. Meanwhile, methods with moderate JSI, such as DeepMAPS and TotalVI, may offer complementary value for exploring novel or cell-type-specific biomarkers, as they potentially capture unique aspects of the data and uncover alternative biomarkers not commonly detected by other methods. To substantiate this observation, we compare candidate marker genes identified for NK cells by DeepMAPS (moderate JSI) and GLUE (highest JSI). Notably, DeepMAPS uniquely captures SLAMF7 and KLRG1, which are absent from GLUE’s results. SLAMF7 is a well-established NK-specific surface receptor involved in cytotoxicity and immune activation^55^, while KLRG1 is a key inhibitory receptor highly expressed on mature NK cells and associated with terminal differentiation^56^. These biologically relevant markers, missed by the highest-consensus method but captured by a moderate-JSI approach, demonstrate that different methods can offer complementary insights into the biomarker landscape, with moderate-JSI methods potentially identifying functionally significant markers beyond the consensus set. To assess biomarker detection against established knowledge, we evaluate each method’s ability to recover experimentally validated cell-type markers from CellMarker 2.0 database for the *PBMC-10k* dataset (Supplementary Fig. 4). Several methods, including scGPT-scVI, scMoMaT, and scJoint, show strong recall of known biomarkers alongside high consistency scores, indicating their effectiveness in identifying biologically meaningful cell markers beyond statistical associations. When comparing FM and DM approaches in biomarker detection, we find that FMs demonstrate promising performance in this task. Notably, the zero-shot version of scGPT outperforms many DMs in identifying NK cell biomarkers, demonstrating the strong biological knowledge encoded in pre-trained language models. Interestingly, scGPT’s fine-tuned version shows relatively lower performance compared to its zero-shot counterpart, suggesting that introducing information from another omics modality may compromise some of the intrinsic biological knowledge specific to gene expression patterns during joint representation learning.

Figure 3b presents a comprehensive evaluation of biomarker detection performance at the transcriptomic level, incorporating results from both paired and unpaired settings across human and mouse datasets. For this comparison, we exclude FMs that could not be applied to the mouse data. FM-scVI denotes scGPT-scVI when applied to human data and UCE-scVI when applied to mouse data, selected based on their respective performance advantages. The ranking reveals that UnionCom, bindSC, and MOFA consistently achieve top performance with relatively stable scores across different conditions. GLUE, Harmony, and scJoint also demonstrate strong results, though with higher variability across datasets and settings. Despite UCE’s cross-species capabilities, it achieves lower BM scores in zero-shot mode. Our adaptation experiments show promise, with scGPT-scVI demonstrating competitive performance for NK cells in human data. However, there remains a considerable gap between FM approaches and top-performing DMs, particularly in unpaired scenarios and mouse data. This gap highlights promising directions for future research in adapting FMs for multi-omics integration across diverse experimental conditions and species.

Beyond evaluating transcriptomic-level markers, we extend our assessment to the epigenomic dimension by analyzing chromatin accessibility patterns in scATAC-seq data. This extension includes two modality-specific features: DARs identified using similar statistical approaches as DE genes, and enriched transcription factor binding motifs within the top 100 DARs determined through GimmeMotifs analysis. These evaluations are conducted across all datasets, including paired, unpaired human, and mouse samples, with results displayed in Fig. 3c, d, while detailed dataset-specific results are provided in Supplementary Fig. 5. In the epigenomic assessment, Seurat4 achieves the highest scores for both DARs and enriched motifs detection, demonstrating its exceptional capability in capturing chromatin accessibility patterns and regulatory elements. scJoint, LIGER, and Harmony follow closely behind, indicating their strong performance in preserving epigenomic features during integration.

Figure 3e presents the overall biomarker detection performance ranking across all three metrics. Consistently, bindSC, Seurat4, PCA, and scJoint demonstrate superior performance, establishing themselves as effective methods for multi-omics biomarker discovery. Despite moderate transcriptomic performance, UCE achieves results comparable to several DMs, while at the scATAC-seq level, FM-scVI shows comparable effectiveness to established methods like Harmony and GLUE. These findings suggest that while specialized integration methods currently lead in biomarker detection, FMs demonstrate promising capabilities that could be enhanced through targeted improvements to preserve both transcriptomic and epigenomic signals.

### Assessing trajectory alignment for enhanced biological dynamics

Assessing how well integrated representations preserve essential biological features and dynamics is crucial^57,58^. An effective integration method should maintain trajectory information from the original data. We evaluate this by measuring trajectory alignment between integrated and unintegrated data, then quantify preservation using our Trajectory Conservation (TC) score as defined in the Methods. This approach prioritizes biological relevance over technical metrics^37,59^. We evaluate the TC capability of integration methods using TC scores across both paired and unpaired settings, as well as multiple biological hierarchies.

**Fig. 4:**
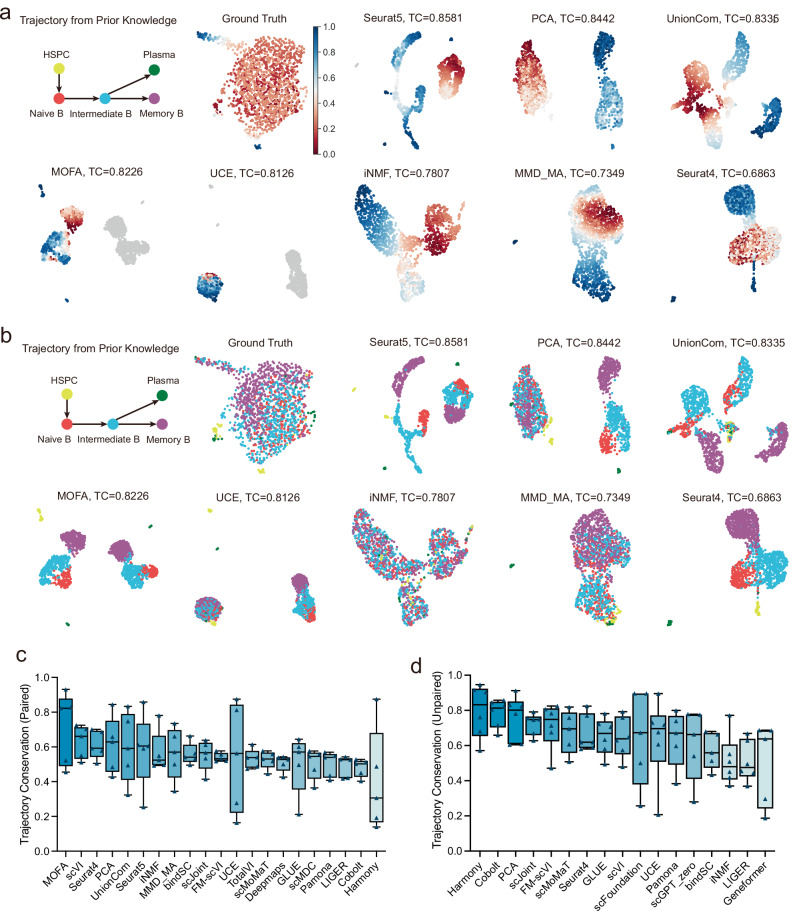
Comparison of biological dynamics preservation performance of DMs and FMs through trajectory alignment. **a**, **b** PAGA representation of the ground-truth lineage, and UMAP visualizations of pseudotime inference and cell type annotations, derived from the integrated embeddings of the top eight methods on *PBMC-10k* (out of 19 DMs and four FMs). The analysis focuses on B cell development. All subplots are arranged in descending order based on the TC score. **c**, **d** Box plot comparison of trajectory conservation for (**c**) paired datasets and (**d**) unpaired datasets, where the depth of color corresponds to the performance level. Centre line, median; box bounds, 25–75th percentiles; whiskers, minimum and maximum values. Triangles represent individual scores (*n* = 5 in (**c**); *n* = 6 in (**d**)). For overall metrics, FM-scVI represents the best-performing foundation model combined with scVI on each dataset. Source data are provided as a Source Data file.

For the *PBMC-10k* dataset, we compute TC scores under three scenarios: all cells, bidirectional development, and unidirectional development. MOFA achieves the highest performance with a TC score of 0.9307 when considering all cells. Additionally, we analyze TC scores across major and minor cell type groups to investigate whether cell population abundance affects trajectory inference performance. Interestingly, methods fall into two groups in Supplementary Fig. 6: those performing better on major cell types and those excelling with minor populations, suggesting that cell type abundance is not the primary determinant of TC capability. For detailed trajectory analysis, we examine immune cell differentiation pathways with HSPC as the root cell type, expecting ideally to observe T cell subtypes (CD8 TEM_1/2, CD4/CD8 Naive, CD4 TCM/TEM) and B cell lineages (Naive, Intermediate, Memory B) distributed at opposite ends of the bidirectional development trajectory. In Supplementary Fig. 7, we observe that the pseudo-time inference result of MOFA has a clear color change trend similar to the ground truth, with HSPC around dark red and B cells around blue. Within the groups, naive T cells are found closer to the HSPC cluster. scVI, Seurat4, bindSC, GLUE, and DeepMAPS also preserve the trajectory and align well with biological interpretation. The color and size of each group are relatively consistent between the ground truth and DeepMAPS, likely due to its multi-head graph transformer architecture, which effectively models biological networks. We then examine a unidirectional, well-characterized B cell developmental lineage (HSPCs  → Naive B  → Intermediate B  → Memory B  → Plasma cells). Figure 4a shows the UMAP visualizations of pseudotime inference results from the top eight performing methods, alongside cell type annotations. Among these, Seurat5, PCA, UnionCom, MOFA, and UCE achieve the highest TC scores. For reference, the canonical trajectory derived from prior biological knowledge is also shown. Notably, the embedding generated by Seurat5 clearly captures the developmental progression from Naive B to Memory B cells. Overall comparison of all paired scenarios can be found in Fig. 4c. We also evaluate TC on the *Ma-2020* dataset focused on neuronal differentiation in the mouse cortex, specifically capturing the progression from intermediate progenitor populations (IP-Hmgn2, IP-Gadd45g, IP-Eomes) to upper-layer excitatory neurons (Ex-L2/3-Cntn2 and Ex-L2/3-Cux1) (Supplementary Fig. 8). Notably, the developmental trajectory is more apparent in the RNA modality, reflecting the stronger transcriptional signal of differentiation. Most methods yield lower TC scores of below 0.7 in this complex developmental system, with the exception of UCE, which maintains superior performance across both datasets. We further evaluate TC in the unpaired data scenario using all cells across multiple datasets. As shown in Fig. 4d and Supplementary Fig. 14f, Cobolt and Harmony demonstrate superior TC performance with statistically significant results compared to other methods. PCA and scJoint also show notable effectiveness in preserving trajectory information in the unpaired setting, highlighting that simpler methods can sometimes maintain important biological relationships even without complete data alignment.

It is noteworthy that scGPT preserves trajectory information better than many DMs, though its performance gap between zero-shot and fine-tuned modes is considerably larger for TC than for integration accuracy. When evaluating the preservation of trajectory information in zero-shot mode using RNA embedding alone versus joint embedding (see Supplementary Data 3), we find that FMs generally outperform DMs by a substantial margin in the RNA-only scenario. This indicates that FMs effectively retain dynamic signals related to biological processes by leveraging their pre-trained knowledge of scRNA-seq. However, introducing ATAC-seq data significantly deteriorates the performance of FMs in zero-shot mode, indicating that they require proper adaptation to effectively handle multi-omics data integration. UCE shows moderate performance on all cells and pivotal cell populations, but excels in simpler unidirectional developmental branches. Particularly in the mouse cortex neuronal differentiation dataset, UCE is the only method achieving a TC score above 0.7, highlighting its strength as a cross-species FM in capturing conserved developmental trajectories across different organisms. When examining unpaired settings (Fig. 4d), our adapter-based integration approach (FM-scVI) performs comparably to scJoint, further demonstrating that FMs can be effectively adapted to multi-modal integration tasks through appropriate architectural modifications and training strategies, even in challenging unpaired scenarios.

Our comprehensive assessment of TC across diverse settings reveals that MOFA excels in paired integration scenarios while Cobolt performs best in unpaired settings. Harmony demonstrates variable performance dependent on dataset characteristics. Among FMs, UCE shows particular strength with mouse data and developmental trajectories. While FMs exhibit promising biological signal preservation capabilities, they typically require appropriate adaptation for multi-modal integration. These findings emphasize the importance of method selection based on specific experimental contexts and research objectives.

### Thorough and quantitative evaluation of batch effect correction

We conduct a comprehensive benchmark to evaluate the batch effect correction capabilities of various multi-omics integration methods on both real and simulated datasets. To address scalability challenges observed with methods like scMoMaT, MMD-MA, UnionCom, and Pamona on large datasets, we downsize each batch of *Ma-2020* and *Muto-2021* datasets to 2500 cells to evaluate batch correction capabilities. In evaluating real datasets, we assess batch effect correction performance using both paired and unpaired datasets. For paired datasets, as shown in Fig. 2a, bindSC and LIGER achieve the highest scores in batch correction, though their performance in integration accuracy is sub-optimal. In contrast, Seurat4, scJoint, and GLUE demonstrate a balanced performance between integration accuracy and batch effect correction. For unpaired datasets, we observe consistent patterns that bindSC, scJoint, and LIGER excel in batch effect correction, while Seurat4 and GLUE perform strongly across both batch effect correction and integration accuracy metrics.

**Fig. 5:**
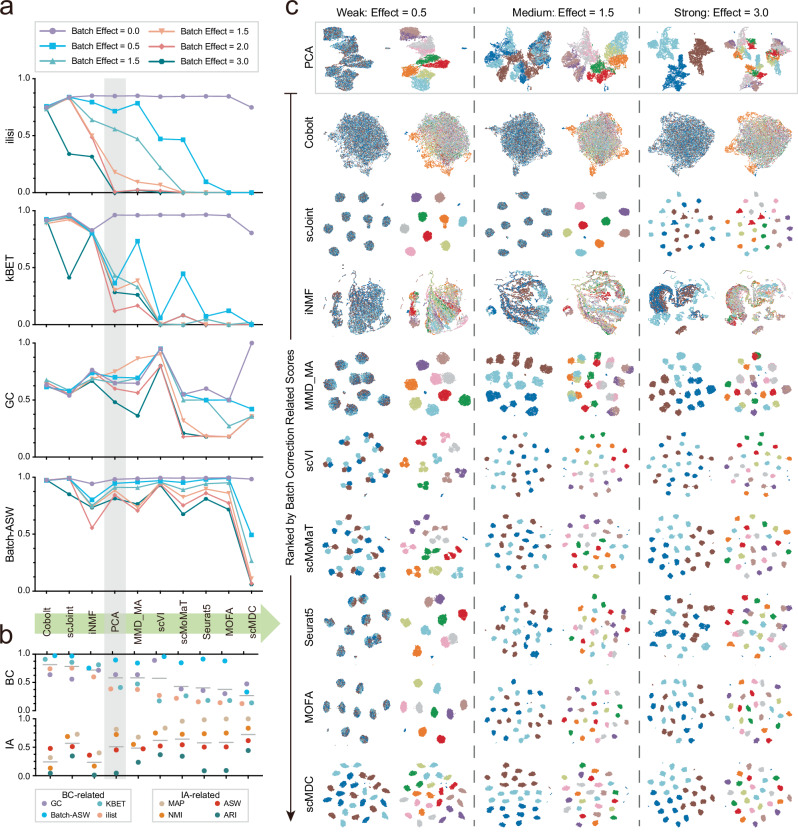
Comprehensive comparison on simulated datasets with quantitative batch effects. **a** Line graphs of the performance comparison of 10 multiomics integration methods on six simulated datasets with batch effects. Methods are arranged according to their averaged batch correction-related scores (from highest to lowest, left to right). **b** The comparison of batch correction-related scores and the integration accuracy-related scores. Each dot represents one metric averaged across six simulated batch effect levels. The gray horizontal line indicates the mean across all metrics for each method. The green arrow shows the descending method ranking in terms of their overall batch correction-related score. We find that methods with strong batch correction capabilities do not necessarily exhibit good integration accuracy, while scJoint demonstrated impressive performance across both tasks. **c** UMAP visualization of ten multiomics integration methods on three simulated datasets with different levels of batch effects. The left subfigure is colored by batch labels, and the right subfigure is colored by cell types. Source data are provided as a Source Data file. Created in BioRender. Group, A. (2026) https://BioRender.com/fu6hida.

Quantifying batch effects in real-world datasets is challenging. Therefore, benchmarking batch effect correction performance with varying levels of simulated batch effects allows for a more comprehensive and quantitative evaluation. It is important to note that current multi-omics data simulation methods cannot simulate real gene names and peak locations, rendering methods dependent on gene networks and scATAC peak region sequences inapplicable to simulated data. We compare the performance of ten multi-omics integration methods across six simulated datasets with varying batch effects generated using scMultiSim^60^. Figure 5a presents the results as line graphs, with methods arranged in descending order based on their average batch correction-related scores. Figure 5b and Supplementary Figs. 9, 10 visualize eight quantitative metrics related to integration accuracy and batch effect correction. Analysis of batch correction-related scores alongside integration accuracy-related scores reveals a fundamental trade-off in multi-omics integration^12,61^. First, the mathematical objectives of these two goals are inherently competing. Methods designed for effective batch effect correction typically employ aggressive normalization or alignment strategies that minimize technical variation across batches, but these same strategies can inadvertently remove biologically relevant signals. Conversely, approaches prioritizing the preservation of biological structures often retain unwanted technical artifacts. This intrinsic tension stems from the algorithmic challenge of distinguishing technical noise from true biological variation, particularly challenging when batch effects correlate with biological features—a common scenario in multi-omics studies where different platforms or protocols may preferentially capture distinct cellular properties. For instance, methods like iNMF and Cobolt demonstrate superior batch effect removal but fail to generate cell-type-specific embeddings, resulting in low biological conservation metrics. This suggests their algorithms may overcompensate for batch effects, inadvertently removing biologically meaningful variations. The principal components extracted by these methods likely capture technical variation at the expense of the biological signal. Conversely, scMDC achieves the highest performance in cell type identification metrics but is significantly impacted by batch effects, indicating it prioritizes preserving biological heterogeneity over removing technical noise. Notably, scJoint and MMD-MA emerge as standout methods, demonstrating impressive results in both batch correction and integration accuracy, highlighting their effectiveness in addressing the batch correction challenges of multi-omics data integration. Figure 5c displays UMAP low-dimensional representations of latent embeddings generated by the ten methods under three different levels of batch effects. More detailed visualizations for six different levels of batch effects are shown in Supplementary Figs. 11–13. When batch effects are weak, all methods except Cobolt and iNMF are robust and clearly separate different cell types. As batch effect size increases, all methods are affected, with cell embeddings becoming increasingly separated by batch identity. Interestingly, PCA demonstrates greater robustness to medium batch effects compared to more advanced methods like scMDC. scJoint remains the most robust, with cell embeddings remaining well-integrated even at medium batch effect sizes. Under strong batch effects, almost all methods are severely affected, while scVI and scJoint still generate highly cell-type-specific embeddings.

### Foundation models: capabilities, limitations, and adaptation for multi-omics integration

FMs have emerged as valuable tools in single-cell research^28–31^. They naturally align with diverse tasks and demonstrate robust generalization capabilities. While scGPT stands out as a dedicated FM explicitly designed for paired multi-omics integration, it is noteworthy that all FMs can be adapted by examining the extracted cell embeddings. However, it is important to note that current single-cell foundation models are predominantly trained on scRNA-seq data, and their application to scATAC-derived inputs represents a non-standard usage scenario that may introduce out-of-distribution effects. We include this setup to simulate realistic use cases where non-specialist users may attempt to leverage FMs across modalities. For scATAC-seq data, we evaluate three preprocessing strategies: (i) calculating biologically meaningful gene activity scores from chromatin accessibility data using the regulatory potential model implemented in MAESTRO^44^, which captures the relationship between chromatin accessibility and gene regulation; (ii) deriving gene activities using Seurat4^21^, which provides a commonly used alternative implementation; and (iii) translating scATAC profiles into scRNA-like representations using BABEL^45^, a cross-modal translation method. Validation analyses of the activity matrices derived from these strategies confirm preservation of cell-type-specific signals and biological clustering structure (Supplementary Figs. 1, 16). Comparison of FM performance using MAESTRO versus BABEL preprocessing reveals dataset-dependent effects, with neither strategy consistently outperforming the other across all metrics (Supplementary Fig. 17). These preprocessing strategies enable FMs to process multi-omics data while preserving biological interpretability. Compared to DMs, FMs excel at handling datasets of varying sizes, including large-scale datasets at the atlas level. Their advanced architecture is designed to efficiently process and analyze massive amounts of data, making them particularly well-suited for applications that demand scalability and computational efficiency.

Our analysis reveals that FMs generally underperform compared to state-of-the-art DMs in single-cell multi-omics data alignment and integration tasks. Nevertheless, FMs demonstrate unique strengths in specific biological contexts. In biomarker detection, scGPT’s zero-shot mode shows surprisingly strong results for NK cells, suggesting effective pre-trained representation of transcriptomic signatures. For trajectory conservation, UCE uniquely excels in mouse cortical neuronal differentiation, achieving TC scores above 0.7 where other methods struggle. These findings indicate that FMs possess valuable biological knowledge that can effectively preserve certain cell-type-specific signals and developmental trajectories, particularly when analyzing data similar to their training distributions.

Despite these strengths, FMs face significant limitations in multi-omics integration. Most models are predominantly trained on human data, leading to cross-species bias when generalizing to other organisms. Even UCE, which attempts to address this by training on diverse tissues and species, suffers from data imbalance issues in its training atlas. More critically, FMs show particular vulnerability when integrating across modalities, with performance deteriorating significantly when introducing scATAC-seq data in zero-shot settings. This suggests fundamental limitations in their ability to bridge different omics layers without additional training or architectural modifications. Computational demands present another challenge, with models like scGPT requiring substantial resources for fine-tuning approaches.

**Fig. 6:**
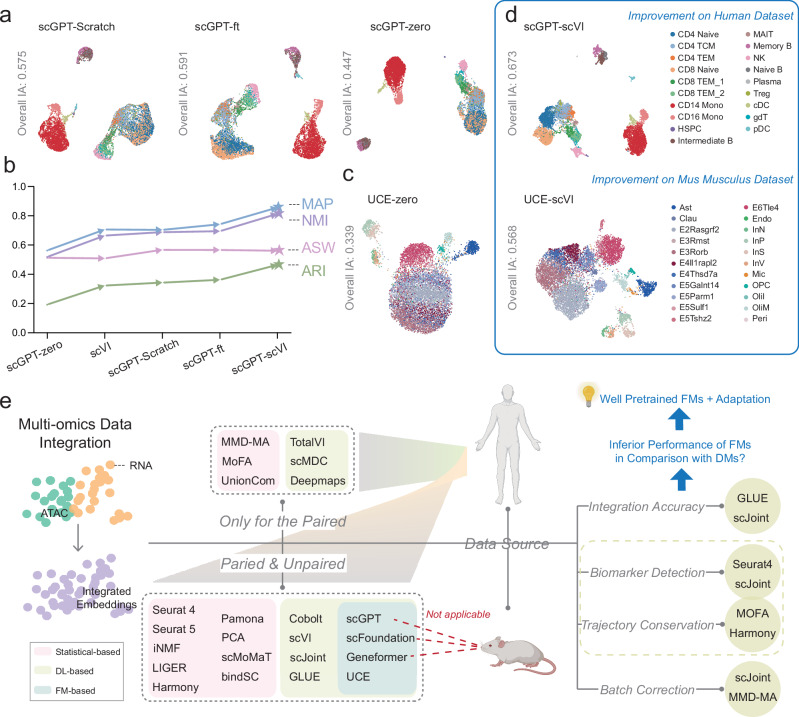
In-depth analysis of the FMs with adaptation strategies and recommendation pipeline of SCMBench. **a** UMAP visualization of scGPT-Scratch (fine-tuned only with pre-trained gene token embeddings), scGPT-ft (fine-tuned with the first eight transformer modules), and scGPT-zero (without fine-tuning) for human data. The overall integration accuracy of the obtained embeddings is annotated. **b** Evaluation metrics (MAP, NMI, ASW, and ARI) of different strategies of scGPT on *PBMC-10k*. Source data are provided as a Source Data file. **c** UMAP visualization of UCE-zero without fine-tuning for mouse data. **d** UMAP of the FMs-scVI method, which concatenates an adaptation layer to FMs. The adapter method effectively improves the performance of both human and mouse datasets. **e** Software recommendation pipeline. For multi-omics data integration in this study, existing approaches can be categorized into two groups based on their ability to handle unpaired datasets. Methods are color-coded by category as indicated in the figure legend. It is worth noting that the majority of FMs, trained primarily on human data, exhibit limited scalability to other species, such as *Mus musculus*. Methods unsuitable for mouse data are appropriately labeled.

To effectively leverage FMs in this field and explore their potential across various application scenarios, we systematically evaluate multiple adaptation strategies, including adapter-based integration of FM embeddings, fine-tuning, and parameter-efficient tuning approaches. Among these strategies, adapter-based integration employs scVI as a lightweight adaptation module that transforms FM embeddings into more effective representations for single-cell multi-omics integration. To explore the potential of FMs in multi-omics integration tasks on human data, we compare the scGPT-scVI with zero-shot prediction and two fine-tuning approaches on scGPT: scGPT-Scratch, utilizing only the pretrained gene embeddings module, and scGPT-ft, employing the first eight pretrained transformer modules, which is the best configuration identified in our fine-tuning experiments (Supplementary Table 4). Figure 6a and the top of Fig. 6d present UMAP visualizations of the representations, with corresponding integration accuracy scores annotated. Figure 6b illustrates detailed evaluation metrics of the different versions of scGPT, with scVI added for comparison. These results indicate the superior performance of scGPT with the adaptation layer, surpassing all other strategies and scVI. We further investigate whether this adaptation can improve performance on mouse datasets using UCE. An efficient fine-tuning layer is added to the pre-trained UCE model. As shown in Fig. 6c and the bottom of Fig. 6d, UCE-scVI increases the overall integration accuracy by over 65% compared to its zero-shot version. This approach bridges the gap between FMs’ biological knowledge and multi-omics integration requirements while maintaining computational efficiency.

In conclusion, FMs show promise for multi-omics integration through their ability to capture complex biological relationships, but require appropriate adaptation strategies to overcome modality-specific and cross-species limitations. Our adaptation approach demonstrates a viable path forward, enabling these models to better preserve both technical accuracy and biological significance in integrated representations. This hybrid strategy offers a promising direction for leveraging the complementary strengths of FMs and specialized integration algorithms in single-cell multi-omics analysis.

### Tailored method selection guide for multi-omics integration scenarios

Based on our comprehensive benchmarking, we offer the following practical recommendations for researchers selecting integration methods: for integration accuracy, GLUE, scMDC, scJoint, and Seurat4 emerge as top methods. Seurat4 demonstrates particularly strong performance in unpaired settings, while scJoint stands out as the premier choice for unpaired integration with superior performance and moderate computational requirements. For cross-species analyses, UCE performs best among FMs, though adapter-based integration (UCE-scVI) offers improved performance while maintaining cross-species capabilities. For biomarker detection, Seurat4 and scJoint demonstrate superior overall performance. Seurat4 particularly excels in chromatin accessibility analysis, while GLUE and Harmony better preserve transcriptomic-level markers in specific cell types. Methods with moderate JSI may identify unique biomarkers missed by other approaches, offering complementary biological insights. For trajectory conservation, MOFA performs best in paired settings, while Harmony leads in unpaired scenarios. For specific developmental branches, Seurat5 shows strong performance in B cell trajectories, and UCE demonstrates unique strength in mouse developmental pathways, highlighting its cross-species capabilities in preserving evolutionary conserved developmental programs. For batch effect correction, scJoint and MMD-MA stand out by achieving impressive results in both batch correction and integration accuracy, effectively addressing the inherent trade-off between these objectives in multi-omics data integration. When computational resources are limited, scJoint, bindSC, and Harmony offer good performance-to-efficiency ratios across different analysis tasks, making them versatile options for various research contexts.

We provide a detailed recommendation pipeline that integrates the strengths of statistical-based methods and deep learning-based methods, considering their capabilities in handling unpaired datasets and scalability across different species (see Fig. 6e). The top two recommended methods are annotated for each evaluation aspect. Given that FMs typically do not perform as well as specialized deep learning methods, we systematically evaluate multiple adaptation strategies, including adapter-based integration, fine-tuning, and parameter-efficient tuning. These evaluations provide practical guidance for enhancing FM performance and inform future model design. We have made all benchmark code, evaluation metrics, and our adapter-based integration implementation publicly available to facilitate method selection and future benchmarking efforts. All detailed evaluation results are provided as Supplementary Data with clear documentation for comprehensive reference. As the field continues to evolve, we encourage researchers to consider these comparative results alongside their specific biological questions and technical constraints.

## Discussion

With the rapid advancement of techniques for integrating single-cell multi-omics data, thorough evaluations are crucial for selecting appropriate methods in practical experiments. Previous studies, such as Lee et al.^32^, benchmark nine integration methods but overlook the latest deep learning-based approaches and lack comprehensive downstream task analyses. More recently, Xiao et al.^33^ expand this scope to twelve multi-omics integration methods and include trajectory inference tasks. However, their study does not fully explore critical tasks such as biomarker detection and batch effect correction. In our study, we evaluate 19 DMs and four FMs across a diverse collection of both real and simulated multi-omics datasets. We analyze not only integration accuracy but also the maintenance of cellular features, biological processes, and regulatory mechanisms through comprehensive downstream analyses. Our evaluation framework consists of 13 metrics spanning integration accuracy, biomarker detection, trajectory inference, and batch effect correction. Previous methods have primarily focused on datasets comprising no more than three components. However, statistical analysis cannot be performed on a single data point, especially when evaluating the performance separately for paired and unpaired scenarios. In this study, we consider a total of six datasets, with two containing different batches that we partition for additional evaluation beyond batch correction, and one *triple-omics* dataset from the mouse cortex. For a detailed comparison between our study and existing works, please refer to Supplementary Table 3.

Our comprehensive evaluation reveals important trade-offs in method performance across different tasks. The varying effectiveness of integration methods across trajectory conservation assessments reflects the inherent challenges in preserving complex biological signals during multi-omics integration. We observe a clear division between methods optimized for paired versus unpaired settings, with MOFA and Cobolt emerging as recommended choices, respectively. The inconsistent performance of Harmony across datasets highlights the sensitivity of some methods to specific data characteristics, including species origin and experimental design. Most current methods focus primarily on technical integration accuracy rather than biological signal preservation. Our biomarker detection and trajectory conservation evaluations highlight this issue, with methods excelling at cell-type clustering often underperforming in preserving developmental trajectories or regulatory relationships. This finding underscores the need for integration methods that balance technical accuracy with biological relevance, particularly as integration results increasingly inform downstream functional and mechanistic analyses.

While FMs have demonstrated transformative potential in other fields, their capabilities in single-cell biology remain underexplored. Our benchmark results, alongside published literature, suggest that current single-cell FMs have not yet been adequately harnessed for this domain. Despite lower overall integration accuracy compared to specialized DMs, FMs demonstrate unique strengths in versatility and efficiency. Their versatility enables adaptation across diverse single-cell tasks with the same architecture, while their efficiency advantages include both reduced training requirements and computational feasibility on large-scale datasets where many specialized DMs become computationally prohibitive or fail to complete. However, their struggle with multi-modal integration in the zero-shot setting underscores the necessity for appropriate adaptation strategies when handling different data modalities. Most FMs are predominantly trained on human data, leading to cross-species bias when generalizing to other organisms. Even UCE, which attempts to address this by training on diverse tissues and species, suffers from data imbalance issues in its training atlas. The exceptional performance of UCE on mouse developmental data suggests that species-specific pre-training may confer advantages for certain biological contexts. To address these limitations while leveraging FMs’ rich biological knowledge, we systematically evaluate multiple adaptation strategies. Among these, adapter-based integration combines FM embeddings with scVI’s variational autoencoder framework. We implement these adaptations as standalone methods and incorporate them into our comprehensive benchmarking pipeline, evaluating them alongside traditional deep learning methods across all metrics and datasets. Our results demonstrate that this strategy effectively bridges the gap between FMs’ biological knowledge and the specific requirements of multi-omics integration tasks, showing particular improvements in biomarker detection and trajectory conservation while maintaining computational efficiency. However, further refinement and validation are necessary to fully realize its potential.

Despite our comprehensive benchmarking efforts, several limitations should be acknowledged. First, our evaluation focuses primarily on scRNA-seq and scATAC-seq integration, while other important multi-omics combinations (such as protein, spatial, or metabolomics data) remain to be explored. We also conduct triple-omics integration experiments, including single-cell DNA methylation data, which show that only seven methods are capable of handling triple-omics integration, yet all methods exhibit decreased performance compared to dual-omics results (Supplementary Data 2). This highlights the technical challenges in integrating three or more modalities simultaneously. As multi-omics technologies continue to advance, more sophisticated computational approaches are needed to effectively handle complex multi-modal data integration. Second, the field of FMs is rapidly evolving, with new pre-trained models continually emerging; our assessment represents a snapshot of currently popular models rather than an exhaustive evaluation. Due to the limited number of FMs designed to handle multi-omics integration tasks, comparing their zero-shot performance with specialized DMs may not be entirely fair. We include adapter-based integration strategies to partially address this limitation, though this represents just one possible direction for leveraging FMs in this domain and is not the primary focus of this work. Additionally, while scATAC-specific FMs such as GET^62^ have recently emerged, they currently operate at the cell-type level rather than providing cell-level representations, limiting their applicability for single-cell integration benchmarking. Substantial further research will be needed to fully realize the potential of FMs in multi-omics integration. Third, computational scalability for atlas-level integration remains a critical challenge, and our benchmarking platform itself imposes certain limitations on comprehensive evaluation across all methods for the largest datasets. Despite using a high-performance computing setup with multi-core CPUs, 128GB RAM, and NVIDIA RTX 3090 GPUs, several methods still encounter resource limitations. While several methods (GLUE, LIGER, Seurat4, scVI, iNMF, Cobolt, and scJoint) successfully process our largest unpaired dataset (*Yao-2021* with 69,727 scRNA-seq cells and 54,844 snATAC-seq cells), others encounter significant barriers. We observe out-of-memory errors with scMoMaT, MMD-MA, and UnionCom on moderately sized datasets like *Ma-2020* (32,231 cells), while Pamona’s runtime exceeds 24 h. These platform-imposed constraints highlight the urgent need for more efficient algorithms capable of handling the increasingly massive datasets generated by contemporary single-cell technologies, as computational limitations remain a significant practical barrier to method adoption. Fourth, expanding the benchmark with additional datasets would further enhance the generalizability of our findings. Importantly, our results highlight that method performance is often case-specific, and we find it challenging to identify any single method that consistently outperforms others across all metrics, datasets, and analysis tasks. This context-dependence is further evidenced by our statistical analyses using the Friedman test followed by Conover post-hoc comparisons (Supplementary Fig. 14). With few exceptions, we observe widespread statistical parity among methods. While our Friedman test indicates significant overall differences among methods, post-hoc pairwise comparisons via critical difference diagrams show that most methods fall within statistically indistinguishable groups (*p* > 0.05), which is notable considering the breadth of algorithmic designs represented. This statistical equivalence suggests we may be approaching optimization plateaus with current integration paradigms, and indicates that researchers have multiple viable options when selecting methods, allowing secondary considerations such as computational efficiency to guide decision-making without significantly compromising performance. As the field continues to generate more multi-omics datasets, developing truly adaptable, universally effective integration methods that perform consistently across varied biological contexts remains a fundamental challenge and important direction for future research.

Our comprehensive benchmarking of DMs and FMs provides practical guidance for method selection and highlights directions for improving multi-omics integration. Looking forward, single-cell multi-omics integration represents a critical step toward building AI Virtual Cells, computational models that simulate cellular behavior by unifying molecular measurements across modalities^63^. This benchmarking framework and adaptation strategies provide a foundation for evaluating and improving the integration modules essential to such virtual cell models.

## Methods

### Datasets and preprocessing

We select three paired datasets (*PBMC-10k*^46^, *Chen-2019*^47^, *Ma-2020*^48^), two unpaired datasets (*Muto-2021*^8^, *Yao-2021*^49^), and one *triple-omics* dataset^5,50^, considering factors such as species and organ diversity, sample size, batch effects, and sequencing techniques, to comprehensively evaluate various methods across a wide range of application scenarios (see Fig. 1b and Supplementary Table 1). To examine results under varying conditions, we also generate simulated paired data with progressively increasing batch effect sizes to assess the performance limits of the methods.

#### Real data preprocessing

To minimize the excessive influence of batch effects on the exploration of integration accuracy and biomarker detection, we partition the *Ma-2020* and *Muto-2021* datasets, which inherently contain batch effects. The *Ma-2020* dataset is divided into three batches containing 5692, 10,700, and 9903 cells, respectively, while the *Muto-2021* dataset is split into five batches with 3683, 5464, 3804, 4114, and 2920 cells. To ensure that a wide range of methods could successfully operate across different datasets, we sample the larger *Ma-2020* and *Muto-2021* datasets to maintain 2500 cells per batch. This resulted in the combined sampled datasets, *Ma-2020-sampled* and *Muto-2021-sampled*, which are used for experiments investigating batch effects. The original *Ma-2020* and *Muto-2021* datasets are employed to test batch effects under imbalanced conditions. Subsequently, we utilize Scanpy, with flavor="seurat_v3”, to uniformly filter the highly variable features of the data, retaining 80,000 peaks for scATAC-seq and 8000 genes for scRNA-seq. To ensure fairness among methods requiring an activity matrix, we standardize the calculation using the ATACCalculateGenescore from the MAESTRO library^44^, following the implementation approach of DeepMAPS^43^, while additionally evaluating alternative activity derivation strategies, including the GeneActivity module in Seurat4^21^ and the cross-modal translation outputs from BABEL^45^, for completeness and consistency across methods.

#### Simulated data generation

We use scMultiSim^60^ to generate simulated datasets for comprehensively evaluating existing multiomics integration methods on batch effect correction and cell type clustering. The simulated dataset contains 10,000 cells with paired scRNA-seq and scATAC-seq datasets. We set the simulated batch number to 3 while varying the batch effect size across [0, 0.5, 1, 1.5, 2, 3] for comprehensive evaluation from weak to strong batch effects. The number of simulated genes and peaks is set to 3000.

### Evaluation metrics

#### Integration accuracy

- Mean average precision (MAP) is used to evaluate the cell type resolution. Supposing that the cell type of the ith cell is y(i) and that the cell types of its *K* ordered nearest neighbors are ${y}_{1}^{(i)}$,${y}_{2}^{(i)}$,${y}_{K}^{(i)}$, the mean average precision is then defined as follows:
  1 $$\begin{array}{c}{{{\rm{MAP}}}}=\frac{1}{N}{\sum }_{i=1}^{N}{{{{\rm{AP}}}}}^{(i)}\\ {{{{\rm{AP}}}}}^{(i)}=\left\{\begin{array}{ll}\frac{{\sum }_{k=1}^{K}{1}_{{y}^{(i)}={y}_{k}}\cdot \frac{{{\sum }_{j=1}^{k}}^{1}(i)={y}_{j}^{(i)}}{k}}{{\sum }_{k=1}^{K}{1}_{{y}^{(i)}={y}_{k}^{(i)}}},& {{{\rm{if}}}}\,{\sum }_{k=1}^{K}{1}_{{y}^{(i)}={y}_{k}^{(i)}} > 0\\ 0,\hfill & {{{\rm{otherwise}}}}\end{array}\right.\end{array}$$
  where ${1}_{{y}^{(i)}={y}_{k}^{(i)}}$ is an indicator function that equals 1 if ${y}^{(i)}={y}_{k}^{(i)}$ and 0 otherwise. For each cell, average precision (AP) computes the average cell type precision up to each cell type-matched neighbor, and MAP is the average AP across all cells. Mean average precision has a range of 0 to 1, and higher values indicate better cell type resolution
- Normalized mutual Information (NMI) is a metric used to assess the similarity between two clusterings. In our study, we employ NMI to compare the cell-type labels with the Louvain clusters derived from the combined latent embedding from different omics. To scale the overlap, we calculate the average of the entropy terms associated with the cell type and cluster labels. NMI scores of 0 or 1 indicate no correlation or a perfect match between the clusterings, respectively. The scikit-learn library^64^ (version 0.22.1) is utilized for the NMI implementation.
- Average Silhouette Width (ASW) is used to evaluate the cell type resolution, defined as
  2 $${{{\rm{ASW}}}}=\frac{1}{2}\left(\frac{{\sum }_{i=1}^{N}{s}_{{{{\rm{cell}}}}\,{{{\rm{type}}}}}^{(i)}}{N}+1\right)$$
  Where ${s}_{{{{\rm{cell}}}}\,{{{\rm{type}}}}}^{(i)}$ is the cell type silhouette width of the *i*th cell. The obtained value is between 0 and 1, and the higher the value, the better the cell type resolution.
- Adjusted Rand Index (ARI) compares the overlap of two clusterings; it considers both correct clustering overlaps while also counting correct disagreements between two clusterings. Similar to NMI, we compare the cell-type labels with the NMI-optimized Louvain clustering computed on the combined latent embedding from different omics. The adjustment of the Rand index corrects for randomly correct labels. An ARI of 0 or 1 corresponds to random labeling or a perfect match, respectively. We also use the scikit-learn^64^ (v.0.22.1) implementation of the ARI.

#### Bio conservation

- Trajectory Conservation (TC) We calculate the trajectory conservation score using Spearman’s rank correlation coefficient^12^. Assuming that the trajectories in the unintegrated data (RNA-seq) represent the correct inference, we compute the Spearman’s rank correlation coefficient between the trajectories inferred before and after integration. A higher score indicates that the trajectory after integration closely aligns with the trajectory before integration. To infer the trajectory, we utilize diffusion pseudotime implemented in *scanpy.tl.dpt* with the assistance of scanpy. For the unintegrated data, the starting cell of the trajectory needs to be assigned manually based on the specific dataset. On the other hand, the starting cell for the integrated data is determined by selecting the most extreme cell from the cell-type cluster that contains the starting cells of the pre-integration diffusion pseudotime. It is important to note that only the largest connected component cells in the neighborhood graph are considered. The Spearman’s rank correlation coefficient is denoted as *s*, and the final trajectory conservation score is calculated as follows:
  3 $${{{\rm{trajectory}}}} \; {{{\rm{conservation}}}} \,=\frac{s+1}{2}$$
  The trajectory conservation score ranges from 0 to 1, where a higher score indicates a greater similarity between the order of cells in the integrated trajectory and the order of cells in the unintegrated data. However, in cases where the computation of diffusion pseudotime is not feasible due to the presence of multiple connected components in the KNN graphs of the integrated data, the score is set to 0.
- Biomarkers Detection Consistency (BM) To evaluate the preservation of cell-type-specific biological signals, we compute a biomarker detection score for each method as follows. First, cell types are annotated based on the embeddings produced by each method using a k-nearest neighbors (kNN) classifier. For each annotated cell type, we identify the top 100 differentially expressed genes (DEGs) as candidate markers. The DEGs are identified from scRNA-seq data using ‘rank_genes_groups’ function with the t-test of the Scanpy package. We then calculate the Jaccard similarity index (JSI) of marker gene sets between all pairs of methods, separately for each cell type. The biomarker detection score of a method is defined as the average JSI across all cell types and method pairs involving that method. This score reflects the consistency of cell-type-specific marker detection across different integration approaches.
- Biomarker Detection with Prior Knowledge To evaluate biomarker prediction against known references for the *PBMC-10k* dataset, we collect curated cell-type-specific markers from CellMarker 2.0 as ground truth^65^. For each method and cell type, we compute the recall by measuring the proportion of predicted candidate biomarkers that match the known markers. This metric reflects agreement with established knowledge, but does not account for potentially novel or unannotated biomarkers.
- Differential Accessible Regions Detection (DARs) This score is computed similarly to the biomarker detection score, except that the top 100 DARs are identified for each cell type from scATAC-seq data instead of marker genes. Jaccard similarity indices of DAR sets are calculated across all method pairs and averaged to quantify consistency.
- Enriched Motifs (Motifs) Building on the DARs identified in the previous step, we perform motif enrichment analysis using GimmeMotifs (with MEME, BioProspector, and Homer as default tools)^54^. Only known motifs curated in the GimmeMotifs database are considered. Surprisingly, the identified DARs are all upregulated, and the enriched motifs may be involved in gene regulation processes. We compute the consistency score by comparing enriched motifs across methods using Jaccard similarity indices, as done for biomarkers and DARs.
  - Jaccard Similarity Index (JSI) is a statistical measure used to assess the similarity and diversity between sample sets. It is particularly applied in our study for comparing sets of biomarkers identified by various integration methods. The JSI is defined as:
    4 $${{{\rm{JSI}}}}=\frac{| A\cap B| }{| A\cup B| }$$
    where *A* and *B* represent the sets of biomarkers identified by two distinct integration methods. Here, ∣*A* ∩ *B*∣ is the count of biomarkers common to both sets *A* and *B*, and ∣*A* ∪ *B*∣ denotes the total count of unique biomarkers identified when both sets are combined. The JSI score ranges from 0 to 1, with 0 indicating no overlap (no shared biomarkers) and 1 indicating complete congruence (all biomarkers are shared).
    Within the framework of single-cell data integration, a higher JSI suggests a stronger agreement between methods regarding the biomarkers detected, implying reliability and robustness in the biomarker identification process. On the contrary, a lower JSI may indicate unique discoveries or method-specific sensitivities in biomarker detection.

#### Batch correction

- iLISI (integration Local Inverse Simpson’s Index^41^). iLISI selects the nearest neighbors based on the local distribution of distances. The selected neighbors are then used to compute the inverse Simpson’s index for diversity, which is the number of batch labels present in this neighborhood. The higher the score, the better the batch mixing. The iLISI score is normalized to [0,1] for better comparison.
- kBET The kBET algorithm^66^ is a widely used batch effect quantification metric. kBET measures the bias of a batch variable in the kNN graph. Specifically, kBET is quantified as the average rejection rate of Chi-squared tests of local vs global batch label distributions. We normalize the kBET metric range to [0,1] as^12^, so a higher kBET score means better batch mixing.
- Graph connectivity (GC) is used to evaluate the mixing between omics, and its calculation is defined as:
  5 $${{{\rm{GC}}}}=\frac{1}{M}{\sum }_{j=1}^{M}\frac{\left|LC{C}_{j}\right|}{{N}_{j}}$$
  Where *LCC*_*j*_ denotes the number of cells with the largest connected component in the k-nearest neighbor graph for cell type *j*, *N*_*j*_ is the number of cells in cell type *j*, and *M* is the total number of cell types. The obtained value is between 0 and 1, and the higher the value, the better the omics mixing effect.
- Batch-average silhouette width (Batch-ASW) For the batch mixing score, we consider the absolute silhouette width, *s*^(*i*)^, on batch labels per cell *i*. Here, 0 indicates that batches are well mixed, and any deviation from 0 indicates a batch effect
  6 $${s}_{{{{\rm{batch}}}}}^{(i)}=| {s}^{(i)}|$$
  To ensure higher scores indicate better batch mixing, these scores are scaled by subtracting them from 1. As we expect batches to integrate within cell identity clusters, we compute the Batch − ASW_*j*_ score for each cell label *j* separately, using the equation:
  7 $${{{\rm{Batch}}}}-{{{{\rm{ASW}}}}}_{j}=\frac{1}{\left|{C}_{j}\right|}{\sum }_{i\in {C}_{j}}1-{s}_{{{{\rm{batch}}}}}^{(i)}$$
  To obtain the final Batch − ASW score, the label-specific Batch − ASW_*j*_ scores are averaged:
  8 $${{{\rm{Batch}}}}-{{{\rm{ASW}}}}=\frac{1}{| M| }{\sum }_{j\in M}{{{\rm{Batch}}}}-{{{{\rm{ASW}}}}}_{j}$$
  Here, *M* is the set of unique cell labels. Overall, a Batch − ASW of 1 represents ideal batch mixing and a value of 0 indicates strongly separated batches. We use the scikit-learn^64^ (v.0.22.1) implementation to compute these scores.

#### Computation efficiency

In order to show the superiority of the computation efficiency of different methods more intuitively, we convert the running time *T*_*i*_ (in seconds) of method *i* to Relative_Time_Score:

9 $${{{\rm{Relative}}}}\_{{{\rm{Time}}}}\_{{{\rm{Score}}}}=\frac{\max (\log T)-\log {T}_{i}}{\max (\log T)-\min (\log T)},$$

where $\max (\log T)$ and $\min (\log T)$ represent the logarithm of the maximum and minimum time value in all the methods considered, respectively. These two values serve as a reference point for the calculation. Relative_Time_Score provides a normalized measure that expresses how far the specific time value is from the maximum time value compared to the entire time range. A higher Relative_Time_Score indicates that the time complexity of that method is better, while a lower score suggests that the running time for this method is longer.

### Benchmarking setup

#### Hyperparameter tuning

Given the numerous methods we consider, each with its distinct characteristics, we aim for a fair comparison by categorizing them into three groups and employing corresponding strategies for each. First, for methods that do not specify explicit hyperparameters or are written in R, we adhere to the default parameters of each algorithm. This approach guarantees a fair comparison by minimizing variations arising from differing settings. Second, for algorithms with documented impacts of parameter variations, such as UnionCom, Pamona, and GLUE, we follow the recommendations in their respective literature to ensure their optimal performance. Lastly, for methods like scMDC, scJoint, Cobolt, and scGPT, where their literature offers a range of parameter choices without specifying the optimal settings, we conduct a systematic exploration of these parameters. By thoroughly traversing the defined ranges, we identify the optimal configurations to report in our experiments, thus capturing the best possible performance for these methodologies. This multifaceted approach to hyperparameter tuning enhances the reliability and validity of our comparative analysis.

#### Consistent workstation in performance evaluation

By running all methods on a maximum of two GPU nodes of an internal workstation equipped with NVIDIA GeForce RTX 3090 and memory of 24,564 MiB, we maintain consistency in the computational environment, enabling a reliable evaluation of performance differences solely based on algorithmic variations rather than hardware disparities. This approach not only enhances the reproducibility of our results but also facilitates a more accurate assessment of each method’s efficacy.

Detailed implementation of all the methods is listed below:

- GLUE (0.3.2)^11^ For the Graph-guided Variational Autoencoders (GLUE) method, we leverage its unique approach to data integration by constructing a graph that captures the relationships between cells across different modalities. The variational autoencoder is then guided by this graph to generate a cohesive latent space that effectively merges the datasets. The implementation is conducted using the GLUE software package, with parameters set to default, referring to their manuscript unless otherwise specified for our datasets.
- TotalVI (scvi-tools=1.2.2)^27^ Total Variational Inference (TotalVI) integrates single-cell RNA sequencing and protein abundance data by constructing a deep generative model that infers a joint representation. We utilize the scvi-tools implementation of TotalVI, normalizing the data according to the provided protocols and using the recommended settings to ensure consistency with other methods being benchmarked. The model is trained until convergence, with early stopping to prevent overfitting, ensuring that the learned latent space accurately captures both the transcriptomic and proteomic profiles of the cells.
- scMoMaT (0.2.2)^17^ Matrix Tri-factorization (scMoMaT) employs a tri-factorization approach to integrate multiple types of genomic data. For our implementation, we process each dataset by normalizing and selecting highly variable genes, then apply scMoMaT to decompose the matrices into shared and specific factors, aiming to capture the underlying biological signals while distinguishing between cell types and conditions. Parameters are tuned to balance the reconstruction accuracy and the integration of diverse datasets.
- LIGER (rliger=2.1.0)^40^ Integrative Nonnegative Matrix Factorization (LIGER) uses an algorithmic strategy to identify shared and distinct patterns across datasets from different omics layers. Our application of LIGER follows a standardized preprocessing pipeline, where we normalize the data, identify variable features, and then run the LIGER algorithm. The integration is performed without any pre-initialized matrices, and the model is iterated until it reaches a stable solution that reflects both joint and individual dataset features.
- MOFA (0.7.2)^14^ MOFA stands for multi-omic factor analysis. MOFA takes *M* data matrices as input, with one or more from each data modality. MOFA decomposes these matrices into a matrix of factors (*Z*) for each sample and *M* weight matrices, one for each data modality. The model makes use of a variational Bayesian framework to infer the parameters *Z* and *M* weight matrices. The number of factors is set to 5. The training iteration is set to 100. All other hyperparameters are set to default values.
- PCA PCA is a simple and straightforward baseline for multiomics integration. The scRNA-seq latent embedding is computed as the top 50 principal components of the gene expression profile. The scATAC-seq latent embeddings are computed by latent semantic indexing, following the same procedures as^38^ and^11^.
- bindSC (1.0.0)^39^ bindSC is designed to integrate single-cell multi-omics data of unpaired cases. The key algorithm of bindSC is the utilization of a transition matrix *Z* as a bridge to integrate observed *X* and *Y*. The optimization process is to solve matrix *Z* by maximizing the correlation of pair (*X*, *Z*) and the correlation of pair (*Y*, *Z*). The correspondence is calculated by standard CCA (Canonical Correlation Analysis). Following the default setting, we use the dimReduce function to reduce the columns of *X* (RNA-seq) and rows of Z to 30. We set the number of singular values for *Y* (ATAC-seq) to 50 when using the runSVD function. For the key function BiCCA, the parameters include alpha = 0.5, lambda = 0.5, and K = 15.
- Seurat4 (5.2.0)^21^ Seurat4 introduces weighted-nearest neighbor analysis to learn the relative utility of each data type in each cell, enabling integration of multiple modalities of single cell^21^. We run Seurat4 on the default setting that the dimension after UMAP reduction is 30. All other steps follow the default Seurat4 workflow.
- Seurat5 (5.2.0)^22^ Seurat5 is developed to map single-cell sequencing profiles to comprehensive reference datasets, which integrates single-cell datasets across modalities using a multiomic dataset as a molecular bridge. In this study, we adapt each dataset itself as its own bridge to evaluate the various aspects of Seurat’s generated embeddings, so we only implement Seurat5 on paired cases. For other settings, we follow the examples of integration provided by ref. ^22^.
- MMD-MA^18^ MMD-MA uses a maximum mean discrepancy (MMD) measure-based manifold alignment algorithm to integrate single-cell data. We use the runMMD-MA.py provided by ref. ^11^ to implement.
- UnionCom (0.9.0)^20^ UnionCom is an unsupervised topological alignment based method. We also use the runUnicom.py provided by ref. ^11^ to implement.
- scVI (0.17.1)^42^ scVI is a software package designed for performing probabilistic modeling and conducting analysis on single-cell omics data. Here we use the .get_latent_representation() function in version 0.17.1 to get the reduced embedding for different omics data. The n_layers is set to 2, n_latent is set to 30, and we use a negative binomial distribution for calculating the gene likelihood, consistent with their tutorial, to guarantee generally good performance for the integration task.
- scMDC (1.0.1)^23^ In Single Cell Multimodal Deep Clustering (scMDC), the multimodal deep learning clustering method, there is a single encoder that handles the combined data, and there are separate decoders for each modality within the multimodal dataset. We use the scripts and commands provided by the authors and further process the datasets following their instructions. We apply the learning rate that yields the best performance from [0.01, 0.005, 0.001], which is 0.01. All other hyperparameters are set according to their recommendation.
- Online-iNMF (rliger=2.1.0)^16^ For Online integrative non-negative matrix factorization (iNMF), we use the core functions and packages written in the R programming language to integrate the multi-omics datasets on top of normalizing, scaling, and selecting the highly variable genes. To be fair to all the other approaches, we do not initialize the matrices following GLUE.
- DeepMAPS (1.0)^43^ DeepMAPS (Deep learning-based Multi-omics Analysis Platform for Single-cell data), adopts a heterogeneous graph transformer framework for cell-type-specific biological network inference from single-cell multi-omics data. We follow the tutorial provided by the authors to get the integration results of all the paired datasets. The number of attention heads is set to 16, the number of hidden dimensions is set to 128, the learning rate is set to 0.2, and all the other hyperparameters are defaults.
- Cobolt (0.0.1)^25^ Cobolt estimates the representations of cells across modalities by applying a Multimodal Variational Autoencoder combined with a hierarchical generative model. It generalizes to unpaired datasets by leveraging paired sequences as bridging information. For our two unpaired datasets *Muto-2021* (*Homo sapiens* kidney) and *Yao-2021* (*Mus musculus* MOp), *PBMC-10k* (*Homo sapiens* PBMC) and *Chen-2019* (*Mus musculus* cortex) are used as the bridge dataset, respectively. We follow the standard tutorial for gene selection and hyperparameter settings. We search for the learning rate recommended (0.01, 0.005, 0.001). We find 0.001 is the best and most applicable, as other values resulted in ValueError.
- Pamona (0.1.0)^19^ Pamona formulates the integration task as a partial manifold alignment problem based on a partial Gromov-Wasserstein optimal transport framework, which aims at delineating and aligning structures not only between but also within different modalities. We follow the documented procedure of normalizing data and applying the model. All hyperparameters are set the same as in the published codes.
- scJoint^24^ scJoint focuses on aligning modalities via a semi-supervised transfer learning neural network framework, co-training labeled scRNA-seq data and unlabeled data from the other modality, where measures from the two modalities are not necessarily paired. Procedures and hyperparameters are conducted and set guided by tutorials and default codes posted publicly. Moreover, we also chose the best learning rate among [0.01, 0.005, 0.001].
- Harmony (0.0.10)^41^ Harmony is an integration method that projects data into a shared embedding space by iteratively correcting for known batch effects, such as differences across modalities. In our implementation, we apply the *harmonypy* package to infer joint embeddings based on the PCA-reduced features of concatenated RNA and ATAC data, using modality labels as batch information.
- scGPT^29^ We follow the official implementation of scGPT on *PBMC-10k* and use the zero-shot mode to obtain the cell embeddings for the rest of the datasets. We select the scGPT models provided based on the source of each dataset. For the *PBMC-10k* dataset, we utilize the scGPT_bc model, while for the *Muto-2021* kidney dataset, we employ the scGPT_kidney model. Since there is no specific pre-trained model available for mouse samples, we do not include mouse datasets in the evaluation of scGPT. All hyperparameters mentioned in the code are set to their default values. As for the fine-tuning part, we adopt the default way of scGPT’s multi-omics integration as scGPT-Scratch, which only inherits the pre-trained gene tokens’ embeddings but trains transformer modules from scratch. scGPT-ft also inherits the first few pre-trained transformer modules on the basis of scGPT-Scratch (best setting is when keeping the first eight modules). The scGPT-Adapter method takes scVI as the adapter, which treats the concatenation of scGPT’s zero-shot output of scRNA-seq and scATAC-seq data as input for scVI, and adopts the concatenation of two modules as the reconstruction target. During the fine-tuning process of the scGPT-Scratch method, we use the default hyperparameters given by ref. ^29^. However, we also found that fine-tuning with 25 epochs is better than 10 epochs (default setting) when we use scGPT_bc model, while it is worse than 10 epochs when we use scGPT_human. It demonstrates that the fine-tuning performance of scGPT also depends on training epochs and the selected pre-trained model version. We further assess the impact of the number of transformer layers (Supplementary Table 4), with the optimal performance obtained when loading 8 transformer layers from the human-pretrained model. However, the scGPT-Adapter always surpasses all those fine-tuned models and is a relatively better and stable strategy to consider.
- Geneformer (0.1.0)^28^ Geneformer, a foundational transformer model, undergoes pretraining on an extensive corpus comprising approximately 30 million single-cell transcriptomes. This pretraining facilitates context-aware predictions, particularly in scenarios where data availability is limited within the realm of network biology. Following their official instructions, we first fine-tune the pre-trained model geneformer-12L-30M with a cell type classification object and then extract the embeddings with *emb*_*layer* set to 0.
- scFoundation^30^ scFoundation is a large-scale pre-trained model with 100M parameters. We use their official API for cell embedding inference. https://aigp.biomap.com/models.
- UCE^31^ UCE is a foundation model pre-trained on millions of cells. A key feature of UCE is that it can generalize across different species by mapping genes to their protein product. We use the pre-trained 4-layer checkpoint of UCE and use all the default parameters when extracting cell embeddings. The implementation of the UCE-Adapter model is similar to the scGPT-Adapter, which also takes scVI as an adapter concatenated to the pre-trained UCE.

### Statistics and reproducibility

Datasets were selected to cover diverse species, organs, sequencing technologies, and pairing scenarios. No statistical method was used to predetermine sample size. No data were excluded from the analyses. The experiments were not randomized. The investigators were not blinded to allocation during experiments and outcome assessment. All benchmarking experiments were conducted on a standardized computational environment (NVIDIA GeForce RTX 3090, 128 GB RAM) to ensure reproducibility. Hyperparameters for each method were tuned following the strategies described in the Benchmarking Setup section.

### Reporting summary

Further information on research design is available in the Nature Portfolio Reporting Summary linked to this article.

## Supplementary information

Supplementary Information

Description of Additional Supplementary Files

Supplementary Data 1

Supplementary Data 2

Supplementary Data 3

Supplementary Data 4

Supplementary Data 5

Reporting Summary

Transparent Peer Review file

## Source data

Source Data

## Data Availability

All datasets used in this study are publicly available. The *PBMC-10k* dataset is available from 10X Genomics [https://www.10xgenomics.com/datasets/pbmc-from-a-healthy-donor-granulocytes-removed-through-cell-sorting-10-k-1-standard-1-0-0]. The *Chen-2019* SNARE-seq dataset is available from the Gene Expression Omnibus (GEO) under accession code GSE126074. The *Ma-2020* SHARE-seq dataset is available from GEO under accession code GSE140203. The *Muto-2021* kidney dataset is available from GEO under accession code GSE151302. The *Yao-2021* MOp dataset is available from the BICCN NeMO archive [https://assets.nemoarchive.org/dat-ch1nqb7]. The Triple dataset combines scATAC-seq and Drop-seq data from Saunders et al.^50^, available from GEO under accession code GSE116470, and snmC-seq data from Luo et al.^5^, available from GEO under accession code GSE97179. Simulated datasets were generated using scMultiSim^60^ as described in Methods. Source data are provided with this paper.

## Code availability

Code for reproducing the baseline results and figures is available at the SCMBench GitHub repository [https://github.com/ml4bio/SCMBench] and archived on Zenodo^67^ under the MIT license.
