# Supplementary material for: SCMBench: benchmarking domain-specific and foundation models for single-cell multi-omics data integration
Source: Nat Commun. 2026 May 2;17:5967. doi: 10.1038/s41467-026-72570-x (PMC13342640; doi:10.1038/s41467-026-72570-x)
Supplement: Supplementary file 1 — Supplementary Information [file 41467_2026_72570_MOESM1_ESM.pdf]

Supplementary Information for SCMBench

Yixuan Wang[1,2], Yimin Fan[1], Xuesong Wang[1], Tingyang Yu[3], Yongshuo Zong[4], Xinyuan Liu[1], Gaoyang Zhong[5], Meitong Liu[6], Qing Li[1], Kin hei Lee[1], Khachatur Dallakyan[1], Zhichao Hu[7], Yaqian Qi[8], Junjie Huang[9], Gengjie Jia[10], Jiao Yuan[11], Ting-Fung Chan[12,13], Xin Gao[14,15,16], Irwin King[1], and Yu Li*[1,2,17,18,19,20,21]

[1]Department of Computer Science and Engineering, CUHK, Hong Kong SAR, China
[2]Mohamed bin Zayed University of Artificial Intelligence, Abu Dhabi, United Arab Emirates
[3]Department of Information Engineering, CUHK, Hong Kong SAR, China
[4]School of Informatics, University of Edinburgh, Edinburgh, UK
[5]Department of Forensic Medicine, Tongji Medical College, Huazhong University of Science and Technology, Wuhan, China
[6]Department of Computer Science, HKU, Hong Kong SAR, China
[7]Department of Information Technology & Operations Management, Nanyang Business School, NTU, Singapore
[8]Quantitative Methods and Modeling, Baruch College, CUNY, 55 Lexington Ave, New York, NY 10010
[9]JC School of Public Health and Primary Care, Faculty of Medicine, CUHK
[10]Genome Analysis Laboratory of the Ministry of Agriculture and Rural Affairs, Agricultural Genomics Institute at Shenzhen, Chinese Academy of Agricultural Sciences, Shenzhen 518120, China
[11]GMU-GIBH Joint School of Life Sciences, The Guangdong-Hong Kong-Macau Joint Laboratory for Cell Fate Regulation and Diseases, Guangzhou National Laboratory, Guangzhou Medical University, Guangzhou, China
[12]School of Life Sciences, CUHK, Hong Kong SAR, China
[13]State Key Laboratory of Agrobiotechnology, The Chinese University of Hong Kong, Hong Kong SAR, China
[14]Computer Science Program, Computer, Electrical and Mathematical Sciences and Engineering Division, King Abdullah University of Science and Technology (KAUST), Thuwal 23955-6900, Kingdom of Saudi Arabia.
[15]Center of Excellence for Smart Health (KCSH), King Abdullah University of Science and Technology (KAUST), Thuwal 23955-6900, Kingdom of Saudi Arabia.
[16]Center of Excellence on Generative AI, King Abdullah University of Science and Technology (KAUST), Thuwal 23955-6900, Kingdom of Saudi Arabia.
[17]The CUHK Shenzhen Research Institute, Hi-Tech Park, Nanshan, Shenzhen, 518057, China
[18]Institute for Medical Engineering and Science, Massachusetts Institute of Technology, Cambridge, MA, USA
[19]Wyss Institute for Biologically Inspired Engineering, Harvard University, Boston, MA, USA
[20]Broad Institute of MIT and Harvard, Cambridge, MA, USA
[21]Kunming Institute of Zoology - The Chinese University of Hong Kong Joint Laboratory of Bioresources and Molecular Research of Common Diseases, The Chinese University of Hong Kong, Hong Kong SAR, China

List of Tables

1 Real Datasets used in evaluation. 3
2 Classification and other information of the 23 integration methods. 3
3 Comparison of the existing benchmarking works. 4
4 Comparison between different versions of finetuned scGPT. 4

List of Figures

1 Analysis and comparison of ATAC-derived activity matrix of *PBMC-10k* dataset 6
2 Detailed integration accuracy 7
3 Benchmarking integration of scRNA-seq, scATAC-seq and DNA methylation in mouse cortex (*Triple* dataset). 8

*Corresponding Author. Email: liyu@cse.cuhk.edu.hk

4 Evaluation of biomarker detection on annotated biomarkers. 9
5 Biological conservation evaluation at the ATAC level. 10
6 Trajectory conservation (TC) scores of major and minor cell type groups of the *PBMC-10k* dataset. 11
7 Trajectory conservation scores and UMAP visualization of *PBMC-10k* dataset. 12
8 Trajectory conservation scores and UMAP visualization of *Chen-Traj* dataset of mouse cortex (Top6). 13
9 Performance Comparison of 10 multi-omics integration methods on six simulated datasets 14
10 Performance Comparison of 10 multi-omics integration methods on six simulated datasets (Continued) 15
11 UMAP visualization of three multiomics integration methods (PCA, scMDC, scMoMaT) on six simulated datasets with different levels of batch effects 16
12 UMAP visualization of three multiomics integration methods (scVI, iNMF, MOFA) on six simulated datasets with different levels of batch effects 17
13 UMAP visualization of three multiomics integration methods (Seurat5, Cobolt, scJoint) on six simulated datasets with different levels of batch effects 18
14 Critical difference diagrams of different metrics. 19
15 Comparison of running time between CPU and GPU versions. 20

Supplementary Table 1: **Real Datasets used in evaluation.** 'T' and 'F' stand for 'True' and 'False' respectively. Paired datasets: *PBMC-10k*, *Chen-2019*, *Ma-2020*; unpaired datasets: *Muto-2021*, *Yao-2021*.

Dataset	Species	Organ	Protocol	Omics	Cell numbers	Batch effect	Source
PBMC-10k	Homo sapiens	PBMC	10x Multiome	RNA-seq	9631	F	[1]
				ATAC-seq	9631		
Chen-2019	Mus musculus	Cortex	SNARE-seq	RNA-seq	9190	F	[2]
				ATAC-seq	9190		
Ma-2020	Mus musculus	Skin, Brain, Lung	SHARE-seq	RNA-seq	32231	T	[3]
				ATAC-seq	32231		
Muto-2021	Homo sapiens	Kidney	snRNA-seq	snRNA-seq	19985	T	[4]
			snATAC-seq	snATAC-seq	24205		
Yao-2021	Mus musculus	MOp	scRNA 10x v3	RNA-seq	69727	F	[5]
			snATAC-seq	ATAC-seq	54844		
Triple	Mus musculus	Cortex	scATAC 10x	ATAC-seq	2317	F	[6, 7]
			snmC-seq	DNA Methylation	3377		
			Drop-seq	RNA-seq	55803		

Supplementary Table 2: **Classification and other information of the 23 integration methods.** Methods details, including classification of methods, applicability of unpaired scenarios and Triple-omics integration, Programming Language, and so on.

Methods	Statistical-based	DL-based	DM or FM	Unpaired	Triple-Omics	Programming Language	Year	Reference
MOFA	✓		DM			Python	2018	[8]
MMD-MA	✓		DM			Python	2019	[9]
PCA	✓		DM	✓	✓	Python	2019	[10]
Harmony	✓		DM	✓		Python	2019	[11]
UnionCom	✓		DM			Python	2020	[12]
LIGER	✓		DM	✓	✓	R	2020	[13]
TotalVI		✓	DM			Python	2021	[14]
Seurat4	✓		DM	✓		R	2021	[15]
iNMF	✓		DM	✓	✓	R	2021	[16]
Cobolt		✓	DM	✓		Python	2021	[17]
scMDC		✓	DM			Python	2022	[18]
Pamona	✓		DM	✓		Python	2022	[19]
bindSC	✓		DM	✓		R	2022	[20]
scJoint		✓	DM	✓	✓	Python	2022	[21]
GLUE		✓	DM	✓	✓	Python	2022	[22]
DeepMAPS		✓	DM			R	2023	[23]
Seurat5	✓		DM	✓		R	2023	[24]
scMoMaT	✓		DM	✓	✓	Python	2023	[25]
scVI		✓	DM	✓	✓	Python	2023	[26]
Geneformer		✓	FM	✓		Python	2023	[27]
UCE		✓	FM	✓		Python	2023	[28]
scGPT		✓	FM	✓		Python	2024	[29]
scFoundation		✓	FM	✓		Python	2024	[30]

Supplementary Table 3: Comparison of the existing benchmarking works.

Paper	Omics	Datasets	Methods	Downstream Tasks			Comparing with FMs
				BD	TI	BC	
Lee [31]	2	3	9	✓			
Xiao [32]	2	3	12	✓	✓		
SCMBench	3	6	23	✓	✓	✓	✓

Supplementary Table 4: Comparison between different versions of finetuned scGPT.

Pretrain-model	#Transformer Layers	Load pre-trained encoder	MAP	NMI	ASW	ARI	Overall
Human	4	FALSE	0.7262	0.6731	0.5677	0.3454	0.5781
		TRUE	0.717	0.6621	0.5692	0.3262	0.568625
	8	FALSE	0.704	0.6875	0.567	0.3428	0.575325
		**TRUE**	**0.7421**	**0.6942**	**0.5661**	**0.3619**	**0.591075**
	12	FALSE	0.6992	0.6462	0.5628	0.333	0.5603
		TRUE	0.7245	0.6828	0.5697	0.327	0.576
Blood	4	FALSE	0.7221	0.6939	0.5658	0.3425	0.581075
		TRUE	0.6899	0.6981	0.5674	0.3451	0.575125
	8	FALSE	0.7192	0.6896	0.5664	0.326	0.5753
		TRUE	0.7242	0.6977	0.5712	0.3289	0.5805
	12	FALSE	0.6984	0.6656	0.5696	0.3528	0.5716
		TRUE	0.7137	0.6972	0.5702	0.358	0.584775

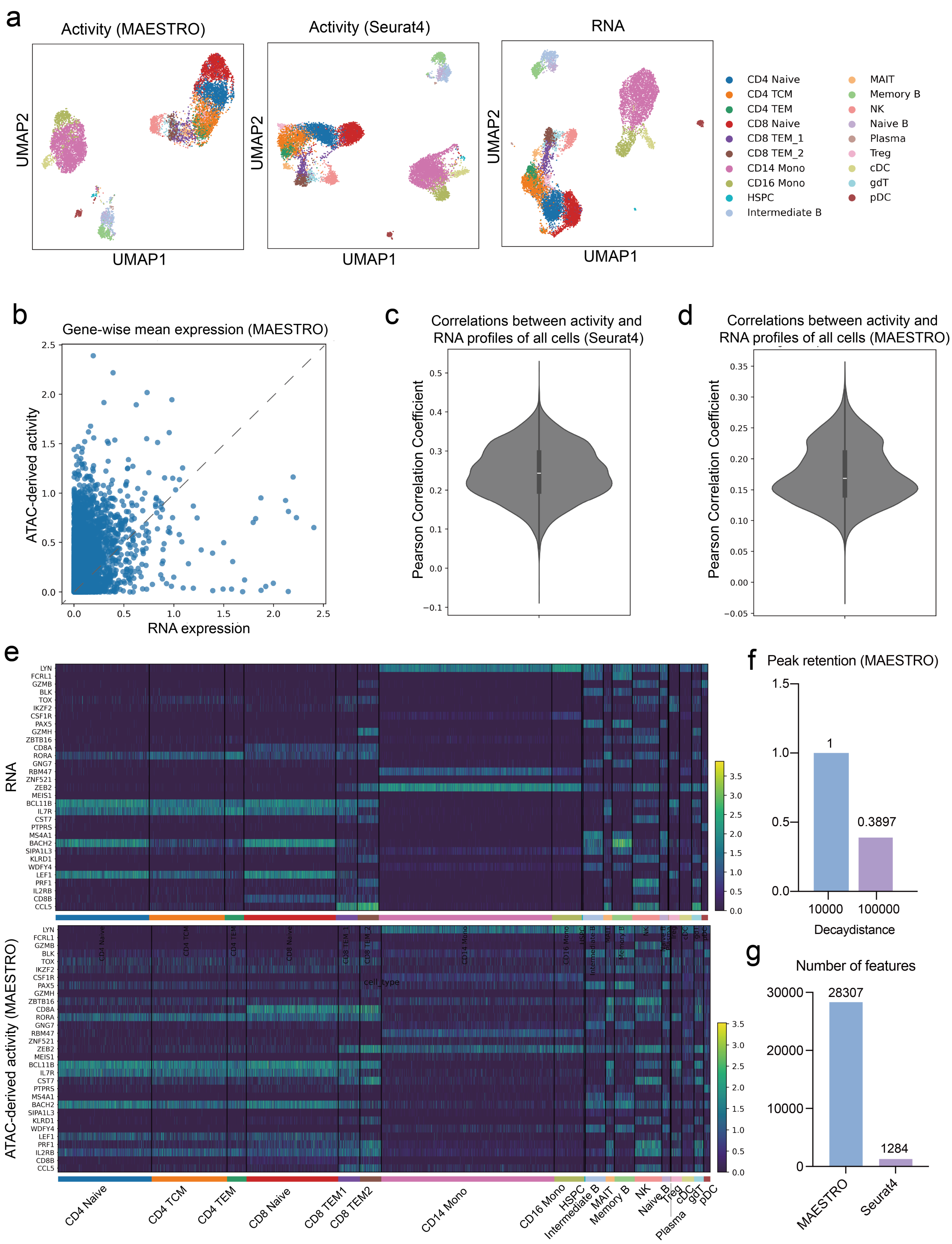
a
Activity (MAESTRO)
Activity (Seurat4)
RNA
UMAP1
UMAP2
CD4 Naive
CD4 TCM
CD4 TEM
CD8 Naive
CD8 TEM_1
CD8 TEM_2
CD14 Mono
CD16 Mono
HSPC
Intermediate B
MAIT
Memory B
NK
Naive B
Plasma
Treg
cDC
gdT
pDC
b
Gene-wise mean expression (MAESTRO)
ATAC-derived activity
RNA expression
c
Correlations between activity and RNA profiles of all cells (Seurat4)
Pearson Correlation Coefficient
d
Correlations between activity and RNA profiles of all cells (MAESTRO)
e
RNA
ATAC-derived activity (MAESTRO)
f
Peak retention (MAESTRO)
1
0.3897
10000
100000
Decaydistance
g
Number of features
28307
1284
MAESTRO
Seurat4

Supplementary Figure 1: **Analysis and comparison of ATAC-derived activity matrix of *PBMC-10k* dataset.** **a**, UMAP visualization of both the RNA expression and the corresponding ATAC-derived gene activity matrix from MAESTRO and Seurat4. **b**, Scatter plot comparing gene-wise average expression between RNA and ATAC-derived activity (MAESTRO) across all cells. Each dot represents one gene. **c-d**, Pearson correlation coefficients between RNA expression and ATAC-derived activity across all cells of Seurat4 (**c**) and MAESTRO (**d**). It reflects the intrinsic difference between chromatin accessibility and gene expression, suggesting that gene expression is influenced by factors beyond local chromatin accessibility near transcription start sites (TSSs). **e**, Comparison of expression patterns of differentially expressed genes in each cell type, between RNA and ATAC-derived activity matrices (MAESTRO). The activity matrix captures the expression patterns of genes with strong cell-type specificity, such as ZEB2 in CD14+ monocytes and CCL5 in CD8+ TEM cells. **f**, Peak retention analysis of the ATAC-derived activity matrix (MAESTRO) relative to the original ATAC-seq peak matrix. For each non-zero gene in the activity matrix, TSSs were identified and overlapping ATAC-seq peaks within a defined genomic window (decay distance) were retrieved. The peak retention ratio is defined as the proportion of matched peaks from the activity matrix relative to all non-zero peaks in the original ATAC-seq data. When the decay distance was set to 10,000 bp, the retention ratio reached 100%, whereas a decay distance of 100,000 bp resulted in a retention ratio of 38.97%. These results suggest that the transformation process preserves local regulatory information captured by ATAC-seq, and that the choice of decay distance substantially influences the resulting activity profiles. **g**, Comparison of the number of features in the ATAC-derived activity matrix between MAESTRO and Seurat4.

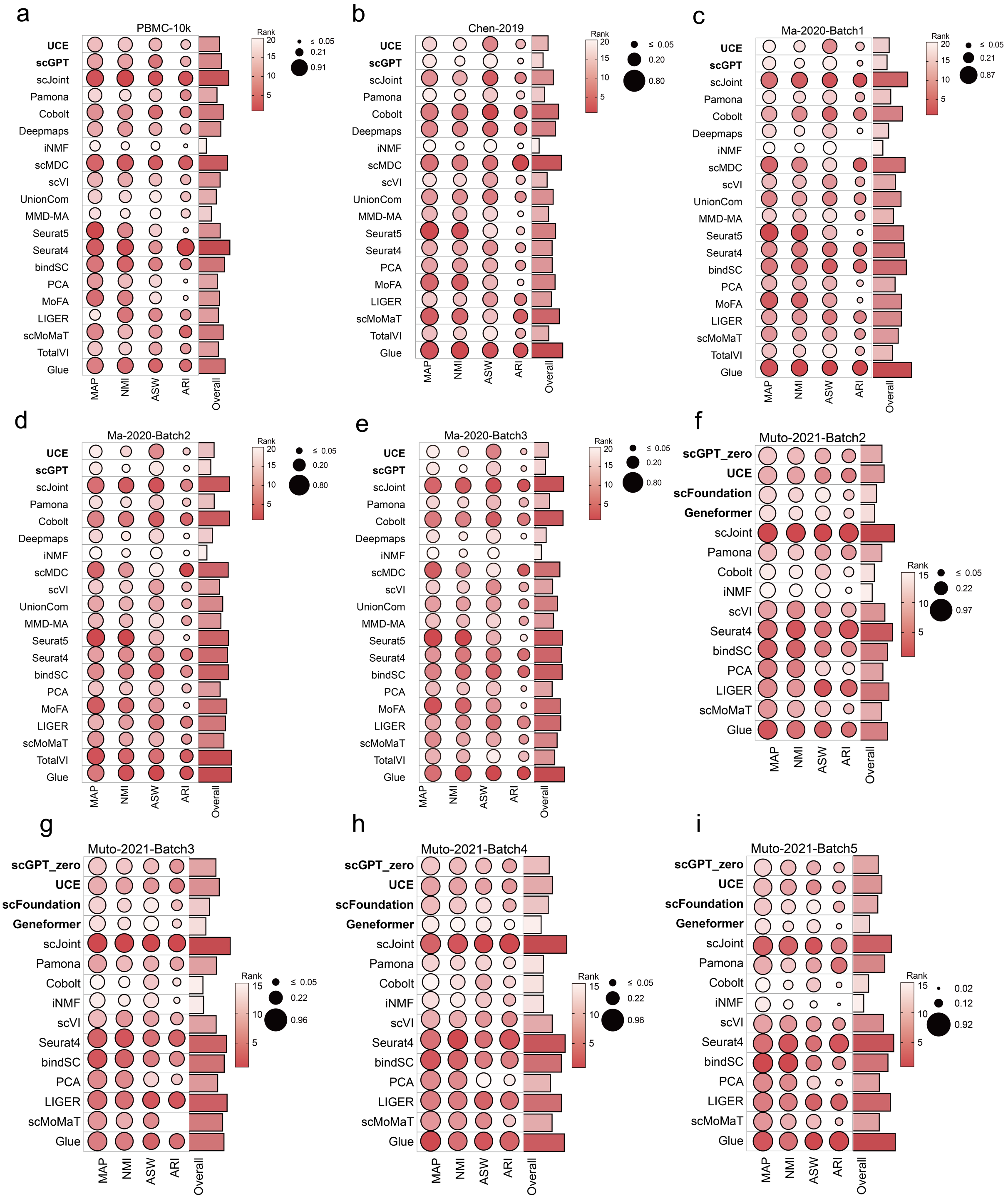

Supplementary Figure 2: **Detailed integration accuracy.** **a-e.** Integration accuracy of all the paired datasets, where *Ma-2020* is split into 3 smaller datasets according to the batch label on the purpose of batch effects removal. **f-i.** Integration accuracy of the representative unpaired datasets, where *Muto-2021* is split into 5 smaller datasets according to the batch label (Muto-2021-Batch1 is shown in Fig. 2 **c**). Since Yao-2021 contains over 120,000 samples, which is too large for more than half of the considered approaches, we only include it in Fig. 2 **d**.

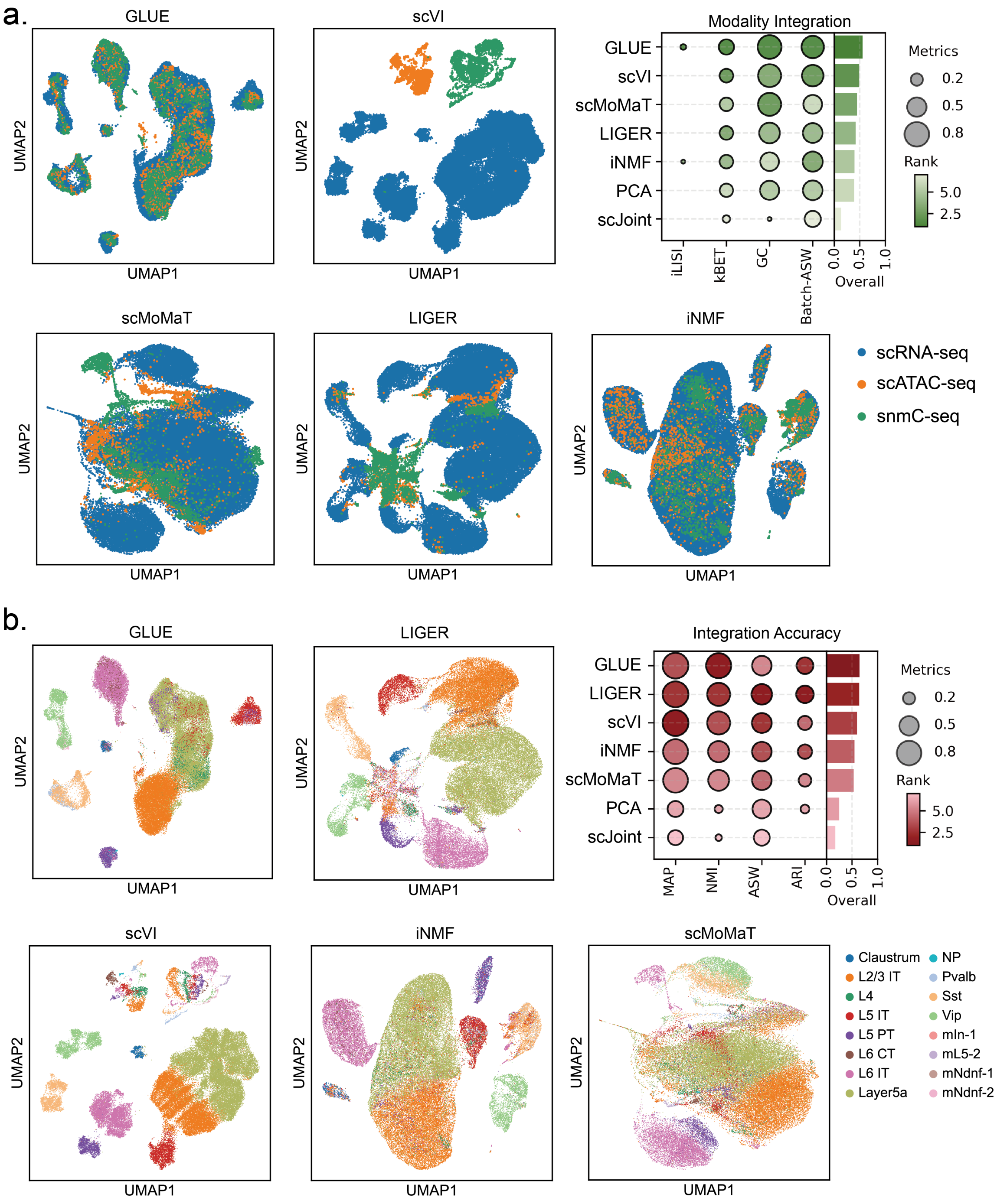

Supplementary Figure 3: **Benchmarking integration of scRNA-seq, scATAC-seq and DNA methylation in mouse cortex (*Triple* dataset). a,** Integration performance of seven methods capable of handling triple-omics data, assessed using batch correction metrics with modality treated as batch labels. **b,** Cell type clustering accuracy following integration. GLUE achieves the highest performance in both modality alignment and cell type resolution.

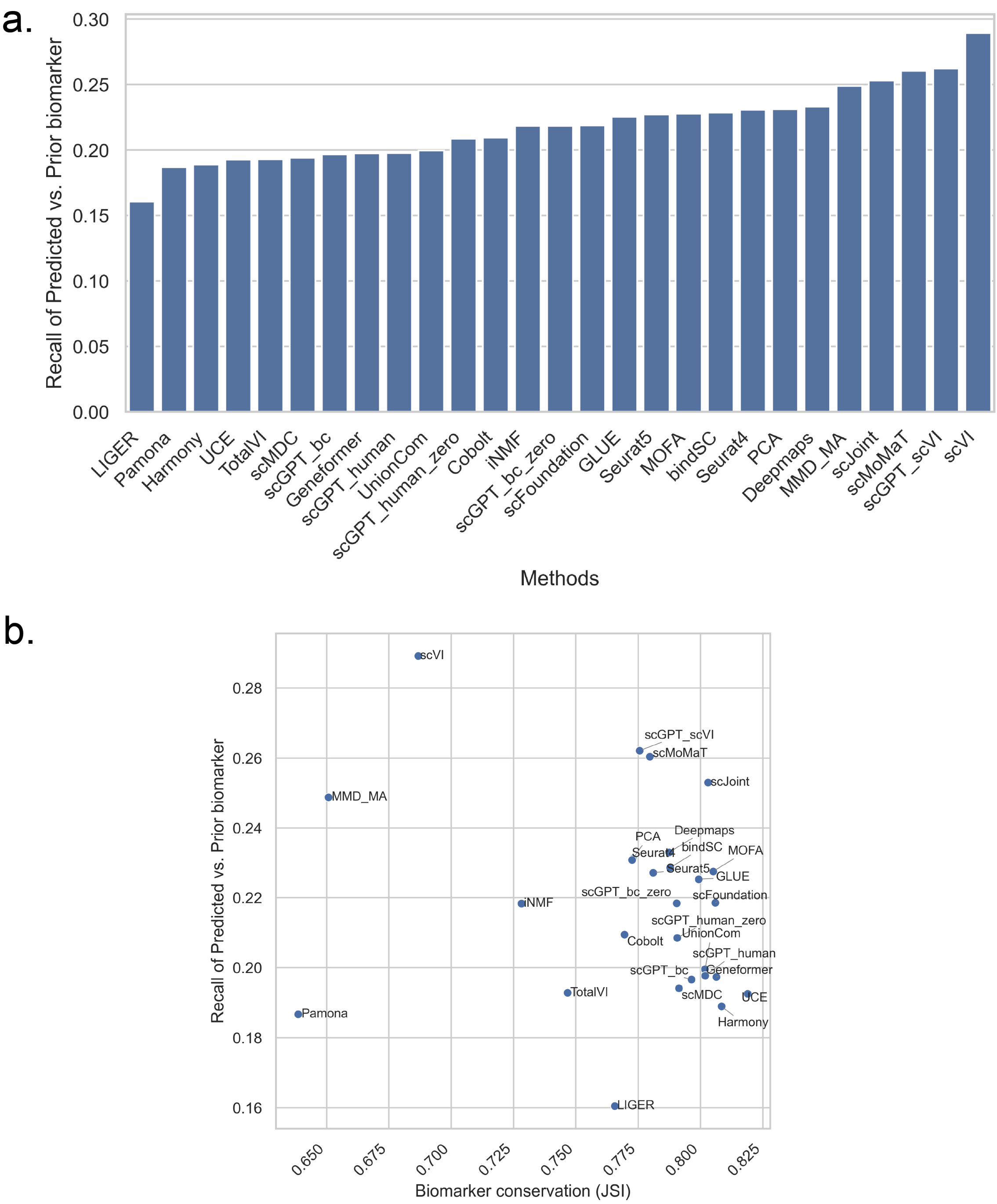

Supplementary Figure 4: **Evaluation of biomarker detection on annotated biomarkers. a.** Recall values of different methods in detecting known biomarkers, calculated by comparing DE genes from inferred cell type clusters against curated biomarkers in the CellMarker 2.0 database. **b.** Comparison of recall performance for predicted biomarkers and CellMarker biomarkers, plotted against biomarker conservation scores derived from statistically computed DEGs. Among them, scGPT-scVI, scMoMaT, and scJoint demonstrate consistent and superior detection of biologically validated markers across both analyses.

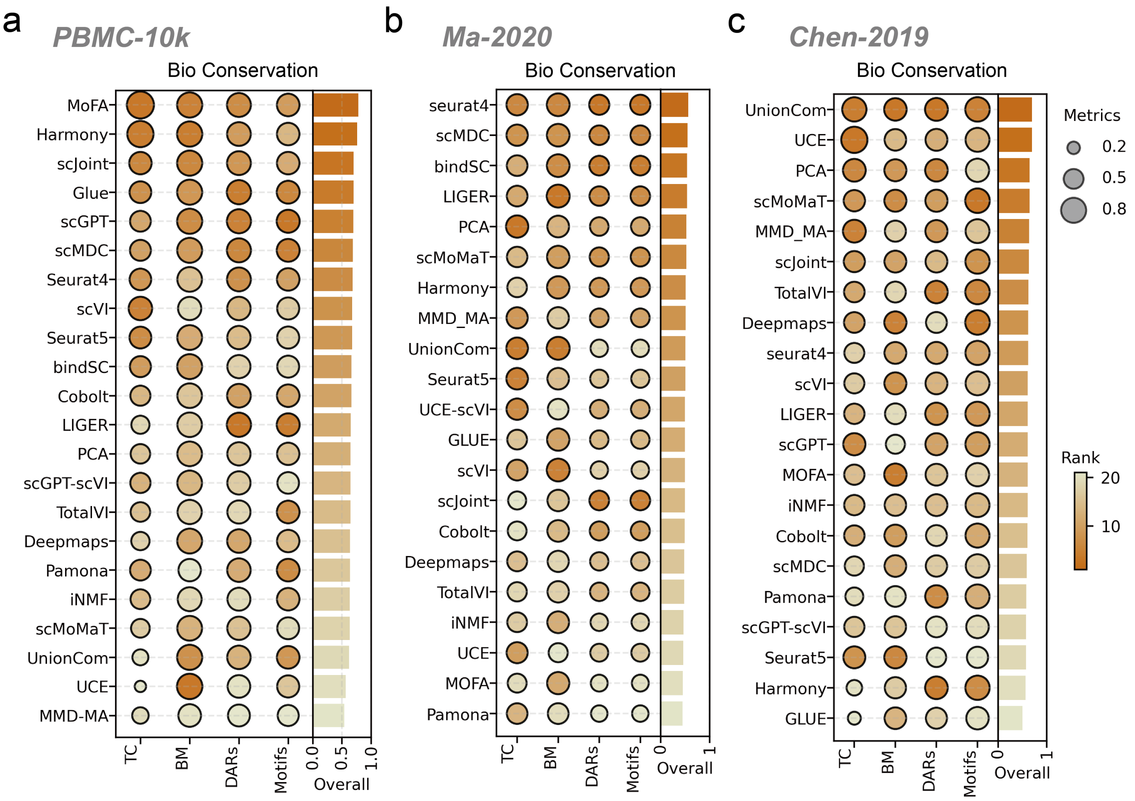

Supplementary Figure 5: **Biological conservation evaluation at the ATAC level. a-c.** Evaluation of biological conservation across four aspects: trajectory preservation, biomarker identification, differential accessible regions (DARs), and motif enrichment. The latter two are specific to the scATAC-seq modality. Results are presented for three datasets—*PBMC-10k*, *Ma-2020*, and *Chen-2019*—which include both human and mouse tissues. Methods are ranked for each dataset based on their overall biological conservation scores.

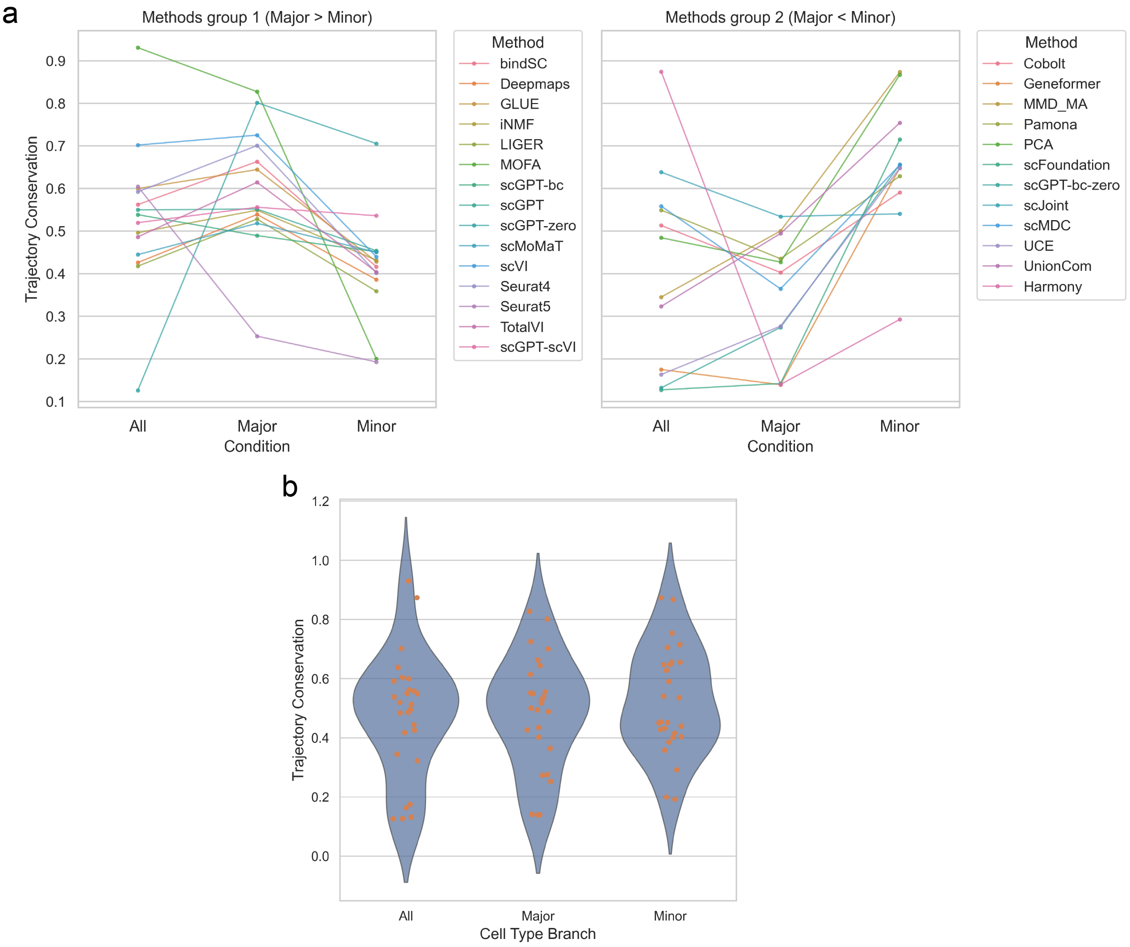

Supplementary Figure 6: **Trajectory conservation scores of major and minor cell type groups of the *PBMC-10k* dataset. a,** Comparison of TC scores calculated across three different subsets: all cells, major cell types, and minor cell types. And methods are grouped based on whether TC scores are higher in major cell types than in minor ones (Group 1: TC_major > TC_minor) or vice versa (Group 2: TC_major < TC_minor). **b,** The violin plot showing the overall distribution of TC scores in three cell groups. These results suggest that the size or abundance of cell types is not the primary determinant of trajectory inference performance, which varies across different methods.

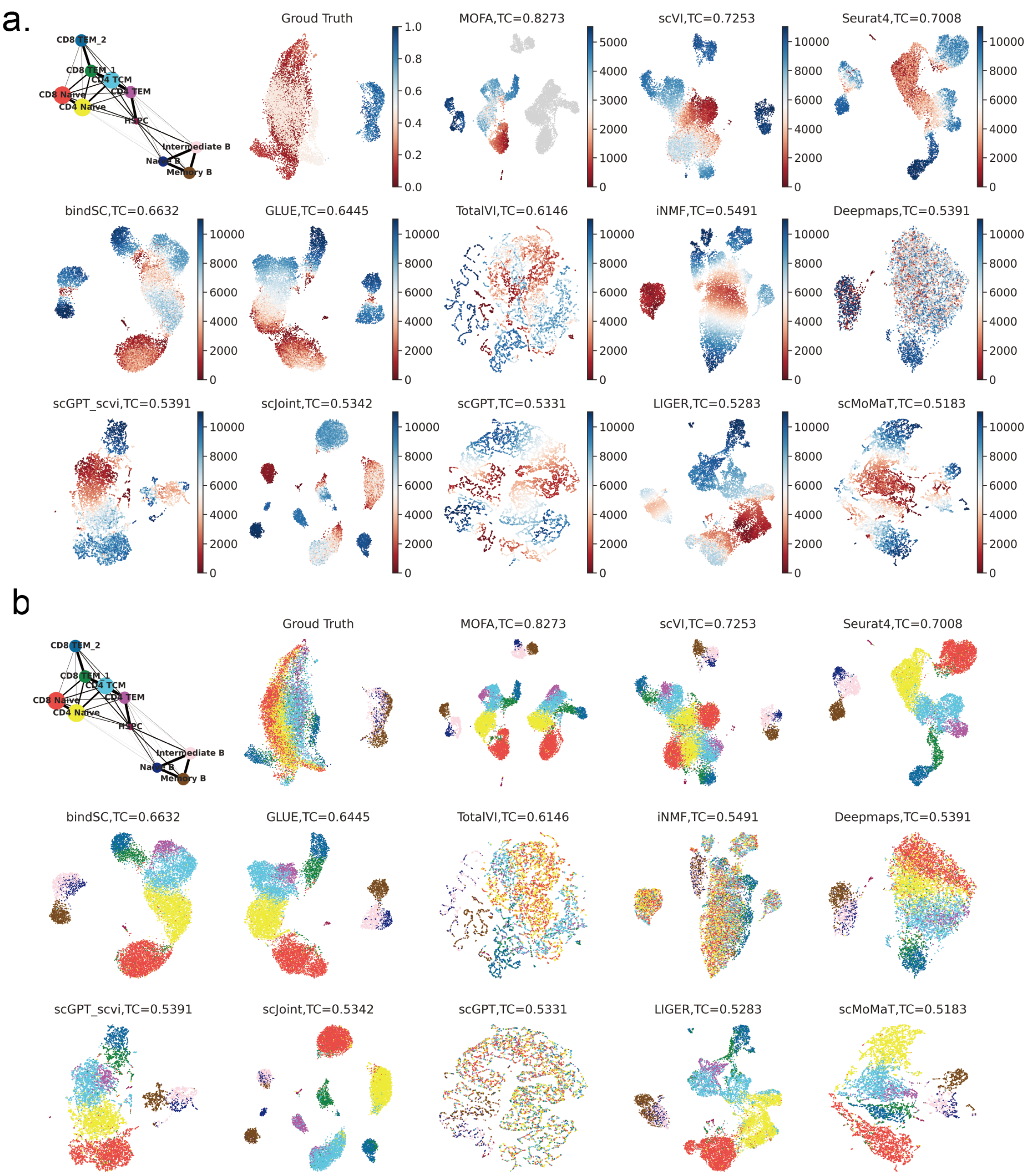

Supplementary Figure 7: **Trajectory conservation scores and UMAP visualization of *PBMC-10k* dataset.** PAGA plot of the ground truth and UMAP visualizations of the pseudotime inference result from the integrated cell embeddings of 10 major cell types. All subplots are arranged in descending order based on the TC score, and we show the UMAP plots for the top 13 methods. The pseudotime estimations and cell type annotations are presented in **a** and **b**.

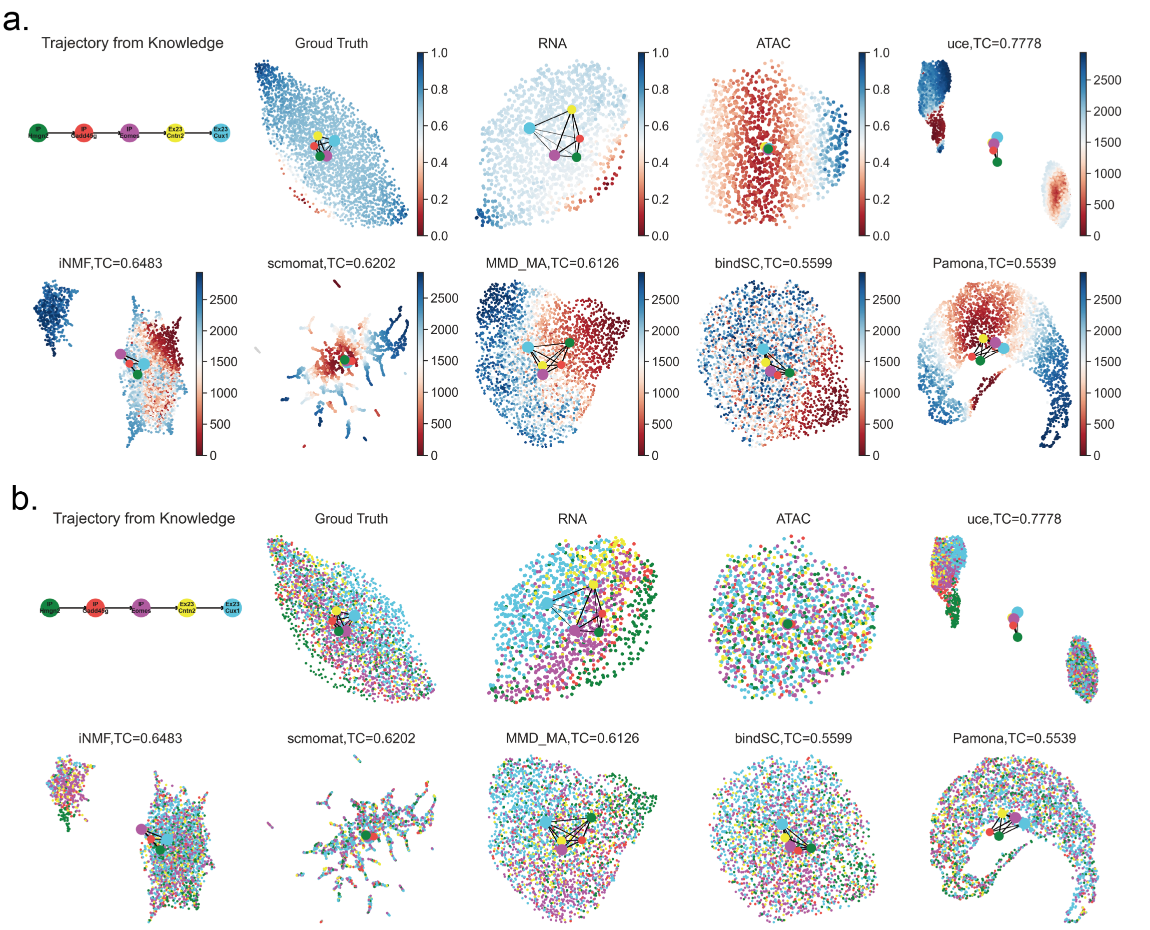

Supplementary Figure 8: **Trajectory conservation scores and UMAP visualization of *Chen-Traj* dataset of mouse cortex (Top6).** The pseudotime estimations and cell type annotations are presented in **a** and **b**.

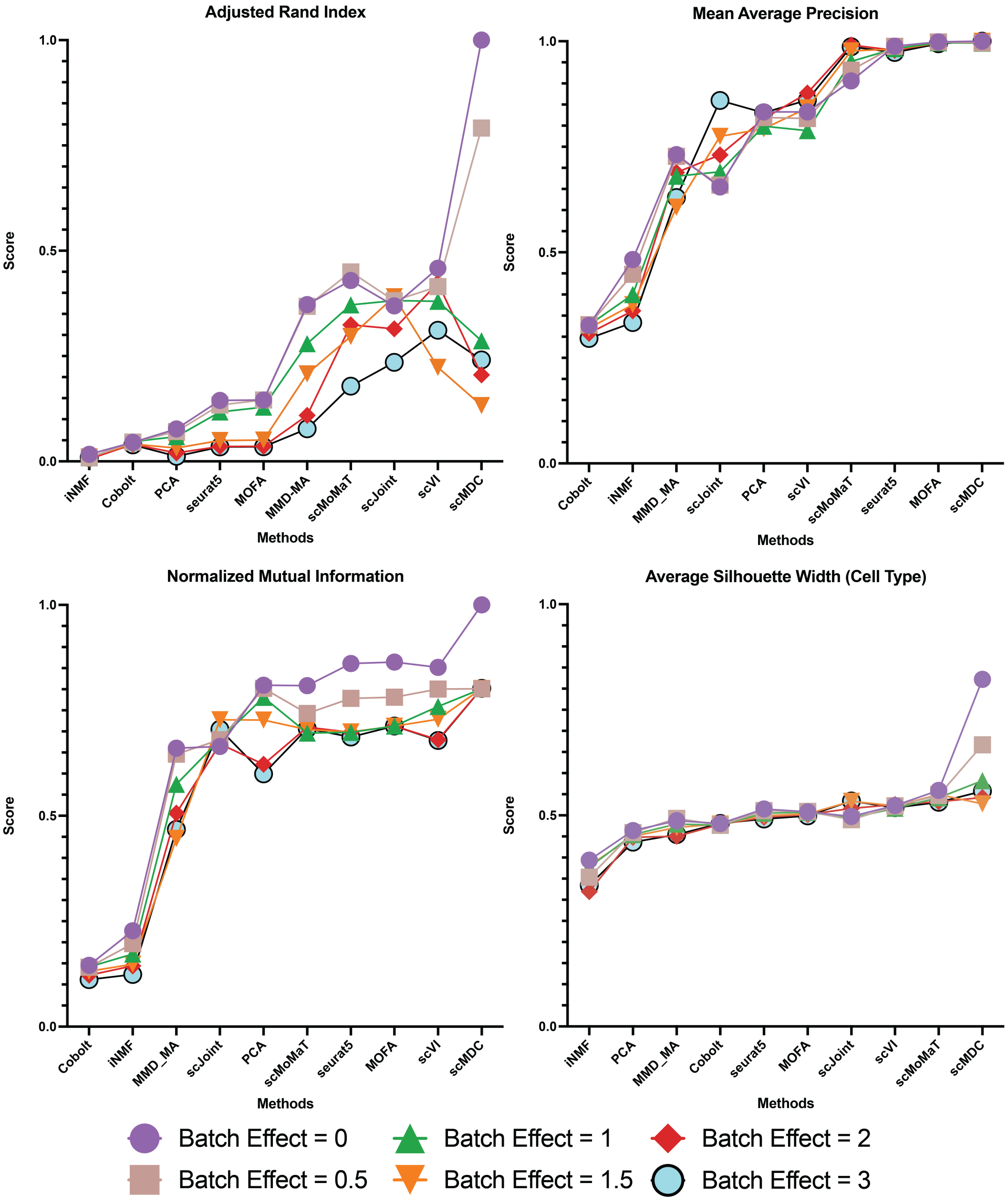

Supplementary Figure 9: **Performance Comparison of 10 multi-omics integration methods on six simulated datasets.** Performance of the integration accuracy is shown via Adjusted Rand Index (ARI), Mean Average Precision (MAP), Normalized Mutual Information (NMI), and Average Silhouette Width (Cell Type). For ARI, scMDC consistently outperforms other methods, especially at higher batch effects, while most methods show declining performance as batch effect increases. The MAP graph indicates an overall improvement for all methods moving right along the x-axis, with scMDC, MOFA, and Seurat5 performing well across different batch effects. NMI results mirror ARI trends, with scMDC showing strong performance. The Average Silhouette Width (Cell Type) graph shows less variation between effects, with a slight upward trend for most methods and a notable performance jump for scMDC in the weak batch effects.

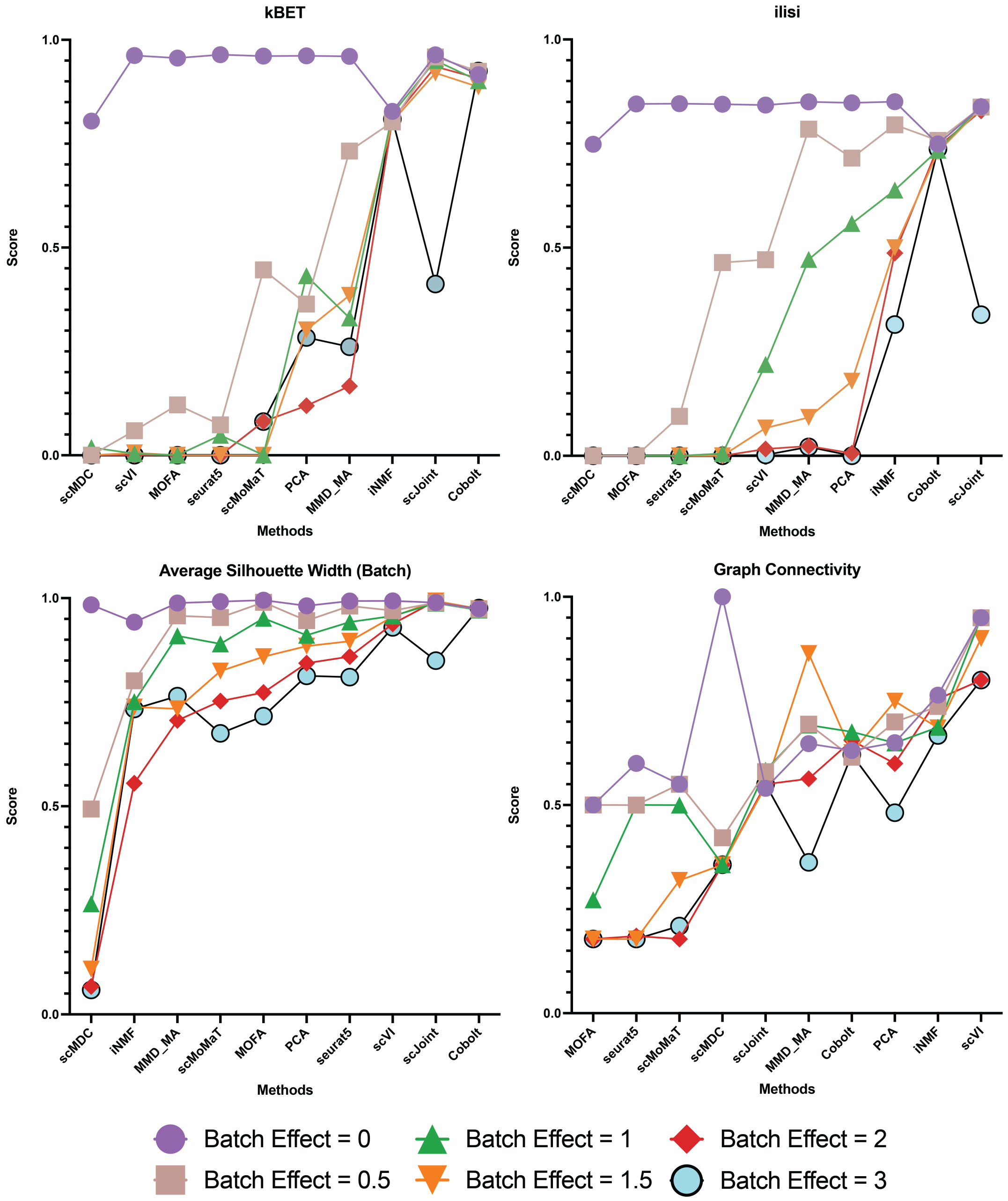

Supplementary Figure 10: **Performance Comparison of 10 multi-omics integration methods on six simulated datasets (Continued).** Continues the comparison with four additional metrics: kBET, LISI, Average Silhouette Width (Batch), and Graph Connectivity. The kBET and LISI graphs show high variability across methods and batch effects. Some methods like scMDC and MOFA show sharp performance drops as batch effect increases. The Average Silhouette Width (Batch) graph indicates good batch correction for most methods, with less variation between them compared to other metrics. Graph Connectivity results show high variability with no clear consistent top performer across all batch effects. Overall, these graphs suggest that Cobolt and scJoint perform strongly across multiple metrics and batch effect levels, particularly for clustering-related measures. However, the choice of method may depend on specific analysis goals and dataset characteristics, as different methods show strengths in different areas.

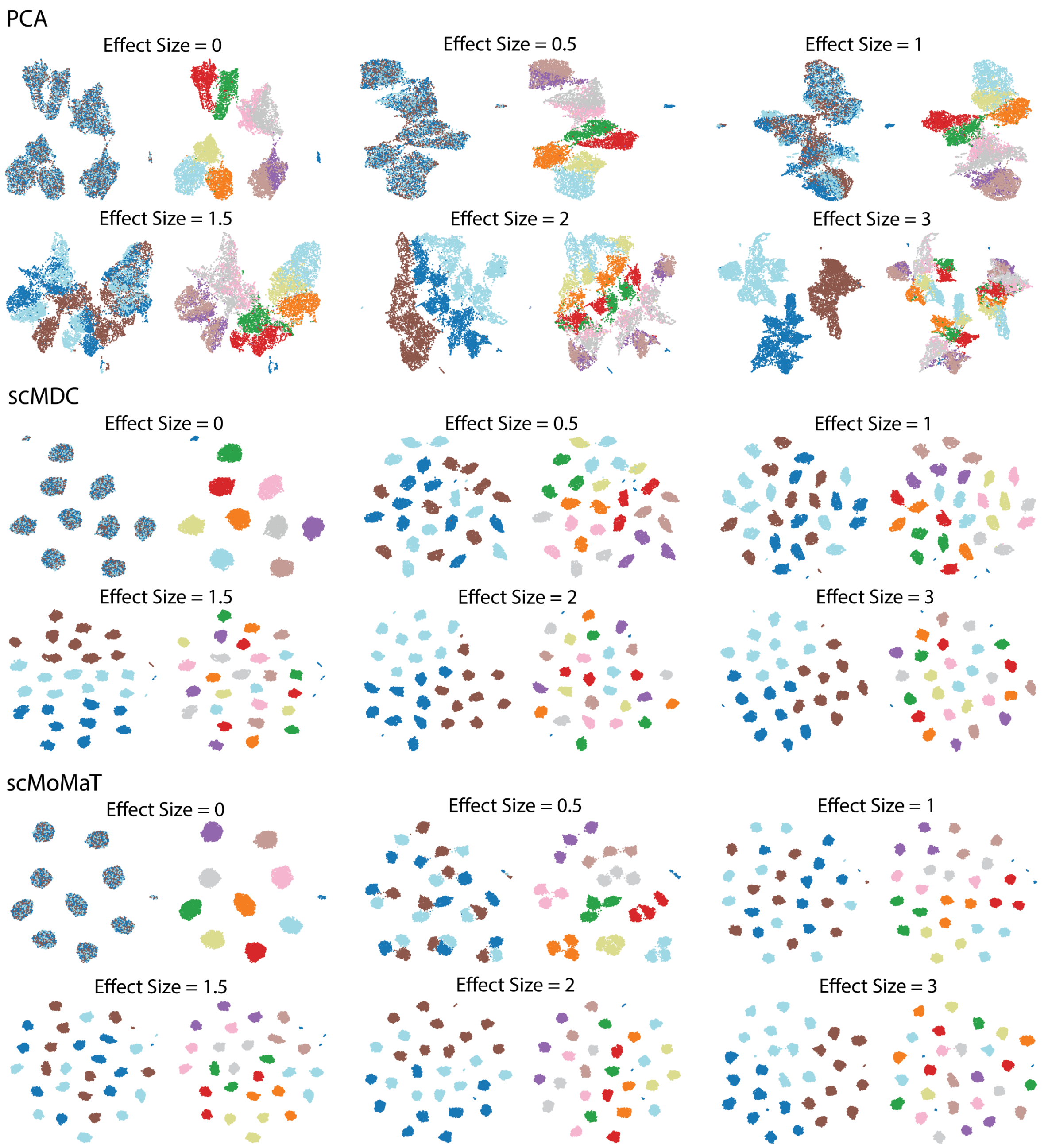

Supplementary Figure 11: **UMAP visualization of three multiomics integration methods (PCA, scMDC, scMoMaT) on six simulated datasets with different levels of batch effects**. The higher the effect size, the larger the batch effects. The left subfigure is colored by batch labels and the right subfigure is colored by cell types.

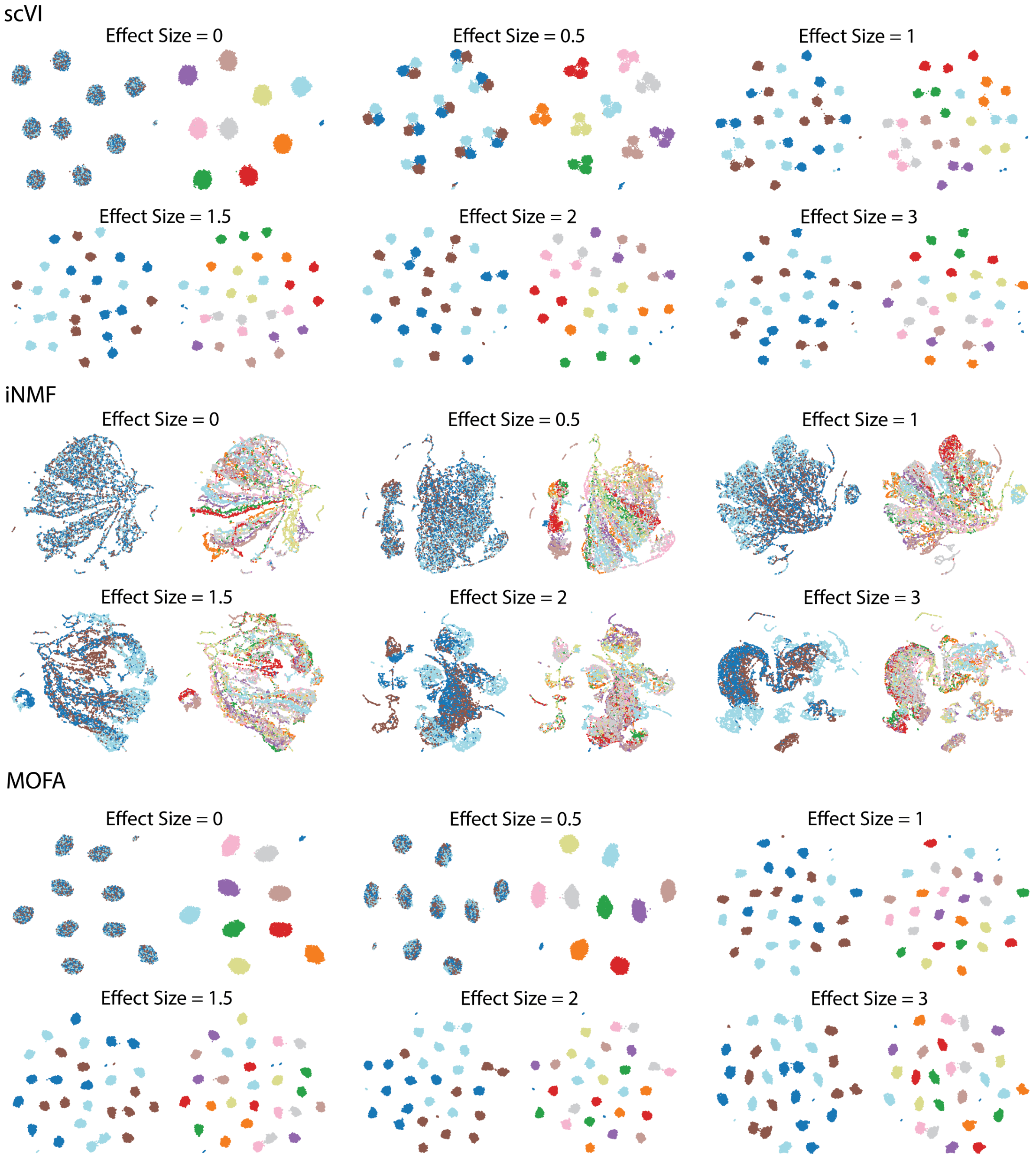

Supplementary Figure 12: **UMAP visualization of three multiomics integration methods (scVI, iNMF, MOFA) on six simulated datasets with different levels of batch effects**. The higher the effect size, the larger the batch effects. The left subfigure is colored by batch labels and the right subfigure is colored by cell types.

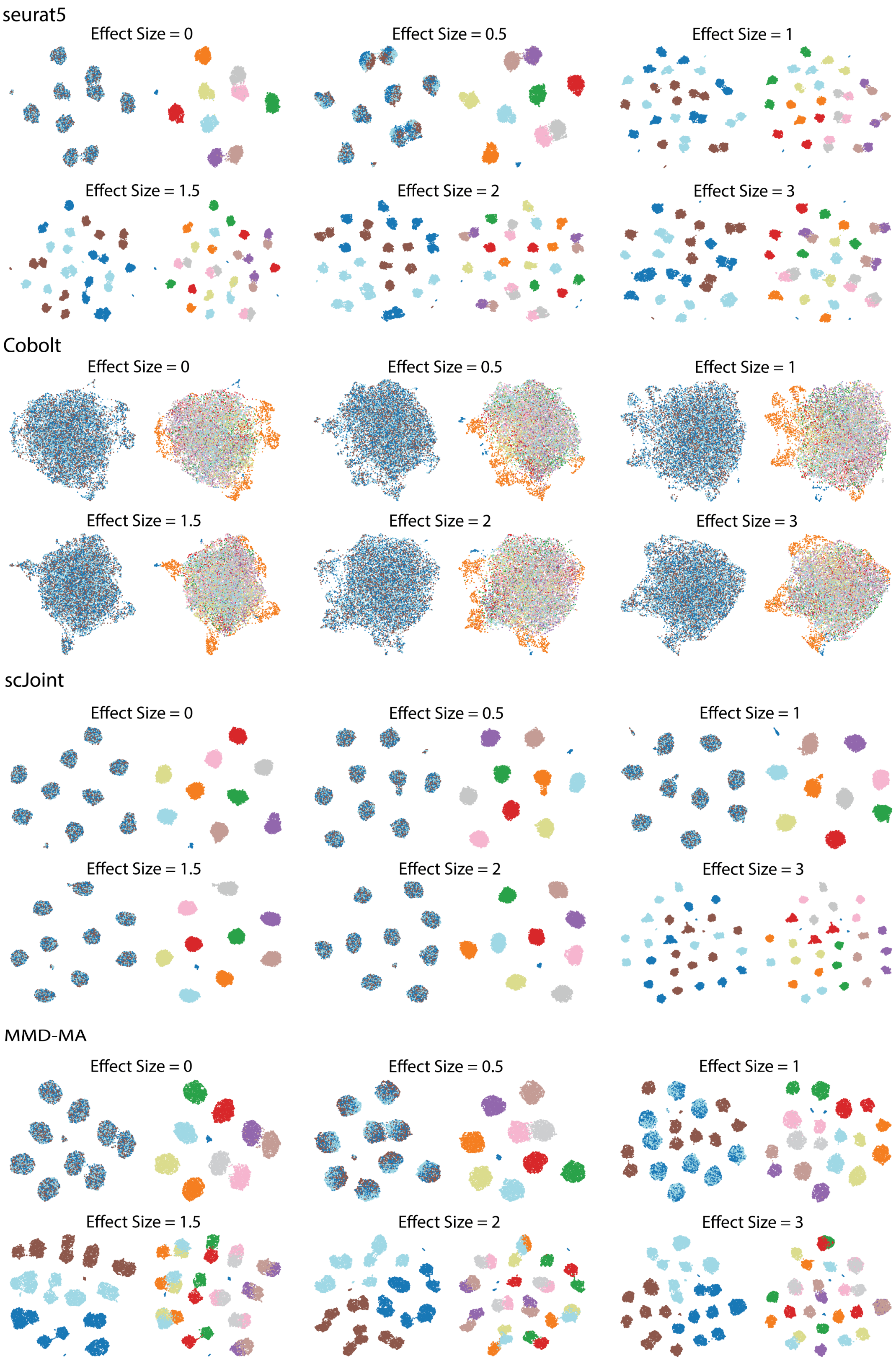

Supplementary Figure 13: **UMAP visualization of three multiomics integration methods (Seurat5, Cobolt, scJoint) on six simulated datasets with different levels of batch effects**. The higher the effect size, the larger the batch effects. The left subfigure is colored by batch labels and the right subfigure is colored by cell types.

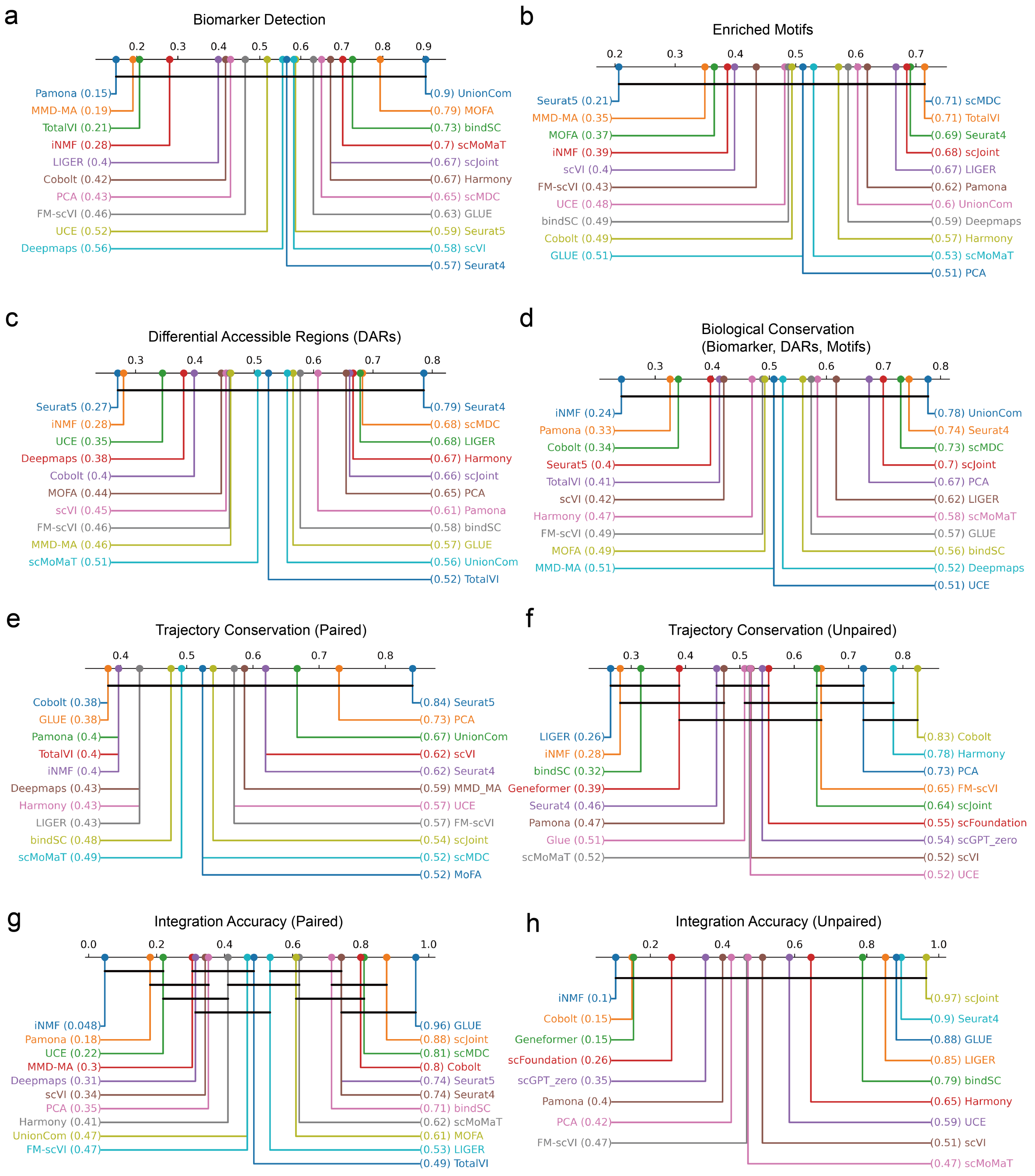

Supplementary Figure 14: **Critical difference diagrams of different metrics.** These diagrams display the average ranks of methods across datasets based on the Friedman test with Conover post-hoc analysis. Methods with lower ranks (positioned more to the right) performed better on average, and horizontal lines connect methods whose performance differences are not statistically significant ($p > 0.05$). Unlike boxplots that show absolute score magnitudes, these diagrams emphasize consistent relative performance across datasets. **a-d.** Critical difference diagrams of (**a**) Biomarker detection, (**b**) Enriched motifs, (**c**) DARs and (**d**) the overall biological conservation scores. **e-f.** Critical difference diagrams of trajectory conservation of paired datasets (**e**) and unpaired datasets (**f**). **g-h**. Critical difference diagrams of integration accuracy of paired datasets (**g**) and unpaired datasets (**h**).

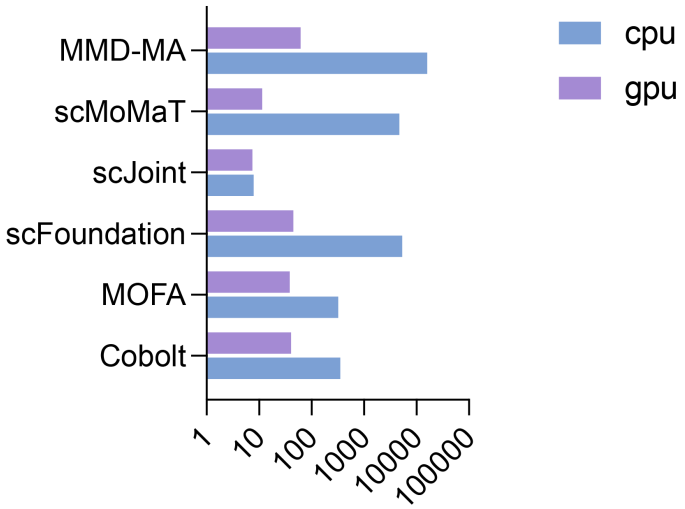

Supplementary Figure 15: **Comparison of running time between CPU and GPU versions.** Five methods applicable to both CPU and GPU versions are included to test the running time (minute).

References

[1] 10X Genomics. PBMC from a healthy donor, single cell multiome ATAC gene expression demonstration data by Cell Ranger ARC 1.0.0 (2020).

[2] Chen, S., Lake, B. B. & Zhang, K. High-throughput sequencing of the transcriptome and chromatin accessibility in the same cell. *Nature biotechnology* **37**, 1452–1457 (2019).

[3] Ma, S. *et al.* Chromatin potential identified by shared single-cell profiling of rna and chromatin. *Cell* **183**, 1103–1116 (2020).

[4] Muto, Y. *et al.* Single cell transcriptional and chromatin accessibility profiling redefine cellular heterogeneity in the adult human kidney. *Nature communications* **12**, 2190 (2021).

[5] Yao, Z. *et al.* A transcriptomic and epigenomic cell atlas of the mouse primary motor cortex. *Nature* **598**, 103–110 (2021).

[6] Saunders, A. *et al.* Molecular diversity and specializations among the cells of the adult mouse brain. *Cell* **174**, 1015–1030 (2018).

[7] Luo, C. *et al.* Single-cell methylomes identify neuronal subtypes and regulatory elements in mammalian cortex. *Science* **357**, 600–604 (2017).

[8] Argelaguet, R. *et al.* Multi-omics factor analysis—a framework for unsupervised integration of multi-omics data sets. *Molecular systems biology* **14**, e8124 (2018).

[9] Liu, J., Huang, Y., Singh, R., Vert, J.-P. & Noble, W. S. Jointly embedding multiple single-cell omics measurements. In *Algorithms in bioinformatics:... International Workshop, WABI..., proceedings. WABI (Workshop)*, vol. 143 (NIH Public Access, 2019).

[10] Stuart, T. *et al.* Comprehensive integration of single-cell data. *Cell* **177**, 1888–1902 (2019).

[11] Korsunsky, I. *et al.* Fast, sensitive and accurate integration of single-cell data with harmony. *Nature methods* **16**, 1289–1296 (2019).

[12] Cao, K., Bai, X., Hong, Y. & Wan, L. Unsupervised topological alignment for single-cell multi-omics integration. *Bioinformatics* **36**, i48–i56 (2020).

[13] Liu, J. *et al.* Jointly defining cell types from multiple single-cell datasets using liger. *Nature protocols* **15**, 3632–3662 (2020).

[14] Gayoso, A. *et al.* Joint probabilistic modeling of single-cell multi-omic data with totalvi. *Nature methods* **18**, 272–282 (2021).

[15] Hao, Y. *et al.* Integrated analysis of multimodal single-cell data. *Cell* **184**, 3573–3587 (2021).

[16] Gao, C. *et al.* Iterative single-cell multi-omic integration using online learning. *Nature biotechnology* **39**, 1000–1007 (2021).

[17] Gong, B., Zhou, Y. & Purdom, E. Cobolt: integrative analysis of multimodal single-cell sequencing data. *Genome biology* **22**, 1–21 (2021).

[18] Lin, X., Tian, T., Wei, Z. & Hakonarson, H. Clustering of single-cell multi-omics data with a multimodal deep learning method. *Nature communications* **13**, 7705 (2022).

[19] Cao, K., Hong, Y. & Wan, L. Manifold alignment for heterogeneous single-cell multi-omics data integration using pamona. *Bioinformatics* **38**, 211–219 (2022).

[20] Dou, J. *et al.* Bi-order multimodal integration of single-cell data. *Genome biology* **23**, 1–25 (2022).

[21] Lin, Y. *et al.* scjoint integrates atlas-scale single-cell rna-seq and atac-seq data with transfer learning. *Nature biotechnology* **40**, 703–710 (2022).

[22] Cao, Z.-J. & Gao, G. Multi-omics single-cell data integration and regulatory inference with graph-linked embedding. *Nature Biotechnology* 1–9 (2022).

[23] Ma, A. *et al.* Single-cell biological network inference using a heterogeneous graph transformer. *Nature Communications* **14**, 964 (2023).

[24] Hao, Y. *et al.* Dictionary learning for integrative, multimodal and scalable single-cell analysis. *Nature Biotechnology* 1–12 (2023).

[25] Zhang, Z. *et al.* scmomat jointly performs single cell mosaic integration and multi-modal bio-marker detection. *Nature Communications* **14**, 384 (2023).

[26] Virshup, I. *et al.* The scverse project provides a computational ecosystem for single-cell omics data analysis. *Nature biotechnology* 1–3 (2023).

[27] Theodoris, C. V. *et al.* Transfer learning enables predictions in network biology. *Nature* **618**, 616–624 (2023).

[28] Rosen, Y. *et al.* Universal cell embeddings: A foundation model for cell biology. *bioRxiv* 2023–11 (2023).

[29] Cui, H. *et al.* scgpt: toward building a foundation model for single-cell multi-omics using generative ai. *Nature Methods* 1–11 (2024).

[30] Hao, M. *et al.* Large-scale foundation model on single-cell transcriptomics. *Nature Methods* 1–11 (2024).

[31] Lee, M. Y., Kaestner, K. H. & Li, M. Benchmarking algorithms for joint integration of unpaired and paired single-cell rna-seq and atac-seq data. *Genome Biology* **24**, 244 (2023).

[32] Xiao, C., Chen, Y., Meng, Q., Wei, L. & Zhang, X. Benchmarking multi-omics integration algorithms across single-cell rna and atac data. *Briefings in Bioinformatics* **25**, bbae095 (2024).
