# Supplementary material for: SCMBench: benchmarking domain-specific and foundation models for single-cell multi-omics data integration
Source: Nat Commun. 2026 May 2;17:5967. doi: 10.1038/s41467-026-72570-x (PMC13342640; doi:10.1038/s41467-026-72570-x)
Supplement: Supplementary file 2 — Description of Additional Supplementary Files [file 41467_2026_72570_MOESM2_ESM.pdf]

Description of Additional Supplementary Files

File Name: Supplementary Data 1
Description: Summary of the 23 benchmarked integration methods (category, model type, unpaired-data support, language, year, reference) and the real datasets used in this study (species, organ, protocol, omics, cell numbers, batch effects, pairing status).

File Name: Supplementary Data 2
Description: Integration accuracy scores (MAP, NMI, ASW, ARI, and overall) for all benchmarked methods across paired, unpaired, and triple-omics datasets, with summary sheets aggregating per-method performance across datasets.

File Name: Supplementary Data 3
Description: Downstream evaluation results for biological conservation, including biomarker detection, differentially accessible regions, enriched transcription factor motifs, trajectory conservation (paired and unpaired), and batch-correction metrics on real multi-batch datasets.

File Name: Supplementary Data 4
Description: Quantitative batch-effect benchmark on simulated paired multi-omics datasets generated by scMultiSim at six batch-effect levels, reporting integration accuracy metrics (MAP, ARI, ASW, NMI, GC) and batch-correction metrics (Batch-ASW, kBET, iLISI) for each method.

File Name: Supplementary Data 5
Description: Computational efficiency measurements (CPU time, peak RAM, peak GPU memory, CPU vs. GPU runtime) for all benchmarked methods, together with the standardized hardware specifications, package versions, and virtual environments used to ensure reproducibility.
