# Supplementary material for: SCMBench: benchmarking domain-specific and foundation models for single-cell multi-omics data integration
Source: Nat Commun. 2026 May 2;17:5967. doi: 10.1038/s41467-026-72570-x (PMC13342640; doi:10.1038/s41467-026-72570-x)
Supplement: Supplementary file 8 — Reporting Summary [file 41467_2026_72570_MOESM8_ESM.pdf]

Corresponding author(s): Yu Li
Last updated by author(s): Mar 16, 2026

Reporting Summary

Nature Portfolio wishes to improve the reproducibility of the work that we publish. This form provides structure for consistency and transparency in reporting. For further information on Nature Portfolio policies, see our Editorial Policies and the Editorial Policy Checklist.

Statistics

For all statistical analyses, confirm that the following items are present in the figure legend, table legend, main text, or Methods section.

n/a	Confirmed	
☐	☒	The exact sample size (*n*) for each experimental group/condition, given as a discrete number and unit of measurement
☐	☒	A statement on whether measurements were taken from distinct samples or whether the same sample was measured repeatedly
☐	☒	The statistical test(s) used AND whether they are one- or two-sided<br>*Only common tests should be described solely by name; describe more complex techniques in the Methods section.*
☒	☐	A description of all covariates tested
☒	☐	A description of any assumptions or corrections, such as tests of normality and adjustment for multiple comparisons
☐	☒	A full description of the statistical parameters including central tendency (e.g. means) or other basic estimates (e.g. regression coefficient) AND variation (e.g. standard deviation) or associated estimates of uncertainty (e.g. confidence intervals)
☐	☒	For null hypothesis testing, the test statistic (e.g. F, t, r) with confidence intervals, effect sizes, degrees of freedom and P value noted<br>*Give P values as exact values whenever suitable.*
☒	☐	For Bayesian analysis, information on the choice of priors and Markov chain Monte Carlo settings
☒	☐	For hierarchical and complex designs, identification of the appropriate level for tests and full reporting of outcomes
☒	☐	Estimates of effect sizes (e.g. Cohen's *d*, Pearson's *r*), indicating how they were calculated

Our web collection on statistics for biologists contains articles on many of the points above.

Software and code

Policy information about availability of computer code

Data collection	No software was used to collect data. All datasets used in this study are publicly available and were downloaded from their respective repositories (10X Genomics, GEO, BICCN NeMO archive). Simulated datasets were generated using scMultiSim (v1.0).
Data analysis	Python (v3.8), R (v4.1). Integration methods and versions: GLUE (0.3.2), scVI/TotalVI (scvi-tools 1.2.2), scMoMaT (0.2.2), LIGER (rliger 2.1.0), MOFA (0.7.2), bindSC (1.0.0), Seurat4/Seurat5 (5.2.0), scMDC (1.0.1), Online-iNMF (rliger 2.1.0), DeepMAPS (1.0), Cobolt (0.0.1), Pamona (0.1.0), scJoint, Harmony (0.0.10), scGPT, Geneformer (0.1.0), scFoundation, UCE. Preprocessing: Scanpy (v1.9), MAESTRO, BABEL. Evaluation: scikit-learn (0.22.1), GimmeMotifs. Visualization: matplotlib, seaborn. Custom benchmarking code is available at https://github.com/ml4bio/SCMBench (DOI: 10.5281/zenodo.19050984).

For manuscripts utilizing custom algorithms or software that are central to the research but not yet described in published literature, software must be made available to editors and reviewers. We strongly encourage code deposition in a community repository (e.g. GitHub). See the Nature Portfolio guidelines for submitting code & software for further information.

Data

Policy information about availability of data

All manuscripts must include a data availability statement. This statement should provide the following information, where applicable:

- Accession codes, unique identifiers, or web links for publicly available datasets
- A description of any restrictions on data availability
- For clinical datasets or third party data, please ensure that the statement adheres to our policy

All datasets used in this study are publicly available. The PBMC-10x dataset is available from 10X Genomics (https://www.10xgenomics.com/datasets/pbmc-from-a-healthy-donor-granulocytes-removed-through-cell-sorting-10-k-1-standard-1-0-0). The Chen-2019 dataset is available from GEO under accession code GSE126074 (https://www.ncbi.nlm.nih.gov/geo/query/acc.cgi?acc=GSE126074). The Ma-2020 dataset is available from GEO under accession code GSE140203 (https://www.ncbi.nlm.nih.gov/geo/query/acc.cgi?acc=GSE140203). The Muto-2021 dataset is available from GEO under accession code GSE151302 (https://www.ncbi.nlm.nih.gov/geo/query/acc.cgi?acc=GSE151302). The Yao-2021 dataset is available from the BICCN NeMO archive (https://assets.nemoarchive.org/dat-ch1nqb7). The Triple dataset combines data from Saunders et al., available from GEO under accession code GSE116470 (https://www.ncbi.nlm.nih.gov/geo/query/acc.cgi?acc=GSE116470), and Luo et al., available from GEO under accession code GSE97179 (https://www.ncbi.nlm.nih.gov/geo/query/acc.cgi?acc=GSE97179). Simulated datasets were generated using scMultiSim as described in Methods. Source data are provided with this paper.

Research involving human participants, their data, or biological material

Policy information about studies with human participants or human data. See also policy information about sex, gender (identity/presentation), and sexual orientation and race, ethnicity and racism.

Reporting on sex and gender	This study is a computational benchmarking study using publicly available single-cell sequencing datasets. Sex and gender were not considered in the study design as the analyses focus on method performance evaluation rather than biological differences between sexes.
Reporting on race, ethnicity, or other socially relevant groupings	Not applicable. This study does not involve socially constructed or socially relevant categorization variables. All analyses are based on cell-type annotations from publicly available datasets.
Population characteristics	Not applicable. This is a computational benchmarking study. No human research participants were recruited.
Recruitment	Not applicable. No participants were recruited. All datasets used are publicly available.
Ethics oversight	Not applicable. This study is a computational analysis of publicly available datasets and does not require ethics approval.

Note that full information on the approval of the study protocol must also be provided in the manuscript.

Field-specific reporting

Please select the one below that is the best fit for your research. If you are not sure, read the appropriate sections before making your selection.

☒ Life sciences ☐ Behavioural & social sciences ☐ Ecological, evolutionary & environmental sciences

For a reference copy of the document with all sections, see nature.com/documents/nr-reporting-summary-flat.pdf

Life sciences study design

All studies must disclose on these points even when the disclosure is negative.

Sample size	We use all samples in the datasets to benchmark the performance. To minimize the excessive influence of batch effects on the exploration of integration accuracy and biomarker detection, we partitioned the Ma-2020 and Muto-2021 datasets, which inherently contain batch effects. The Ma-2020 dataset was divided into four batches containing 5,692, 10,700, 9,903, and 5,927 cells, respectively, while the Muto-2021 dataset was split into five batches with 3,683, 5,464, 3,804, 4,114, and 2,920 cells. To ensure that a wide range of methods could successfully operate across different datasets, we sampled the larger Ma-2020 and Muto-2021 datasets to maintain 2,500 cells per batch. This resulted in the combined sampled datasets, Ma-2020-sampled and Muto-2021-sampled, which were used for experiments investigating batch effects. The original Ma-2020 and Muto-2021 datasets were employed to test batch effects under imbalanced conditions. Triple dataset contains 55803 scRNA-seq cells, 2317 scATAC-seq cells and 3377 snmC-seq cells.
Data exclusions	All data were used following previous researches , no exclusion was done prior to analysis.
Replication	The performance of our model could be reproduced, and we also offer codes, on-line service, and tutorials for the key downstream tasks.
Randomization	We apply use different seed to initialize model's weight before training, and we can get consistent results for different seeds.
Blinding	No, blinding is not applicable in our experiments. We did not have any experiments with different conditions.

Reporting for specific materials, systems and methods

We require information from authors about some types of materials, experimental systems and methods used in many studies. Here, indicate whether each material, system or method listed is relevant to your study. If you are not sure if a list item applies to your research, read the appropriate section before selecting a response.

Materials & experimental systems

n/a	Involved in the study
☒	☐ Antibodies
☒	☐ Eukaryotic cell lines
☒	☐ Palaeontology and archaeology
☒	☐ Animals and other organisms
☒	☐ Clinical data
☒	☐ Dual use research of concern
☒	☐ Plants

Methods

n/a	Involved in the study
☒	☐ ChIP-seq
☒	☐ Flow cytometry
☒	☐ MRI-based neuroimaging

Plants

Seed stocks	Not applicable.
Novel plant genotypes	Not applicable.
Authentication	Not applicable.
