# Supplementary material for: SCMBench: benchmarking domain-specific and foundation models for single-cell multi-omics data integration
Source: Nat Commun. 2026 May 2;17:5967. doi: 10.1038/s41467-026-72570-x (PMC13342640; doi:10.1038/s41467-026-72570-x)
Supplement: Supplementary file 9 — Transparent Peer Review file [file 41467_2026_72570_MOESM9_ESM.pdf]

nature portfolio

Peer Review File

SCMBench: Benchmarking Domain-specific and Foundation Models for Single-cell Multi-omics Data Integration

Corresponding Author: Professor Yu Li

Parts of this Peer Review File have been redacted as indicated to remove third-party material.

This file contains all reviewer reports in order by version, followed by all author rebuttals in order by version.

Version 0:

Reviewer comments:

Reviewer #1

(Remarks to the Author)
The main focus of this manuscript is the benchmarking of foundation models for single-cell multi-omics integration, compared with domain-specific models. In addition to integration, the authors also investigate the models' abilities in downstream analysis tasks, such as biomarker detection, trajectory inference, and batch effect correction. The authors address the gap in assessing foundation models compared to domain-specific models and present an approach to improve the effectiveness of foundation models in integration through a case study. While this work makes a valuable contribution to single-cell multi-omics research, particularly through its focus on foundation model comparison, the paper would benefit from more clarity in its structure and depth in interpretation. The two main weaknesses are the inclusion of excessive technical details in the main manuscript, which would be better suited for the Methods section, and a lack of depth in the interpretation of the key results. Here are our comments:

Major comments:
1) The results/discussion is quite descriptive at this stage and the implication of the benchmarking results is not clear. How can one benefit from this extensive study and what recommendation and insight for scientist? The discussion section can be substantially expand.

2) Figure 2a is confusing to readers. The matrices above the diagram show the names of two datasets (PBMC-10x and Ma-2020). It appears that PBMC-10x is used for the integration and bio-conservation tasks, while Ma-2020 is used for the batch correction task and computational efficiency evaluation. However, in the main manuscript, Figure 2a is described as being based only on PBMC-10x. Please clarify the information presented in this panel.

3) While the study includes five real datasets and multiple batches, the total number of paired and unpaired datasets is six each, which is relatively small. The methods' performance in Figures 2d and 2e may be influenced by the integration performance of a single dataset. Although the authors mentioned that the datasets were carefully selected based on criteria of representativeness and popularity, the limited number of datasets may reduce the generalizability of the results. It would be better to discuss this potential limitation of the benchmark study in the Discussion section.

4) When assessing biomarker detection consistency and trajectory alignment, the authors use both boxplots and critical difference diagrams to show the methods' performance by rank. However, we noticed that the two types of graphs are quite confusing. For example, in Figure 3b, Seurat5 is shown as the top-performing method, but in Figure 3c, it is ranked in the middle. What is the difference between the terms "overall marker detection" and "cell type-specific marker detection"? Further clarification is required to explain why the performance in these two scenarios differs so significantly. Similar issues are observed in Figures 4b and 4c, where scGPT_zero is ranked fourth in the critical difference diagram but appears in the middle of the boxplot.

5) Although critical difference diagrams are included, the interpretation of statistically significant or insignificant differences between methods could be better addressed in the manuscript. Is there any potential meaning behind the statistical insignificance observed among the top-performing methods? For example, Figure 3C, MOFA and GLUE, Figure 4C, Cobolt and PCA, PCA and scJoint.

6) Page 9, please provide more detailed explaination on the motivation/logic behind how moderate Jaccard indices can act as a metric to determine how each of the methods are valuable for exploring novel or cell-type-specific biomarkers.

7) Page 12, Section 2.5 paragraph 2, detailed explanations of potential reasons are required on the trade-offs between batch effect correction and integration accuracy).

Minor issues:
1)Include the specific version of each method involved in this benchmarking study, as performance may change due to implementation upgrades.
2)MoFA should be written as MOFA, refer to the correct capitalisation as used in the original paper.
3)Page 7, in the last paragraph, it is stated that the definition of the Biomarkers Detection score can be found in Section 4.2.2. However, no explicit definition is provided in Section 4.2.2, the score is only briefly mentioned in the last paragraph of page 7. To improve clarity, the manuscript should either include the full definition in Section 4.2.2 or explicitly reference where the definition is located.
4)Page 10, paragraph 2, line 3, the authors mention that MOFA ranks first with a TC score of 0.9307. However, in Figure 4a, MOFA's TC score for the pivotal cell types is shown as 0.8273, indicating a performance drop. Does this also occur with other methods? What could be the potential reasons for this discrepancy? This could provide a new angle for the benchmarking study—whether the performance of the methods differs between pivotal cell types and minor cell types.
5)Page 14, paragraph 2, the term "scFMs" seems to be a typo, should it be "FMs." Please verify and revise accordingly.
6)Page 16, Section 2.7, paragraph 1, Yao-2021 is described as including 69,727 samples and 54,844 samples, better to refer this as “cells”. Please ensure consistent terminology is used throughout the manuscript.

(Remarks on code availability)
This is a benchmarking paper, and the authors have shared their benchmarking code on GitHub. I went through the public repository, the documentation is clear, but it doesn’t include detailed instructions on how to install all the required packages. The 'Directory structure' section in the main README mentions an environment YAML file, but I couldn’t find it in the repository. Since I’m already familiar with these packages, I was able to install them one by one. However, for someone new to this field, setting up and running the code for the first time might be a challenge. Overall, the repository is a great resource for the community, but it would be much better if it included clear package installation instructions and an accessible environment file.

Reviewer #2

(Remarks to the Author)
I co-reviewed this manuscript with one of the reviewers who provided the listed reports. This is part of the Nature Communications initiative to facilitate training in peer review and to provide appropriate recognition for Early Career Researchers who co-review manuscripts.

(Remarks on code availability)

Reviewer #3

(Remarks to the Author)
The manuscript, "SCMBench: Benchmarking Domain-specific and Foundation Models for Single-cell Multi-omics Data Integration," presents a thorough evaluation of 22 integration methods (DMs and FMs) using diverse datasets and metrics, including integration accuracy, biomarker detection, trajectory inference, and batch effect correction. It demonstrated the limitations of FMs, which often underperform DMs and proposed a "FM + Adaptation Layer" approach to improve FM performance. However, I found several significant issues regarding the leverage, evaluation, and adaptation of single-cell foundation models (scFMs) in this benchmarking study, which diminish the paper's credibility and potential impact on the scFMs community.

1. A primary concern lies in the leverage of scFMs on scATAC data, as these models are originally designed and pretrained on scRNA-seq data only. The authors stated that “although scGPT is the only specialized FM for multi-omics integration, other FMs can also be adapted for this purpose by analyzing cell embeddings.” However, neither the manuscript nor the GitHub repository provides sufficient technical details or a clear rationale for adapting scFMs to handle scATAC data. The manuscript describes the approach as “Due to the limitations imposed by the fixed tokens of the pre-trained large model, we are unable to directly input the peak features of single-cell ATAC-seq data. Instead, we employ a strategy similar to those used in Deepmaps and scMDC: mapping scATAC-seq data onto scRNA-seq data. This involves assigning regulatory activity scores or weights to scRNA-seq cells based on the accessibility or openness of regulatory elements observed in the scATAC-seq data. This process allows us to obtain the corresponding activity matrix, which subsequently serves as the input for the FMs.” But it is unclear how this activity matrix is calculated or whether its data distribution, signal-to-noise ratio, and biological relevance align with those of RNA expression. Without further justification, this leverage appears ad hoc, and the meaningfulness and fairness of benchmarking scFMs in this manner, particularly in the zero-shot setting, are doubtful. The underperformance of scFMs on diverse tasks further suggests that such an approach does not fully realize their potential. The study also fine-tuned scGP with scATAC data and claimed that “This performance discrepancy may be

attributed to the possibility that the fine-tuning process for scGPT inadvertently mixes DNA-related information with RNA-related information, potentially disrupting the specific RNA-related details crucial for biomarker detection." This explanation further confirms the incompatibility of the proposed usage of scFMs with scATAC data. Besides, the provided GitHub repository lacks critical scripts and documentation for replicating the scFMs setup described in the study. The repository includes only the official scRNA-seq interface for zero-shot scGPT/UCE/Geneformer, and the fine-tuning script focuses on integrating RNA and protein expression, not RNA and ATAC. No script for scFoundation is provided, and several links in the README file lead to inaccessible pages. This lack of accessible and complete source code significantly hinders the reproducibility and assessment of the study's claims.

2. The authors proposed an adaptation method for scFMs to integrate scRNA and scATAC data, which involved leveraging scVI's multi-omics integration functionality to fuse embeddings derived from zero-shot scFMs (scGPT and UCE were tested). This approach, termed "scFMs+Adaptation layers," was validated on two datasets by comparing the harmonized embeddings from scVI with those generated by various scFM configurations, including zero-shot scGPT/UCE, partially fine-tuned scGPT, and fully retrained scGPT. The study concluded that the joint embeddings from scVI outperformed those from the mechanically applied scFMs. However, several issues remain: i) The fine-tuning strategy in this comparison updated only the top-4 layers of scFMs, differing from the official full fine-tuning approach. The rationale for selecting these specific layers and ensuring their compatibility with the remaining frozen layers is not justified, raising concerns about the robustness of the proposed method. ii) Again, the source code for these "scFM adaptations" is incomplete. The provided GitHub repository includes only a script for scVI-based integration using precomputed RNA and ATAC embeddings, without details of their embedding calculations. iii) If the "scFMs+Adaptation layers" approach represents an optimal integration strategy for scRNA and scATAC using scFMs, it should have been included as part of the benchmarking study rather than presented as a proof-of-concept in Section 2.6.

3.In Figure 2, the authors unexpectedly applied the human data-pretrained scGPT to mouse datasets and concluded that such scFMs lack cross-species transferability. This conclusion is obvious. However, the study fails to provide critical details on how mouse data was input into scGPT, considering the gene set gap between humans and mice. Specifically, scGPT uses a human gene vocabulary, which inherently excludes genes unique to other species. How did the authors ensure the accurate and complete alignment of mouse RNA and ATAC data with scGPT's human gene vocabulary?

4. The biomarker discovery benchmarking in this study relies on the top-ranked genes identified by the Scanpy package's Differentially Expressed Gene (DEG) analysis. These genes, derived from the predicted cell types based on cell embeddings generated by different methods, were used to assess the consistency and differentiation of cell-type-associated genes. However, this approach fundamentally evaluates cell type identification performance rather than true biomarker discovery. DEG-based top genes are merely biomarker candidates, as DEG analysis is known to be sensitive to statistical noise, exhibit limited biological relevance, and ignore gene-gene dependencies. Treating DEG outcomes as a biomarker ground truth overlooks the possibility that scFMs might mitigate these limitations, thereby potentially underestimating their true performance in biomarker discovery.

Given the concerns outlined above, I hardly support the publication of this study in its current form.

(Remarks on code availability)

Reviewer #4

(Remarks to the Author)
Review of "SCMBench: Benchmarking Domain-specific and Foundation Models for Single-cell Multi-omics Data Integration"

This paper presents a comprehensive benchmark study, SCMBench, for evaluating the performance of various integration methods on single-cell multi-omics data. The study compares 22 popular methods, including both statistical-based and deep learning-based approaches, as well as four foundation models (FMs). The benchmark applied the framework established in a previous study by Malte et al. [1], thereby limiting the novelty of the benchmark. The major weakness of the benchmark is that it mostly focuses on the scRNA-seq-related analysis tasks while barely comparing performance on the task to another modality (scATAC-seq). The most significant contribution of the paper is to provide an assessment of FMs in the context of single-cell multi-omics data integration. It demonstrates the current state of FMs in the single-cell bioinformatics community. The abstract indicates that there is still a performance gap between FMs and current mainstream pipelines known as Domain-specific Models (DMs). Therefore, it is difficult to draw a conclusion for the recommendation of FMs as they require intensive downstream fine-tuning. Besides, some of the benchmarks are not well aligned with the use case of the bioinformatics analysis. There are several other issues that the paper needs to address, and the detailed comments are outlined below:

Major comments:
1. The primary weakness of the paper lies in its focus on the "Single-cell Multi-omics Data Integration" setup, where most metrics tested are derived at the transcriptomic level (e.g. trajectory and biomarker score), or cell label (e.g. NMI, ARI) with almost no discussion of performances on other modalities. Furthermore, the paper overlooks additional single-cell data modalities like DNA methylation and chromosome conformation capture (3C) data. Focusing solely on applying metrics to scRNA-seq data is acceptable, considering that previous research primarily evaluated integration methods for transcriptomic data. [1]. In establishing a benchmark setup for multi-omics integration, the author should design modality-specific

assessments. For example, examining biological conservation metrics like the conservation of differentially accessible regions, motif enrichments, and peak calling results at the scATAC-seq level.

2. As mentioned in the comment 1. Testing the performance of integrating data for more modalities such as single-cell methylation is preferred.

3. The benchmark of the Trajectory Conservation (TC) on a PBMC dataset is questionable. The author should test the TC on a dataset that focuses on tracing cell lineages or for longitudinal scRNA-seq study. For instance, the benchmark paper which the author closely follows tested the TC on data studying erythrocytes and their progenitors [1]. Studying the pseudo-time on the PBMC data alone does not reveal significant biological insights, because the root cell type is set manually to be HSPCs. Besides, the author should add annotation on each of the cell clusters in Figure 4 to indicate cell types upon the trajectory.

4. The computation efficiency test is too brief. The author only tested the running time. Is it wall clock time or CPU time? What about their RAM/GPU VRAM consumption? Does each of the methods require GPU? What is the difference in the running time between the GPU version and the CPU version? What is your testing environment and hardware spec?

5. The recommendation of the FM applications seems to be struggling in the paper. The author states “Generally, we find FMs fall short of state-of-the-art DMs in this field.” Besides, there is a need to apply adaptation learning or further fine-tuning for FMs in single-cell tasks. However, the discussion notes that “FMs are crucial tools in single-cell research due to their versatility and strong generalization abilities”. In my opinion, the requirement of adaptation learning (rather than simple prompt engineering) has already contradicted the conclusion of the generalizability of FMs. Downstream learning and fine-tuning also require additional computational power while large models introduce the risk of underfitting on small datasets. Combining the unsatisfactory performance and all the concerns, is the author still confident to conclude that the paper can “unlock the full potential of FMs and promote their effective application in the single-cell multi-omics integration”? Please comment.

Minor comments:
1. The preprocessing methods including the application of pipeline, quality control metrics, and hyper-parameters applied for scRNA-seq and scATAC-seq are unclear. The author should justify them in the method section or the supplementary data

2. Harmony is one of the mainstream integration tools [2]. It has already been integrated into popular packages such as scanpy [3] and Seurat [4]. It is better to benchmark the performance of harmony.

Reference:
[1] M. D. Luecken et al., “Benchmarking atlas-level data integration in single-cell genomics,” Nat. Methods, vol. 19, no. 1, pp. 41–50, Jan. 2022, doi: 10.1038/s41592-021-01336-8.
[2] I. Korsunsky et al., “Fast, sensitive and accurate integration of single-cell data with Harmony,” Nat. Methods, vol. 16, no. 12, pp. 1289–1296, Dec. 2019, doi: 10.1038/s41592-019-0619-0.
[3] F. A. Wolf, P. Angerer, and F. J. Theis, “SCANPY: large-scale single-cell gene expression data analysis,” Genome Biol., vol. 19, no. 1, pp. 1–5, 2018.
[4] R. Satija, J. A. Farrell, D. Gennert, A. F. Schier, and A. Regev, “Spatial reconstruction of single-cell gene expression data,” Nat. Biotechnol., vol. 33, no. 5, pp. 495–502, 2015.

(Remarks on code availability)

Version 1:

Reviewer comments:

Reviewer #1

(Remarks to the Author)
No further comments. The authors have done a nice job addressing all of my comments. The changes has improve the clarity of the paper. I note that the added installation guides and setup scripts that is now accompanying the code. I believe this will make the code much more accessible and user-friendly. Congratulation on a nice manuscript.

(Remarks on code availability)
The code was assess by another ECR reviewer

Reviewer #2

(Remarks to the Author)
I co-reviewed this manuscript with one of the reviewers who provided the listed reports. This is part of the Nature Communications initiative to facilitate training in peer review and to provide appropriate recognition for Early Career

Researchers who co-review manuscripts.

(Remarks on code availability)
The newly added installation instructions and setup scripts are clear and greatly improve the usability of the repository. Thanks.

Reviewer #3

(Remarks to the Author)
I appreciate the authors' thoughtful revision and their partial resolution of my initial concerns. Here are my further comments on this revision:
1. I would not be a deal breaker, but I remain unconvinced by the way the authors leverage scFMs for integrating scATAC and scRNA data. While the authors justify this by claiming it represents the "maximum level of effort feasible for non-specialist users," this approach is clearly outside the intended scope of current scFMs, which are generally trained on scRNA data alone. Feeding scATAC data directly into these models risks severe out-of-distribution effects and undermines the integrity of the benchmarking. It is misleading to treat such usage as representative or valid for model comparison. At a minimum, the authors should explicitly state that this is not the official or recommended use of the models in question. They should also acknowledge and discuss alternative strategies, such as using scATAC-specific FMs (e.g., GET), projecting scATAC data to a shared latent space, or translating scATAC into scRNA-like profiles using methods like BABEL. Ignoring these possibilities gives an incomplete and potentially misleading picture of model performance.

2. The terminology used throughout the manuscript is also problematic. For example, referring to model variants like scGPT-scVI and UCE-scVI as "FM-scVI" is overly broad and implies generality that does not exist. Given the number of foundation models developed in the field, including scBERT, scFoundation, and others. It is inappropriate to use such generic terms for a limited subset of models. Similarly, the phrase "FM-adaptation" is used to refer to a specific combination of scFM and scVI, yet the literature contains a variety of adaptation strategies such as full fine-tuning, partial fine-tuning, and parameter-efficient tuning. The authors' implementation reflects just one of many possible approaches and should be named accordingly. The current labeling overstates the scope and risks confusing readers.

3. On page 6, the authors state that "architecture itself plays a crucial role in multi-omic integration," but this statement is vague and unsupported. It is unclear whether the authors are suggesting that untrained model architectures can integrate modalities effectively, or that pretraining contributes little to integration performance. Either way, this claim requires further justification and possibly empirical support. Without clarification, the statement feels speculative and unsubstantiated.

4. On page 8, the authors note that scFoundation is surprisingly faster than smaller models. This is counterintuitive and should be explained.

5. There is also a structural issue with the organization of the manuscript. The proposed adaptation framework is only explained in Section 2.6 and Figure 6, yet it is invoked much earlier in the Results section. This disrupts the logical flow and makes it difficult for readers to follow the methodology. The explanation of the adaptation approach should be introduced earlier, before its application is described.

6. I was surprised to find that some AI-generated text appears in the manuscript. For instance, "Retry Claude can make mistakes. Please double-check responses." on pages 13 and 16. Such artifacts should have been removed during basic proofreading.

(Remarks on code availability)

Reviewer #4

(Remarks to the Author)
The authors have thoughtfully addressed the majority of concerns raised in my initial review. The revised manuscript now offers a significantly more comprehensive evaluation of multi-omics integration methods, with notable improvements in the assessment of scATAC-seq-related metrics. Key enhancements include:

1. Epigenomic evaluation through differential accessibility regions (DARs) and motif enrichment analysis, providing crucial validation beyond transcriptomic assessments.
2. Improved scATAC-seq processing with a detailed demonstration of gene activity matrix pre-processing for foundation models, directly addressing prior reviewer concerns.
3. More reasonable claims regarding foundation model (FM) performance, now including a discussion of their trade-offs.

The authors have successfully resolved the major issues raised by Reviewer #4, resulting in a more rigorous, reproducible, and biologically insightful benchmark.

Remaining Question:
Why were foundation models (FMs) excluded from the triple-omics integration benchmark?

(Remarks on code availability)
The repository appears to be well-maintained, featuring a comprehensive README document that provides clear guidance. While I have reviewed the relevant code sections related to my review comments via the online repository, I did not perform a local installation or execute the code. As such, I cannot verify or guarantee the functional correctness of the implementation.

Version 2:

Reviewer comments:

Reviewer #3

(Remarks to the Author)
The revision fully addresses my concerns and significantly improves the manuscript’s clarity, rigor, and overall readability. I have no further comments.

(Remarks on code availability)

Open Access This Peer Review File is licensed under a Creative Commons Attribution 4.0 International License, which permits use, sharing, adaptation, distribution and reproduction in any medium or format, as long as you give appropriate credit to the original author(s) and the source, provide a link to the Creative Commons license, and indicate if changes were made.
In cases where reviewers are anonymous, credit should be given to 'Anonymous Referee' and the source.
The images or other third party material in this Peer Review File are included in the article’s Creative Commons license, unless indicated otherwise in a credit line to the material. If material is not included in the article’s Creative Commons license and your intended use is not permitted by statutory regulation or exceeds the permitted use, you will need to obtain permission directly from the copyright holder.
To view a copy of this license, visit https://creativecommons.org/licenses/by/4.0/

Response to reviewers

Manuscript ID: NCOMMS-24-71130-T

Manuscript title: SCMBench: Benchmarking Domain-specific and Foundation Models for Single-cell Multi-omics Data Integration

We would like to express our sincere gratitude to all reviewers for their constructive feedback and valuable suggestions. Your thoughtful comments have significantly improved the quality and clarity of our manuscript. We have carefully addressed all concerns by conducting additional experiments, enhancing our analyses, and revising the manuscript accordingly.

We would like to summarize the key additions and revisions to the paper:

1. **Expanded and reorganized Discussion section**: We've restructured our Discussion with dedicated subsections on performance trade-offs, foundation models, and limitations, providing deeper interpretation of our findings and their implications.
2. **Improved figure clarity and statistical analysis**: We've enhanced figure captions and annotations while moving Critical Difference diagrams to Supplementary Materials, providing comprehensive interpretation of statistical results and their implications for method selection.
3. **Enhanced biomarker detection evaluation**: We've incorporated reference-based validation using ground truth markers from the CellMarker 2.0 database and included concrete examples of biologically relevant markers uniquely identified by methods with moderate Jaccard indices but missed by high-consensus approaches.
4. **Expanded epigenomic assessment**: We've introduced comprehensive scATAC-seq evaluation with two modality-specific features:
    - Differentially Accessible Regions (DARs) identification
    - Transcription factor binding motif enrichment analysis using GimmeMotifs
5. **Extended trajectory conservation analysis**: We've added evaluations on additional biologically meaningful systems:
    - Human B cell lineage in PBMCs – from HSPCs to plasma cells
    - Neuronal differentiation in mouse cortex
    - Analyses distinguishing between major and minor cell types
6. **Included triple-omics integration benchmark**: We've added single-cell RNA-seq, ATAC-seq, and DNA methylation data from mouse cortex tissue to evaluate performance on more complex multi-modal integration scenarios.
7. **Added Harmony evaluation**: Following reviewers' suggestions, we've included Harmony in our benchmarking suite.
8. **Improved foundation model assessment and adaptation**: We've:

- Provided clear rationales for our layer selection approach
- Fully integrated our FM-scVI adaptation across all metrics
- Updated our GitHub repository with comprehensive documentation
- Addressed cross-species application concerns by removing problematic direct comparisons
- More precisely described foundation models' capabilities and limitations

9. **Enhanced reproducibility and transparency**: We've provided:
    - Software versions table for all methods
    - Detailed descriptions of gene activity matrix calculation methodology
    - Comprehensive environment configurations and installation instructions
    - Hardware specifications and computational benchmarking metrics
    - Enhanced preprocessing documentation with all scripts available on GitHub
10. **Clarified terminology and improved overall clarity**: We've addressed unclear or imprecise terminology throughout the manuscript to enhance readability.

Please find below for the point-to-point response to all the three reviewers' comments.

Blue: our response to the reviewer's comments<br><br>Black: reviewer's comments	Orange: reference to the manuscript<br><br>Green: Revised sentences

Reviewer #1 (Remarks to the Author):

The main focus of this manuscript is the benchmarking of foundation models for single-cell multi-omics integration, compared with domain-specific models. In addition to integration, the authors also investigate the models' abilities in downstream analysis tasks, such as biomarker detection, trajectory inference, and batch effect correction. The authors address the gap in assessing foundation models compared to domain-specific models and present an approach to improve the effectiveness of foundation models in integration through a case study. While this work makes a valuable contribution to single-cell multi-omics research, particularly through its focus on foundation model comparison, the paper would benefit from more clarity in its structure and depth in interpretation. The two main weaknesses are the inclusion of excessive technical details in the main manuscript, which would be better suited for the Methods section, and a lack of depth in the interpretation of the key results. Here are our comments:

Response: We greatly appreciate your thorough and insightful review of our manuscript on benchmarking foundation models for single-cell multi-omics integration. Your comments have significantly helped us improve both the clarity and depth of our paper. We have addressed your concerns through several substantive changes:

1. **Expanded and reorganized Discussion section**: We've restructured our Discussion with dedicated subsections on performance trade-offs, foundation models, and limitations, providing deeper interpretation of our findings and their implications.
2. **Improved figure clarity and descriptions**: We've enhanced figure captions and annotations to make the results more accessible and interpretable, with clearer explanations of measurements and interpretation guidelines.
3. **Reorganized statistical visualizations**: We've moved the Critical Difference diagrams to Supplementary Materials to improve the main text flow, while providing comprehensive interpretation of these statistical analyses for method selection.
4. **Added specific biological examples**: We've included concrete examples of biologically relevant markers uniquely identified by methods with moderate Jaccard indices but missed by high-consensus approaches to substantiate our claims.
5. **Enhanced Methods section**: We've added a software versions table, expanded evaluation metric descriptions, and provided more detailed information on preprocessing workflows to improve reproducibility.
6. **Added trajectory analysis for cell subpopulations**: We've included additional trajectory conservation analyses distinguishing between major and minor cell types to provide deeper insights into method performance.
7. **Corrected terminology and improved clarity**: We've addressed unclear or imprecise terminology throughout the manuscript to enhance readability.
8. **Enhanced reproducibility**: We've provided comprehensive environment configuration files and installation instructions in our repository to facilitate result reproduction.

These revisions have significantly improved the manuscript while maintaining technical rigor. Thank you again for your valuable feedback. Please find the point by point response below.

Major comments:
1) The results/discussion is quite descriptive at this stage and the implication of the benchmarking results is not clear. How can one benefit from this extensive study and

what recommendation and insight for scientist? The discussion section can be substantially expand.

Response: Thank you for your insightful feedback regarding the descriptive nature of our results/discussion and the need for clearer implications of our benchmarking results. We agree that providing more explicit recommendations and insights would significantly enhance the manuscript's value to the scientific community.

In response to your suggestion, we have made the following substantial revisions:

1. We have restructured Section 2.7 into a dedicated "Tailored Method Selection Guide for Multi-omics Integration Scenarios" that consolidates our findings from all previous analyses, including integration accuracy, biomarker detection, trajectory conservation, batch effect correction, and computational efficiency. This section now provides clear, practical recommendations for researchers based on their specific experimental contexts and analysis priorities.
2. We have updated Figure 6 to include a comprehensive visualization of the best-performing methods across all evaluation dimensions, making it easier for readers to identify optimal methods for their specific needs. The figure now serves as a practical reference guide that summarizes our extensive benchmarking results.
3. Incorporating additional reviewer comments, we have substantially expanded the Discussion section with a more organized structure that includes:
    - A concise summary of our overall work and contributions
    - A dedicated subsection on "Performance Trade-offs and Biological Signal Preservation" that discusses the key insights regarding the balance between technical integration and biological relevance. This revision also takes into account comment (7) of Reviewer 1.
    - A focused subsection on "Foundation Models in Multi-omics Integration" that examines the unique capabilities and limitations of these emerging approaches. This revision considers comment (5) of Reviewer 4 as well.
    - A comprehensive "Limitations and Future Directions" subsection that not only outlines challenges in the field but also candidly addresses the limitations of our current benchmarking framework, including constraints in dataset selection, computational resources, evaluation metrics, and the evolving nature of foundation models. The revision of this part also considers comment (3) and (5) of Reviewer 1.

This reorganized discussion now provides clearer implications of our findings, highlights the most important insights for method developers and users, and offers specific recommendations for different research scenarios, while also acknowledging the areas

where our benchmarking could be improved in future work. We believe these changes significantly improve the manuscript's utility to the scientific community while maintaining the thoroughness of our benchmarking analysis.

Revised Sentences:

[Section 2.7 Tailored Method Selection Guide for Multi-omics Integration Scenarios]

Based on our comprehensive benchmarking, we offer the following practical recommendations for researchers selecting integration methods: For integration accuracy, GLUE, scMDC, scJoint, and Seurat4 emerge as top methods. Seurat4 demonstrates particularly strong performance in unpaired settings, while scJoint stands out as the premier choice for unpaired integration with superior performance and moderate computational requirements. For cross-species analyses, UCE performs best among FMs, though our adaptation approach (UCE-scVI) offers improved performance while maintaining cross-species capabilities. For biomarker detection, Seurat4 and scJoint demonstrate superior overall performance. Seurat4 particularly excels in chromatin accessibility analysis, while GLUE and Harmony better preserve transcriptomic-level markers in specific cell types. Methods with moderate JSI may identify unique biomarkers missed by other approaches, offering complementary biological insights. For trajectory conservation, MOFA performs best in paired settings, while Harmony leads in unpaired scenarios. For specific developmental branches, Seurat5 shows strong performance in B cell trajectories, and UCE demonstrates unique strength in mouse developmental pathways, highlighting its cross-species capabilities in preserving evolutionary conserved developmental programs. For batch effect correction, scJoint and MMD-MA stand out by achieving impressive results in both batch correction and integration accuracy, effectively addressing the inherent trade-off between these objectives in multi-omics data integration. When computational resources are limited, scJoint, bindSC, and Harmony offer good performance-to-efficiency ratios across different analysis tasks, making them versatile options for various research contexts.

We provide a detailed recommendation pipeline that integrates the strengths of statistical-based methods and deep learning-based methods, considering their capabilities in handling unpaired datasets and scalability across different species (see Fig. 6 e). The top two recommended methods are annotated for each aspect. Given that foundation models typically do not perform as well as specialized deep learning methods, we propose the FM + AdaptationLayer approach. This method is designed to enhance the performance of FMs in this context and provides guidance for future model design. We have made all benchmark code, evaluation metrics, and our FM-scVI implementation publicly available to facilitate method selection and future benchmarking efforts. All detailed evaluation results are provided as extended data with clear documentation for comprehensive reference. As the field continues to evolve, we

encourage researchers to consider these comparative results alongside their specific biological questions and technical constraints.

[Discussion] With the rapid advancement of techniques for integrating single-cell multi-omics data, thorough evaluations are crucial for selecting appropriate methods in practical experiments. Previous studies, such as Lee et al. [32], benchmark nine integration methods but overlook the latest deep learning-based approaches and lack comprehensive downstream task analyses. More recently, Xiao et al. [33] expand this scope to twelve multi-omics integration methods and include trajectory inference tasks. However, their study does not fully explore critical tasks such as biomarker detection and batch effect correction. In our study, we evaluate 19 DMs and four FMs across a diverse collection of both real and simulated multi-omics datasets. We analyze not only integration accuracy but also the maintenance of cellular features, biological processes, and regulatory mechanisms through comprehensive downstream analyses. Our evaluation framework consists of 13 metrics spanning integration accuracy, biomarker detection, trajectory inference, and batch effect correction. Previous methods have primarily focused on datasets comprising no more than three components. However, statistical analysis cannot be performed on a single data point, especially when evaluating the performance separately for paired and unpaired scenarios. In this study, we consider a total of six datasets, with two containing different batches that we partition for additional evaluation beyond batch correction and one triple omics data from mouse cortex. For a detailed comparison between our study and existing works, please refer to Supplementary Table 3.

Performance Trade-offs and Biological Signal Preservation Our comprehensive evaluation reveals important trade-offs in method performance across different tasks. The varying effectiveness of integration methods across trajectory conservation assessments reflects the inherent challenges in preserving complex biological signals during multi-omics integration. We observe a clear division between methods optimized for paired versus unpaired settings, with MOFA and Cobolt emerging as recommended choices, respectively. The inconsistent performance of Harmony across datasets highlights the sensitivity of some methods to specific data characteristics, including species origin and experimental design. Most current methods focus primarily on technical integration accuracy rather than biological signal preservation. Our biomarker detection and trajectory conservation evaluations highlight this issue, with methods excelling at cell-type clustering often underperforming in preserving developmental trajectories or regulatory relationships. This finding underscores the need for integration methods that balance technical accuracy with biological relevance, particularly as integration results increasingly inform downstream functional and mechanistic analyses.

FMs in Multi-omics Integration While FMs have demonstrated transformative potential in other fields, their capabilities in single-cell biology remain underexplored. Our

benchmark results, alongside published literature, suggest that current single-cell FMs have not yet been adequately harnessed for this domain. While generally underperforming compared to specialized DMs in overall integration accuracy, FMs demonstrate unique strengths in versatility and efficiency. Their versatility enables adaptation across diverse single-cell tasks with the same architecture, while their efficiency advantages include both reduced training requirements and computational feasibility on large-scale datasets where many specialized DMs become prohibitively expensive or fail to complete. However, their struggle with multi-modal integration in the zero-shot setting underscores the necessity for appropriate adaptation strategies when handling different data modalities. Most FMs are predominantly trained on human data, leading to cross-species bias when generalizing to other organisms. Even UCE, which attempts to address this by training on diverse tissues and species, suffers from data imbalance issues in its training atlas. The exceptional performance of UCE on mouse developmental data suggests that species-specific pre-training may confer advantages for certain biological contexts. To address these limitations while leveraging FMs' rich biological knowledge, we propose the FM + AdaptationLayer approach. Specifically, we develop FM-scVI, a hybrid architecture that combines FM embeddings with scVI's variational autoencoder framework. We implement this adaptation as a standalone method and incorporate it into our comprehensive benchmarking pipeline, evaluating it alongside traditional deep learning methods across all metrics and datasets. Our results demonstrate that this strategy effectively bridges the gap between FMs' biological knowledge and the specific requirements of multi-omics integration tasks, showing particular improvements in biomarker detection and trajectory conservation while maintaining computational efficiency. However, further refinement and validation are necessary to fully realize its potential.

Limitations and Future Directions Despite our comprehensive benchmarking efforts, several limitations should be acknowledged. First, our evaluation focuses primarily on scRNA-seq and scATAC-seq integration, while other important multi-omics combinations (such as protein, spatial, or metabolomics data) remain to be explored. We also conducted triple-omics integration experiments including single-cell DNA methylation data, which showed that only seven methods are capable of handling triple-omics integration, yet all methods exhibited decreased performance compared to dual-omics results (Supplementary Table 2). This highlights the technical challenges in integrating three or more modalities simultaneously. As multi-omics technologies continue to advance, more sophisticated computational approaches are needed to effectively handle complex multi-modal data integration. Second, the field of FMs is rapidly evolving, with new pre-trained models continually emerging; our assessment represents a snapshot of 19 currently popular models rather than an exhaustive evaluation. Due to the limited number of FMs designed to handle multi-omics integration tasks, comparing their zero-shot performance with specialized DMs may not be entirely

fair. We included our FM + AdaptationLayer approach to partially address this limitation, though this represents just one possible direction for leveraging FMs in this domain and is not the primary focus of this work. Substantial further research will be needed to fully realize the potential of FMs in multi-omics integration. Third, computational scalability for atlas-level integration remains a critical challenge, and our benchmarking platform itself imposed certain limitations on comprehensive evaluation across all methods for the largest datasets. Despite using a high-performance computing setup with multi-core CPUs, 128GB RAM, and NVIDIA RTX 3090 GPUs, several methods still encoun tered resource limitations. While several methods (GLUE, LINGER, Seurat4, scVI, iNMF, Cobolt, and scJoint) successfully processed our largest unpaired dataset (Yao-2021 with 69,727 scRNA-seq cells and 54,844 snATAC-seq cells), others encountered significant barriers. We observed out-of-memory errors with scMoMaT, MMD-MA, and UnionCom on moderately sized datasets like Ma-2020 (32,321 cells), while Pamona's runtime exceeded 24 hours. These platform-imposed constraints highlight the urgent need for more efficient algorithms capable of handling the increasingly massive datasets generated by contemporary single-cell technologies, as computational limitations remain a significant practical barrier to method adoption. Fourth, expanding the benchmark with additional datasets would further enhance the generalizability of our findings. Importantly, our results highlight that method performanceis often case-specific, and we found it challenging to identify any single method that consistently outperformed others across all metrics, datasets, and analysis tasks. This context-dependence is further ev- idenced by our statistical analyses using the Friedman test followed by Conover post-hoc comparisons (Supplementary Figure 14), which reveal that most methods fall within statistically indistinguishable performance groups ($p < 0.05$). With few exceptions, we observe widespread statistical parity among methods, particularly striking given the diversity of their algorithmic approaches—from simple linear techniques to complex deep learning architectures. While our Friedman test indicated significant overall differences among methods ($p < 0.001$), the post-hoc comparisons and resulting critical difference diagrams show that these differences are primarily between clusters of methods rather than between individual approaches. This statistical equivalence suggests we may be approaching optimization plateaus with current paradigms for certain integration tasks, and indicates that researchers have multiple viable options when selecting integration methods, allowing secondary considerations such as computational efficiency to guide decision-making without significantly compromising performance. The convergence of performance across diverse mathematical frameworks also highlights the robustness of biological signals across computational representations. As the field continues to generate more multi-omics datasets, future benchmarking efforts could build upon our evaluation framework, but developing truly adaptable, universally effective integration methods that perform consistently across varied biological contexts remains a fundamental challenge

and important direction for future research. Our study offers a comprehensive benchmarking analysis of both DMs and FMs in the context of aligning and integrating multi-omics data. We anticipate that this analysis will provide valuable guidance for users in selecting appropriate methods and inspire researchers to develop novel multi-omics integration tools with more thorough evaluations. Future development should focus on creating more adaptive approaches that maintain trajectory information regardless of experimental setting, perhaps by incorporating deeper biological priors into integration algorithms. Additionally, we aim to unlock the full potential of FMs and promote their effective application in single-cell multi-omics integration.

2) Figure 2a is confusing to readers. The matrices above the diagram show the names of two datasets (PBMC-10x and Ma-2020). It appears that PBMC-10x is used for the integration and bio-conservation tasks, while Ma-2020 is used for the batch correction task and computational efficiency evaluation. However, in the main manuscript, Figure 2a is described as being based only on PBMC-10x. Please clarify the information presented in this panel.

Response: We greatly appreciate the reviewer's point regarding the potential confusion in Figure 2a. The reviewer is correct in identifying an inconsistency between the figure presentation and its description in the manuscript. We acknowledge that our original presentation could be misleading.

To clarify, our intention with Figure 2a was to provide a comprehensive overview of all methods across all evaluation aspects (integration accuracy, biological conservation, batch correction, computational efficiency, and applicability). Since the PBMC-10x dataset does not contain batch effect information, we utilized the Ma-2020 dataset to demonstrate batch correction capabilities. However, the computational efficiency evaluation was indeed based on the PBMC-10x dataset, as correctly noted in the main text.

We agree that this mixed presentation across different datasets within the same figure panel, despite being described in the text, could create confusion for readers. To address this issue, we have reorganized Figure 2 to ensure clarity and consistency between the figure content and its description in the manuscript. The revised figure now clearly indicates which dataset was used for each evaluation aspect, with appropriate labeling to prevent any misinterpretation. Figure 2a now specifically shows benchmark results for paired datasets, while Figure 2b presents results for unpaired datasets, with added icons to clearly distinguish between them. We have moved the computational efficiency evaluation to Figure 2d, using the PBMC-10x dataset since this is the only dataset that all methods could process completely. We believe this reorganization

significantly improves the presentation of our comprehensive evaluation and makes the information more accessible to readers.

[Figure Redacted]

Figure R1: Figure 2 in the manuscript

Revised Sentences:

[Figure 2] Figure 2: Benchmarking results of the integration accuracy. All metrics are the higher the better. a and b. Benchmarking results on the paired and unpaired datasets. Metrics are categorized into three groups: integration accuracy (represented by the color red), bio-conservation (represented by the color yellow), and batch correction (represented by the color green). FM-scVI represents the adapted scGPT on PBMC-10x and the adapted UCE on Ma-2020. scGPT-ft is the fine-tuned version of scGPT, while scGPT-zero is the zero-shot version. c. Unpaired applicability of integration methods and whether GPU acceleration can be used for each method. d. Computational efficiency evaluated on the full PBMC-10x dataset, showing processing time and memory requirements across methods. e and f. Integration accuracy overall scores are evaluated across all paired and unpaid datasets, respectively. Datasets with batch information are split into smaller ones to minimize the impact of batch effects on the integration metrics.

3) While the study includes five real datasets and multiple batches, the total number of paired and unpaired datasets is six each, which is relatively small. The methods' performance in Figures 2d and 2e may be influenced by the integration performance of a single dataset. Although the authors mentioned that the datasets were carefully selected based on criteria of representativeness and popularity, the limited number of datasets may reduce the generalizability of the results. It would be better to discuss this potential limitation of the benchmark study in the Discussion section.

Response: We appreciate the reviewer's thoughtful comment regarding the number of datasets used in our benchmark study. We acknowledge that our evaluation included six paired and six unpaired datasets across five real biological contexts and multiple batches. While we understand the concern about potential limitations in generalizability, we would like to offer several points of clarification.

The datasets in our study were carefully selected with specific criteria to ensure a comprehensive evaluation of multi-omics integration methods. Notably, many of the datasets we selected follow those used in the benchmark study of GLUE [1], which have been widely adopted and validated in the field. Previous benchmark studies have been limited in scope, with [2] and [3] each evaluating only three datasets respectively. In contrast, our study encompasses six representative real-world datasets to provide a more comprehensive evaluation. As shown in the dataset characteristics table provided in Extended_Data_1_Data&Methods, our selection encompasses both paired and unpaired datasets, human and mouse samples, varying sample sizes, and different omics combinations. We have also incorporated triple-omics datasets to evaluate method performance on more complex integration tasks. The Triple dataset contains

55,803 cells for scRNA-seq, 2,317 cells for scATAC-seq, and 3,377 cells for snmC-seq. Furthermore, the benchmark datasets used in our study span a wide range of dataset sizes, from approximately 2,000 to over 50,000 cells, which allows us to assess the generalization ability of integration methods across varying data scales. Our dataset selection was guided by the primary objective of benchmarking multi-omics integration methods across three key aspects: integration accuracy, biological conservation, and batch correction capability. For datasets like Ma et al. (2020) and Muto et al., which contain explicit batch labels, we strategically partitioned them to enable isolated assessment of integration accuracy and biological conservation without confounding batch effects. The whole datasets were then specifically utilized in our batch correction analysis section to evaluate the methods' performance in addressing technical variation. In summary, our benchmark study leverages six representative real-world datasets from diverse sources, covering paired, unpaired, and triple-omics scenarios. In addition, thoughtful preprocessing, subsampling strategies, and simulated data have further increased the diversity and complexity of the datasets. Updated information can be found in Figure1b and Supplementary Table 3 in the manuscript:

Dataset	Species	Paired	Organ	Protocol	Cell numbers	Batch Effect
PBMC-10x	Homo sapiens	T	PBMC	10x Multiome	9,631	F
Chen-2019	Mus musculus	T	Cortex	SHARE-seq	9,190	F
Ma-2020	Mus musculus	T	Skin	SHARE-seq	32,231	T
Muto-2021	Homo sapiens	F	Kidney	snRNA-seq<br>snATAC-seq	19,985<br>24,205	T
Yao-2021	Mus musculus	F	MOp	scRNA 10x v3<br>snATAC-seq	69,727<br>54,844	F
Triple	Mus musculus	F	Cortex	Drop-seq<br>scATAC 10x<br>snmC-seq	55803<br>2317<br>3377	F

Figure R2: Figure 1b in the manuscript

Table R1: Supplementary Table 3 in the manuscript

Supplementary Table 3: Comparison of the existing benchmarking works.

Paper	Omics	Datasets	Methods	Downstream Tasks			comparing with FM
				BD	TI	BC	
Lee [30]	2	3	9	✓			
Xiao [31]	2	3	12	✓	✓		
SCMBench	3	6	23	✓	✓	✓	✓

We appreciate the suggestion to discuss this potential limitation in our paper. We have added a paragraph in the Discussion section acknowledging this limitation and noting that while our benchmark provides valuable guidance based on the examined datasets, researchers should consider their specific biological contexts when choosing integration methods. We have also highlighted opportunities for future work to expand our evaluation framework with additional datasets representing other biological systems and technical platforms, particularly as the field continues to evolve with new experimental protocols generating increasingly complex multi-omics data.

[1] Cao Z J, Gao G. Multi-omics single-cell data integration and regulatory inference with graph-linked embedding[J]. Nature Biotechnology, 2022, 40(10): 1458-1466.
[2] Lee, M. Y., Kaestner, K. H. & Li, M. Benchmarking algorithms for joint integration of unpaired and paired single-cell rna-seq and atac-seq data. Genome Biology 24, 244 (2023).
[3] Xiao, C., Chen, Y., Meng, Q., Wei, L. & Zhang, X. Benchmarking multi-omics integration algorithms across single-cell rna and atac data. Briefings in Bioinformatics 25, bbae095 (2024).

Added sentences:

[Discussion] **Limitations and Future Directions** Despite our comprehensive benchmarking efforts, several limitations should be acknowledged.First, our evaluation focuses primarily on scRNA-seq and scATAC-seq integration, while other important multi-omics combinations (such as protein, spatial, or metabolomics data) remain to be explored. We also conducted triple-omics integration experiments including single-cell DNA methylation data, which showed that only seven methods are capable of handling triple-omics integration, yet all methods exhibited decreased performance compared to dual-omics results (Supplementary Table 2). This highlights the technical challenges in integrating three or more modalities simultaneously. As multi-omics technologies continue to advance, more sophisticated computational approaches are needed to effectively handle complex multi-modal data integration. Second, the field of FMs is rapidly evolving, with new pre-trained models continually emerging; our assessment represents a snapshot of currently popular models rather than an exhaustive evaluation. Due to the limited number of FMs designed to handle multi-omics integration tasks, comparing their zero-shot performance with specialized DMs may not be entirely fair. We included our FM + AdaptationLayer approach to partially address this limitation, though this represents just one possible direction for leveraging FMs in this domain and is not the primary focus of this work. Substantial further research will be needed to fully realize the potential of FMs in multi-omics integration. Third, computational scalability for atlas-level integration remains a critical challenge, and our benchmarking platform itself imposed certain limitations on comprehensive evaluation across all methods for

the largest datasets. Despite using a high-performance computing setup with multi-core CPUs, 128GB RAM, and NVIDIA RTX 3090 GPUs, several methods still encountered resource limitations. While several methods (GLUE, LINGER, Seurat4, scVI, iNMF, Cobolt, and scJoint) successfully processed our largest unpaired dataset (Yao-2021 with 69,727 scRNA-seq cells and 54,844 snATAC-seq cells), others encountered significant barriers. We observed out-of-memory errors with scMoMaT, MMD-MA, and UnionCom on moderately sized datasets like Ma-2020 (32,321 cells), while Pamona's runtime exceeded 24 hours. These platform-imposed constraints highlight the urgent need for more efficient algorithms capable of handling the increasingly massive datasets generated by contemporary single-cell technologies, as computational limitations remain a significant practical barrier to method adoption. Fourth, expanding the benchmark with additional datasets would further enhance the generalizability of our findings. Importantly, our results highlight that method performance is often case-specific, and we found it challenging to identify any single method that consistently outperformed others across all metrics, datasets, and analysis tasks. This context-dependent performance underscores the complexity of multi-omics integration and suggests that developing more adaptable, universally effective integration methods remains an important direction for future research. As the field continues to generate more multi-omics datasets, future benchmarking efforts could build upon our evaluation framework to provide even more robust guidance for the research community.

4) When assessing biomarker detection consistency and trajectory alignment, the authors use both boxplots and critical difference diagrams to show the methods' performance by rank. However, we noticed that the two types of graphs are quite confusing. For example, in Figure 3b, Seurat5 is shown as the top-performing method, but in Figure 3c, it is ranked in the middle. What is the difference between the terms "overall marker detection" and "cell type-specific marker detection"? Further clarification is required to explain why the performance in these two scenarios differs so significantly. Similar issues are observed in Figures 4b and 4c, where scGPT_zero is ranked fourth in the critical difference diagram but appears in the middle of the boxplot.

Response: We thank the reviewer for this insightful observation about the apparent discrepancies between our boxplot rankings and critical difference diagrams. This comment highlights an important methodological distinction that deserves further clarification.

To directly address the reviewer's question about why performance appears to differ so significantly between these visualizations: the apparent discrepancy is not due to different underlying data or metrics, but rather stems from the fundamentally different statistical approaches used to generate these two visualization types.

We would like to clarify that "overall biomarker detection" and "cell type-specific marker detection" are based on the same underlying scores. However, the boxplots and critical difference diagrams present this information in distinct ways:

The boxplots rank methods according to their mean performance scores across all datasets, with the central line representing the median. This approach emphasizes the actual magnitude of performance differences. A method like Seurat5 may have a high average score (appearing at the top of the boxplot) but potentially with higher variability across datasets.

The critical difference diagrams convert these same scores into ranks for each dataset and then calculate the average rank across datasets, using the Friedman test followed by Conover post-hoc comparisons to determine statistical significance. This approach emphasizes consistent relative performance rather than absolute score values. A method may have moderately high but very consistent performance across all datasets (appearing strong in the critical difference diagram) versus another method with extremely high performance in some datasets but poor performance in others (appearing weaker in the critical difference diagram despite a potentially higher mean score).

For example, a method that ranks 1st, 2nd, and 10th across three datasets would have a worse average rank than a method ranking 3rd, 4th, and 5th, even though the first method might have higher absolute scores in some datasets. This explains why methods like Seurat5 and scGPT_zero can appear in different positions in these complementary visualizations.

In our revised manuscript, we have made the editorial decision to present only boxplots in the main text, as they provide more direct visualization of actual performance metrics. However, we have included corresponding critical difference diagrams for all analyses in the supplementary materials (Supplementary Figure 14). We have added specific descriptions in each figure legend to clarify the statistical interpretation of both visualization types, ensuring readers understand why these two approaches may suggest different rankings.

Revised Sentences:

[Caption of figure 2] Figure 2: Benchmarking results of the integration accuracy. All metrics are the higher the better. A and b. Benchmarking results on the paired and unpaired datasets. Metrics are categorized into three groups: integration accuracy (in red), bio-conservation (in yellow), and batch correction (in green). FM-scVI represents the adapted scGPT on PBMC-10x and the adapted UCE on Ma-2020. scGPT-ft is the fine-tuned version of scGPT, while scGPT-zero is the zero-shot version. c. Unpaired

applicability of integration methods and whether GPU acceleration can be used for each method. d. Computational efficiency evaluated on the full PBMC-10x dataset, showing processing time and memory requirements across methods. e and f. Integration accuracy overall scores are evaluated across all paired and unpaid datasets, respectively. Datasets with batch information are split into smaller ones to minimize the impact of batch effects on the integration metrics. Methods are ranked according to the mean performance and the middle line in each box represents the median score.

[Caption of supplementary figure 14] Supplementary Figure 14: Critical difference diagrams of different metrics. These diagrams display the average ranks of methods across datasets based on the Friedman test with Conover post-hoc analysis. Methods with lower ranks (positioned more to the right) performed better on average, and horizontal lines connect methods whose performance differences are not statistically significant ($p > 0.05$). Unlike boxplots that show absolute score magnitudes, these diagrams emphasize consistent relative performance across datasets. a-d. Critical difference diagrams of (a) Biomarker detection, (b) Enriched motifs, (c) DARs and (d) the overall biological conservation scores. e-f. Critical difference diagrams of trajectory conservation of paired datasets (e) and unpaired datasets (f). g-h. Critical difference diagrams of integration accuracy of paired datasets (g) and unpaired datasets (h).

[Section 2.2] Critical difference diagrams in Supplementary Figure 14g reveal overlapping statistical groups. While GLUE ranks first, it does not significantly outperform scJoint and other top methods. Conversely, scJoint, despite ranking second, shows no significant advantage over the remaining methods. This suggests that GLUE occupies a unique position by grouping with top performers while maintaining consistent first-place ranking, whereas scJoint bridges the top and middle-tier methods. Based on this statistical grouping pattern and GLUE's stable top-tier performance across diverse datasets, we conclude that GLUE emerges as the most consistently reliable method for paired multi-omics integration.

We believe this revision allows readers to understand both the practical performance differences and how consistently methods perform across diverse datasets and conditions.

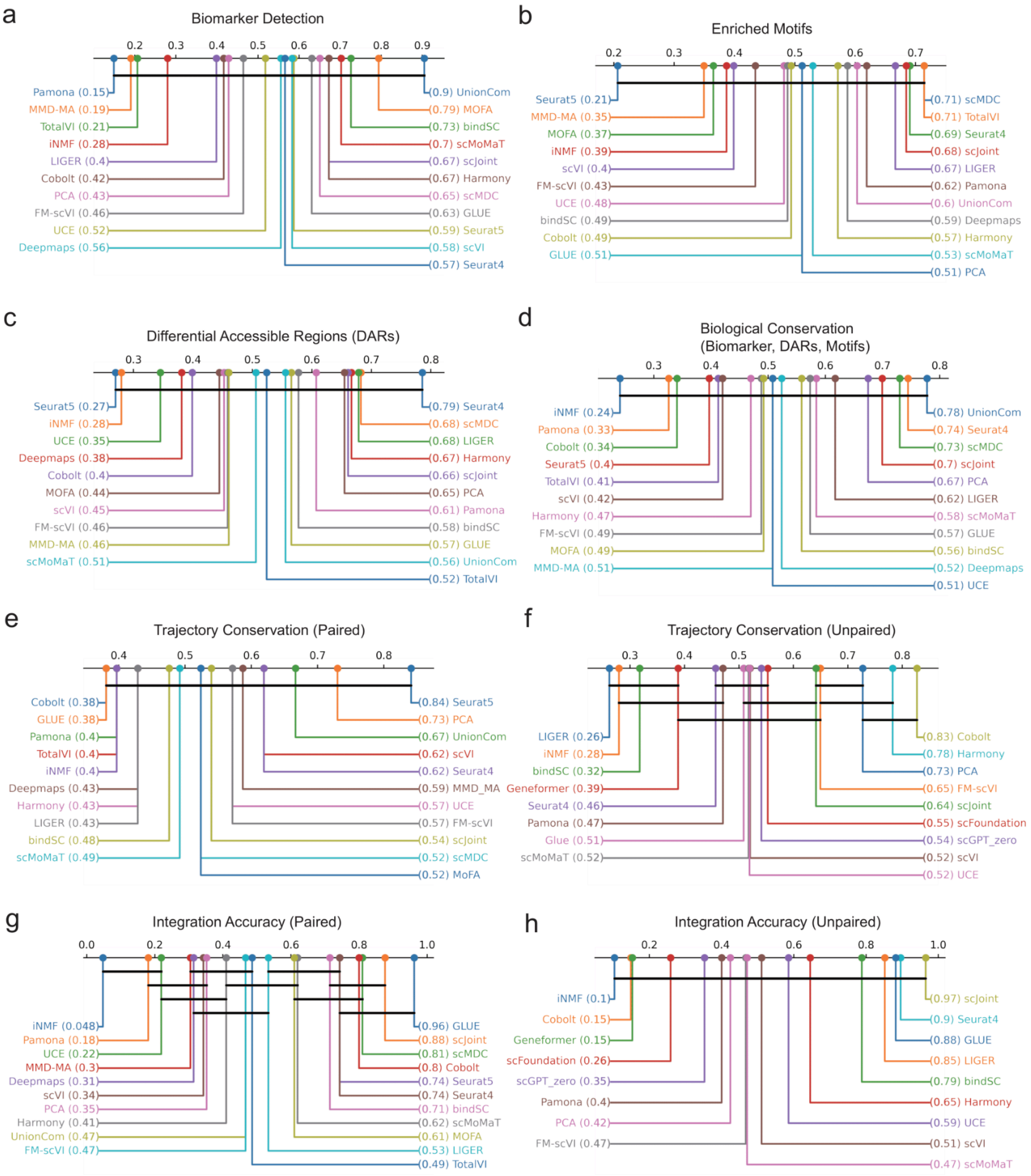

Figure R3: Supplementary Figure 14 in the manuscript

5) Although critical difference diagrams are included, the interpretation of statistically significant or insignificant differences between methods could be better addressed in the manuscript. Is there any potential meaning behind the statistical insignificance

observed among the top-performing methods? For example, Figure 3C, MOFA and GLUE, Figure 4C, Cobolt and PCA, PCA and scJoint.

Response: We appreciate the reviewer's insightful question regarding the interpretation of statistical significance in our critical difference diagrams. The statistical insignificance observed among top-performing methods indeed carries important implications that warrant further discussion.

The horizontal lines connecting methods in our critical difference diagrams indicate that despite numerical differences in their average ranks, these differences are not statistically significant based on the Conover post-hoc test ($p > 0.05$). As demonstrated in our new Supplementary Figure 14, this pattern is prevalent across nearly all our evaluation metrics. With the exception of trajectory conservation in unpaired settings and integration accuracy in paired settings, where top-performing groups show some separation from other methods (though still not complete distinction), most other scenarios display a single connected group of methods or heavily overlapping groups.

This widespread statistical indistinguishability has several meaningful interpretations for the field:

1. **Method selection flexibility**: The lack of statistically significant differences among top methods suggests that researchers have multiple viable options that perform comparably well. This flexibility is valuable in practical applications where other considerations (such as computational efficiency, ease of use, or compatibility with existing workflows) can guide method selection without significantly compromising performance.
2. **Task-specific optimization plateaus**: The statistical ties among different methodological approaches may indicate that we are approaching optimization plateaus for certain integration tasks with current algorithmic paradigms. This suggests that dramatic performance improvements may require fundamentally new approaches rather than incremental refinements to existing methods.
3. **Dataset-dependent variability**: The statistical insignificance often reflects dataset-dependent performance variations, where methods may excel on certain datasets but perform less optimally on others. For example, the statistical tie between Cobolt and PCA in trajectory conservation suggests that while these methods employ dramatically different approaches (complex deep learning versus simple linear dimensionality reduction), their performance advantages may be context-specific rather than universal.
4. **Biological context relevance**: The observed statistical ties between methods with different underlying algorithms (e.g., MOFA's factor analysis approach versus GLUE's graph neural networks) suggest that multiple mathematical

frameworks can effectively capture the same underlying biological signals. This convergence speaks to the robustness of the biological patterns being detected.

In our revised manuscript, we have expanded our discussion of these statistical findings to highlight these implications. We have also emphasized how the complementary information from both boxplots (showing absolute performance magnitudes) and critical difference diagrams (showing statistical significance) should inform researchers' method selection decisions.

Added Sentences:

[Discussion] Fourth, expanding the benchmark with additional datasets would further enhance the generalizability of our findings. Importantly, our results highlight that method performance is often case-specific, and we found it challenging to identify any single method that consistently outperformed others across all metrics, datasets, and analysis tasks. This context-dependence is further evidenced by our statistical analyses using the Friedman test followed by Conover post-hoc comparisons (Supplementary Figure 14). With few exceptions, we observe widespread statistical parity among methods, particularly striking given the diversity of their algorithmic approaches—from simple linear techniques to complex deep learning architectures. While our Friedman test indicated significant overall differences among methods, the critical difference diagrams show that most methods fall within statistically indistinguishable groups ($p > 0.05$), particularly striking given the diversity of their algorithmic approaches. This statistical equivalence suggests we may be approaching optimization plateaus with current integration paradigms, and indicates that researchers have multiple viable options when selecting methods, allowing secondary considerations such as computational efficiency to guide decision-making without significantly compromising performance. As the field continues to generate more multi-omics datasets, developing truly adaptable, universally effective integration methods that perform consistently across varied biological contexts remains a fundamental challenge and important direction for future research.

6) Page 9, please provide more detailed explaination on the motivation/logic behind how moderate Jaccard indices can act as a metric to determine how each of the methods are valuable for exploring novel or cell-type-specific biomarkers.

Response: We appreciate the reviewer's request for more detailed explanation regarding our statement about moderate Jaccard indices and their relationship to novel biomarker discovery. The reviewer is asking for clarification on the logic behind our

assertion that "methods with moderate Jaccard indices, such as DeepMaps and TotalVI, are valuable for exploring novel or cell-type-specific biomarkers."

Our reasoning is based on the interpretation of what different ranges of Jaccard similarity index (JSI) values represent in the context of biomarker detection. Methods with very high JSI values identify marker genes that are largely consistent with those identified by other methods, suggesting these are well-established, commonly detected biomarkers. Conversely, methods with lower to moderate JSI values are detecting partially different sets of marker genes compared to the consensus.

Our intention is not to dismiss this metric's utility for evaluating methods' ability to explore novel or cell-type-specific biomarkers, but rather to illustrate the potential complementary value it reveals. This moderate overlap could indicate that these methods are capturing unique aspects of the data that others miss, potentially identifying novel or alternative biomarkers that might be biologically relevant but not commonly detected. Rather than simply representing poorer performance, these methods could be complementary to high-JSI methods by expanding the repertoire of candidate biomarkers.

To provide more robust support for this claim, we could strengthen our explanation by including specific examples of unique biomarkers identified by moderate-JSI methods that were missed by high-JSI methods but have biological relevance based on literature. Specifically, we compare candidate marker genes identified for NK cells by DeepMAPS (moderate JSI) and GLUE (highest JSI). Notably, DeepMAPS uniquely captured *SLAMF7* and *KLRG1*, which were absent from GLUE's results. *SLAMF7* is a well-established NK-specific surface receptor involved in cytotoxicity and immune activation [1]. *KLRG1* is another key inhibitory receptor highly expressed on mature NK cells and associated with terminal differentiation and NK cell cytotoxicity inhibition [2]. These findings illustrate that moderate-JSI methods can uncover cell-type-specific or functionally relevant markers beyond the consensus, highlighting their complementary value in biomarker discovery.

To more rigorously evaluate this aspect—considering the subsequent reviewer comments (4) of Reviewer 3—we further incorporated prior knowledge from the Cell Marker 2.0 database as ground truth to assess the biological relevance of the identified biomarkers for the PBMC-10x dataset and show in Supplementary Figure 10. We thank the reviewer for highlighting this point, as providing this additional evidence would strengthen our interpretation and offer valuable insights for researchers selecting methods based on their specific biomarker discovery objectives.

[1] Campbell K S, Cohen A D, Pazina T. Mechanisms of NK cell activation and clinical activity of the therapeutic SLAMF7 antibody, elotuzumab in multiple myeloma[J]. Frontiers in immunology, 2018, 9: 2551.
[2] Ito M, Maruyama T, Saito N, et al. Killer cell lectin-like receptor G1 binds three members of the classical cadherin family to inhibit NK cell cytotoxicity[J]. The Journal of experimental medicine, 2006, 203(2): 289-295.

Revised Sentences:
[Section 2.3] Meanwhile, methods with moderate JSI, such as DeepMaps and TotalVI, may offer complementary value for exploring novel or cell-type-specific biomarkers, as they potentially capture unique aspects of the data and uncover alternative biomarkers not commonly detected by other methods. To substantiate this observation, we compared candidate marker genes identified for NK cells by DeepMAPS (moderate JSI) and GLUE (highest JSI). Notably, DeepMAPS uniquely captured SLAMF7 and KLRG1, which were absent from GLUE's results. SLAMF7 is a well-established NK-specific surface receptor involved in cytotoxicity and immune activation [53], while KLRG1 is a key inhibitory receptor highly expressed on mature NK cells and associated with terminal differentiation [54]. These biologically relevant markers, missed by the highest-consensus method but captured by a moderate-JSI approach, demonstrate that different methods can offer complementary insights into the biomarker landscape, with moderate-JSI methods potentially identifying functionally significant markers beyond the consensus set.

Added Figure:
[Supplementary Figure 4]

a.

b.

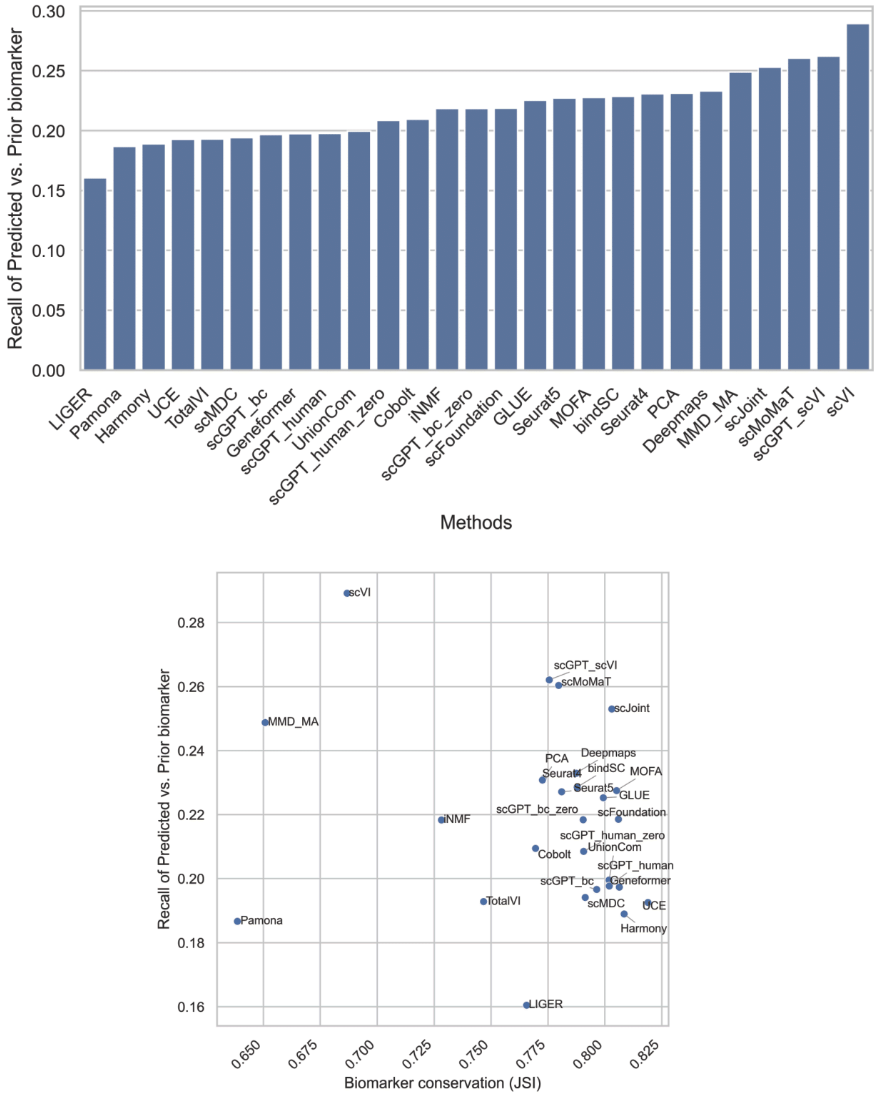

7) Page 12, Section 2.5 paragraph 2, detailed explanations of potential reasons are required on the trade-offs between batch effect correction and integration accuracy.

Thank you for your valuable suggestion regarding the trade-offs between batch effect correction and integration accuracy in Section 2.5, paragraph 2 on page 12. Following your recommendation, we have significantly expanded this section with detailed explanations of the potential reasons behind these trade-offs.

Specifically, we have added the following key explanations:

1. We have clarified the fundamental cause of the trade-off between batch effect correction and integration accuracy, pointing out that the mathematical objectives of these two goals are inherently competing. Methods designed for effective batch correction typically employ aggressive normalization strategies that may reduce the technical variation, but may inadvertently remove biologically relevant signals. This trade-off has also been discussed in several studies on batch effect correction in single-cell genomics data integration. For example, in a benchmarking study of batch-effect correction methods for single-cell RNA sequencing data [1], the researchers observed that "the ASW metrics show no clearly superior method, with methods showing tradeoff between scores in batch mixing and cell type purity." Similarly, in research focused on atlas-level single-cell genomics integration [2], the investigators noted that "particularly in more complex integration tasks, we observed a tradeoff between batch effect removal and bio-conservation." These observations highlight that balancing batch effect correction against biological signal preservation represents a fundamental challenge for data integration methods.
2. We illustrated this trade-off through concrete examples, showing how methods like iNMF and Cobolt excel at batch effect removal but fail to generate cell-type specific embeddings, resulting in low biological conservation metrics; conversely, scMDC achieves the highest performance in cell type identification but is significantly impacted by batch effects.
3. We explained how increasing batch effect strength makes this fundamental trade-off more pronounced, and how under extreme technical variation, methods incorporating both probabilistic modeling and domain adaptation may offer the best compromise between biological signal preservation and batch effect correction.

We believe these additions provide readers with deeper insights into why different methods perform differently on these key metrics and how method selection should be based on specific research questions and data characteristics.

[1] Tran, H.T.N., Ang, K.S., Chevrier, M. et al. A benchmark of batch-effect correction methods for single-cell RNA sequencing data. Genome Biol 21, 12 (2020). https://doi.org/10.1186/s13059-019-1850-9

[2] Luecken, M.D., Büttner, M., Chaichoompu, K. et al. Benchmarking atlas-level data integration in single-cell genomics. Nat Methods 19, 41–50 (2022). https://doi.org/10.1038/s41592-021-01336-8

Revised Sentences:

[Section 2.5] Analysis of batch correction-related scores alongside integration accuracy-related scores reveals a fundamental trade-off in multi-omics integration [1][2]. First, the mathematical objectives of these two goals are inherently competing. Methods designed for effective batch effect correction typically employ aggressive normalization or alignment strategies that may reduce technical variation across batches, but these same strategies can inadvertently remove biologically relevant signals. Conversely, approaches prioritizing the preservation of biological structures often retain unwanted technical artifacts. This intrinsic tension stems from the algorithmic challenge of distinguishing technical noise from true biological variation, particularly challenging when batch effects correlate with biological features—a common scenario in multi-omics studies where different platforms or protocols may preferentially capture distinct cellular properties. For instance, methods like iNMF and Cobolt demonstrate superior batch effect removal but fail to generate cell-type-specific embeddings, resulting in low biological conservation metrics. This suggests their algorithms may overcompensate for batch effects, inadvertently removing biologically meaningful variations. The principal components extracted by these methods likely capture technical variation at the expense of the biological signal. Conversely, scMDC achieves the highest performance in cell type identification metrics but is significantly impacted by batch effects, indicating it prioritizes preserving biological heterogeneity over removing technical noise.

Minor issues:

1)Include the specific version of each method involved in this benchmarking study, as performance may change due to implementation upgrades.

Response:

We thank the reviewer for highlighting the importance of version control in benchmarking studies, as performance can indeed vary across software versions. In response, we have compiled a table listing the specific version used for each method included in this study (Table R2).

For packages released via PyPI, Bioconductor, or CRAN, we provide the exact version number. For methods obtained via source code (e.g., GitHub), we indicate the exact commit hash used in our experiments to ensure reproducibility. This information has also been added to the Section 4.3 where we detail the specific implementation of each method in our benchmark framework.

Table R2: Software Versions Used in the SCMBench Study

Methods	Package Version

bindSC	1.0.0
cobolt	0.0.1
Deepmaps	1.0
Geneformer	0.1.0
GLUE	0.3.2
Harmony	0.0.10
iNMF	2.1.0
liger	2.1.0
MMD-MA	https://bitbucket.org/noblelab/2019_mmd_wabi/src/master/
MOFA	0.7.2
Pamona	0.1.0
PCA	scanpy=1.9.8
scFoundation	commit 69b0710660aded7e071d4f67e9f3ac03b096e587
scGPT	0.2.1
scJoint	commit cbbfa5d7e7faa35f3d725bd5257507a01a22810f
scMDC	v1.0.1
scMoMaT	0.2.2
scVI	scvi-tools=1.2.2
Seurat4	5.2.0
Seurat5	5.2.0
TotalVI	scvi-tools=1.2.2
UCE	commit 7b31528b84e4c8e7a9717c61e3d03ff755

	9c61af
UnionCom	0.4.0

2)MoFA should be written as MOFA, refer to the correct capitalisation as used in the original paper.

Response: We sincerely appreciate the reviewer's attention to detail regarding the correct capitalization of "MOFA." We have carefully replaced all instances of "MoFA" with "MOFA" throughout the entire manuscript, including the main text, figure labels, tables, and supplementary materials. We thank the reviewer for highlighting this issue, which has helped improve the accuracy of our manuscript.

3)Page 7, in the last paragraph, it is stated that the definition of the Biomarkers Detection score can be found in Section 4.2.2. However, no explicit definition is provided in Section 4.2.2, the score is only briefly mentioned in the last paragraph of page 7. To improve clarity, the manuscript should either include the full definition in Section 4.2.2 or explicitly reference where the definition is located.

Response: We sincerely appreciate the reviewer's careful observation regarding the definition of the Biomarkers Detection (BM) score. The reviewer is correct that while we mentioned the BM score in the last paragraph of page 7 and stated it could be found in Section 4.2.2, the explicit definition was not clearly provided there. In Section 4.2.2, we primarily introduced the Jaccard similarity index (JSI), which is indeed the main component of our BM score, but we didn't explicitly define how JSI constitutes our BM metric. To address this issue, we have now added a clear definition of the BM score in Section 4.2.2, explaining precisely how it is calculated from the JSI values across methods and cell types within specific datasets. Additionally, we have included a detailed description of our new metric "Biomarker detection with prior knowledge" in this section, which provides another dimension to our evaluation framework. We have also added descriptions of the analogous evaluation strategies used for *Differential Accessible Regions (DARs)* and *Enriched Motifs*, ensuring that all the components of our biological conservation assessment are now clearly defined and reproducible.

Added sentences: [Section 4.2.2]

- **Biomarkers Detection Consistency** (BM): To evaluate the preservation of cell-type-specific biological signals, we computed a biomarker detection score for each method as follows. First, cell types are annotated based on the embeddings

produced by each method using a k-nearest neighbors (kNN) classifier. For each annotated cell type, we identified the top 100 differentially expressed genes (DEGs) as candidate markers. The DEGs are identified from scRNA-seq data using 'rank_genes_groups' function with t-test of Scanpy package. We then calculated the Jaccard similarity index (JSI) of marker gene sets between all pairs of methods, separately for each cell type. The biomarker detection score of a method was defined as the average JSI across all cell types and method pairs involving that method. This score reflects the consistency of cell-type-specific marker detection across different integration approaches.

- **Biomarker detection with prior knowledge**: To evaluate biomarker prediction against known references for the PBMC-10x dataset, we collected curated cell-type-specific markers from CellMarker 2.0 as ground truth [62]. For each method and cell type, we compute the recall by measuring the proportion of predicted candidate biomarkers that match the known markers. This metric reflects agreement with established knowledge, but does not account for potentially novel or unannotated biomarkers.

- **Differential accessible regions detection** (DARs): This score was computed similarly to the biomarker detection score, except that top 100 differentially accessible regions (DARs) were identified for each cell type from scATAC-seq data instead of marker genes. Jaccard similarity indices of DAR sets were calculated across all method pairs and averaged to quantify consistency.

- **Enriched motifs** (Motifs): Building on the DARs identified in the previous step, we performed motif enrichment analysis using GimmeMotifs (with MEME, BioProspector, and Homer as default tools) [52]. Only known motifs curated in the GimmeMotifs database were considered. Surprisingly, the identified DARs are all upregulated, and the enriched motifs may be involved in gene regulation processes. We computed the consistency score by comparing enriched motifs across methods using Jaccard similarity indices, as done for biomarkers and DARs.

Revised Sentences:
[Section 2.3] Beyond evaluating transcriptomic-level markers, we extend our assessment to the epigenomic dimension by analyzing chromatin accessibility patterns in scATAC-seq data. This extension includes two modality-specific features: Differentially Accessible Regions (DARs) identified using similar statistical approaches as DE genes, and enriched transcription factor binding motifs within the top 100 DARs determined through GimmeMotifs analysis. These evaluations are conducted across all

datasets, including paired, unpaired human, and mouse samples, with results displayed in Figure 3 c and d, while detailed dataset-specific results are provided in Supplementary Figure 5. In the epigenomic assessment, Seurat4 achieves the highest scores for both DARs and enriched motifs detection, demonstrating its exceptional capability in capturing chromatin accessibility patterns and regulatory elements. scJoint, LINGER, and Harmony follow closely behind, indicating their strong performance in preserving epigenomic features during integration.

Figure 3 e presents the overall biomarker detection performance ranking across all three metrics. Consistently, bindSC, Seurat4, PCA, and scJoint demonstrate superior performance, establishing themselves as effective methods for multi-omics biomarker discovery. Despite moderate transcriptomic performance, UCE achieves results comparable to several DMs, while at the scATAC-seq level, FM-scVI shows comparable effectiveness to established methods like Harmony and GLUE. These findings suggest that while specialized integration methods currently lead in biomarker detection, foundation models demonstrate promising capabilities that could be enhanced through targeted improvements to preserve both transcriptomic and epigenomic signals.

4)Page 10, paragraph 2, line 3, the authors mention that MOFA ranks first with a TC score of 0.9307. However, in Figure 4a, MOFA's TC score for the pivotal cell types is shown as 0.8273, indicating a performance drop. Does this also occur with other methods? What could be the potential reasons for this discrepancy? This could provide a new angle for the benchmarking study—whether the performance of the methods differs between pivotal cell types and minor cell types.

Response: We appreciate the reviewer’s insightful feedback regarding trajectory conservation evaluation. We also observed that MOFA exhibited a lower TC (trajectory conservation) score within major cell types compared to the score computed across all cells. To assess whether this trend is method-specific, we computed TC scores across three different subsets: all cells, major cell types, and minor cell types. As shown in Figure R4a, there is no consistent discrepancy in TC score distributions across these subsets. Figure R4b further breaks down the TC scores by method, grouping them based on whether TC scores are higher in major cell types than in minor ones (Group 1: TC_major > TC_minor) or vice versa (Group 2: TC_major < TC_minor). These results suggest that the size or abundance of cell types is not the primary determinant of trajectory inference performance, which varies across different methods.

To further strengthen and clarify the evaluation of trajectory conservation, we focused on a well-characterized B cell developmental lineage (HSPCs → Naive B → Intermediate B → Memory B → Plasma cells). Figure R5 shows the UMAP

visualizations of pseudotime inference results from the top eight performing methods, alongside cell type annotations. Among these, Seurat5, PCA, UnionCom, MOFA, and UCE achieved the highest TC scores. For reference, the canonical trajectory derived from prior biological knowledge is also shown. Notably, the embedding generated by Seurat5 clearly captures the developmental progression from Naive B to Memory B cells.

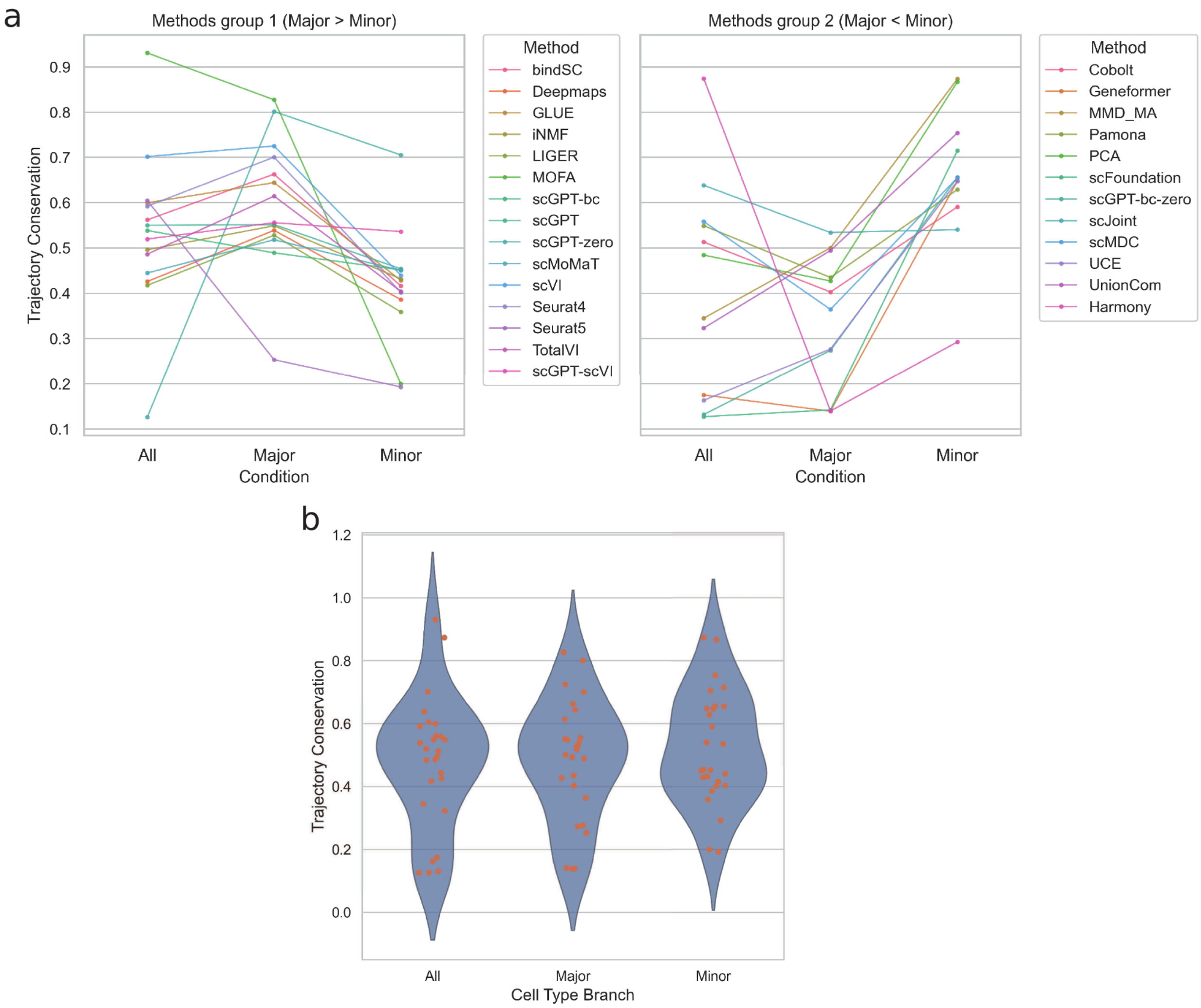

Figure R4: Supplementary Figure 6 in the manuscript

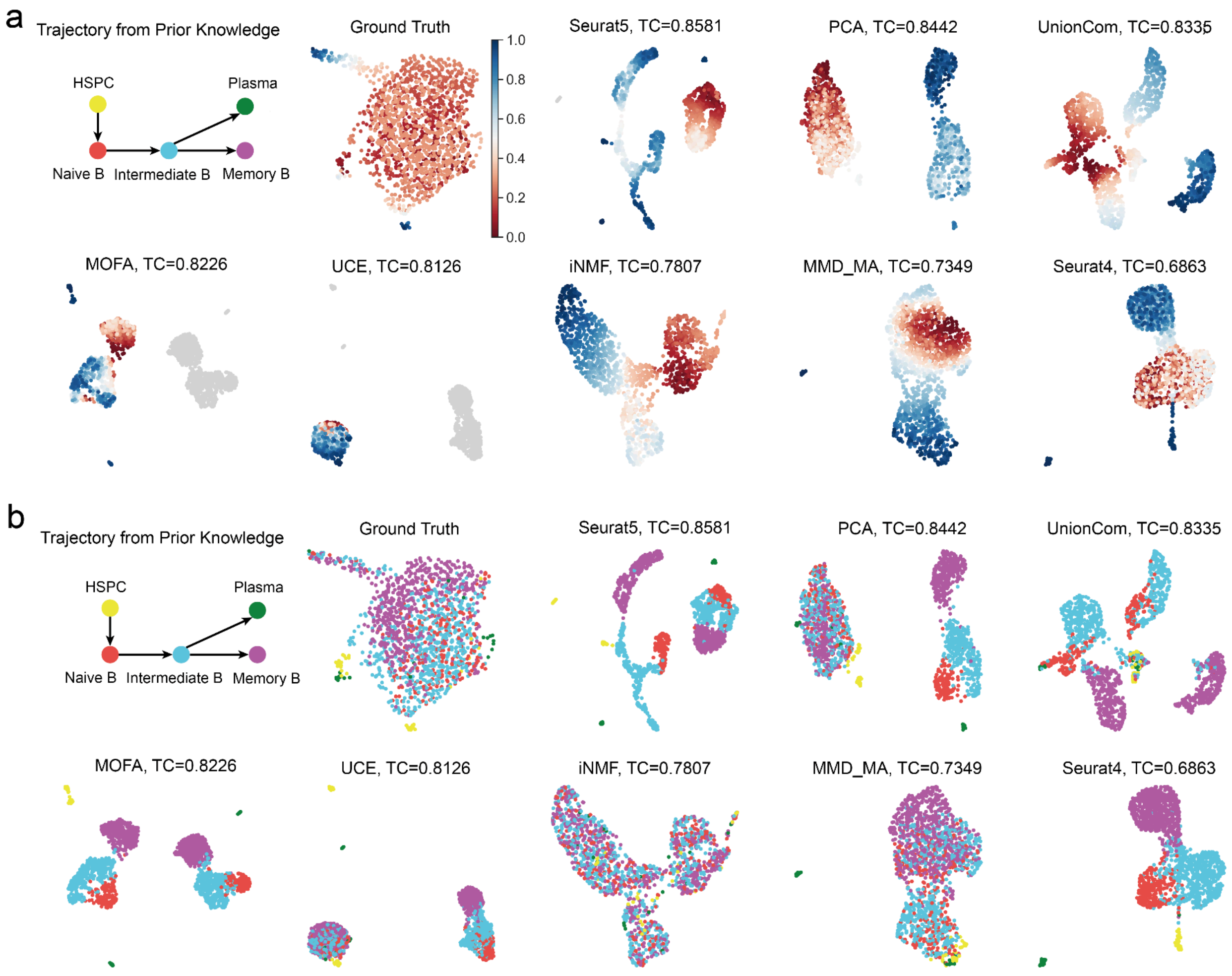

Figure R5. Figure 4 a,b in the manuscript

Added sentences:

[Section 2.4] Additionally, we analyze TC scores across major and minor cell type groups to investigate whether cell population abundance affects trajectory inference performance. Interestingly, methods fall into two groups in Supplementary Figure 6: those performing better on major cell types and those excelling with minor populations, suggesting that cell type abundance is not the primary determinant of trajectory conservation capability. For detailed trajectory analysis, we examine immune cell differentiation pathways with HSPC as the root cell type, expecting ideally to observe T cell subtypes (CD8 TEM 1/2, CD4/CD8 Naive, CD4 TCM/TEM) and B cell lineages (Naive, Intermediate, Memory B) distributed at opposite ends of the bidirectional development trajectory. In Fig. Supplementary Figure 7, we observe that the pseudo-time inference result of MOFA has a clear color change trend similar to the ground truth, with HSPC around dark red and B cells around blue. Within the groups, naive T cells are found closer to the HSPC cluster. scVI, Seurat4, bindSC, GLUE, and

Deepmaps also preserve the trajectory and align well with biological interpretation. The color and size of each group are relatively consistent between the ground truth and Deepmaps, likely due to its multi-head graph transformer architecture, which effectively models biological networks. We then examine a unidirectional, well-characterized B cell developmental lineage (HSPCs → Naive B → Intermediate B → Memory B → Plasma cells). Fig. 4 a. shows the UMAP visualizations of pseudotime inference results from the top eight performing methods, alongside cell type annotations. Among these, Seurat5, PCA, UnionCom, MOFA, and UCE achieved the highest TC scores. For reference, the canonical trajectory derived from prior biological knowledge is also shown. Notably, the embedding generated by Seurat5 clearly captures the developmental progression from Naive B to Memory B cells. Overall comparison of all paired scenarios can be found in Fig. 4 c.

[Caption of Supplementary Figure 6] **Trajectory conservation scores of major and minor cell type groups of the PBMC-10x dataset. a,** Comparison of TC scores calculated across three different subsets: all cells, major cell types, and minor cell types. And methods are grouped based on whether TC scores are higher in major cell types than in minor ones (Group 1: TC_major > TC_minor) or vice versa (Group 2: TC_major < TC_minor).**b,** The violin plot showing the overall distribution of TC scores in three cell groups. These results suggest that the size or abundance of cell types is not the primary determinant of trajectory inference performance, which varies across different methods.

Added Figure:
[Supplementary Figure 6]

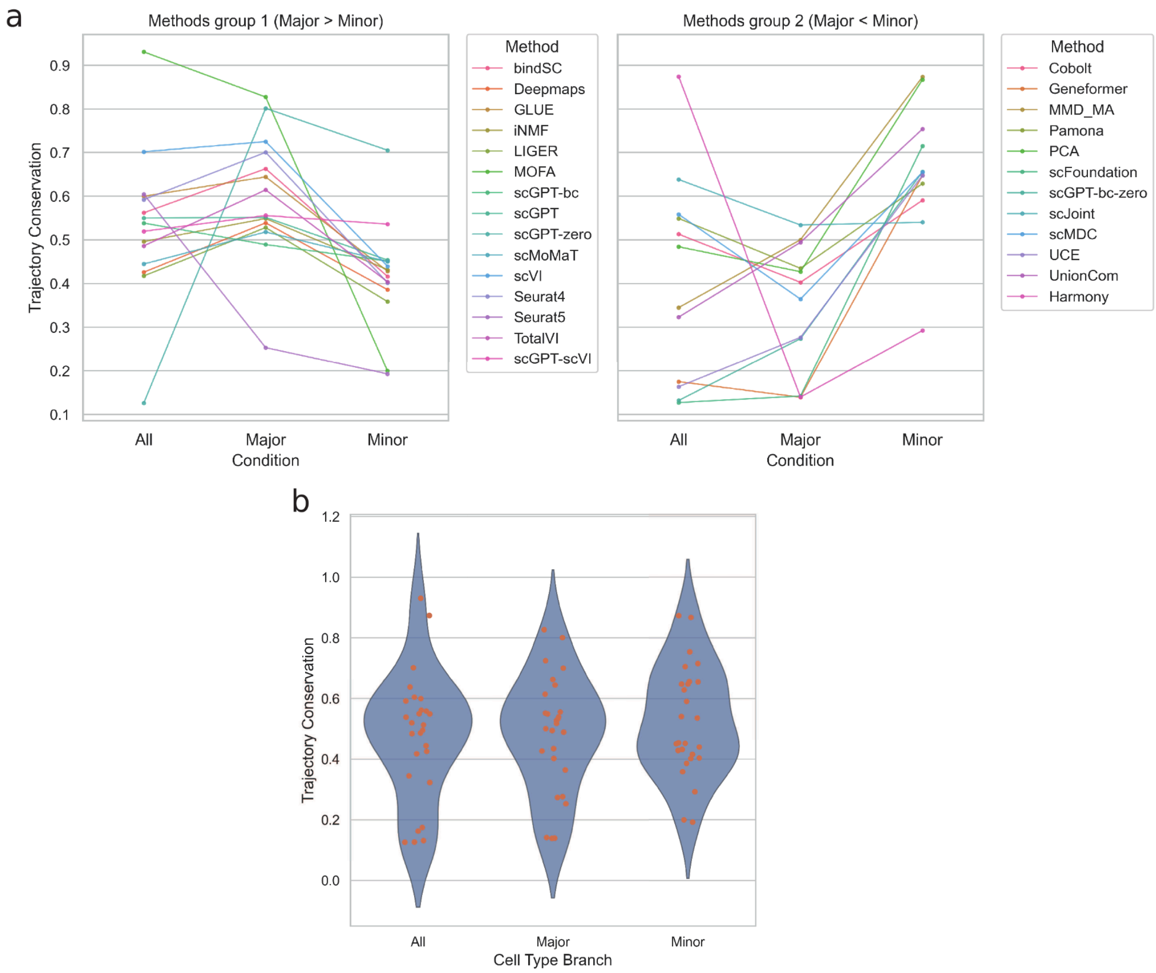

5)Page 14, paragraph 2, the term "scFMs" seems to be a typo, should it be "FMs." Please verify and revise accordingly.

Response: Thank you for pointing out the potential inconsistency with the term "scFMs." We would like to clarify that "scFMs" is the defined abbreviation for "single cell foundation models" that we introduced in the manuscript's introduction. This specific terminology was used to distinguish foundation models designed for single-cell analysis from foundation models in other domains.

However, considering the reviewer's suggestion and to ensure consistency throughout the manuscript, we have decided to standardize our terminology by using "FM" uniformly to refer to foundation models, with the understanding that in our context, these are specifically single-cell foundation models. We have made this change throughout the entire manuscript and updated all relevant figures and tables accordingly. This standardization should improve readability while maintaining the clarity of our discussions regarding foundation models in single-cell analysis.

Original Sentence: Through extensive experiments, we find that single-cell FMs (scFMs) generally underperform compared to DMs for integrating single-cell multi-omics data.

Revised Sentence: Through extensive experiments, we find that FMs generally underperform compared to DMs for integrating single-cell multi-omics data.

Original Sentence: Given that scFMs typically do not perform as well as DMs, we propose the FM+Adaptation layer approach.

Revised Sentence: Given that FMs typically do not perform as well as DMs, we propose the FM+Adaptation layer approach.

Original Sentence: Our analysis reveals that scFMs generally underperform compared to state-of-the-art DMs in single-cell multi-omics data alignment and integration tasks.

Revised Sentence: Our analysis reveals that FMs generally underperform compared to state-of-the-art DMs in single-cell multi-omics data alignment and integration tasks.

6)Page 16, Section 2.7, paragraph 1, Yao-2021 is described as including 69,727 samples and 54,844 samples, better to refer this as “cells”. Please ensure consistent terminology is used throughout the manuscript.

Response: Thank you for highlighting this terminology inconsistency. You are absolutely correct that "cells" is the appropriate term to use when referring to single-cell data, rather than "samples." We have carefully revised the manuscript to ensure that "cells" is consistently used when describing the individual data points in single-cell datasets.

Original Sentence: The largest unpaired dataset, *Yao-2021*, includes 69,727 scRNA-seq samples and 54,844 snATAC-seq samples. Several methods, including GLUE, LINGER, Seurat4, scVI, iNMF, Cobolt, and scJoint, can process this large dataset. Notably, scJoint and GLUE achieve MAP scores of 0.8623 and 0.8982, respectively. The paired dataset Ma-2020 contains 32,321 samples.

Revised Sentences: The largest unpaired dataset, *Yao-2021*, includes 69,727 scRNA-seq cells and 54,844 snATAC-seq cells. Several methods, including GLUE, LINGER, Seurat4, scVI, iNMF, Cobolt, and scJoint, can process this large dataset. Notably, scJoint and GLUE achieve MAP scores of 0.8623 and 0.8982, respectively. The paired dataset Ma-2020 contains 32,321 cells.

Reviewer #1 (Remarks on code availability):

This is a benchmarking paper, and the authors have shared their benchmarking code on GitHub. I went through the public repository, the documentation is clear, but it doesn't include detailed instructions on how to install all the required packages. The 'Directory structure' section in the main README mentions an environment YAML file, but I couldn't find it in the repository. Since I'm already familiar with these packages, I was able to install them one by one. However, for someone new to this field, setting up and running the code for the first time might be a challenge. Overall, the repository is a great resource for the community, but it would be much better if it included clear package installation instructions and an accessible environment file.

Response:
We appreciate the reviewer's comment regarding the clarity and accessibility of the code setup. To address this, we have summarized the software environments used for each method in the TableR3 below, including the Python/R version and the corresponding virtual environment:

Table R3. Virtual environments of SCMBench.

Virtual Envs	Python/R Version	Methods
env1	python=3.8	cobolt;GLUE;Harmony;MMD-MA;MOFA;Pamona;PCA;scJoint;scMDC;scMoMaT;UCE;UnionCom
env2	python=3.11	Geneformer;scFoundation;scVI;TotalVI
env3	python=3.10	scGPT
env4	R=4.3.3	bindSC;Deepmaps;iNMF;liger;Seurat4;Seurat5

To improve reproducibility, we have provided installation scripts for each of the four environments mentioned above. These are included as four separate .sh files in our GitHub repository. Users can execute these scripts directly to set up the environments, or refer to them for manual installation as needed. Detailed instruction can be found in our github repository: [https://github.com/ml4bio/SCMBench/tree/main .](https://github.com/ml4bio/SCMBench/tree/main)

Reviewer #2 (Remarks to the Author):

I co-reviewed this manuscript with one of the reviewers who provided the listed reports. This is part of the Nature Communications initiative to facilitate training in peer review and to provide appropriate recognition for Early Career Researchers who co-review manuscripts.

Reviewer #3 (Remarks to the Author):

The manuscript, "SCMBench: Benchmarking Domain-specific and Foundation Models for Single-cell Multi-omics Data Integration," presents a thorough evaluation of 22 integration methods (DMs and FMs) using diverse datasets and metrics, including integration accuracy, biomarker detection, trajectory inference, and batch effect correction. It demonstrated the limitations of FMs, which often underperform DMs and proposed a "FM + Adaptation Layer" approach to improve FM performance. However, I found several significant issues regarding the leverage, evaluation, and adaptation of single-cell foundation models (scFMs) in this benchmarking study, which diminish the paper's credibility and potential impact on the scFMs community.

Response: Thank you for your valuable feedback. We understand your concerns about our scFM evaluation approach. In fact, we previously recognized that FMs may underperform in certain scenarios, which is why we implemented three different FM approaches under different usage conditions. We would like to clarify that our study's primary goal is to provide an unbiased, user-oriented benchmark of existing foundation models from the perspective of non-developers, rather than to develop novel FM-based integration methods. With the rapid emergence of FMs in single-cell analysis—including versatile models like scGPT that have shown promise across multiple tasks including multi-omics integration—we believe it is essential to benchmark their practical performance against established domain-specific methods. This provides the community with realistic expectations of current FM capabilities and guides method selection for typical users.

Our approach follows established practices from previous studies. For scGPT fine-tuning, we followed the original scGPT protocol by creating new tokens for scATAC-seq peaks while inheriting trained gene embeddings for RNA data, maintaining proper modality distinction as described in their paper. For activity matrix conversion, we adopted the standard approach used by previous methods to enable foundation models to work with scATAC-seq data.

Our focus is evaluating the practical utility of these models for end users who seek to apply FMs without extensive customization or retraining. Given that current FMs are predominantly pretrained on scRNA-seq data, non-specialist users have limited viable options for scATAC-seq data, making regulatory activity inference a reasonable strategy adopted by previous studies. This represents a realistic scenario for assessing FM performance across modalities under minimal adaptation—the maximum effort feasible for typical users.

Thank you for your constructive criticism regarding our manuscript. We have addressed your concerns about the evaluation and adaptation of single-cell foundation models through several substantial improvements:

1. **Enhanced scATAC-seq processing documentation**: We have added detailed descriptions of our gene activity matrix calculation methodology along with comprehensive code. We've included additional analyses (Supplementary Figure 1) validating the alignment between ATAC-derived activity scores and original data.
2. **Improved FM implementation transparency**: Following your suggestions we conducted extensive hyperparameter search for layer selection and explored whether to load pretrained modules. We updated our GitHub repository with comprehensive documentation for all FMs implementations.
3. **Completed FM adaptation benchmarking:** We've fully integrated our FM-scVI adaptation into the benchmarking across all metrics, allowing direct comparison with domain-specific methods.
4. **Addressed cross-species concerns**: Following your feedback, we've recognized the limitations of applying human-pretrained models to mouse data. The fixed vocabulary constraints and limited ortholog mappings created an inequitable comparison. We've removed these problematic direct cross-species comparisons and focused on UCE and adaptation approaches that better address these limitations.
5. **Added reference-based validation**: We have incorporated biomarker evaluation using ground truth markers from the CellMarker 2.0 database and expanded our epigenomic assessment with two new scores for differentially accessible regions and enriched transcription factor binding motifs, creating a more comprehensive framework spanning both transcriptomic and epigenomic features.

These revisions provide a more balanced assessment of foundation models in multi-omics integration and enhance the reproducibility and credibility of our benchmarking study. We appreciate your thoughtful critique which has helped strengthen the manuscript.

1. A primary concern lies in the leverage of scFMs on scATAC data, as these models are originally designed and pretrained on scRNA-seq data only. The authors stated that “although scGPT is the only specialized FM for multi-omics integration, other FMs can also be adapted for this purpose by analyzing cell embeddings.” However, neither the manuscript nor the GitHub repository provides sufficient technical details or a clear rationale for adapting scFMs to handle scATAC data. The manuscript describes the approach as “Due to the limitations imposed by the fixed tokens of the pre-trained large model, we are unable to directly input the peak features of single-cell ATAC-seq data. Instead, we employ a strategy similar to those used in Deepmaps and scMDC: mapping scATAC-seq data onto scRNA-seq data. This involves assigning regulatory activity scores or weights to scRNA-seq cells based on the accessibility or openness of regulatory elements observed in the scATAC-seq data. This process allows us to obtain the corresponding activity matrix, which subsequently serves as the input for the FMs.” But it is unclear how this activity matrix is calculated or whether its data distribution, signal-to-noise ratio, and biological relevance align with those of RNA expression. Without further justification, this leverage appears ad hoc, and the meaningfulness and fairness of benchmarking scFMs in this manner, particularly in the zero-shot setting, are doubtful. The underperformance of scFMs on diverse tasks further suggests that such an approach does not fully realize their potential. The study also fine-tuned scGP with scATAC data and claimed that “This performance discrepancy may be attributed to the possibility that the fine-tuning process for scGPT inadvertently mixes DNA-related information with RNA-related information, potentially disrupting the specific RNA-related details crucial for biomarker detection.” This explanation further confirms the incompatibility of the proposed usage of scFMs with scATAC data. Besides, the provided GitHub repository lacks critical scripts and documentation for replicating the scFMs setup described in the study. The repository includes only the official scRNA-seq interface for zero-shot scGPT/UCE/Geneformer, and the fine-tuning script focuses on integrating RNA and protein expression, not RNA and ATAC. No script for scFoundation is provided, and several links in the README file lead to inaccessible pages. This lack of accessible and complete source code significantly hinders the reproducibility and assessment of the study’s claims.

Response: We are thankful for your insightful comments regarding the application of foundation models on scATAC-seq data. Your concerns are indeed valid and align precisely with what we found in our manuscript through comprehensive evaluation across various experimental settings. However, we would like to clarify that these findings are not a weakness of our paper, but rather exactly what we aim to disclose to the readers. We agree with the suggestions that the rationality of the leverage of FMs

on scATAC data and the process of activity matrix calculation should be demonstrated clearly. The corresponding code needs to be complemented and improved as well.

We would like to clarify that the primary goal of our study is not to develop a novel FM-based integration method, but rather to provide an unbiased, user-oriented benchmark of existing FMs from the perspective of non-developers. Our motivation for comparing FMs with domain-specific methods stems from the rapid emergence of foundation models in this field [1-5]. Among these, scGPT has demonstrated particularly strong performance due to its versatility across multiple single-cell analysis tasks, including cell type annotation, gene regulatory network inference, multi-omics integration, perturbation analysis, and reference mapping. Notably, scGPT has explored multi-omics integration tasks, showing promising potential in cross-modal applications. As an increasing number of FMs continue to emerge in the single-cell field, we believe it is essential to benchmark their performance against established domain-specific methods to provide the community with realistic expectations of their current capabilities and guide users in method selection.

Our focus is on evaluating the practical utility of these models for end users—those who seek to apply FMs for data analysis and integration tasks without engaging in extensive model customization or retraining. Given that current FMs are predominantly pretrained on scRNA-seq data, users without FM development expertise are left with only limited viable options when working with scATAC-seq data. In such cases, a reasonable and practical strategy—adopted in previous studies such as DeepMAPS and scMDC—is to map scATAC-seq profiles onto RNA space through regulatory activity inference, enabling zero-shot inference or simple fine-tuning using existing settings. This reflects the maximum level of effort feasible for non-specialist users and represents a realistic scenario for assessing the performance of FMs across modalities. As stated in comment (5) by Reviewer 4: the requirement of adaptation learning (rather than simple prompt engineering) has already contradicted the conclusion of the generalizability of FMs, our benchmarking results also show that a normal user is not recommended to use FMs in this task without any customized adaptation or engineering. And we have made efforts to simulate realistic usage scenarios and assess the maximum performance that typical users can achieve with FMs under minimal adaptation.

Q1: But it is unclear how this activity matrix is calculated or whether its data distribution, signal-to-noise ratio, and biological relevance align with those of RNA expression.

Response: We appreciate the reviewer's detailed concerns regarding our approach to leveraging FMs for scATAC-seq data integration. This is indeed a critical point that requires clarification. The foundational challenge we faced is that current single-cell

FMs are primarily designed for and pre-trained on transcriptomic data, with limited direct applicability to chromatin accessibility data. Our strategy was to address this limitation through a principled approach to data transformation.

We acknowledge that our original description in the manuscript 'To ensure fairness among methods requiring an activity matrix, we standardize the calculation using the ATACCalculateGenescore from the MAESTRO library, following the implementation approach of DeepMAPS [42].' lacked sufficient detail for reproducibility. To clarify, we calculate the activity matrix from original scATAC-seq data using the function 'ATACCalculateGeneScore' from the released MAESTRO package. Specifically, the activity matrix is inferred by the regulatory potential (RP) model. It assumes that the effect of a scATAC-seq peak on the expression of a given gene is independent and additive, but follows an exponential decay with the distance from the peak to the transcription start site (TSS). The sum of weighted peak signals around TSS and exon of target genes is the final activity score. This approach is grounded in established biological principles linking chromatin accessibility to gene regulation, and uses a well-validated method from the literature rather than an ad hoc transformation. We selected this method specifically because it preserves the biological relationship between chromatin accessibility and gene regulation while converting the data into a format compatible with FM processing. And the activity matrix calculated by regulatory score inference is also a common strategy in other methods, like Seurat4 [6] and GLUE [7]. All preprocessing scripts can be found in our Github under folder:

https://github.com/ml4bio/SCMBench/tree/main/data/preprocessing

To further examine the alignment of ATAC-derived activity scores with original RNA and ATAC data, we conducted additional analyses using both MAESTRO and Seurat4 methods, which we have added to the supplementary materials. Figure R6 shows the visualization of results. As demonstrated in Figure R6a, both MAESTRO and Seurat4-derived activity matrices show consistent clustering patterns with the original RNA UMAP, indicating that both methods preserve biologically meaningful cell-type structures. The gene-wise average expression comparison between RNA and ATAC-derived activity shows that these two modalities are not perfectly aligned (Figure R6b). The Pearson correlation coefficients between RNA expression and ATAC-derived activity across all cells are comparable for both Seurat4 (Figure R6c) and MAESTRO (Figure R6d), with mean values around 0.2. This discrepancy is expected, as gene expression is regulated by complex mechanisms beyond chromatin accessibility near the TSS, such as post-transcriptional regulation and enhancer-promoter interactions. Nevertheless, the activity matrix retains biologically meaningful information, capturing clustering structures and cell-type-specific signatures for key marker genes such as *ZEB2* in CD14 Monocytes and *CCL5 in CD8 Effector Memory T cells* (Figure R6a, e).

To assess how much original ATAC signal is retained, we calculated the peak retention ratio—defined as the proportion of peaks in the activity matrix that overlap with original ATAC peaks within a defined window around the TSS. As shown in Figure R6f, using the same decay distance applied during activity score computation (10,000 bp), the retention ratio reaches 100%, indicating that the transformation process preserves proximal regulatory features encoded in the ATAC-seq data. Importantly, as shown in Figure R6g, MAESTRO retains significantly more features compared to Seurat4 (which only preserves ~1.2k features), making our choice of MAESTRO for activity matrix calculation well-justified for comprehensive downstream analysis.

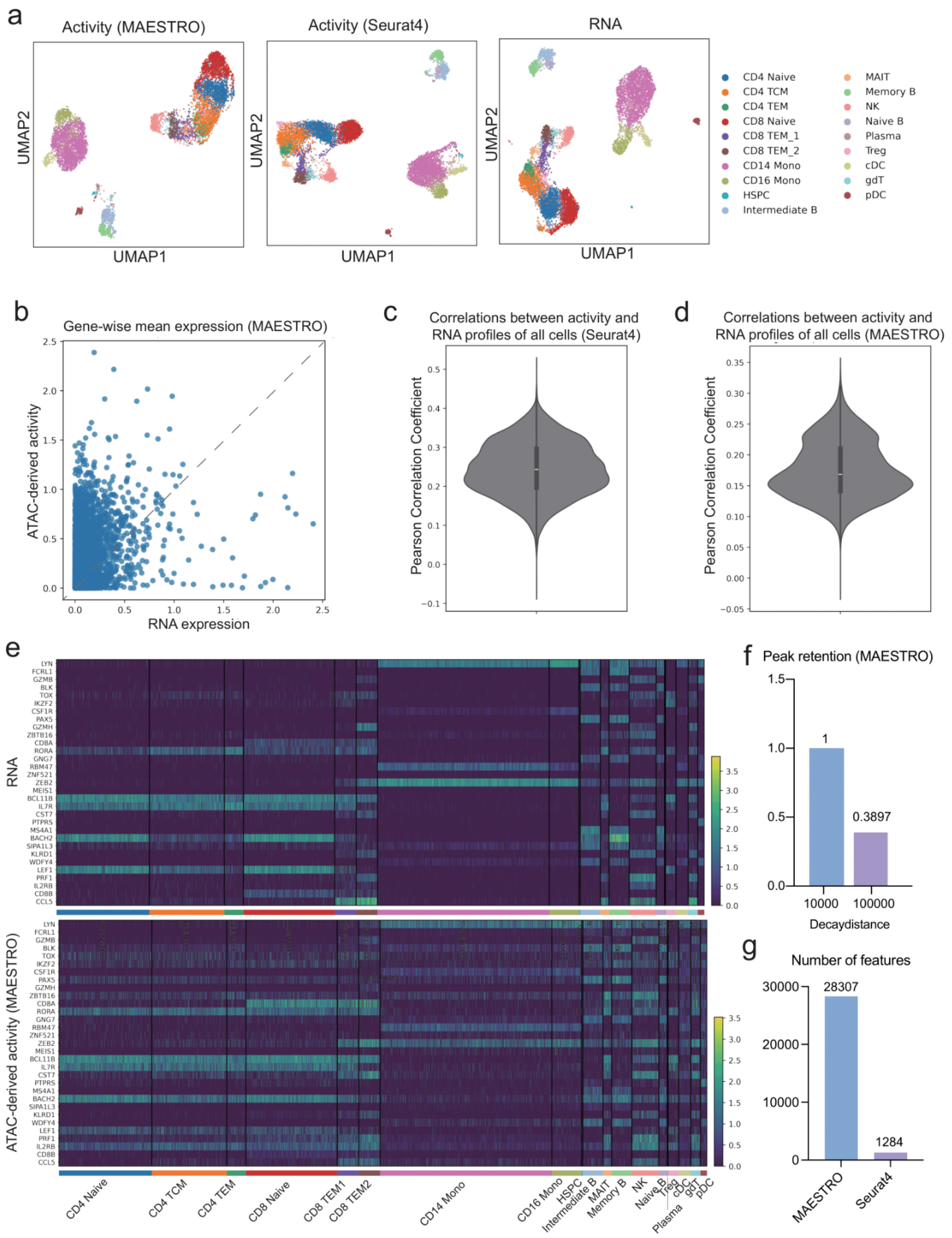

Figure R6: Supplementary Figure 1 in the manuscript

Q2:The underperformance of scFMs on diverse tasks further suggests that such an approach does not fully realize their potential. The study also fine-tuned scGPT with scATAC data and claimed that "This performance discrepancy may be attributed to the possibility that the fine-tuning process for scGPT inadvertently mixes DNA-related information with RNA-related information, potentially disrupting the specific RNA-related

details crucial for biomarker detection." This explanation further confirms the incompatibility of the proposed usage of scFMs with scATAC data.

Response: We appreciate the your concern and would like to clarify our approach. We agree with your assessment that current methods, including our adaptation approach, do not fully realize the potential of foundation models. We have expanded our discussion to include this observation.

We would like to emphasize that the primary goal of our study is not to develop a novel FM-based integration method to fully realize FMs' potential, but rather to provide an unbiased, user-oriented benchmark of existing FMs from the perspective of non-developers. We employed two complementary strategies to comprehensively evaluate FMs with scATAC-seq data:

For scGPT fine-tuning, we create new tokens specifically for scATAC-seq peaks to maintain modality distinction, allowing the model to learn ATAC-specific representations while preserving the separation between different data modalities. This approach is consistent with the original scGPT paper, which states: "Due to the presence of additional ATAC and/or protein tokens, we inherited the trained gene embeddings for RNA data only and trained the additional token embeddings and the rest of the model from scratch." We followed this exact protocol to ensure proper modality handling.

For zero-shot evaluation and our FM-scVI adaptation, we convert scATAC-seq data to gene activity matrices—a standard practice widely adopted by domain-specific methods in this field, not our innovation. This approach enables us to leverage the pre-trained tokens and vocabulary that the foundation models were originally trained on, allowing us to assess their zero-shot capabilities without requiring extensive retraining.

Additionally, we introduced FM-scVI precisely to address the inherent limitations of zero-shot evaluation, where FMs lack task-specific optimization for multi-omics integration. While domain-specific methods are explicitly designed and trained for integration tasks, foundation models in zero-shot mode have no such specialized training. Our adaptation approach allows for appropriate task-specific adjustments while leveraging FMs' biological knowledge, demonstrating that with proper domain adaptation, foundation models can achieve competitive performance in multi-omics integration.

These three approaches provide a comprehensive evaluation framework: the fine-tuning approach tests the models' adaptability to new modalities, the zero-shot approach evaluates their generalization using existing representations, and our adaptation framework demonstrates how foundation models can be effectively enhanced for

specific integration tasks. The competitive results of our adaptation framework indicate that FMs can be effectively leveraged for this task when appropriately adapted to the specific requirements of multi-omics integration. Together, they offer a thorough assessment of how FMs can be practically applied to multi-omics integration tasks by typical users.

Q3:Besides, the provided GitHub repository lacks critical scripts and documentation for replicating the scFMs setup described in the study. The repository includes only the official scRNA-seq interface for zero-shot scGPT/UCE/Geneformer, and the fine-tuning script focuses on integrating RNA and protein expression, not RNA and ATAC. No script for scFoundation is provided, and several links in the README file lead to inaccessible pages.

Response: Thank you for pointing out the issues in the GitHub repository. We sincerely apologize for the missing scripts, incomplete documentation, and incorrect links that hindered replication of the scFMs setup as described in our study. We acknowledge these oversights and have now thoroughly updated the repository. The revised version includes all necessary scripts for reproducing the zero-shot and fine-tuning experiments, including those for RNA–ATAC integration and scFoundation. You can find the scripts in our github below:

- scGPT-zero: https://github.com/ml4bio/SCMBench/tree/main/methods/scGPT-zero
- scGPT-finetune: *https://github.com/ml4bio/SCMBench/tree/main/methods/scGPT-ft*
- UCE: *https://github.com/ml4bio/SCMBench/tree/main/methods/UCE*
- Geneformer: *https://github.com/ml4bio/SCMBench/tree/main/methods/Geneformer*
- scFoundation: *https://github.com/ml4bio/SCMBench/tree/main/methods/scFoundation*
- FM-scVI: *https://github.com/ml4bio/SCMBench/tree/main/methods/scVI*

There are python and example shell scripts in each method’s folder for reference. Moreover, we have also corrected all broken links and provided clearer documentation to guide users through the installation, inference and evaluation processes:

- Installation: *https://github.com/ml4bio/SCMBench/tree/main/environments*
- Inference: *https://github.com/ml4bio/SCMBench/blob/main/README.md*
- Evaluation: *https://github.com/ml4bio/SCMBench/blob/main/README.md*

We appreciate your feedback and have taken it seriously to ensure better accessibility and reproducibility.

[1] Theodoris, C. V. et al. Transfer learning enables predictions in network biology. Nature 618, 616–624 (2023).
[2] Cui, H. et al. scgpt: toward building a foundation model for single-cell multi-omics using generative ai. Nature Methods 1–11 (2024).
[3] Hao, M. et al. Large-scale foundation model on single-cell transcriptomics. Nature Methods 1–11 (2024).
[4] Rosen, Y. et al. Universal cell embeddings: A foundation model for cell biology. bioRxiv 2023–11 (2023).
[5] Heimberg, Graham, et al. "A cell atlas foundation model for scalable search of similar human cells." *Nature* 638.8052 (2025): 1085-1094.
[6]. Hao Y, Hao S, Andersen-Nissen E, et al. Integrated analysis of multimodal single-cell data[J]. Cell, 2021, 184(13): 3573-3587. e29.
[7]. Cao Z J, Gao G. Multi-omics single-cell data integration and regulatory inference with graph-linked embedding[J]. Nature Biotechnology, 2022, 40(10): 1458-1466.

Added sentences:
[Section 2.1] Due to the token constraints of pre-trained foundation models, which are primarily designed to process gene expression data, we cannot directly input scATAC-seq peak features. Instead, we transform chromatin accessibility data into a gene-centric format using a biologically informed approach implemented in the MAESTRO package [44]. This method calculates gene activity scores through a regulatory potential model that weights peak signals based on their distance from transcription start sites, reflecting the established relationship between chromatin accessibility and gene regulation. Our supplementary analyses (Supplementary Figure 1) further validate this approach, confirming that the activity matrix successfully preserves cell-type-specific marker gene signals, maintains biological clustering structure, and retains chromatin accessibility features critical for integration. The resulting activity matrix is compatible with foundation model processing, enabling integration while preserving the biological relationship between chromatin accessibility patterns and gene expression regulation.

[Caption of Supplementary Figure 1] Analysis and comparison of ATAC-derived activity matrix of PBMC-10x dataset.
a. UMAP visualization of both the RNA expression and the corresponding ATAC-derived gene activity matrix from MAESTRO and Seurat4.

b. Scatter plot comparing gene-wise average expression between RNA and ATAC-derived activity (MAESTRO) across all cells. Each dot represents one gene.

c-d. Pearson correlation coefficients between RNA expression and ATAC-derived activity across all cells of Seurat4 (**c**) and MAESTRO (**d**). It reflects the intrinsic difference between chromatin accessibility and gene expression, suggesting that gene expression is influenced by factors beyond local chromatin accessibility near transcription start sites (TSSs).

e. Comparison of expression patterns of differentially expressed genes in each cell type, between RNA and ATAC-derived activity matrices (MAESTRO). The activity matrix captures the expression patterns of genes with strong cell-type specificity, such as ZEB2 in CD14+ monocytes and CCL5 in CD8+ TEM cells.

f. Peak retention analysis of the ATAC-derived activity matrix (MAESTRO) relative to the original ATAC-seq peak matrix. For each non-zero gene in the activity matrix, TSSs were identified and overlapping ATAC-seq peaks within a defined genomic window (decay distance) were retrieved. The peak retention ratio is defined as the proportion of matched peaks from the activity matrix relative to all non-zero peaks in the original ATAC-seq data. When the decay distance was set to 10,000 bp, the retention ratio reached 100%, whereas a decay distance of 100,000 bp resulted in a retention ratio of 38.97%. These results suggest that the transformation process preserves local regulatory information captured by ATAC-seq, and that the choice of decay distance substantially influences the resulting activity profiles.

g. Comparison of the number of features in the ATAC-derived activity matrix between MAESTRO and Seurat4.

[Discussion] **FMs in Multi-omics Integration** While FMs have demonstrated transformative potential in other fields, their capabilities in single-cell biology remain underexplored. Our benchmark results, alongside published literature, suggest that current single-cell FMs have not yet been adequately harnessed for this domain. While generally underperforming compared to specialized DMs in overall integration accuracy, FMs demonstrate unique strengths in versatility and efficiency. Their versatility enables adaptation across diverse single-cell tasks with the same architecture, while their efficiency advantages include both reduced training requirements and computational feasibility on large-scale datasets where many specialized DMs become prohibitively expensive or fail to complete. However, their struggle with multi-modal integration in the zero-shot setting underscores the necessity for appropriate adaptation strategies when handling different data modalities. Most FMs are predominantly trained on human data, leading to cross-species bias when generalizing to other organisms. Even UCE, which attempts to address this by training on diverse tissues and species, suffers from data imbalance issues in its training atlas. The exceptional performance of UCE on mouse developmental data suggests that species-specific pre-training may confer advantages

for certain biological contexts. To address these limitations while leveraging FMs' rich biological knowledge, we propose the FM + AdaptationLayer approach. Specifically, we develop FM-scVI, a hybrid architecture that combines FM embeddings with scVI's variational autoencoder framework. We implement this adaptation as a standalone method and incorporate it into our comprehensive benchmarking pipeline, evaluating it alongside traditional deep learning methods across all metrics and datasets. Our results demonstrate that this strategy effectively bridges the gap between FMs' biological knowledge and the specific requirements of multi-omics integration tasks, showing particular improvements in biomarker detection and trajectory conservation while maintaining computational efficiency. However, further refinement and validation are necessary to fully realize its potential.

2. The authors proposed an adaptation method for scFMs to integrate scRNA and scATAC data, which involved leveraging scVI's multi-omics integration functionality to fuse embeddings derived from zero-shot scFMs (scGPT and UCE were tested). This approach, termed "scFMs+Adaptation layers," was validated on two datasets by comparing the harmonized embeddings from scVI with those generated by various scFM configurations, including zero-shot scGPT/UCE, partially fine-tuned scGPT, and fully retrained scGPT. The study concluded that the joint embeddings from scVI outperformed those from the mechanically applied scFMs. However, several issues remain: i) The fine-tuning strategy in this comparison updated only the top-4 layers of scFMs, differing from the official full fine-tuning approach. The rationale for selecting these specific layers and ensuring their compatibility with the remaining frozen layers is not justified, raising concerns about the robustness of the proposed method. ii) Again, the source code for these "scFM adaptations" is incomplete. The provided GitHub repository includes only a script for scVI-based integration using precomputed RNA and ATAC embeddings, without details of their embedding calculations. iii) If the "scFMs+Adaptation layers" approach represents an optimal integration strategy for scRNA and scATAC using scFMs, it should have been included as part of the benchmarking study rather than presented as a proof-of-concept in Section 2.6.

Response: We greatly appreciate your careful review of our scFMs' fine-tuning strategies. Your concerns help us to refine our benchmark experiments. We will address them respectively below.

Q1: The fine-tuning strategy in this comparison updated only the top-4 layers of scFMs, differing from the official full fine-tuning approach. The rationale for selecting these specific layers and ensuring their compatibility with the remaining frozen layers is not justified, raising concerns about the robustness of the proposed method.

Response: In the experiment setting, since our goal is not to engineer the best possible adaptation, but rather to benchmark how non-developer users can leverage scFMs with minimal effort. Therefore, we follow the official scGPT tutorial (https://github.com/bowang-lab/scGPT/blob/main/tutorials/Tutorial_Multiomics.ipynb) that fine-tune only the top 4 transformer layers, which is a common practice among regular users. To more comprehensively investigate how the fine-tuning strategy influences integration performance, we further benchmarked different configurations: varying the number of trainable transformer layers, comparing models initialized from pretrained weights versus training from scratch, and using two pretrained versions of scGPT (trained on whole human data and blood data, respectively). Notably, consistent with the official fine-tuning guidance, we always load the pretrained token embeddings. Additionally, unlike the default setting, we set data_is_raw=True to better match the characteristics of our benchmark datasets. Four metrics of integration accuracy are presented in Table R4. The fine-tuned version, using human pretrained model and 8 loaded transformer layers, performs the best with overall score as 0.591, and is bolded in Table R4. Nonetheless, the discrepancy between different fine-tuning settings is subtle, and all of them can achieve an overall score of 0.56. We also add this additional benchmark result in the Supplementary material.

To make a fair comparison between scGPT-finetune and other embeddings, the best version (loaded 8 pretrained encoder layers of human model) is leveraged as the representation of scGPT-finetune. The updated results are included in Figure 2 (Figure R1) and Figure 6 in the manuscript (Figure R7).

Table R4. Supplementary Table 4 in the manuscript.

Pretrain-model	#Transformer Layers	Load pre-trained encoder	MAP	NMI	ASW	ARI	Overall
Human	4	FALSE	0.7262	0.6731	0.5677	0.3454	0.5781
		TRUE	0.717	0.6621	0.5692	0.3262	0.568625
	8	FALSE	0.704	0.6875	0.567	0.3428	0.575325
		**TRUE**	**0.7421**	**0.6942**	**0.5661**	**0.3619**	**0.591075**
	12	FALSE	0.6992	0.6462	0.5628	0.333	0.5603
		TRUE	0.7245	0.6828	0.5697	0.327	0.576
Blood	4	FALSE	0.7221	0.6939	0.5658	0.3425	0.581075
		TRUE	0.6899	0.6981	0.5674	0.3451	0.575125
	8	FALSE	0.7192	0.6896	0.5664	0.326	0.5753
		TRUE	0.7242	0.6977	0.5712	0.3289	0.5805

	12	FALSE	0.6984	0.6656	0.5696	0.3528	0.5716
		TRUE	0.7137	0.6972	0.5702	0.358	0.584775

Q2:ii) Again, the source code for these "scFM adaptations" is incomplete. The provided GitHub repository includes only a script for scVI-based integration using precomputed RNA and ATAC embeddings, without details of their embedding calculations.

Response: Thank you for raising this issue of the source code availability. We have updated the GitHub repository and include more documents and scripts of foundation models to guide users:

Documents of foundation models:

scGPT-zero: *https://github.com/ml4bio/SCMBench/tree/main/methods/scGPT-zero*
scGPT-finetune: *https://github.com/ml4bio/SCMBench/tree/main/methods/scGPT-ft*
UCE: *https://github.com/ml4bio/SCMBench/tree/main/methods/UCE*
Geneformer: *https://github.com/ml4bio/SCMBench/tree/main/methods/Geneformer*
scFoundation: *https://github.com/ml4bio/SCMBench/tree/main/methods/scFoundation*
FM-scVI:*https://github.com/ml4bio/SCMBench/tree/main/methods/FM-scVI*

Q3: If the "scFMs+Adaptation layers" approach represents an optimal integration strategy for scRNA and scATAC using scFMs, it should have been included as part of the benchmarking study rather than presented as a proof-of-concept in Section 2.6

Response: Thank you for the insightful suggestion. In response, we have incorporated both FM-scVI and Harmony into our benchmark analyses to provide a more comprehensive comparison across existing integration strategies. The results have been updated accordingly in Figure 2-4 (Figure R1, 8-9) of the revised manuscript.

For the benchmarking, we utilize scGPT-zero embeddings as input for FM-scVI on the human datasets, and UCE embeddings for the mouse datasets, ensuring fair and representative input conditions across species. In this updated comparison, FM-scVI ranks 12th in overall integration accuracy on paired datasets, and 10th on unpaired datasets.

While we acknowledge that the "FMs + adaptation layers" approach demonstrates strong performance, we position it as a proof-of-concept to showcase the flexibility and extensibility of FMs in adapting to multi-modal integration scenarios. Our primary goal in Section 2.6 is to highlight how FMs can serve as a unifying foundation across various downstream adaptation strategies. This distinction complements the benchmarking section rather than substituting it, as it emphasizes methodological innovation without conflating it with standardized benchmarking pipelines.

Added Table [Supplementary Table 4]:

Supplementary Table 4: Comparison between different versions of finetuned scGPT

Pretrain-model	#Transformer Layers	Load pre-trained encoder	MAP	NMI	ASW	ARI	Overall
Human	4	FALSE	0.7262	0.6731	0.5677	0.3454	0.5781
		TRUE	0.717	0.6621	0.5692	0.3262	0.568625
	8	FALSE	0.704	0.6875	0.567	0.3428	0.575325
		**TRUE**	**0.7421**	**0.6942**	**0.5661**	**0.3619**	**0.591075**
	12	FALSE	0.6992	0.6462	0.5628	0.333	0.5603
		TRUE	0.7245	0.6828	0.5697	0.327	0.576
Blood	4	FALSE	0.7221	0.6939	0.5658	0.3425	0.581075
		TRUE	0.6899	0.6981	0.5674	0.3451	0.575125
	8	FALSE	0.7192	0.6896	0.5664	0.326	0.5753
		TRUE	0.7242	0.6977	0.5712	0.3289	0.5805
	12	FALSE	0.6984	0.6656	0.5696	0.3528	0.5716
		TRUE	0.7137	0.6972	0.5702	0.358	0.584775

Revised Figure [Figure 6]:

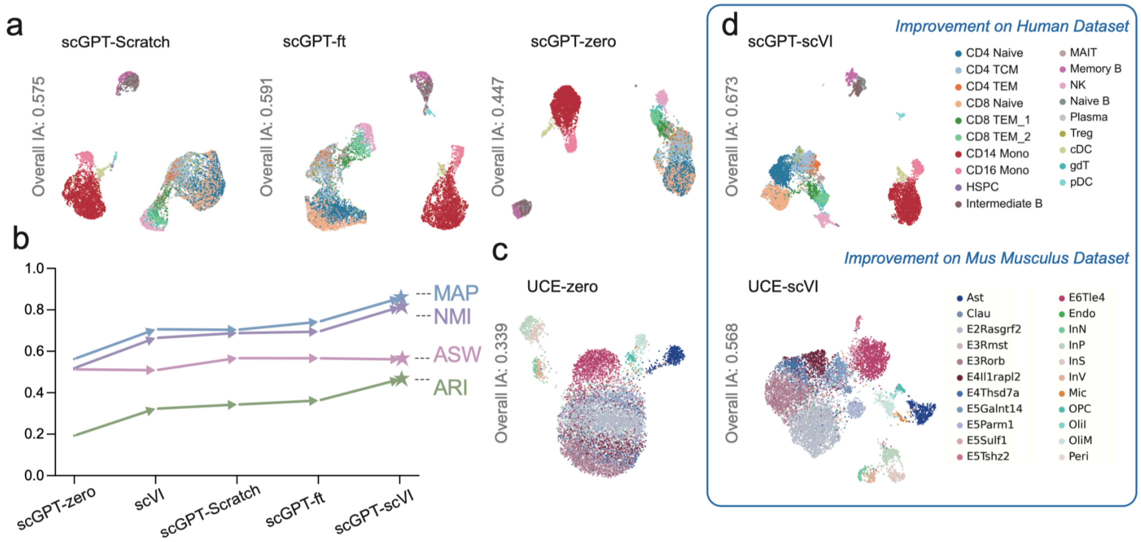

Figure R7: Figure 6a-d in the manuscript

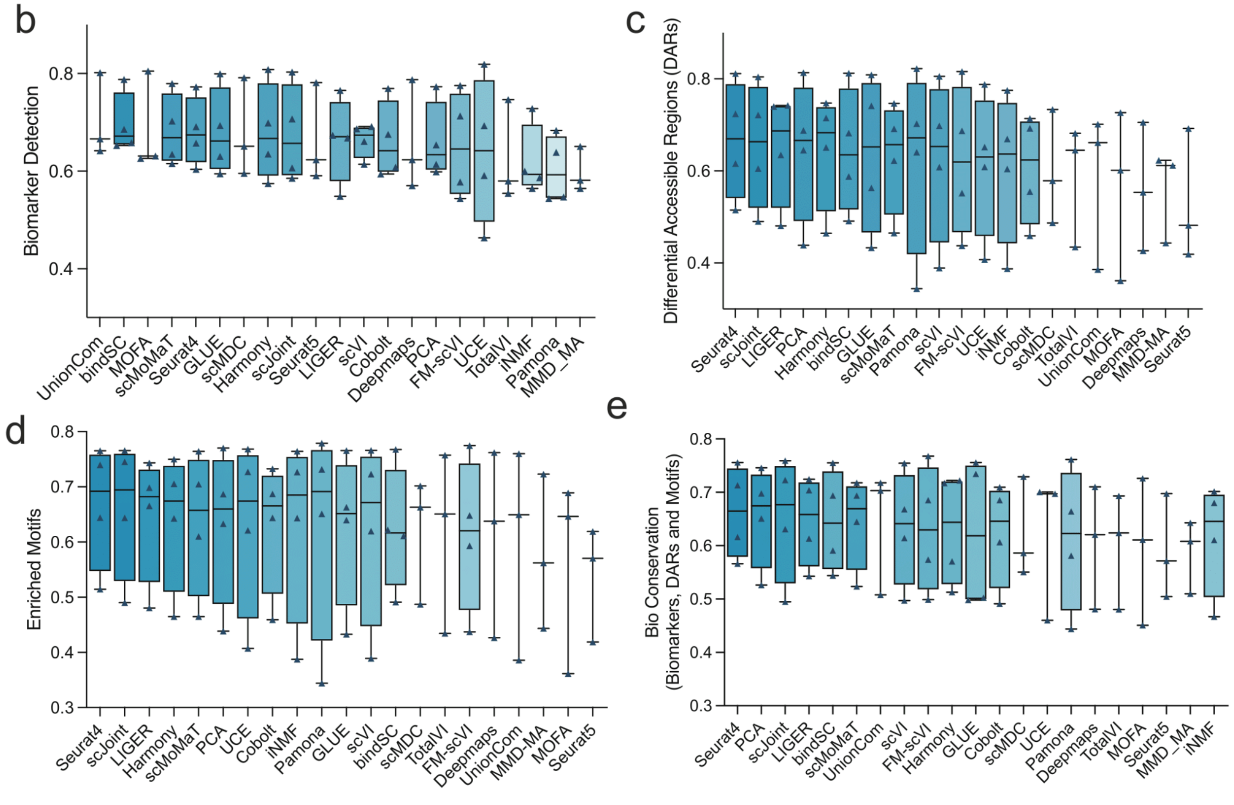

Figure R8: Figure 3 b-e in the manuscript

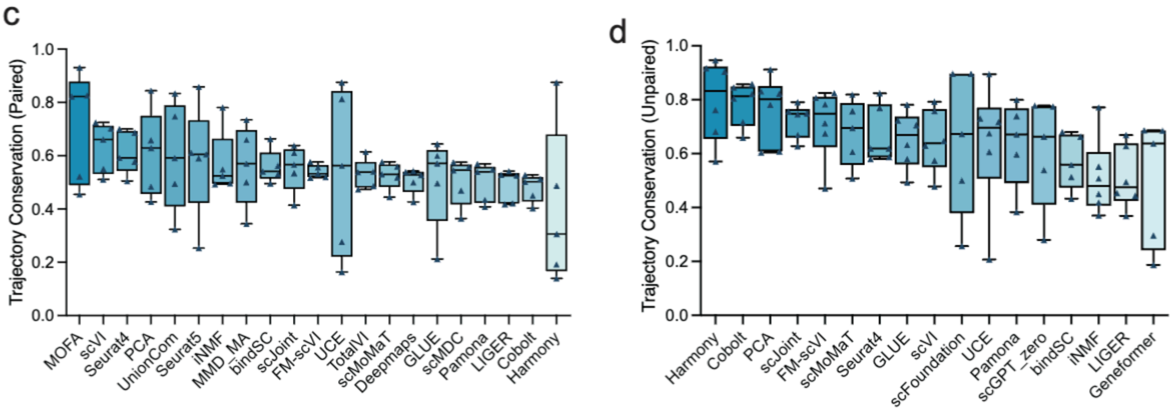

Figure R9: Figure 4 c,d in the manuscript

Revised Sentences:

[Caption of figure 2] Figure 2: Benchmarking results of the integration accuracy. All metrics are the higher the better. a and b. Benchmarking results on the paired and unpaired datasets. Metrics are categorized into three groups: integration accuracy (in red), bio-conservation (in yellow), and batch correction (in green). FM-scVI represents the adapted scGPT on PBMC-10x and the adapted UCE on Ma-2020. scGPT-ft is the fine-tuned version of scGPT, while scGPT-zero is the zero-shot version.

[Section 2.2] To thoroughly investigate how fine-tuning influences integration performance, we benchmarked different depths of transformer layers and compared loading pretrained parameters versus re-training from scratch. We also compared performance using scGPT's whole-human model and blood-specific model. Interestingly, our analysis revealed that pretrained weights did not provide substantial benefits, suggesting that the model's architecture itself plays a crucial role in integration performance. The scGPT blood model generally outperformed the human model under the same settings, suggesting that models fine-tuned with datasets similar in cellu-
lar context to their training data typically exhibit competitive performance. Among foundation models, scGPT and UCE demonstrated superior performance, with our scGPT adaptation (FM-scVI) achieving results comparable to GLUE.

[Caption of figure 3] Figure 3: Comparison of the cellular identity preservation performance of DMs and FMs through biomarker detection. a. Comparative analysis of biomarker detection performance across various computational methods. Heatmaps display the JSI for biomarker detection between methods, with darker red shades representing higher overlap. NK cells in PBMX-10x and ENDO cells in Muto-2021 are taken as examples. Methods are ranked according to their BM score. b-d. Method rankings based on average performance across datasets for the detection of (b)

cell-type-specific biomarkers, (c) differentially accessible regions (DARs), and (d) enriched transcription factor motifs. These rankings highlight the relative effectiveness of each method in preserving biologically meaningful features. e. Box plot comparing overall biological conservation scores across methods. The conservation score is computed as the average detection consistency of cell-type-specific biomarkers, DARs, and enriched transcription factor motifs. The central line in each box represents the median score, with narrower boxes and shorter whiskers indicating greater robustness and consistency across datasets. For FM-scVI, results represent a combination of scGPT-scVI applied to human data and UCE-scVI applied to mouse data as a preliminary species-specific adaptation approach.

[Section 2.3] Several methods, including scGPT-scVI, scMoMaT, and scJoint, show strong recall of known biomarkers alongside high consistency scores, indicating their effectiveness in identifying biologically meaningful cell markers beyond statistical associations. When comparing FM and DM approaches in biomarker detection, we find that FMs demonstrate promising performance in this task. Notably, the zero-shot version of scGPT outperforms many DMs in identifying NK cell biomarkers, demonstrating the strong biological knowledge encoded in pre-trained language models. Interestingly, scGPT's fine-tuned version shows relatively lower performance compared to its zero-shot counterpart, suggesting that introducing information from another omics modality may compromise some of the intrinsic biological knowledge specific to gene expression patterns when attempting to create a joint representation.

Fig. 3 b presents a comprehensive evaluation of biomarker detection performance at the transcriptomic level, incorporating results from both paired and unpaired settings across human and mouse datasets. For this comparison, we exclude FM that could not be applied to mouse data. The FM-scVI results shown represent a combination of scGPT-scVI applied to human data and UCE-scVI applied to mouse data, reflecting species-specific adaptations of our preliminary approach. The ranking reveals that UnionCom, bindSC, and MOFA consistently achieve top performance with relatively stable scores across different conditions. GLUE, Harmony, and scJoint also demonstrate strong results, though with higher variability across datasets and settings. Despite UCE's cross-species capabilities, it achieves lower BM scores when used directly. Our initial adaptation experiments show promise in specific contexts, with scGPT-scVI demonstrating competitive performance for NK cells in human data. However, there remains a considerable gap between foundation model approaches and top-performing DMs, particularly in unpaired scenarios and mouse data. This gap highlights promising directions for future research in adapting foundation models for multi-omics integration across diverse experimental conditions and species.

Figure 3 e presents the overall biomarker detection performance ranking across all three metrics. Consistently, bindSC, Seurat4, PCA, and scJoint demonstrate superior

performance, establishing themselves as effective methods for multi-omics biomarker discovery. Despite moderate transcriptomic performance, UCE achieves results comparable to several DMs, while at the scATAC-seq level, FM-scVI shows comparable effectiveness to established methods like Harmony and GLUE. These findings suggest that while specialized integration methods currently lead in biomarker detection, foundation models demonstrate promising capabilities that could be enhanced through targeted improvements to preserve both transcriptomic and epigenomic signals.

[Section 2.4] When examining unpaired settings (Fig. 4 d), our proposed adaptation approach, FM-scVI, performs comparably to scJoint, further demonstrating that foundation models can be effectively adapted to multi-modal integration tasks through appropriate architectural modifications and training strategies, even in challenging unpaired scenarios.RetryClaude can make mistakes. Please double-check responses.
Our comprehensive assessment of trajectory conservation across diverse settings reveals that MOFA excels in paired integration scenarios while Cobolt performs best in unpaired settings. Harmony demonstrates variable performance dependent on dataset characteristics. Among foundation models, UCE shows particular strength with mouse data and developmental trajectories. While FMs exhibit promising biological signal preservation capabilities, they typically require appropriate adaptation for multi-modal integration. These findings emphasize the importance of method selection based on specific experimental contexts and research objectives.

[Section 2.6] To effectively apply the capabilities of FMs in this field and fully explore their potential across various application scenarios, we propose the FM + AdaptationLayer approach. We leverage a lightweight adaptation module (from scVI) that adapts the unified representation ability of FMs into more effective and context-specific applications in single-cell multi-omics integration. To explore the potential of FMs in multi-omics integration tasks on human data, we compare the scGPT-scVI with zero-shot prediction and two fine-tuning approaches on scGPT: scGPT-Scratch, utilizing only pretrained gene embeddings module, and scGPT-ft, employing the first eight pretrained transformer modules, which is the best configuration identified in our fine-tuning experiments (Supplementary Table 4). Fig. 6 a and the top of Fig. 6 d present UMAP visualizations of the representations, with corresponding integration accuracy scores annotated. Fig. 6 c illustrates detailed evaluation metrics of the different versions of scGPT, with scVI added for comparison. These results indicate the superior performance of scGPT with the adaptation layer, surpassing all other strategies and scVI. We further investigate whether this adaptation can improve performance on mouse datasets using UCE. An efficient fine-tuning layer is added to the pre-trained UCE model. As shown in Fig. 6 c and the bottom of Fig. 6 d, UCE-scVI increases the overall integration accuracy by over 65% compared to its zero-shot

version. This approach bridges the gap between FMs' biological knowledge and multi-omics integration requirements while maintainingcomputational efficiency.

3.In Figure 2, the authors unexpectedly applied the human data-pretrained scGPT to mouse datasets and concluded that such scFMs lack cross-species transferability. This conclusion is obvious. However, the study fails to provide critical details on how mouse data was input into scGPT, considering the gene set gap between humans and mice. Specifically, scGPT uses a human gene vocabulary, which inherently excludes genes unique to other species. How did the authors ensure the accurate and complete alignment of mouse RNA and ATAC data with scGPT's human gene vocabulary?

Response: We sincerely appreciate the reviewer's critical observation regarding the application of human data-pretrained scGPT to mouse datasets. Our initial intent was to evaluate the cross-species generalization capabilities of foundation models, which is an important consideration for the broader applicability of these models in single-cell analysis. For this cross-species analysis, we implemented ortholog mapping between mouse and human genes. However, as the reviewer correctly points out, this approach has significant limitations. The number of high-confidence ortholog gene pairs we could identify was relatively small, and FMs are inherently constrained by their fixed vocabulary size - a well-recognized limitation in the field. This resulted in an experimental setup that was potentially biased and not fully representative of the models' true capabilities.

After careful reconsideration prompted by the reviewer's feedback, we agree that this comparison does not provide a fair assessment of cross-species transferability and creates an inequitable comparison between FMs and DMs, since DMs can be directly trained on mouse data without vocabulary constraints. We recognize that cross-species transferability is a complex challenge, and appreciate the opportunity to clarify and correct this point. In the interest of scientific rigor, we have removed these results from our revised manuscript. We thank the reviewer for highlighting this important issue, which has helped us improve the accuracy and validity of our benchmarking study.

Original sentences:
[Method section 4.3, scGPT part] Since there is no specific pre-trained model available for mouse samples, we use the scGPT default model, which is trained on whole human data, for inference. All hyperparameters mentioned in the code are set to their default values.

Revised sentences:

[Method section 4.3, scGPT part] Since there is no specific pre-trained model available for mouse samples, we do not include mouse datasets in the evaluation of scGPT. All hyperparameters mentioned in the code are set to their default values.

4. The biomarker discovery benchmarking in this study relies on the top-ranked genes identified by the Scanpy package's Differentially Expressed Gene (DEG) analysis. These genes, derived from the predicted cell types based on cell embeddings generated by different methods, were used to assess the consistency and differentiation of cell-type-associated genes. However, this approach fundamentally evaluates cell type identification performance rather than true biomarker discovery. DEG-based top genes are merely biomarker candidates, as DEG analysis is known to be sensitive to statistical noise, exhibit limited biological relevance, and ignore gene-gene dependencies. Treating DEG outcomes as a biomarker ground truth overlooks the possibility that scFMs might mitigate these limitations, thereby potentially underestimating their true performance in biomarker discovery.

Response: We appreciate the reviewer's thoughtful observation regarding the limitations of differential expression (DE)-based gene ranking in evaluating biomarker discovery. To address this concern, we conducted an additional benchmarking analysis on PBMC-10x dataset using experimentally annotated biomarkers from the CellMarker 2.0 database as the ground truth, rather than relying solely on DEGs derived from Scanpy. This approach allows us to assess the biological relevance and true biomarker detection capabilities of each method beyond statistical noise.

As shown in the new figure (Figure R10), we compared the recall values of biomarkers detected from cell type clusters inferred using different method embeddings. Figure R10*a* illustrates the recall of CellMarker-annotated biomarkers among DEGs identified per cluster. In Figure R10*b*, we further contrast recall scores between predicted and prior biomarkers and their corresponding biomarker conservation scores, revealing how well each method captures conserved and functionally relevant markers. Notably, models such as **scGPT-scVI**, **scMoMaT**, and **scJoint** exhibit superior performance under both evaluation metrics, indicating their potential to recover biologically meaningful biomarkers beyond what conventional DEG-based methods can detect.

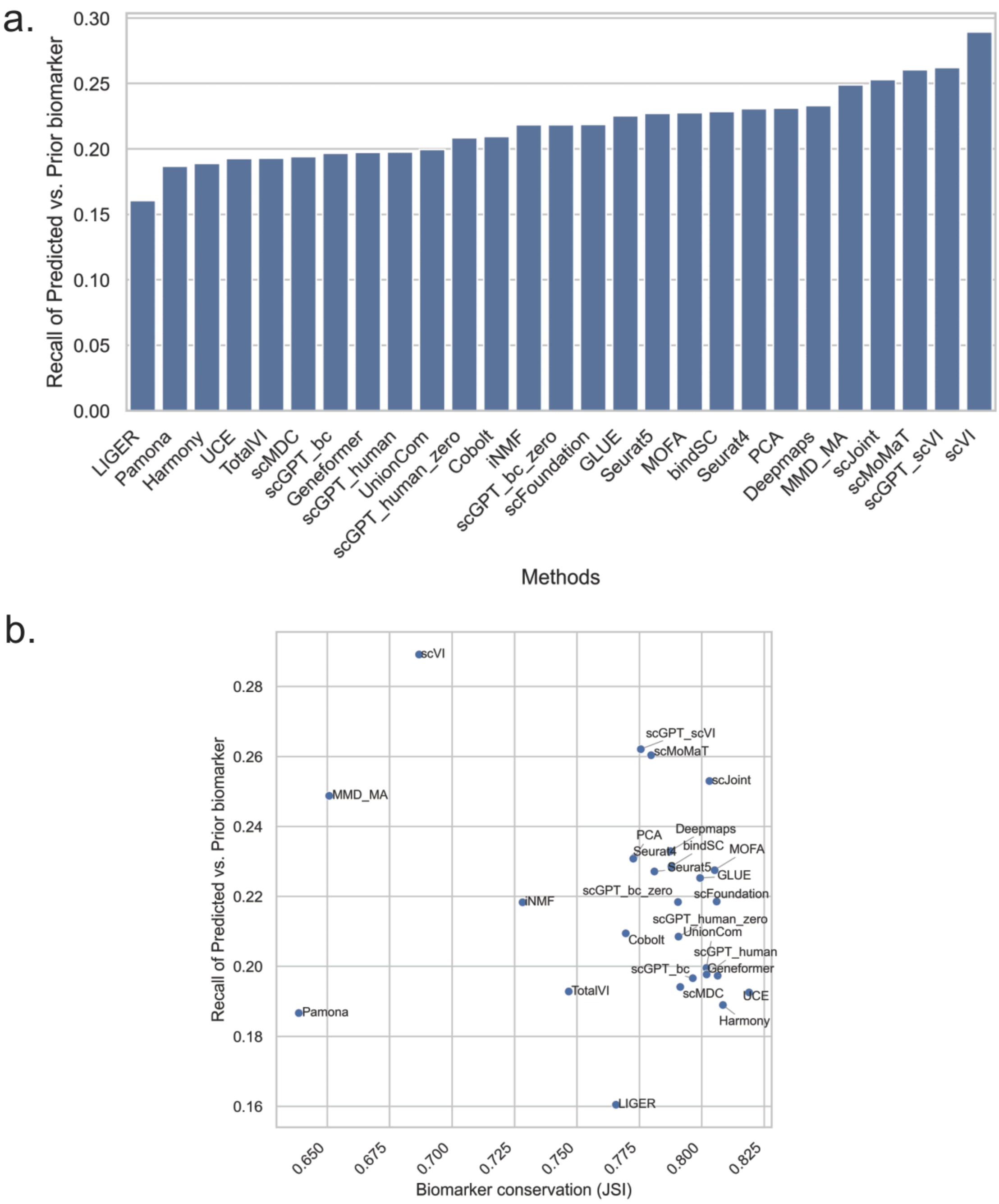

Figure R10: Supplementary Figure 4 in the manuscript

Added sentences:

[Supplementary Figure 4 caption]: Evaluation of biomarker detection on annotated biomarkers. **a.** Recall values of different methods in detecting known biomarkers, calculated by comparing DE genes from inferred cell type clusters against curated biomarkers in the CellMarker 2.0 database. **b.** Comparison of recall performance for predicted biomarkers and CellMarker biomarkers, plotted against biomarker conservation scores derived from statistically computed DEGs. Among them, scGPT-scVI, scMoMaT, and scJoint demonstrate consistent and superior detection of biologically validated markers across both analyses.

[Section 2.3] To assess biomarker detection against established knowledge, we evaluate each method's ability to recover experimentally validated cell-type markers from CellMarker 2.0 database for the PBMC-10x dataset (Supplementary Figure 4). Several methods, including scGPT-scVI, scMoMaT, and scJoint, show strong recall of known biomarkers alongside high consistency scores, indicating their effectiveness in identifying biologically meaningful cell markers beyond statistical associations. When comparing FM and DM approaches in biomarker detection, we find that FMs demonstrate promising performance in this task. Notably, the zero-shot version of scGPT outperforms many DMs in identifying NK cell biomarkers, demonstrating the strong biological knowledge encoded in pre-trained language models. Interestingly, scGPT's fine-tuned version shows relatively lower performance compared to its zero-shot counterpart, suggesting that introducing information from another omics modality may compromise some of the intrinsic biological knowledge specific to gene expression patterns when attempting to create a joint representation.

Given the concerns outlined above, I hardly support the publication of this study in its current form.

Response: We sincerely appreciate the reviewer's thorough feedback and have carefully addressed each concern through substantial revisions and additional analyses. We hope our comprehensive responses and improvements will address the reviewer's concerns and demonstrate the value of our benchmarking study.

Reviewer #4 (Remarks to the Author):

Review of "SCMBench: Benchmarking Domain-specific and Foundation Models for Single-cell Multi-omics Data Integration"

This paper presents a comprehensive benchmark study, SCMBench, for evaluating the performance of various integration methods on single-cell multi-omics data. The study compares 22 popular methods, including both statistical-based and deep learning-based approaches, as well as four foundation models (FMs). The benchmark applied the framework established in a previous study by Malte et al. [1], thereby limiting the novelty of the benchmark. The major weakness of the benchmark is that it mostly focuses on the scRNA-seq-related analysis tasks while barely comparing performance on the task to another modality (scATAC-seq). The most significant contribution of the paper is to provide an assessment of FMs in the context of single-cell multi-omics data integration. It demonstrates the current state of FMs in the single-cell bioinformatics community. The abstract indicates that there is still a performance gap between FMs and current mainstream pipelines known as Domain-specific Models (DMs). Therefore, it is difficult to draw a conclusion for the recommendation of FMs as they require intensive downstream fine-tuning. Besides, some of the benchmarks are not well aligned with the use case of the bioinformatics analysis. There are several other issues that the paper needs to address, and the detailed comments are outlined below:

Response: Thank you for your valuable feedback on our SCMBench manuscript. We appreciate your recognition of our work in providing an assessment of foundation models for single-cell multi-omics integration. We have addressed your concerns regarding the focus on scRNA-seq-related tasks and other issues through several substantial improvements:

1. **Expanded multi-omics evaluation:** We have included a triple-omics integration benchmark using single-cell RNA-seq, ATAC-seq, and DNA methylation data from mouse cortex tissue, providing insights into foundation model performance in more complex multi-modal integration scenarios.
2. **Enhanced scATAC-seq evaluation:** We have introduced comprehensive biological conservation evaluation at the scATAC-seq level with two modality-specific features:
    - Differentially Accessible Regions (DARs) – identifying condition-specific chromatin accessibility patterns using t-test with Benjamini-Hochberg correction
    - Motif Enrichment Analysis – determining transcription factor binding motifs enriched within Top 100 DARs using GimmeMotifs with MEME, BioProspector, and Homer

3. **Extended trajectory conservation analysis:** We have added evaluations on two additional biologically meaningful systems:
    - Human B cell lineage in PBMCs – focusing on the developmental continuum from HSPCs to plasma cells
    - Neuronal differentiation in mouse cortex – capturing progression from intermediate progenitor populations to upper-layer excitatory neurons
4. **Comprehensive computational benchmarking:** We have provided detailed specifications including:
    - Hardware configurations used in the benchmark
    - Package versions for each method
    - CPU/GPU compatibility information
    - Comprehensive runtime metrics for both CPU and GPU executions
5. **Clarified foundation model evaluation:** We have been more precise in describing foundation models' capabilities and reorganized the discussion to include a dedicated section on FMs in multi-omics integration
6. **Enhanced preprocessing documentation:** We have added detailed descriptions of preprocessing pipelines and all scripts are available at our Github
7. **Added Harmony evaluation:** Following your suggestion, we have included Harmony in our benchmarking, providing a more comprehensive comparison of integration methods.

We greatly appreciate your suggestions that guided us to take a more practical perspective and develop a more comprehensive benchmarking framework. These improvements significantly enhance the manuscript's utility for researchers selecting integration methods for real-world applications.

Major comments:
1. The primary weakness of the paper lies in its focus on the "Single-cell Multi-omics Data Integration" setup, where most metrics tested are derived at the transcriptomic level (e.g. trajectory and biomarker score), or cell label (e.g. NMI, ARI) with almost no discussion of performances on other modalities. Furthermore, the paper overlooks additional single-cell data modalities like DNA methylation and chromosome conformation capture (3C) data. Focusing solely on applying metrics to scRNA-seq data is acceptable, considering that previous research primarily evaluated integration methods for transcriptomic data. [1]. In establishing a benchmark setup for multi-omics integration, the author should design modality-specific assessments. For example, examining biological conservation metrics like the conservation of differentially accessible regions, motif enrichments, and peak calling results at the scATAC-seq level.

Response: We appreciate the reviewer's thoughtful feedback regarding our study's focus on primarily transcriptomic-level metrics in evaluating multi-omics integration methods. In designing our benchmark, we intentionally selected downstream evaluation tasks based on their prevalence in mainstream integration methods and their biological relevance. While transcriptomic-level assessments are widely used in the field, we fully agree that a comprehensive evaluation should give appropriate attention to non-transcriptomic modalities as well. Additionally, we did not include triple-omics integration in the main benchmark due to the limited number of existing methods currently capable of handling this task, with the mainstream of methodological development and application still predominantly focused on RNA–ATAC integration. In response, we have significantly expanded our benchmark framework to include modality-specific assessments across multiple data types in the revised manuscript to address this important concern.

Specifically, we have included a **triple-omics integration benchmark** using single-cell RNA-seq, ATAC-seq, and DNA methylation data from mouse cortex tissue (Figure R11). This newly added experiment evaluates the integration accuracy of seven methods that are capable of handling triple-omics datasets, with performance assessed with integrated clustering accuracy metrics and multi-modalities matching.

Added Sentences:

[Section 2.2] To further assess the versatility and robustness of existing multi-omics integration methods, we extend our benchmarking to the triple-omics dataset comprising scRNA-seq, scATAC-seq, and DNA methylation data derived from mouse cortex. Seven methods capable of handling these three modalities are evaluated. Integration performance is assessed using established batch correction metrics, where omic modality is treated as a surrogate for batch labels (Supplementary Figure 3a). In parallel, we evaluate cell type resolution by measuring the accuracy of clustering after integration (Supplementary Figure 3b). Among the tested methods, GLUE consistently achieves the highest scores in both modality alignment and cell type clustering, demonstrating its strong capacity to integrate heterogeneous omic layers and preserve biologically meaningful structures.

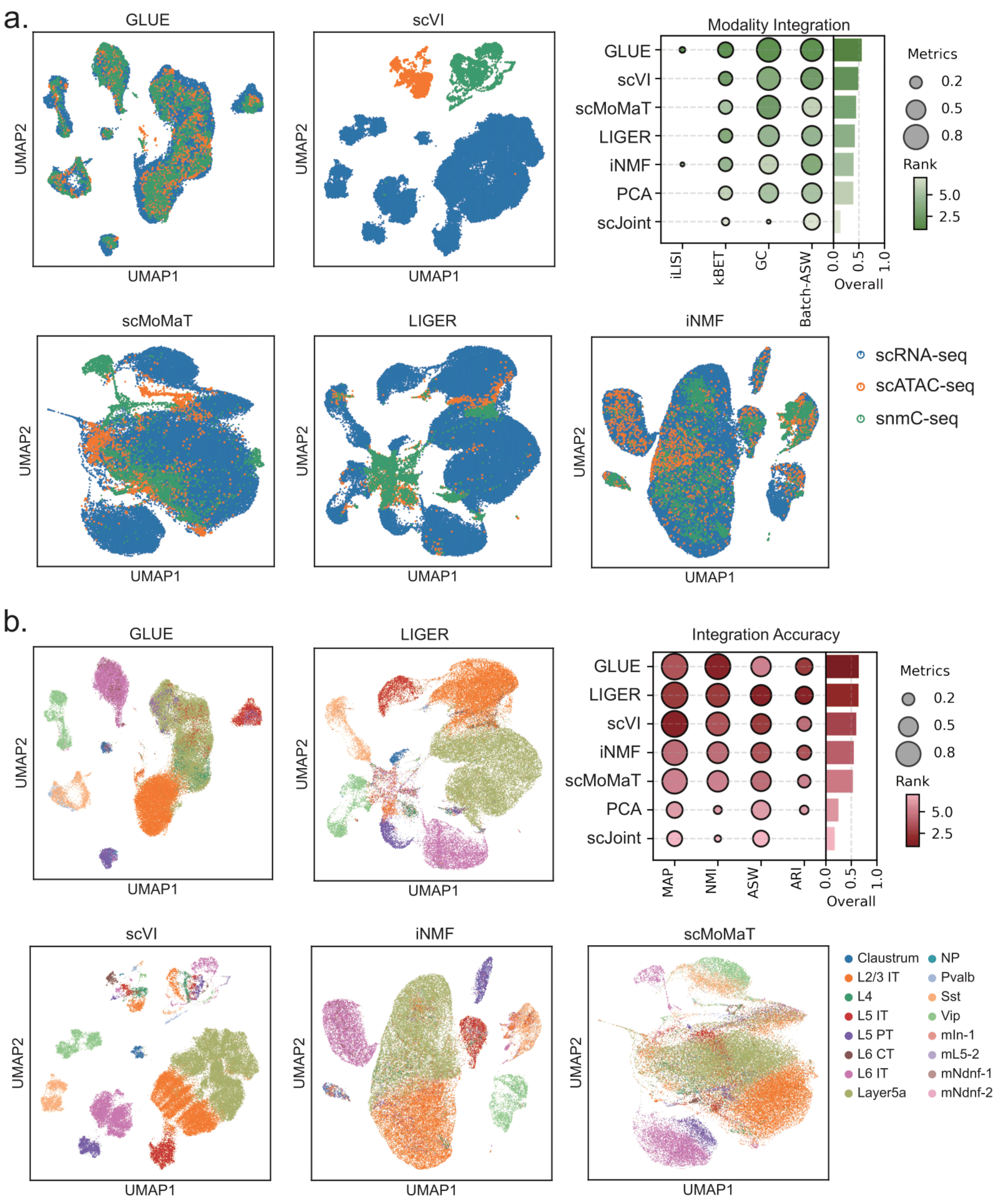

Figure R11: Supplementary Figure 3 in the manuscript

Furthermore, we have introduced a biological conservation evaluation at the scATAC-seq level , in addition to transcriptomic-level metrics. This includes two modality-specific features:

1. **Differentially Accessible Regions (DARs)** – identifying condition-specific chromatin accessibility patterns. Since the input datasets are at single cell level, we utilize similar manner with DE genes to identify DARs, with t-test and 'benjamini-hochberg' P-value corrected method.

2. **Motif Enrichment Analysis** – determining transcription factor binding motifs enriched within Top 100 DARs. In practice, we use GimmeMotifs (with MEME, BioProspector and Homer as default prediction tools) to perform motif enrichment analysis. Only known motifs curated in GimmeMotifs database are considered.

These two metrics, along with trajectory conservation and biomarker detection, provide a more holistic evaluation of biological signal preservation across multiple modalities. The results, spanning three datasets that include both human and mouse tissues (PBMC-10x, Ma-2020, Chen-2019), are summarized in Figure R12. Figure R13 presents the biological conservation scores of each metrics for both paired and unpaired datasets.

We believe these additions significantly strengthen the manuscript by offering a modality-aware evaluation framework and addressing the limitations highlighted by the reviewer.

Original sentences:
[Caption of Figure 3] **b.** The box plot shows the comparison of biomarker detection consistency for paired datasets. These visualizations capture the variability and reliability of each method in identifying cell-type-specific markers, with narrower boxes and shorter whiskers indicating greater robustness. **c.** Critical difference diagram for the cell-type-specific biomarker detection capability of each method across all paired datasets. Methods are ranked along the x-axis according to their biomarker detection consistency, with linked methods showing no significant statistical difference in performance.

Revised sentences:
[Caption of Figure 3] **b.** Box plot comparing biological conservation scores across methods. The conservation score is computed as the average detection consistency of cell-type-specific biomarkers, differentially accessible regions (DARs), and enriched transcription factor motifs. Narrower boxes and shorter whiskers indicate greater robustness and consistency across datasets. c-e. Method rankings based on average performance across datasets for the detection of (**c**) cell-type-specific biomarkers, (**d**) DARs, and (**e**) enriched motifs. These rankings highlight the relative effectiveness of each method in preserving biologically meaningful features.

Added sentences:
[Caption of Supplementary Figure 5]: Biological conservation evaluation at the ATAC level.
a-c. Evaluation of biological conservation across four aspects: trajectory preservation, biomarker identification, differential accessible regions (DARs), and motif enrichment. The latter two are specific to the scATAC-seq modality. Results are presented for three datasets—PBMC-10x (**a**), Ma-2020 (**b**), and Chen-2019 (**c**)—which include both human and mouse tissues. Methods are ranked for each dataset based on their overall biological conservation scores.

[Caption of Supplementary Figure 3]: Benchmarking integration of scRNA-seq, scATAC-seq and DNA methylation in mouse cortex.
a. Integration performance of seven methods capable of handling triple-omics data, assessed using batch correction metrics with modality treated as batch labels.
b. Cell type clustering accuracy following integration. GLUE achieves the highest performance in both modality alignment and cell type resolution.

[Section 4.2.2]

- **Biomarkers Detection Consistency** (BM): To evaluate the preservation of cell-type-specific biological signals, we computed a biomarker detection score for each method as follows. First, cell types are annotated based on the embeddings produced by each method using a k-nearest neighbors (kNN) classifier. For each annotated cell type, we identified the top 100 differentially expressed genes (DEGs) as candidate markers. The DEGs are identified from scRNA-seq data using 'rank_genes_groups' function with t-test of Scanpy package. We then calculated the Jaccard similarity index (JSI) of marker gene sets between all pairs of methods, separately for each cell type. The biomarker detection score of a method was defined as the average JSI across all cell types and method pairs involving that method. This score reflects the consistency of cell-type-specific marker detection across different integration approaches.

- **Biomarker detection with prior knowledge**: To evaluate biomarker prediction against known references for the PBMC-10x dataset, we collected curated cell-type-specific markers from CellMarker 2.0 as ground truth [62]. For each method and cell type, we compute the recall by measuring the proportion of predicted candidate biomarkers that match the known markers. This metric reflects agreement with established knowledge, but does not account for potentially novel or unannotated biomarkers.

- **Differential accessible regions detection** (DARs): This score was computed similarly to the biomarker detection score, except that top 100 differentially accessible regions (DARs) were identified for each cell type from scATAC-seq data instead of marker genes. Jaccard similarity indices of DAR sets were calculated across all method pairs and averaged to quantify consistency.

- **Enriched motifs** (Motifs): Building on the DARs identified in the previous step, we performed motif enrichment analysis using GimmeMotifs (with MEME, BioProspector, and Homer as default tools) [52]. Only known motifs curated in the GimmeMotifs database were considered. Surprisingly, the identified DARs are all upregulated, and the enriched motifs may be involved in gene regulation processes. We computed the consistency score by comparing enriched motifs across methods using Jaccard similarity indices, as done for biomarkers and DARs.

[Section 2.3] Beyond evaluating transcriptomic-level markers, we extend our assessment to the epigenomic dimension by analyzing chromatin accessibility patterns in scATAC-seq data. This extension includes two modality-specific features: Differentially Accessible Regions (DARs) identified using similar statistical approaches as DE genes, and enriched transcription factor binding motifs within the top 100 DARs determined through GimmeMotifs analysis. These evaluations are conducted across all datasets, including paired, unpaired human, and mouse samples, with results displayed in Figure 3 c and d, while detailed dataset-specific results are provided in Supplementary Figure 5. In the epigenomic assessment, Seurat4 achieves the highest scores for both DARs and enriched motifs detection, demonstrating its exceptional capability in capturing chromatin accessibility patterns and regulatory elements. scJoint, LINGER, and Harmony follow closely behind, indicating their strong performance in preserving epigenomic features during integration.

Figure 3 e presents the overall biomarker detection performance ranking across all three metrics. Consistently, bindSC, Seurat4, PCA, and scJoint demonstrate superior performance, establishing themselves as effective methods for multi-omics biomarker discovery. Despite moderate transcriptomic performance, UCE achieves results comparable to several DMs, while at the scATAC-seq level, FM-scVI shows comparable effectiveness to established methods like Harmony and GLUE. These findings suggest that while specialized integration methods currently lead in biomarker detection, foundation models demonstrate promising capabilities that could be enhanced through targeted improvements to preserve both transcriptomic and epigenomic signals.

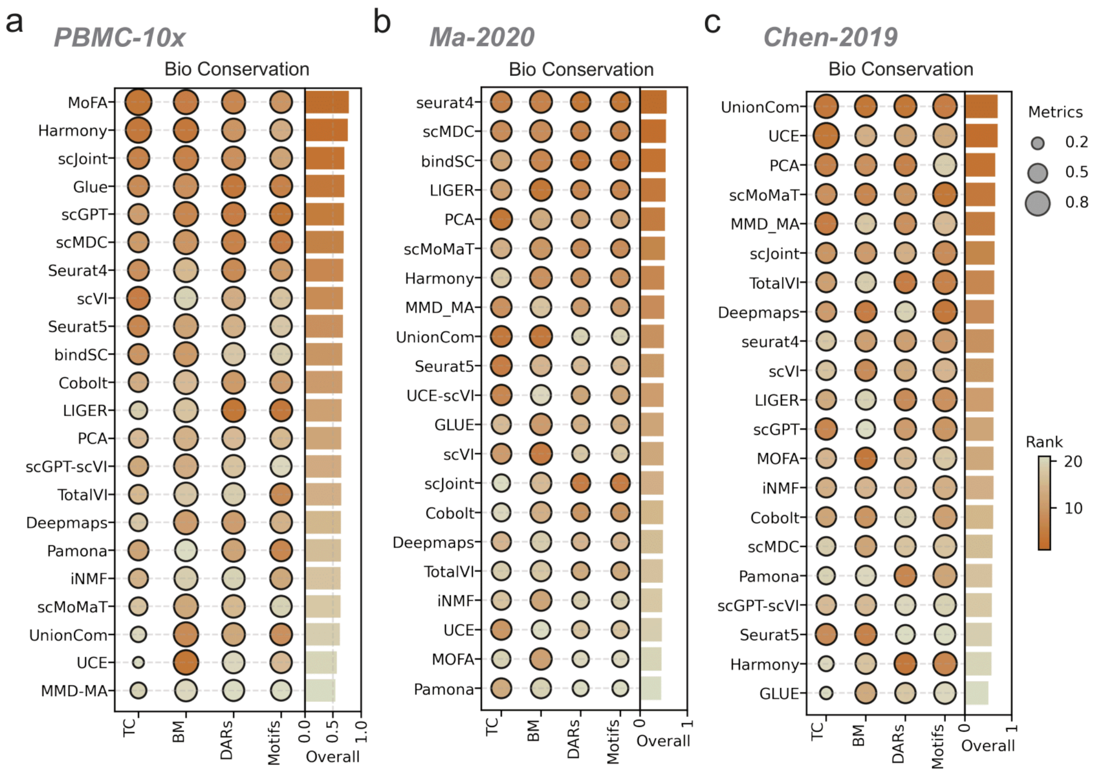

Figure R12: Supplementary Figure 5 in the manuscript

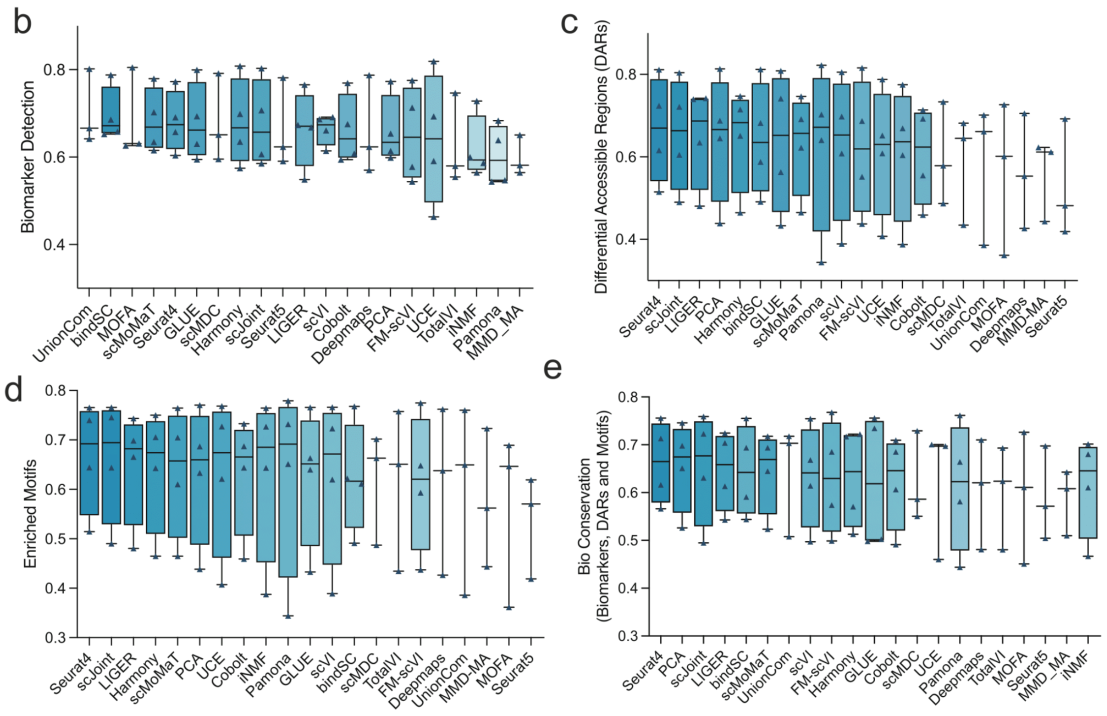

Figure R13: Figure 3 b-e in the manuscript

2. As mentioned in the comment 1. Testing the performance of integrating data for more modalities such as single-cell methylation is preferred.

Response: Thank you for your suggestions. The benchmark experiments of triple-omics integration including single cell DNA methylation are performed (Figure R11). And you can refer to the response of comment 1 for the detailed explanation. This is indeed a valid comment that prompted us to further examine our current methodology. We found that only seven methods are capable of handling triple-omics integration, with GLUE achieving the best performance among them. However, compared to the dual-omics results, the overall performance shows a decline across all methods, indicating that more sophisticated methods are needed for effective triple-omics integration. The detailed statistical information has been included in Supplementary Table 4, and we have discussed this limitation and future research direction in the Discussion section.

Revised sentences:
[Discussion] **Limitations and Future Directions Despite** our comprehensive benchmarking efforts, several limitations should be acknowledged. First, our evaluation focuses primarily on scRNA-seq and scATAC-seq integration, while other important multi-omics combinations (such as protein, spatial, or metabolomics data) remain to be explored. We also conducted triple-omics integration experiments including single-cell DNA methylation data, which showed that only seven methods are capable of handling triple-omics integration, yet all methods exhibited decreased performance compared to dual-omics results (Supplementary Table 2). This highlights the technical challenges in integrating three or more modalities simultaneously. As multi-omics technologies continue to advance, more sophisticated computational approaches are needed to effectively handle complex multi-modal data integration.

[Caption of Supplementary Table 2] Classification and other information of the 23 integration methods. Methods details, including classification of methods, applicability of unpaired scenarios and Triple-omics integration, Programming Language, and so on.

Supplementary Table 2: **Classification and other information of the 23 integration methods.** Methods details, including classification of methods, applicability of unpaired scenarios and Triple-omics integration, Programming Language, and so on.

Methods	Statistical-based	DL-based	DM or FM	Unpaired	Triple-Omics	Programming Language	Year	Reference
MOFA	✓		DM			Python	2018	[8]
MMD-MA	✓		DM			Python	2019	[9]
PCA	✓		DM	✓	✓	Python	2019	[10]
Harmony		✓	DM	✓		Python	2019	[11]
UnionCom	✓		DM			Python	2020	[12]
LIGER	✓		DM	✓	✓	R	2020	[13]
TotalVI		✓	DM			Python	2021	[14]
Seurat4	✓		DM	✓		R	2021	[15]
iNMF	✓		DM	✓	✓	R	2021	[16]
Cobolt		✓	DM	✓		Python	2021	[17]
scMDC		✓	DM			Python	2022	[18]
Pamona	✓		DM	✓		Python	2022	[19]
bindSC	✓		DM	✓		R	2022	[20]
scJoint		✓	DM	✓	✓	Python	2022	[21]
GLUE		✓	DM	✓	✓	Python	2022	[22]
Deepmaps		✓	DM			R	2023	[23]
Seurat5	✓		DM	✓		R	2023	[24]
scMoMaT	✓		DM	✓	✓	Python	2023	[25]
scVI		✓	DM	✓	✓	Python	2023	[26]
Geneformer		✓	FM	✓		Python	2023	[5]
UCE		✓	FM	✓		Python	2023	[27]
scGPT		✓	FM	✓		Python	2024	[28]
scFoundation		✓	FM	✓		Python	2024	[29]

3. The benchmark of the Trajectory Conservation (TC) on a PBMC dataset is questionable. The author should test the TC on a dataset that focuses on tracing cell lineages or for longitudinal scRNA-seq study. For instance, the benchmark paper which the author closely follows tested the TC on data studying erythrocytes and their progenitors [1]. Studying the pseudo-time on the PBMC data alone does not reveal significant biological insights, because the root cell type is set manually to be HSPCs. Besides, the author should add annotation on each of the cell clusters in Figure 4 to indicate cell types upon the trajectory.

Response: We thank the reviewer for the insightful suggestion regarding the benchmark of trajectory conservation (TC). We acknowledge that the peripheral blood mononuclear cell (PBMC) dataset, while commonly used for benchmarking integration tasks, may not be ideal for evaluating trajectory inference due to its heterogeneous nature and the manual designation of root cell types such as HSPCs. To address this concern and improve the biological interpretability of our evaluation, we have expanded our benchmark to include datasets with well-characterized developmental trajectories.

Specifically, we performed trajectory conservation analysis on two additional biologically meaningful systems:

1. **Human B cell lineage in PBMCs** – a subset focusing on the developmental continuum from HSPCs to Naive B cells, Intermediate B cells, Memory B cells, and Plasma cells (Figure R14; revised as Figure 4 in the main manuscript).

2. **Neuronal differentiation in the mouse cortex** – capturing the progression from intermediate progenitor populations (IP-Hmgn2, IP-Gadd45g, IP-Eomes) to upper-layer excitatory neurons (Ex-L2/3-Cntn2 and Ex-L2/3-Cux1) (Figure R15; added as Supplementary Figure 8).

In each case, we provide cell type annotations and pseudotime trajectories derived from established biological knowledge for reference. This allows for a more rigorous and biologically grounded assessment of trajectory conservation across integration methods.

As shown in Figure R14, five methods (Seurat v5, PCA, UnionCom, MOFA, and UCE) achieved TC scores greater than 0.8, successfully recovering the known B cell developmental trajectory. The pseudotime ordering—from Naive B to Memory B cells—is clearly observed. In the mouse cortex dataset (Figure R15), we presented the top six methods and visualized the RNA modality, ATAC modality, and the integrated data. Notably, the developmental trajectory is more apparent in the RNA modality, reflecting the stronger transcriptional signal of differentiation. Most methods yielded lower TC scores (< 0.7) in this setting, with the exception of UCE, which maintained superior performance across both datasets.

In response to the reviewer's final suggestion, we have added explicit cell type annotations to all clusters along the trajectories shown in Figure 4 and Supplementary Figure 8 to enhance biological clarity.

Original sentences:

[Figure 4 caption]: **a-b.** PAGA plot of the ground truth and UMAP visualizations of the pseudotime inference result from the integrated cell embeddings of 18 DMs and four FMs on PBMC-10x. Both the fine-tuned result and the zero-shot learning result from scGPT_bc are displayed. All subplots are arranged in descending order based on the TC score.

Revised Sentences:

[Figure 4 caption]: **a-b.** PAGA representation of the ground-truth lineage, and UMAP visualizations of pseudotime inference and cell type annotations, derived from the integrated embeddings of the top 8 methods on PBMC-10x (out of 18 DMs and 4 FMs). The analysis focuses on B cell development. All subplots are arranged in descending order based on the TC score.

[Section 2.4] We also evaluate trajectory conservation on the Ma-2020 dataset focused on neuronal differentiation in the mouse cortex, specifically capturing the progression from intermediate progenitor populations (IP-Hmgn2, IP-Gadd45g, IP-Eomes) to upper-layer excitatory neurons (Ex-L2/3-Cntn2 and Ex-L2/3-Cux1) (Supplementary Figure 8). Notably, the developmental trajectory is more apparent in the RNA modality, reflecting the stronger transcriptional signal of differentiation. Most methods yielded lower TC scores less than 0.7 in this complex developmental system, with the exception of UCE, which maintained superior performance across both datasets. We further evaluate trajectory conservation in the unpaired data scenario using all cells across multiple datasets. As shown in Fig. 4 d and Supplementary Figure 14f, Cobolt and Harmony demonstrate superior TC performance with statistically significant results compared to other methods. PCA and scJoint also show notable effectiveness in preserving trajectory information in the unpaired setting, highlighting that simpler methods can sometimes maintain important biological relationships even without complete data alignment.

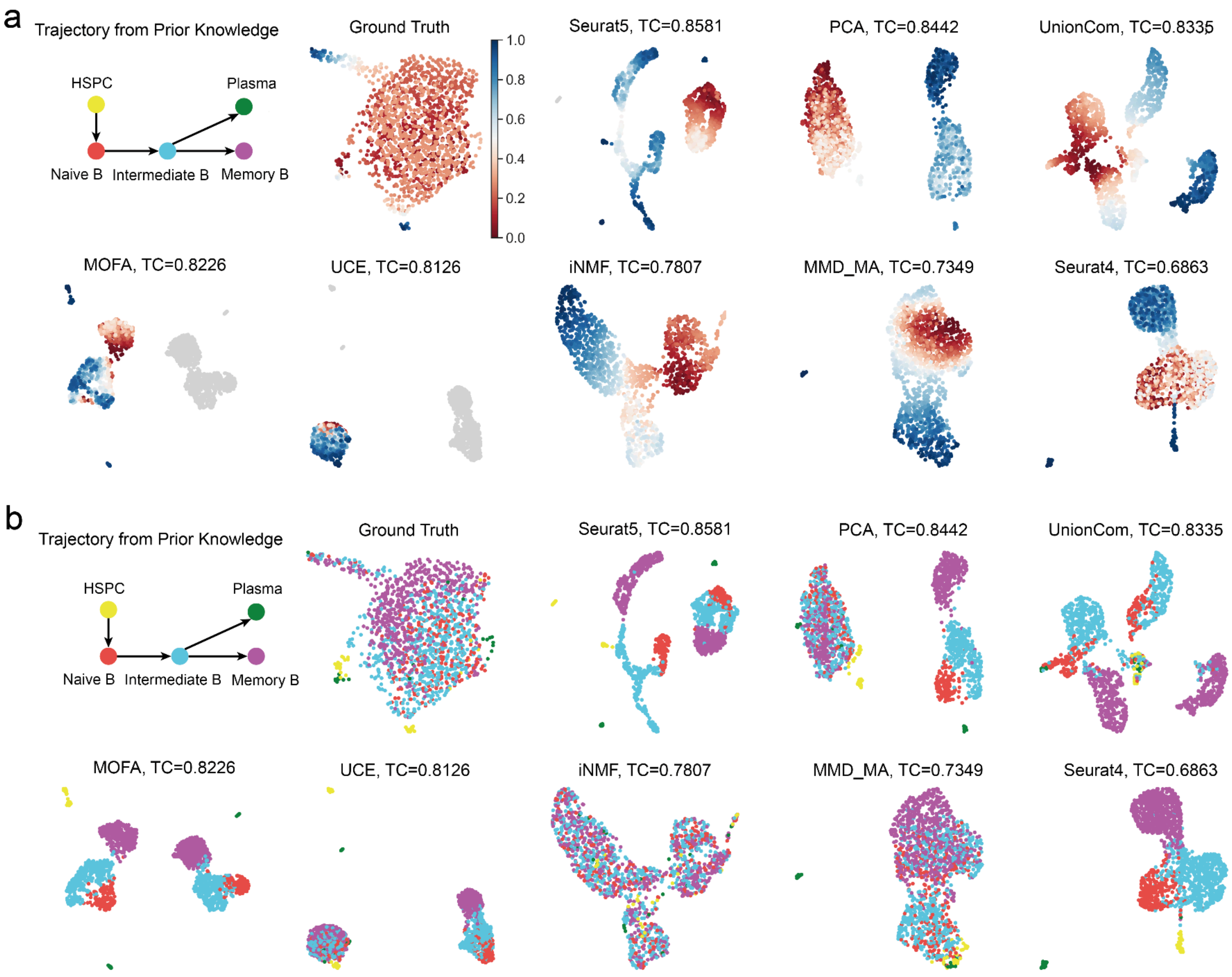

Figure R14: Figure 4 a,b in the manuscript

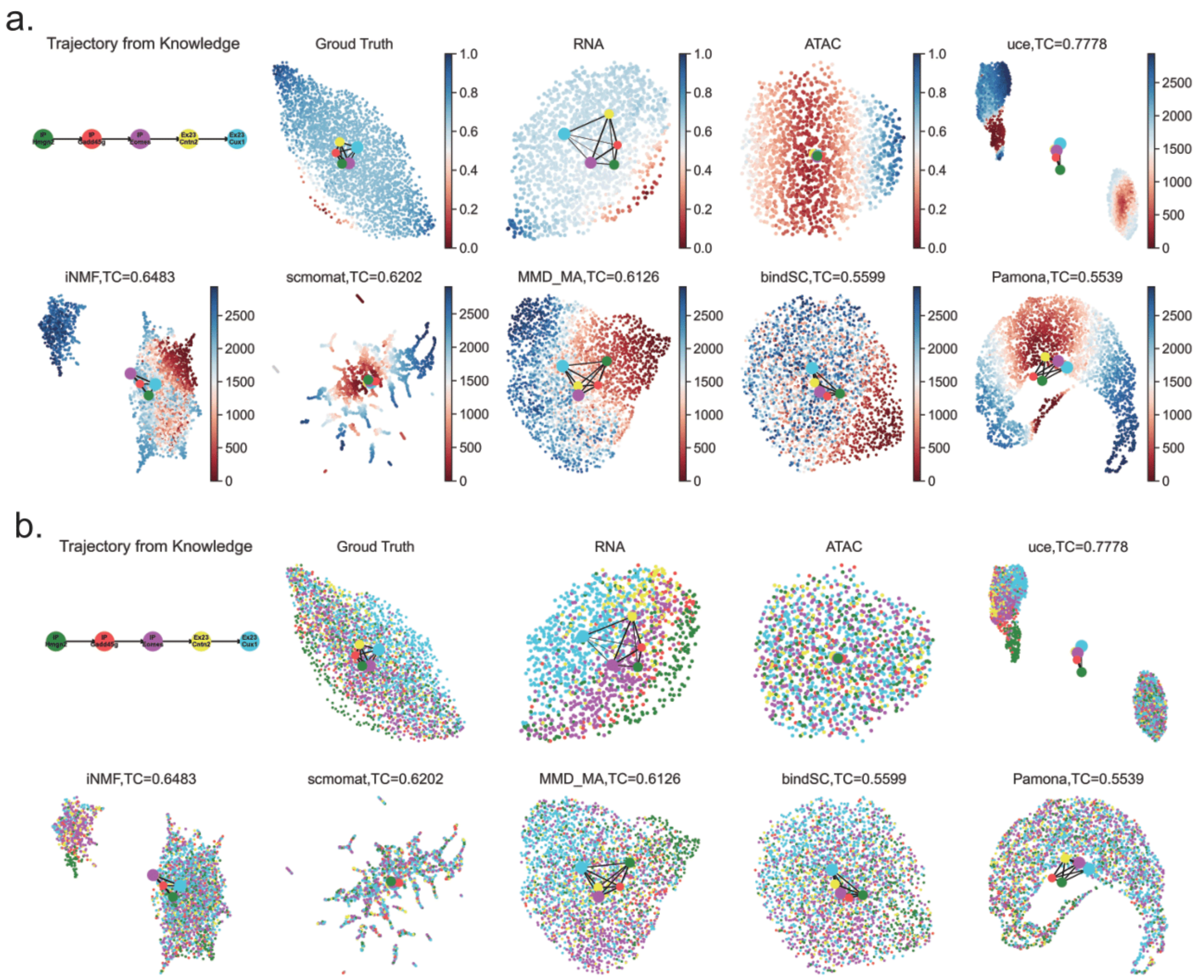

Figure R15: Supplementary Figure 8 in the manuscript

4. The computation efficiency test is too brief. The author only tested the running time. Is it wall clock time or CPU time? What about their RAM/GPU VRAM consumption? Does each of the methods require GPU? What is the difference in the running time between the GPU version and the CPU version? What is your testing environment and hardware spec?

Response: We appreciate the reviewer's insightful suggestion regarding the computational efficiency evaluation. We acknowledge that the description of computation efficiency in our previous manuscript is not enough. To address these concerns, in the revised manuscript, we have added a comprehensive assessment of computational performance for all methods. Specifically, we report the **CPU execution**

time, **peak RAM usage**, and **peak GPU memory consumption** for each method in Figure 2. Additionally, we compare the running time of CPU and GPU versions of six methods applicable to both (Figure R16). All detailed information regarding the computational efficiency evaluation is provided in the file *Extended_Data_5_tech.xlsx*. This file includes:

- Specifications of the installation environments
- Hardware configurations used in the benchmark
- Package versions for each evaluated method
- Information on CPU/GPU compatibility, including whether a method supports GPU acceleration and its default computational backend
- A summary of CPU time, peak RAM usage, and peak GPU memory consumption
- Comprehensive runtime metrics for both CPU and GPU executions of each tool, including wall-clock time, CPU time, memory usage, and GPU memory utilization.

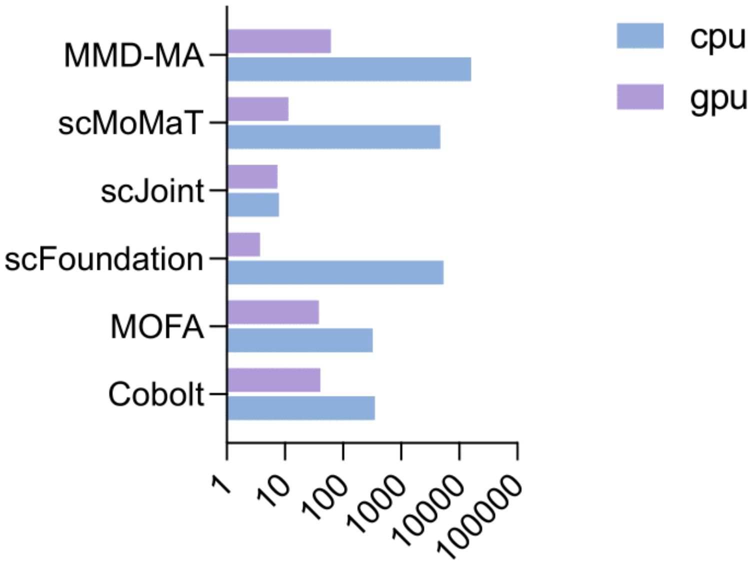

Figure R16: Supplementary Figure 15 in the manuscript

Revised Sentences:

[Section 2.2] Computational efficiency represents a critical factor in method selection, particularly for large-scale multi-omics analyses that can strain computational resources. To provide comprehensive guidance for practical applications, we conduct detailed measurements of wall clock time, CPU time, RAM and GPU VRAM

consumption across all methods in a standardized testing environment (detailed hardware specifications provided in Extended Data). As shown in Figure 2 d, methods vary dramatically in their computational demands. We sort the results by CPU processing time, revealing that scFoundation demonstrates remarkably fast inference times despite being a FM. scJoint also performs efficiently with moderate processing times and notably low peak RAM and GPU memory usage, making it suitable for resource-constrained environments. In contrast, fine-tuning scGPT requires substantially longer processing times and higher memory consumption, presenting potential barriers for researchers with limited computational resources. Our FM-scVI adaptation approach maintains computational efficiency similar to standard scVI, offering a significant advantage over direct FM fine-tuning. This strategy leverages pre-trained biological knowledge while avoiding the computational burden of large language models, demonstrating a cost-effective approach to harnessing FMs' capabilities.

5. The recommendation of the FM applications seems to be struggling in the paper. The author states "Generally, we find FMs fall short of state-of-the-art DMs in this field." Besides, there is a need to apply adaptation learning or further fine-tuning for FMs in single-cell tasks. However, the discussion notes that "FMs are crucial tools in single-cell research due to their versatility and strong generalization abilities". In my opinion, the requirement of adaptation learning (rather than simple prompt engineering) has already contradicted the conclusion of the generalizability of FMs. Downstream learning and fine-tuning also require additional computational power while large models introduce the risk of underfitting on small datasets. Combining the unsatisfactory performance and all the concerns, is the author still confident to conclude that the paper can "unlock the full potential of FMs and promote their effective application in the single-cell multi-omics integration"? Please comment.

Response: We thank the reviewer for this thoughtful comment that allows us to clarify our position on foundation models (FMs) in single-cell multi-omics integration. We acknowledge the apparent contradiction pointed out. Based on your comments and our comprehensive evaluation during the revision, we have tuned down our claims regarding the superiority of FMs, especially for multi-omics integration tasks. Our results clearly demonstrate that FMs currently fall short of state-of-the-art DMs in this field, and the requirement for adaptation learning does indeed challenge the notion of their inherent generalizability. We recognize that the current performance gap, combined with the additional computational overhead and the risk of underfitting on smaller datasets, presents significant practical limitations. Unlike other fields such as protein or RNA sequence analysis where FMs have achieved remarkable success, single-cell

multi-omics integration appears to present unique challenges that current FM architectures have not yet adequately addressed.

We have revised this to emphasize their 'versatility and efficiency' instead, which better reflects their actual capabilities as demonstrated in our experiments. The 'versatility' refers to their ability to be adapted across different single-cell analysis tasks and data types with the same pre-trained architecture, while 'efficiency' highlights their advantage in requiring less task-specific training data and computational resources compared to training domain-specific models from scratch. Additionally, our experiments revealed that for large datasets, some domain-specific methods become computationally prohibitive with excessive training time and resource requirements that prevent completion, whereas FMs can be efficiently fine-tuned even on large-scale data. However, we acknowledge that this efficiency comes at the cost of requiring fine-tuning to achieve competitive performance, rather than demonstrating true generalization capabilities. We also reorganized the discussion part to include a FMs in Multi-omics Integration paragraph to discuss in more details.

Regarding computational considerations, we acknowledge that adaptation learning and fine-tuning require additional computational resources. However, our FM-scVI adaptation approach offers a relatively efficient solution. Since we only need to perform inference with the pre-trained FM once to obtain embeddings before feeding them into the adaptation layer, the computational burden is significantly reduced compared to full fine-tuning of large models. In fact, this adaptation approach can be more computationally efficient than many domain-specific models, particularly those employing graph neural networks or complex attention mechanisms. Our benchmarking includes the computational efficiency assessment, which demonstrates that our FM adaptation approach maintains reasonable computational requirements compared to several DMs.

The results of incorporating our FM-scVI approach into comprehensive benchmarking across integration accuracy, biomarker detection, and trajectory inference provide encouraging evidence for the potential of adaptation strategies. Despite being a relatively straightforward approach, FM-scVI achieves performance comparable to—and in some cases exceeding—specialized methods that were explicitly designed for multi-omics integration. This suggests that the rich biological knowledge encoded in FMs can indeed be effectively leveraged for integration tasks with appropriate architectural modifications.

Overall, for users equipped with expertise in fine-tuning or adapting FMs, the application of FMs may offer potential value in certain scenarios, particularly where model flexibility and scalability are prioritized. We believe our work provides useful insights into the

current capabilities and limitations of FMs in single-cell multi-omics integration. While our results indicate that current FM performance has not yet consistently matched specialized methods across all evaluated tasks, they reveal both challenges and opportunities in this emerging area. We hope that future adaptation strategies—potentially incorporating modality-specific designs, improved alignment methods, or more sophisticated architectural approaches—may build upon our findings to enhance FM performance in this domain. Our benchmarking framework offers a systematic foundation for evaluating such developments as the field continues to evolve, helping researchers and practitioners make informed decisions about method selection based on their specific requirements and expertise levels.

Revised Sentences:

[Discussion] **FMs in Multi-omics Integration** While FMs have demonstrated transformative potential in other fields, their capabilities in single-cell biology remain underexplored. Our benchmark results, alongside published literature, suggest that current single-cell FMs have not yet been adequately harnessed for this domain. While generally underperforming compared to specialized DMs in overall integration accuracy, FMs demonstrate unique strengths in versatility and efficiency. Their versatility enables adaptation across diverse single-cell tasks with the same architecture, while their efficiency advantages include both reduced training requirements and computational feasibility on large-scale datasets where many specialized DMs become prohibitively expensive or fail to complete. However, their struggle with multi-modal integration in the zero-shot setting underscores the necessity for appropriate adaptation strategies when handling different data modalities. Most FMs are predominantly trained on human data, leading to cross-species bias when generalizing to other organisms. Even UCE, which attempts to address this by training on diverse tissues and species, suffers from data imbalance issues in its training atlas. The exceptional performance of UCE on mouse developmental data suggests that species-specific pre-training may confer advantages for certain biological contexts. To address these limitations while leveraging FMs' rich biological knowledge, we propose the FM + AdaptationLayer approach. Specifically, we develop FM-scVI, a hybrid architecture that combines FM embeddings with scVI's variational autoencoder framework. We implement this adaptation as a standalone method and incorporate it into our comprehensive benchmarking pipeline, evaluating it alongside traditional deep learning methods across all metrics and datasets. Our results demonstrate that this strategy effectively bridges the gap between FMs' biological knowledge and the specific requirements of multi-omics integration tasks, showing particular improvements in biomarker detection and trajectory conservation while maintaining computational efficiency. However, further refinement and validation are necessary to fully realize its potential.

Minor comments:

1. The preprocessing methods including the application of pipeline, quality control metrics, and hyper-parameters applied for scRNA-seq and scATAC-seq are unclear. The author should justify them in the method section or the supplementary data.

Response: We appreciate the reviewer's comment regarding the clarity of our preprocessing methods. We agree that transparent reporting of preprocessing steps is crucial for reproducibility and interpretation of benchmarking results.

In our manuscript, we have included preprocessing details in Section 4.1 (Real Data Preprocessing), where we describe our approach to data subsampling and feature selection. Additionally, we have provided complete preprocessing code in our GitHub repository to ensure full reproducibility:
https://github.com/ml4bio/SCMBench/tree/main/data/preprocessing

However, we acknowledge that our description of scATAC-seq processing, particularly regarding the generation of gene activity matrices, may not have been sufficiently clear. Therefore, we have expanded this section to include more detailed information on:

1. The sampling strategy applied to both scRNA-seq and scATAC-seq data to avoid OOM on some settings, including cell and feature filtering thresholds.
2. The computational pipeline used to generate gene activity scores from scATAC-seq data.

Specifically, we have clarified that we calculate gene activity scores from chromatin accessibility data using the regulatory potential model implemented in the MAESTRO package. This biologically informed approach weights peak signals based on their distance from transcription start sites, reflecting the established relationship between chromatin accessibility and gene regulation. Our supplementary analyses (now included in Supplementary Fig. 1) validate this approach by demonstrating that the resulting activity matrix successfully preserves cell-type-specific marker gene signals, maintains biological clustering structure, and retains chromatin accessibility features critical for integration.

We believe these additions provide the necessary details for readers to understand and reproduce our preprocessing workflow, while also justifying our methodological choices from a biological perspective.

2. Harmony is one of the mainstream integration tools [2]. It has already been integrated into popular packages such as scanpy [3] and Seurat [4]. It is better to benchmark the performance of harmony.

Response: Thank you for your valuable suggestion to include Harmony in our benchmarking study. We have now incorporated Harmony into our comprehensive evaluation framework, assessing its performance across all three key aspects: integration accuracy, biological conservation, and batch effect correction. Figures 2, 3, and 4 in our manuscript have been updated to include both Harmony and our new FM-Adaptation approach (FM-scVI). Additionally, we have added detailed information about Harmony's characteristics to our supplementary methods summary table. This inclusion provides a more complete landscape of the available integration methods and strengthens the comprehensiveness of our benchmark study.

Added sentences:

[Section 4.3]:

- Harmony (0.0.10)[41] Harmony is an integration method that projects data into a shared embedding space by iteratively correcting for known batch effects, such as differences across modalities. In our implementation, we apply the harmonypy package to infer joint embeddings based on the PCA-reduced features of concatenated RNA and ATAC data, using modality labels as batch information.

[Figure Redacted]

Figure R17: Figure 2 in the manuscript

Table R5: Supplementary Table 2 in the manuscript

Supplementary Table 2: **Classification and other information of the 23 integration methods.** Methods details, including classification of methods, applicability of unpaired scenarios and Triple-omics integration, Programming Language, and so on.

Methods	Statistical-based	DL-based	DM or FM	Unpaired	Triple-Omics	Programming Language	Year	Reference
MOFA	✓		DM			Python	2018	[8]
MMD-MA	✓		DM			Python	2019	[9]
PCA	✓		DM	✓	✓	Python	2019	[10]
Harmony		✓	DM	✓		Python	2019	[11]
UnionCom	✓		DM			Python	2020	[12]
LIGER	✓		DM	✓	✓	R	2020	[13]
TotalVI		✓	DM			Python	2021	[14]
Seurat4	✓		DM	✓		R	2021	[15]
iNMF	✓		DM	✓	✓	R	2021	[16]
Cobolt		✓	DM	✓		Python	2021	[17]
scMDC		✓	DM			Python	2022	[18]
Pamona	✓		DM	✓		Python	2022	[19]
bindSC	✓		DM	✓		R	2022	[20]
scJoint		✓	DM	✓	✓	Python	2022	[21]
GLUE		✓	DM	✓	✓	Python	2022	[22]
Deepmaps		✓	DM			R	2023	[23]
Seurat5	✓		DM	✓		R	2023	[24]
scMoMaT	✓		DM	✓	✓	Python	2023	[25]
scVI		✓	DM	✓	✓	Python	2023	[26]
Geneformer		✓	FM	✓		Python	2023	[5]
UCE		✓	FM	✓		Python	2023	[27]
scGPT		✓	FM	✓		Python	2024	[28]
scFoundation		✓	FM	✓		Python	2024	[29]

Reference:

[1] M. D. Luecken et al., “Benchmarking atlas-level data integration in single-cell genomics,” Nat. Methods, vol. 19, no. 1, pp. 41–50, Jan. 2022, doi: 10.1038/s41592-021-01336-8.
[2] I. Korsunsky et al., “Fast, sensitive and accurate integration of single-cell data with Harmony,” Nat. Methods, vol. 16, no. 12, pp. 1289–1296, Dec. 2019, doi: 10.1038/s41592-019-0619-0.
[3] F. A. Wolf, P. Angerer, and F. J. Theis, “SCANPY: large-scale single-cell gene expression data analysis,” Genome Biol., vol. 19, no. 1, pp. 1–5, 2018.
[4] R. Satija, J. A. Farrell, D. Gennert, A. F. Schier, and A. Regev, “Spatial reconstruction of single-cell gene expression data,” Nat. Biotechnol., vol. 33, no. 5, pp. 495–502, 2015.

REVIEWER COMMENTS

Manuscript ID: NCOMMS-24-71130B

Manuscript title: SCMBench: Benchmarking Domain-specific and Foundation Models for Single-cell Multi-omics Data Integration

Blue: our response to the reviewer's comments<br><br>Black: reviewer's comments	Orange: original text<br><br>Green: revised sentences

Reviewer #1 (Remarks to the Author):

No further comments. The authors have done a nice job addressing all of my comments. The changes has improve the clarity of the paper. I note that the added installation guides and setup scripts that is now accompanying the code. I believe this will make the code much more accessible and user-friendly. Congratulation on a nice manuscript.

Reviewer #1 (Remarks on code availability):

The code was assess by another ECR reviewer

Response: We thank Reviewer #1 for their positive assessment of our revisions and for acknowledging the improvements in clarity. We are pleased that the added installation guides and setup scripts meet the reviewer's expectations for accessibility and user-friendliness. We appreciate the reviewer's thorough and constructive feedback throughout the review process, which has been invaluable in strengthening our manuscript.

Reviewer #2 (Remarks to the Author):

I co-reviewed this manuscript with one of the reviewers who provided the listed reports. This is part of the Nature Communications initiative to facilitate training in peer review and to provide appropriate recognition for Early Career Researchers who co-review manuscripts.

Reviewer #2 (Remarks on code availability):

The newly added installation instructions and setup scripts are clear and greatly improve the usability of the repository. Thanks.

Response: We thank Reviewer #2 for their time in co-reviewing this manuscript and for their positive remarks regarding the installation instructions and setup scripts. We appreciate the Nature Communications initiative to support Early Career Researchers through the co-review process, and we are glad that our revisions have improved the usability of the repository. We also thank Reviewer #2 for their contribution to the peer review process.

Reviewer #3 (Remarks to the Author):

I appreciate the authors' thoughtful revision and their partial resolution of my initial concerns. Here are my further comments on this revision:

1. I would not be a deal breaker, but I remain unconvinced by the way the authors leverage scFMs for integrating scATAC and scRNA data. While the authors justify this by claiming it represents the "maximum level of effort feasible for non-specialist users," this approach is clearly outside the intended scope of current scFMs, which are generally trained on scRNA data alone. Feeding scATAC data directly into these models risks severe out-of-distribution effects and undermines the integrity of the benchmarking. It is misleading to treat such usage as representative or valid for model comparison. At a minimum, the authors should explicitly state that this is not the official or recommended use of the models in question. They should also acknowledge and discuss alternative strategies, such as using scATAC-specific FMs (e.g., GET), projecting scATAC data to a shared latent space, or translating scATAC into scRNA-like profiles using methods like BABEL. Ignoring these possibilities gives an incomplete and potentially misleading picture of model performance.

Response: We thank the reviewer for raising this important concern. We fully agree that directly inputting scATAC data into scFMs trained on scRNA may lead to out-of-distribution issues and is not an ideal or recommended usage. In response, we have made the following clarifications and additional efforts:

1. **Clarification in manuscript:** We have explicitly stated in the revised manuscript that such usage is not the intended or officially recommended use of scFMs. The goal of including this setup was to simulate a realistic scenario where non-specialist users may attempt to apply FMs to different modalities due to ease-of-use or lack of available alternatives. We now clearly describe this as a non-standard usage scenario and discuss its limitations.

2. **Attempt to include scATAC-specific foundation models (FMs):** We attempted to incorporate GET into our benchmark. However, we encountered several limitations that made it infeasible to proceed:

    - **Lack of cell-level embeddings:** GET operates on pseudobulk chromatin accessibility aggregated at the cell-type level rather than individual cells. As stated in the GET paper: "To aggregate single cells into 'pseudobulk cell types', we used the Louvain clustering results from each study" and "The data were only presented in pseudobulked format" (Fu et al., Nature 2025). This design is insufficient for our benchmark, which requires cell-level latent representations for integration evaluation.

[Figure Redacted]

Figure R1: Screenshot from the GET paper. *Fu, Xi, et al. "A foundation model of transcription across human cell types." Nature 637.8047 (2025): 965-973*。

- **Extremely high storage requirements:** Even for a single dataset (PBMC 10x), one output file of a cell type exceeded 31 GB, and the full tutorial exceeded 286 GB in disk usage (see the screenshot below). This represents a significant technical burden for typical users.

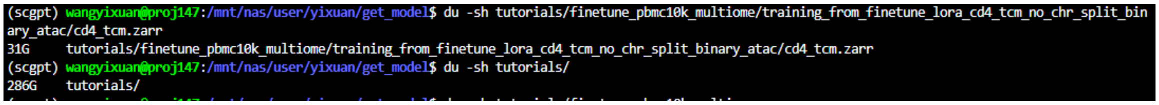

Figure R2: Attempts to run GET.

- **Model instability with real data:** We contact the GET authors, who state the model struggles to converge with more than 1,000 peaks, while our datasets contain tens of thousands of peaks per cell.

- **Software issues:** We encountered a bug during execution when running `https://github.com/GET-Foundation/get_model/blob/master/tutorials/finetune_pbmc.ipynb` (see the screenshot below). While the authors kindly responded, the above limitations prevented further testing.

```
---------------------------------------------------------------------------
AttributeError                            Traceback (most recent call last)
Cell In[21], line 8
      6 cfg.run.run_name='interpret_training_from_finetune_lora_cd4_tcm_no_chr_split_binary_atac' # the run name you want to l
      7 zarr_path = '/mnt/nas/user/yixuan/get_model/tutorials/finetune_pbmc10k_multiome/interpret_training_from_finetune_lora_
----> 8 hydra_celltype = GETHydraCellType.from_config(cfg, celltype=celltype,
      9                                               zarr_path=zarr_path)

File /mnt/nas/user/yixuan/miniconda3/envs/get/lib/python3.12/site-packages/gcell/cell/celltype.py:1990, in GETHydraCellType.fr
   1988 if prediction_target is None:
   1989     prediction_target = list(cfg.model.cfg.loss.components.keys())[0]
-> 1990 return cls(
   1991     celltype=celltype,
   1992     zarr_path=zarr_path,
   1993     prediction_target=prediction_target,
   1994 )

File /mnt/nas/user/yixuan/miniconda3/envs/get/lib/python3.12/site-packages/gcell/cell/celltype.py:1671, in GETHydraCellType.__
   1669 else:
   1670     self.num_cls = 1
-> 1671 self._process_data()
   1673 # Process jacobians if available
   1674 if "jacobians" in self._zarr_data:

File /mnt/nas/user/yixuan/miniconda3/envs/get/lib/python3.12/site-packages/gcell/cell/celltype.py:1698, in GETHydraCellType._p
...
   1703             self._zarr_data["preds"][self.prediction_target][
   (...)   1707         ]
   1708     )

AttributeError: 'GETHydraCellType' object has no attribute 'gene2strand'
```

Figure R3: Bug encountered when running GET.

3. **Inclusion of BABEL translation:** To address the reviewer's suggestion of translating scATAC into scRNA-like profiles, we additionally evaluated BABEL as an alternative preprocessing strategy. We compared FM performance using:

   (i) activity-score-transformed scATAC data (MAESTRO), and (ii) BABEL-translated scATAC data.

   We first validated that BABEL translation preserves biological information (Supplementary Figure 16). The gene-wise mean expression between RNA and BABEL-translated activity shows high correlation (Pearson r = 0.98, Supplementary Figure 16b), cell-level correlations have a median of approximately 0.70 (Supplementary Figure 16d), and the translated matrix maintains cell-type clustering structure (Supplementary Figure 16a) and cell-type-specific marker genes (Supplementary Figure 16c,e).

   We then systematically compared FM performance using MAESTRO versus BABEL preprocessing across all datasets (Supplementary Figure 17). The results indicate dataset-dependent effects: BABEL preprocessing improves integration accuracy on PBMC-10x and Chen-2019, while MAESTRO achieves better batch correction on Ma-2020 and Muto-2021. Overall, neither method consistently outperforms the other across all metrics and datasets, suggesting that the preprocessing choice should be considered alongside dataset characteristics.

We appreciate the reviewer's guidance in highlighting alternative strategies and have revised the manuscript to better reflect these complexities and limitations.

Revised Sentences:

[Section 2.1] Due to the token constraints of pre-trained FMs, which are primarily designed to process gene expression data, we cannot directly input scATAC-seq peak features. Instead, we transform chromatin accessibility data into a gene-centric format using a biologically informed approach implemented in the MAESTRO package [44]. This approach weights peak signals based on their distance to transcription start sites, reflecting the established relationship between chromatin accessibility and gene regulation. To ensure the validity of this transformation, we perform supplementary analyses on the PBMC-10x dataset (Supplementary Figure 1), confirming that the MAESTRO-derived activity matrix preserves key biological structure and gene-level correspondence with RNA. We additionally compare MAESTRO with alternative gene-activity derivation methods, including Seurat4 [21] and the cross-modal translation model BABEL [45]. The BABEL-translated activity matrix also demonstrates strong consistency with gene expression and provides comparable FM-compatible representations. Further comparisons of FM performance across datasets (Supplementary Figure 16–17) show that MAESTRO and BABEL each have dataset-dependent advantages and neither uniformly dominates. Together, these results indicate that transforming ATAC-seq data into activity matrices offers a reasonable and biologically grounded strategy for adapting chromatin accessibility profiles to FM processing, while multiple alternative implementations remain feasible and validated.

[Section 2.6] However, it is important to note that current scFMs are predominantly trained on scRNA-seq data, and their application to scATAC-derived inputs represents a non-standard usage scenario that may introduce out-of-distribution effects. We include this setup to simulate realistic use cases where non-specialist users may attempt to leverage FMs across modalities. For scATAC-seq data, we evaluate three preprocessing strategies: (i) calculating biologically meaningful gene activity scores from chromatin accessibility data using the regulatory potential model implemented in MAESTRO [44], which captures the relationship between chromatin accessibility and gene regulation; (ii) deriving gene activities using Seurat4, which provides a commonly used alternative implementation; and (iii) translating scATAC profiles into scRNA-like representations using BABEL [45], a cross-modal translation method. Validation analyses of the activity matrices derived from these strategies confirm preservation of cell-type-specific signals and biological clustering structure (Supplementary Figures 1, 16). Comparison of FM performance using MAESTRO versus BABEL preprocessing reveals dataset-dependent effects, with neither strategy consistently outperforming the other across all metrics (Supplementary Figure 17). These preprocessing strategies enable FMs to process multi-omics data while preserving biological interpretability.

[Section 4.1] To ensure fairness among methods requiring an activity matrix, we standardize the calculation using the AT ACCalculateGenescore from the MAESTRO library [44], following the implementation approach of DeepMAPS [43], while additionally evaluating alternative activity derivation strategies, including the GeneActivity module in Seurat4 [21] and the cross-modal translation outputs from BABEL [45], for completeness and consistency across methods.

[Discussion] Second, the field of FMs is rapidly evolving, with new pre-trained models continually emerging; our assessment represents a snapshot of currently popular models rather than an exhaustive evaluation. Due to the limited number of FMs designed to handle multi-omics integration tasks, comparing their zero-shot performance with specialized DMs may not be entirely fair. We include adapter-based integration strategies to partially address this limitation, though this represents just one possible direction for leveraging FMs in this domain and is not the primary focus of this work. Additionally, while scATAC-specific FMs such as GET [61] have recently emerged, they currently operate at the cell-type level rather than providing cell-level representations, limiting their applicability for single-cell integration benchmarking. Substantial further research will be needed to fully realize the potential of FMs in multi-omics integration.

[Caption of Supplementary Figure 16] Supplementary Figure 16: Validation of BABEL-translated activity matrix on the PBMC-10x dataset. a. UMAP visualization of the BABEL-translated activity matrix. b. Scatter plot comparing gene-wise mean expression between RNA and BABEL-translated activity (Pearson r = 0.98). c. Jaccard Similarity Index (JSI) of marker genes between RNA and BABEL-translated activity for each cell type. d. Distribution of cell-level Pearson correlation coefficients between RNA and BABEL-translated activity profiles (median around 0.70). e Heatmap of differentially expressed genes across cell types in the BABEL-translated activity matrix.

[Caption of Supplementary Figure 17] Supplementary Figure 17: Comparison of foundation model performance using MAESTRO versus BABEL preprocessing. Performance comparison of FMs applied to scATAC data preprocessed using MAESTRO (activity score transformation) or BABEL (cross-modal translation) across four datasets. Methods are labeled as BABEL FM or MAESTRO FM. Dot size indicates metric values; bar color indicates overall rank (darker = better). Results show dataset-dependent effects: BABEL preprocessing improves integration accuracy on PBMC-10x and Chen-2019, while MAESTRO achieves better batch correction on Ma-2020 and Muto-2021. Neither method consistently outperforms the other across all metrics and datasets.

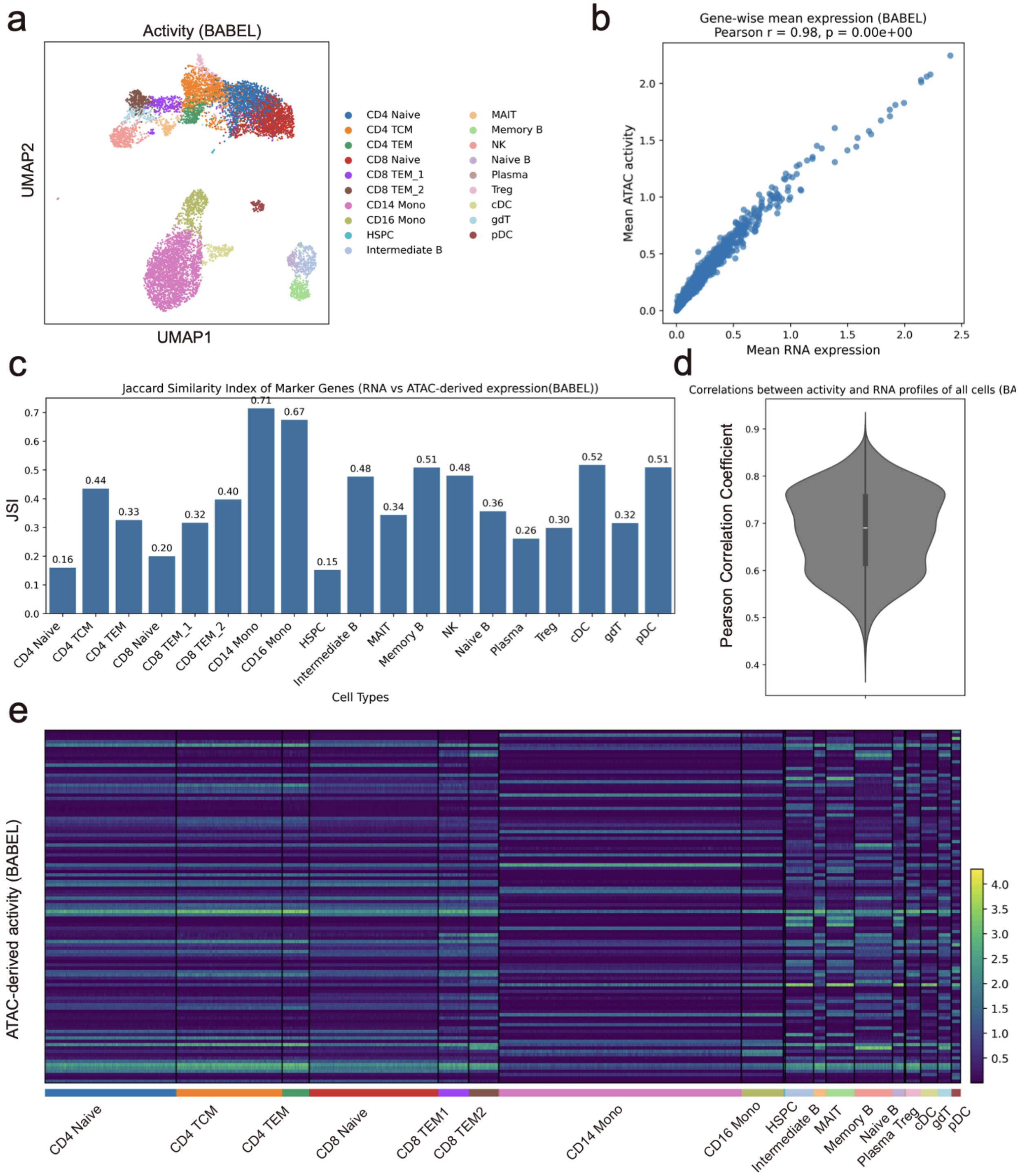

Figure R4: Supplementary Figure 16 in the manuscript

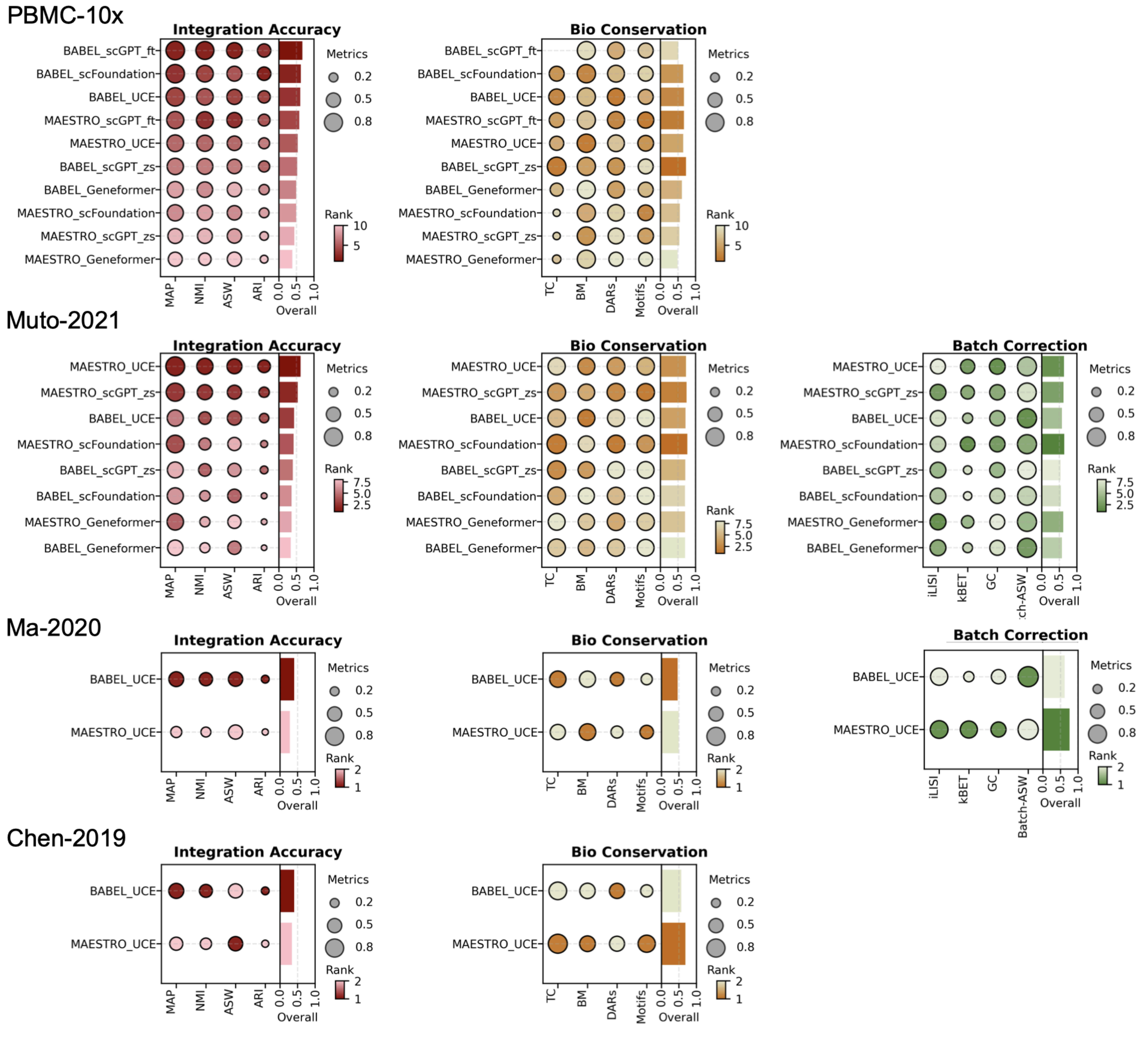

Figure R5: Supplementary Figure 17 in the manuscript

2. The terminology used throughout the manuscript is also problematic. For example, referring to model variants like scGPT-scVI and UCE-scVI as "FM-scVI" is overly broad and implies generality that does not exist. Given the number of foundation models developed in the field, including scBERT, scFoundation, and others. It is inappropriate to use such generic terms for a limited subset of models. Similarly, the phrase "FM-adaptation" is used to refer to a specific combination of scFM and scVI, yet the literature contains a variety of adaptation strategies such as full fine-tuning, partial fine-tuning, and parameter-efficient tuning. The authors' implementation reflects just one of many possible approaches and should be named accordingly. The current labeling overstates the scope and risks confusing readers.

Response: We appreciate the reviewer's concern about terminology precision, which is indeed important for clarity and avoiding confusion in the field. Our initial choice of "FM-scVI" was

intended to represent the best-performing foundation model combined with scVI, as selected from multiple FM candidates (e.g., scGPT, UCE, Geneformer) during our benchmarking process. This result was added in response to reviewer comments in the previous revision round. However, we acknowledge that this generic labeling may appear overly broad given the diversity of foundation models in single-cell genomics and could risk confusing readers.

To address this concern, we have made the following revisions:

1. **Replacing "FM-scVI" with explicit method names:** For dataset-specific results, we have replaced "FM-scVI" with specific method names such as "scGPT-scVI" and "UCE-scVI" throughout the manuscript, figures, and captions to clearly indicate which foundation model is being evaluated. For overall performance metrics aggregated across all datasets, we retain "FM-scVI" to denote the best-performing FM and scVI combination, as this varies across datasets and involves multiple foundation models. This convention is explicitly defined in the corresponding figure captions to ensure clarity.
2. **Clarifying "FM-adaptation" terminology:** We acknowledge that "FM-adaptation" was initially used to refer specifically to the combination of foundation models with scVI. However, as the reviewer correctly notes, the literature encompasses a variety of adaptation strategies, including full fine-tuning, partial fine-tuning, and parameter-efficient tuning. In our revised benchmarking, we have expanded our evaluation to include multiple adaptation strategies. Accordingly, we have revised the original sentence: *"To address this, we propose an FM+AdaptationLayer approach to enhance the performance of FMs in this task."* to: *"To systematically assess the potential of foundation models in multi-omics integration, we evaluate multiple adaptation strategies, including using scVI as an adaptation module for FM embeddings, alongside fine-tuning and parameter-efficient tuning approaches."*
3. **Figure and caption updates:** All relevant figure labels and captions have been updated to reflect the specific method names and adaptation strategies, ensuring consistency and clarity throughout the manuscript.

Revised Sentences:

[Introduction] To systematically assess the potential of foundation models in multi-omics integration, we evaluate multiple adaptation strategies, including using scVI as an adaptation module for FM embeddings, alongside fine-tuning and parameter-efficient tuning approaches.

[Section 2.2] Among FMs, scGPT and UCE demonstrate superior performance, with scGPT-scVI achieving results comparable to GLUE. Figure 2 e presents the overall paired performance across datasets.

Our adapter-based integration approach (e.g., scGPT-scVI) maintains computational efficiency similar to standard scVI, offering a significant advantage over direct FM fine-tuning.

FMs in zero-shot mode exhibit inferior performance compared to specialized deep learning methods, but proper fine-tuning and adapter-based integration strategies demonstrate

promising improvements in integration accuracy while maintaining reasonable computational efficiency.

[Caption of Figure 2] Figure 2: Benchmarking results of the integration accuracy. All metrics are the higher the better. A. and b. Benchmarking results on the paired and unpaired datasets. Metrics are categorized into three groups: integration accuracy (in red), bio-conservation (in yellow), and batch correction (in green). For dataset-specific results, FM-scVI denotes the specific foundation model combined with scVI:scGPT on PBMC-10x and the adapted UCE on Ma-2020. scGPT-ft refers to the fine-tuned version of scGPT, while scGPT-zero is the zero-shot version. c. Applicability of integration methods to unpaired data and availability of GPU acceleration for each method. d. Computational efficiency evaluated on the full PBMC-10x dataset, showing processing time and memory requirements across methods. e and f. Overall integration accuracy scores across all paired and unpaired datasets, respectively. For overall metrics, FM-scVI represents the best-performing foundation model combined with scVI, which varies across datasets. Datasets with batch information are split into smaller ones to minimize the impact of batch effects on the integration metrics. Methods are ranked according to the mean performance, and the middle line in each box represents the median score.

[Section 4.3] FM-scVI denotes scGPT-scVI when applied to human data and UCE-scVI when applied to mouse data, selected based on their respective performance advantages.

Our adaptation experiments show promise, with scGPT-scVI demonstrating competitive performance for NK cells in human data.

[Caption of Figure 3] Figure 3: Comparison of the cellular identity preservation performance of DMs and FMs through biomarker detection. a. Comparative analysis of biomarker detection performance across various computational methods. Heatmaps display the JSI for biomarker detection between methods, with darker red shades representing higher overlap. NK cells in PBMX-10x and ENDO cells in Muto-2021 are taken as examples. Methods are ranked according to their BM score. b-d. Method rankings based on average performance across datasets for the detection of (b) cell-type-specific biomarkers, (c) differentially accessible regions (DARs), and (d) enriched transcription factor motifs. These rankings highlight the relative effectiveness of each method in preserving biologically meaningful features. e. Box plot comparing overall biological conservation scores across methods. The conservation score is computed as the average detection consistency of cell-type-specific biomarkers, DARs, and enriched transcription factor motifs. The central line in each box represents the median score, with narrower boxes and shorter whiskers indicating greater robustness and consistency across datasets. For overall metrics (b–e), FM-scVI represents the best-performing foundation model combined with scVI on each dataset, comprising scGPT-scVI for human data and UCE-scVI for mouse data.

[Section 2.4] When examining unpaired settings (Fig. 4d), our adapter-based integration approach (FM-scVI) performs comparably to scJoint, further demonstrating that foundation models can be effectively adapted to multi-modal integration tasks through appropriate architectural modifications and training strategies, even in challenging unpaired scenarios.

[Caption of Figure 4] Figure 4: Comparison of biological dynamics preservation performance of DMs and FMs through trajectory alignment. a-b. PAGA representation of the ground-truth lineage, and UMAP visualizations of pseudotime inference and cell type annotations, derived from the integrated embeddings of the top 8 methods on PBMC-10x (out of 19 DMs and 4 FMs). The analysis focuses on B cell development. All subplots are arranged in descending order based on the TC score. c-d. Box plot comparison of trajectory conservation for (c) paired datasets and (d) unpaired datasets, where the depth of color corresponds to the performance level. For overall metrics, FM-scVI represents the best-performing foundation model combined with scVI on each dataset.

[Section 2.6] To effectively leverage FMs in this field and explore their potential across various application scenarios, we systematically evaluate multiple adaptation strategies, including adapter-based integration of FM embeddings, fine-tuning, and parameter-efficient tuning approaches. Among these strategies, adapter-based integration employs scVI as a lightweight adaptation module that transforms FM embeddings into more effective representations for single-cell multi-omics integration.

[Section 2.7] For cross-species analyses, UCE performs best among FMs, though adapter-based integration (UCE-scVI) offers improved performance while maintaining cross-species capabilities.

Given that FMs typically do not perform as well as specialized deep learning methods, we systematically evaluate multiple adaptation strategies, including adapter-based integration, fine-tuning, and parameter-efficient tuning. These evaluations provide practical guidance for enhancing FM performance and inform future model design. This method is designed to enhance the performance of FMs in this context and provides guidance for future model design. We have made all benchmark code, evaluation metrics, and our adapter-based integration implementation publicly available to facilitate method selection and future benchmarking efforts.

[Discussion] FMs in Multi-omics Integration. While FMs have demonstrated transformative potential in other fields, their capabilities in single-cell biology remain underexplored. Our benchmark results, alongside published literature, suggest that current single-cell FMs have not yet been adequately harnessed for this domain. Despite lower overall integration accuracy compared to specialized DMs, FMs demonstrate unique strengths in versatility and efficiency. Their versatility enables adaptation across diverse single-cell tasks with the same architecture, while their efficiency advantages include both reduced training requirements and computational feasibility on large-scale datasets where many specialized DMs become computationally prohibitive or fail to complete. However, their struggle with multi-modal integration in the zero-shot setting underscores the necessity for appropriate adaptation strategies when handling different data modalities. Most FMs are predominantly trained on human data, leading to cross-species bias when generalizing to other organisms. Even UCE, which attempts to address this by training on diverse tissues and species, suffers from data imbalance issues in its training atlas. The exceptional performance of UCE on mouse developmental data suggests that species-specific pre-training may confer advantages for certain biological contexts. To address these limitations while leveraging FMs' rich biological knowledge, we systematically evaluate

multiple adaptation strategies. Among these, adapter-based integration combines FM embeddings with scVI's variational autoencoder framework. We implement these adaptations as standalone methods and incorporate them into our comprehensive benchmarking pipeline, evaluating them alongside traditional deep learning methods across all metrics and datasets. Our results demonstrate that this strategy effectively bridges the gap between FMs' biological knowledge and the specific requirements of multi-omics integration tasks, showing particular improvements in biomarker detection and trajectory conservation while maintaining computational efficiency. However, further refinement and validation are necessary to fully realize its potential.

We include adapter-based integration strategies to partially address this limitation, though this represents just one possible direction for leveraging FMs in this domain and is not the primary focus of this work.

[Figure Redacted]

Figure R6: Revised Figure 2a in the manuscript

[Figure Redacted]

Figure R7: Revised Figure 2b in the manuscript

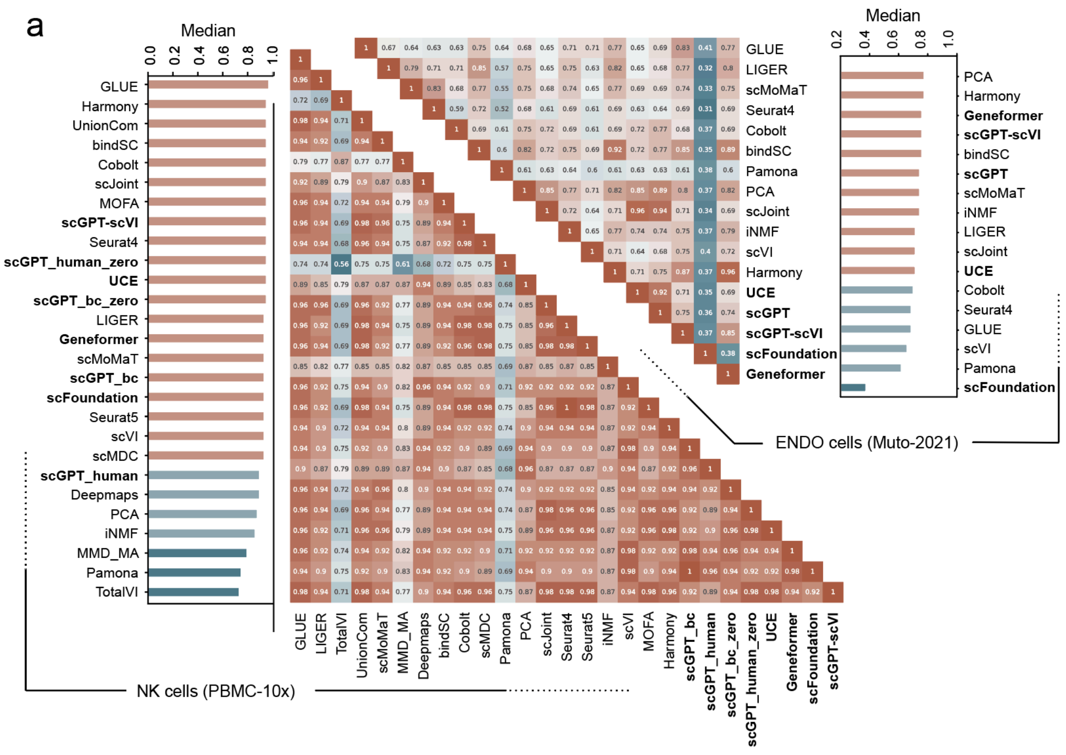

Figure R8: Revised Figure 3a in the manuscript

We thank the reviewer for highlighting this important issue, and we believe these revisions improve the precision and interpretability of our presentation.

3. On page 6, the authors state that “architecture itself plays a crucial role in multi-omic integration,” but this statement is vague and unsupported. It is unclear whether the authors are suggesting that untrained model architectures can integrate modalities effectively, or that pretraining contributes little to integration performance. Either way, this claim requires further justification and possibly empirical support. Without clarification, the statement feels speculative and unsubstantiated.

Response: We thank the reviewer for pointing out the imprecise language in our discussion. We agree that our statement about architecture's role was overstated, given our current evidence.

We revised this section to more accurately reflect our findings. Instead of claiming that "architecture itself plays a crucial role," we rephrased to focus on what our data actually demonstrates: “Interestingly, our analysis reveals that pretrained weights did not provide substantial benefits compared to training from scratch, suggesting that for this specific multi-omics integration task, the benefits may primarily come from the model's capacity to learn task-specific representations during fine-tuning rather than leveraging pretrained knowledge.”

This revision removes the speculative claim about architecture's role and instead presents a more conservative interpretation that is directly supported by our experimental observations. We also ensure that all conclusions are clearly tied to our empirical findings without overgeneralization.

4. On page 8, the authors note that scFoundation is surprisingly faster than smaller models. This is counterintuitive and should be explained.

Response: Thank you for pointing out this counterintuitive observation. Upon reviewing our measurement methodology, we discovered that our initial runtime recording for scFoundation inadvertently captured only the RNA modality processing time, while other methods correctly recorded the full multi-modal (RNA + ATAC) integration time. After correcting this measurement error and recording the complete multi-modal processing time for scFoundation, the results now show the expected pattern where larger models require longer runtime. We have also double-checked that all embedding-based methods consistently record the full multi-modal processing time. The corrected runtime values are included in the revised manuscript.

Revised Sentences: We sort the results by CPU processing time, revealing that computational demands generally scale with model complexity.

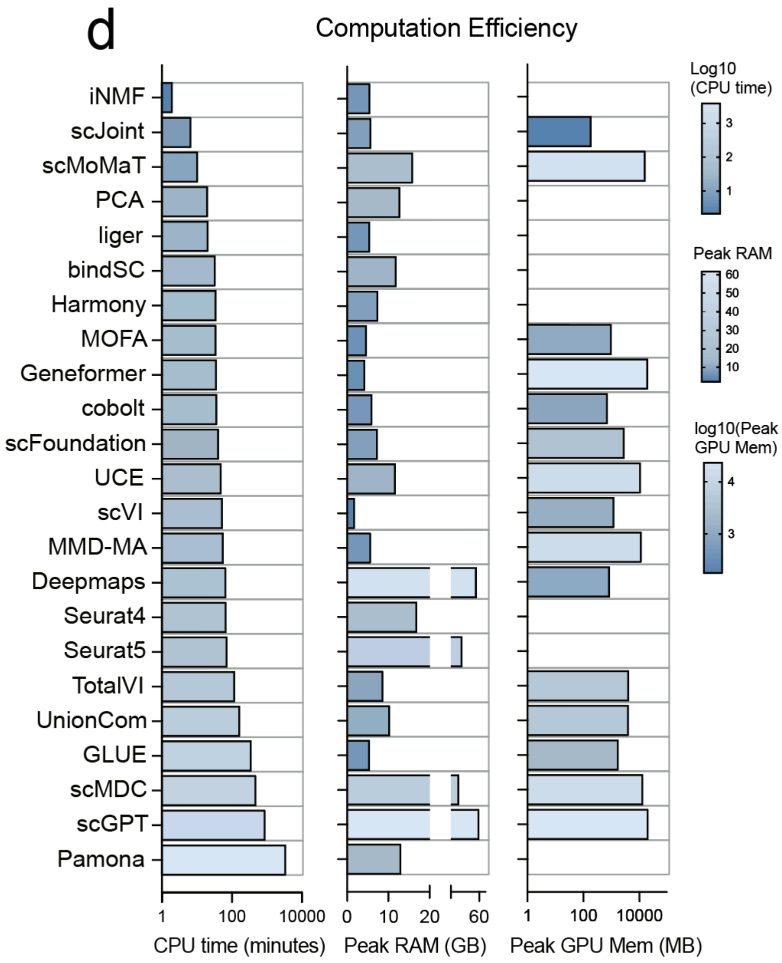

Figure R9: Revised Figure 2d in the manuscript

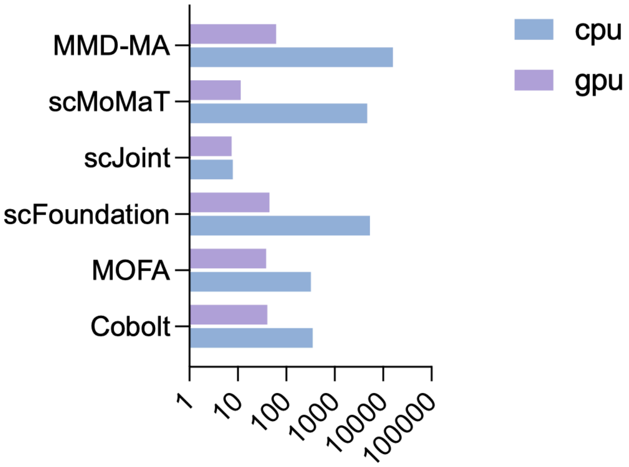

Figure R10: Revised Supplementary Figure 15 in the manuscript

5. There is also a structural issue with the organization of the manuscript. The proposed adaptation framework is only explained in Section 2.6 and Figure 6, yet it is invoked much earlier in the Results section. This disrupts the logical flow and makes it difficult for readers to follow the methodology. The explanation of the adaptation approach should be introduced earlier, before its application is described.

Response: We thank the reviewer for this suggestion. We note that the adaptation framework was already introduced in Section 2.1 (Overview), where we described the evaluation of FM-based approaches alongside other integration methods. However, we acknowledge that this initial description could benefit from additional detail to improve clarity for readers.

In the revised manuscript, we have expanded the description in Section 2.1 to more explicitly introduce the adaptation strategies: "To comprehensively evaluate FMs for multi-omics integration, we evaluate multiple adaptation strategies, including adapter-based integration that combines FM embeddings with scVI's framework, fine-tuning, and parameter-efficient tuning approaches. We implement these adaptations as standalone methods and benchmark them alongside other integration methods throughout our pipeline (detailed implementation in Section 2.6)."

This revision provides readers with a clearer understanding of the adaptation framework before encountering its application in subsequent results sections, while Section 2.6 continues to offer detailed implementation and in-depth analysis.

6. I was surprised to find that some AI-generated text appears in the manuscript. For instance, “Retry Claude can make mistakes. Please double-check responses.” on pages 13 and 16. Such artifacts should have been removed during basic proofreading.

Response: We sincerely apologize for this oversight. These artifacts were inadvertently left in the manuscript during the drafting process and should have been caught during proofreading. We have thoroughly reviewed the entire manuscript and removed all such instances in the revised version. We have also strengthened our proofreading procedures to prevent similar issues in the future. We thank the reviewer for bringing this to our attention.

Reviewer #4 (Remarks to the Author):

The authors have thoughtfully addressed the majority of concerns raised in my initial review. The revised manuscript now offers a significantly more comprehensive evaluation of multi-omics integration methods, with notable improvements in the assessment of scATAC-seq-related metrics. Key enhancements include:

1. Epigenomic evaluation through differential accessibility regions (DARs) and motif enrichment analysis, providing crucial validation beyond transcriptomic assessments.
2. Improved scATAC-seq processing with a detailed demonstration of gene activity matrix pre-processing for foundation models, directly addressing prior reviewer concerns.

3. More reasonable claims regarding foundation model (FM) performance, now including a discussion of their trade-offs.

The authors have successfully resolved the major issues raised by Reviewer #4, resulting in a more rigorous, reproducible, and biologically insightful benchmark.

Remaining Question:
Why were foundation models (FMs) excluded from the triple-omics integration benchmark?
Response: Thank the reviewer for the comment. We excluded FMs for the methylation modality because snmC-seq yields continuous β-values between 0 and 1, whereas current scRNA-trained FMs are designed for sparse, over-dispersed count data—this distributional mismatch made them unusable in pilot tests. We have clarified this point in the revised manuscript.

Added Sentence: (Section 4.1) FMs are excluded due to the distributional mismatch between continuous methylation β-values and the sparse count data these models are trained on.

Reviewer #4 (Remarks on code availability):

The repository appears to be well-maintained, featuring a comprehensive README document that provides clear guidance. While I have reviewed the relevant code sections related to my review comments via the online repository, I did not perform a local installation or execute the code. As such, I cannot verify or guarantee the functional correctness of the implementation.

Response: We thank the reviewer for examining the repository and for their positive remarks on the README documentation and overall organization. We understand that a full local installation was not performed, and we appreciate the reviewer's transparency in this regard. We remain committed to maintaining the repository and addressing any issues that users may encounter.
